# Patient preferences for Interferon-beta in Iran: A discrete choice experiment

**DOI:** 10.1371/journal.pone.0193090

**Published:** 2018-03-28

**Authors:** Farimah Rahimi, Hamid Reza Rasekh, Ezatollah Abbasian, Farzad Peiravian, Masoud Etemadifar, Fereshteh Ashtari, Ali Mohammad Sabzghabaee, Mohammad Reza Amirsadri

**Affiliations:** 1 Health Management and Economics Research Center, Isfahan University of Medical Sciences, Isfahan, Iran; 2 Department of Pharmacoeconomics and Pharmaceutical Management, School of Pharmacy, Shahid Beheshti University of Medical Sciences, Tehran, Iran; 3 Department of Economics, Bu Ali Sina University, Hamedan, Iran; 4 Department of Neurology, Medical School, Isfahan University of Medical Sciences, Isfahan, Iran; 5 Isfahan Clinical Toxicology Research Center, Isfahan University of Medical Sciences, Isfahan, Iran; 6 Department of Clinical Pharmacy and Pharmacy Practice, School of Pharmacy and Pharmaceutical and Health Management and Economics Sciences, Isfahan University of Medical Sciences, Isfahan, Iran; Klinikum rechts der Isar der Technischen Universitat Munchen, GERMANY

## Abstract

Multiple sclerosis is a chronic, progressive, and common disease affecting the central nervous system in young adults. Interferon-beta is one of the most widely used medicines to reduce the disease progression. Given the variety of drugs in this category, we aimed to identify the preferences of patients for IFN-β that play an important role in policymaking in this area. Discrete choice experiment method was used in the present study to identify and prioritize those attributes that are of interest to MS patients and increases the utility of the use of IFN-β in their treatment. Questionnaires were given to 358 patients in Isfahan-Iran, who were asked to choose between the two treatment choices in each scenario. The results of the logit model showed that the changes in the efficacy lead to the most changes in the patient utility. Changes in side effects and ease of injection have been placed in the next rankings. Considering the drug attributes considered more desirable by patients can lead to greater medication adherence and possibly better treatment outcomes. Also, pharmaceutical companies, the health ministry, the Food and Drug Administration, insurance organizations, and neurologists can benefit from this information in production and importation, policymaking, and prescription.

## Introduction

Multiple sclerosis (MS) is a complex, chronic, progressive, and prevalent disease affecting the central nervous system, in which myelin and axons link is impaired [1,2]. This disease is more prevalent in young adults (30 years) and in women rather than men [3]. According to international statistics published by the Multiple Sclerosis International Federation, the number of people with MS has increased from 2.1 million in 2008 to 2.3 million in 2013 [4]. The study for prevalence estimation of MS in Iran in 2013 indicated a high prevalence rate in Isfahan (89 per 100000 of population) and Tehran (88/100000), and the point prevalence of MS was 101.39 per 100000 populations in 2014 [5]. The primary goals of MS treatment are to restore body function after a relapse, preventing new relapse and disability. One of the drug therapies used for MS disease is Interferon-beta (IFN-β), which has proved to be effective in reducing the relapse rate, disability progression, and disease progression[6].

In recent years, more attention has been paid to studies assessing the preferences of people in healthcare interventions because it is believed that understanding patient preferences and involving them in clinical decision-making can lead to improved patient satisfaction with the treatment, thereby increasing the adherence to treatment. On the other hand, information on patient preferences can be useful for healthcare prescribers, insurance organizations, pharmaceutical industry, and policymakers responsible for developing guidelines[7]. One of the ways to evaluate patient preferences is to use the conjoint analysis in order to extract stated preferences[8].

Many studies have been conducted by using methods of extraction utility in recent years—particularly studies that have used this method to implement programmes and services in healthcare and medicine sectors[9–12] or studies that have specifically investigated certain diseases such as cancer[13–15], diabetes[15–18], rheumatoid arthritis [19] and etc. In MS, patient preferences have not been overlooked, and various studies have focused on patient preferences by using choice-based conjoint methods, particularly in recent years. Most of the studies have investigated patient preferences towards the disease-modifying treatment[20–23], or selection of disease modifying drugs (DMDs)-related injection devices[24], or a choice between oral medication or injections[25], and analyse the preferences associated with the intended attributes discussed.

Since MS patients should use IFN-β on a routine basis throughout their lives to control their disease, it is essential to consider their desires towards drugs to promote services provided to them. To reach this goal, their desires should be considered. In this study, discrete choice experiment was used to quantify patient preferences. Since there is no study on the drug attributes affecting MS patient utility in Iran, it is essential to identify and prioritize such attributes in IFN-β products. The aim of this study was to evaluate the significance of attributes considered for IFN-β from MS patients’ perspective in Iran.

## Materials & methods

Considering that Isfahan province is located in a mysterious area in terms of sharp rise in the prevalence of MS during the last decade in Iran,[26] the population of this study included MS patients monitored by the Isfahan MS Society (IMSS). Inclusion criteria included having MS disease, history of IFN-β use, patients’ willingness to participate and at least 15 years old. This study was reviewed and approved by Institutional Ethics Committee of the Faculty of Pharmacy and Nursing, Midwifery of Shahid Beheshti University of Medicine Science before the study began and participants have a full consent and the right to withdraw existed during the interview. In Cochran's formula by considering the error rate of 5% and statistical population (5260 patients according to the latest statistics of the number of patients registered in the MS Society of Isfahan), the number of samples required was estimated about 358 patients. The samples were randomly selected from MS patients referred to the Isfahan MS Centre and two clinics located in Kashani and Al-Zahra Hospitals (that they are two major centres of MS are affiliated to the University of Medical Sciences) from December 2015 to May 2016. Questioners were blinded about different scenarios and they explained the study, and after approving the patients' satisfaction for enter the study, they asked questions according questioner without any directing answers. Questionnaires that did not go on until the end, or people refused to give more information, or were left with problems in choices (about 40 questionnaires) did not enter the study.

In this study, conjoint valuation method was used to determine and measure MS patient preferences for IFN-β in Isfahan province. Five steps are considered to complete this study in accordance with the literature as well as the guide published by the ISPOR (International Society For Pharmacoeconomics and Outcomes Research) on the use of conjoint studies in the healthcare field[8,15,27–29]: identifying attributes, determining levels for attributes, scenario selection, use of preferences and data analysis. According this guideline, six attributes of IFN-β were selected based on available published studies, and after opinion-polling experts (experts in pharmacoeconomics, neurology, and clinical pharmacy) the attributes related with Interferon-beta are available in Iran’s market. Also, owing to the lack of adequate comparative studies on the selected attributes of drugs in this category and heterogeneity between physicians in the field, qualitative data rather than quantitative data is used to determine the levels of all attributes—except prices of the medicines. The selected attributes and levels include: the country producing the IFN-β that includes two levels, namely imported interferon or the one produced in Iran. The monthly cost of the interferon, which is divided into three levels, is based on the range 0 to 231 dollars. Administration and injection frequency includes three following of levels: muscular injection (once a week), subcutaneous injection (three times a week), and subcutaneous injection (every other day). Drug efficacy that refers to a reduced frequency of relapses, the disease progression and disability progression has two levels including moderate and high. Low and medium levels were considered for drug side effects that include common symptoms including flu-like symptoms and skin reactions at the injection site. Finally, two levels were considered for the ease of the use variable that includes an easy level, which means preparation of the syringe and lack of the need for pre- injection preparations, and the difficult level reflects drug preparation prior to injection by the patient or PWID (persons who inject drugs). The combination of these attributes and levels make up scenarios for the patients.

Considering the number of attributes and levels, among possible scenarios in the full factorial design (2^4^ × 3^2^ = 144), a total of 12 scenarios (24 paired profiles) were selected by using the fractional factorial and D-efficient methods in JMP software to enhance the response quality. In each scenario, there are two choices under choice A and choice B—similar to the example shown in Table 1—were determined in pairs. The patients were later asked to choose one of these IFN-β types. Then, the intended questionnaire was prepared in three parts. The first part included demographic and socio-economic characteristics, the second part was related to the disease status and the treatment procedure and finally, the third part included the scenarios presented in the present study. Since the questionnaires were completed by the patients in the study pilot, and desired results were not achieved due to failure to answer all questions, high missed data and low Cronbach's alpha coefficient (0.43), the questions were completed by using face-to-face interviews while patients were in the waiting room to visit physicians.

**Table 1:** Example of scenarios in a questionnaire designed to elicit preferences of MS patients.

*Scenario 11*	*A*	*B*
Interferon—beta	Iran	Other countries
Injection	Subcutaneously- every other day	Subcutaneously- three times a week
Cost per month	132–232$	0–33$
Effectiveness	High	Moderate
Side effects	Moderate	Low
How to use	Easy	Hard
Choice:	A□	B□

In order to validate the estimated model and to evaluate the results, three tests were used[30]: 1) The whole model test, which is similar to variance table analysis and evaluates the overall fit of the model to remove all effects of repressors, except the intercept parameters. In this model, negative log-likelihood and sum of squares are similar to chi-square test and F test. 2) The lack of fit test (goodness of fit) seeks to answer the question of whether the variables in the current model are sufficient or more complex phrases should to be added. 3) The Wald test to assess the significance of the coefficients in the logit model.

## Results

Data analysis related to individual, socio- economic characteristics and the experiences of MS patients was performed by using SPSS (ver.16.0). The results of the descriptive statistics of the variables showed that most participants who suffered from the disease, on average, for 7.7 years are aged between 30–40 years. Also, disease has been diagnosed in almost half of these patients aged between 20–30 years. Moreover, the female population accounted for 79% of the participants, which was almost 3.7 times the number of men. On the other hand, 68% of the patients are married, and diploma and BA (Bachelor’s degree) patients accounted for 36.87% and 28.21%, respectively. Since most patients were female high school graduates, it was highly expected that almost 47% of them would be housewives. Among the samples of this study, 65% of patients currently use IFN-β as the main treatment. More information on these variables is shown in Table 2.

**Table 2:** Characteristics of the 358 MS patients.

Characteristic	Category	Frequency	Percent
Age	<20	10	2.79
	[20–30)	90	25.14
	[30–40)	159	44.41
	[40–50)	80	22.35
	[50–60)	15	4.19
	≥60	4	1.12
Gender	Female	282	78.77
	Male	76	21.23
Marital status	Never married	97	27.09
	Married	244	68.16
	Separated	17	4.75
Education	High School or less	68	18.99
	Diploma	132	36.87
	Associate Degree	34	9.50
	Bachelor’s Degree	101	28.21
	Master's Degree	20	5.59
	PhD	3	0.84
Employment status	Employed	66	18.44
	Self-employed	57	15.92
	Retired	3	0.84
	Home duties	167	46.65
	Student	31	8.66
	Unemployed	34	9.50
Current treatments	AVONEX® (IFN-β 1A 30 MCG, muscular- once a week)	4	1.12
	ACTOVEX® (IFN-β 1A 30 MCG, muscular- once a week)	24	6.7
	CINNOVEX® (IFN-β 1A 30 MCG, muscular- once a week) *	77	21.51
	REBIF® (IFN-β 1A 44MCG/0.5ML, Subcutaneously- 3 times a week)	17	4.75
	ACTORIF® (IFN-β 1A 44MCG/0.5ML, Subcutaneously- 3 times a week)	11	3.07
	RECIGEN® (IFN-β 1A 44MCG/0.5ML, Subcutaneously- 3 times a week) *	34	9.5
	BETAFERON® (IFN-β 1B 8MIU, Subcutaneously- every other day)	26	7.26
	EXTAVIA® (IFN-β 1B 8MIU, Subcutaneously-every other day)	1	0.28
	ACTOFERON® IFN-β 1B 8MIU, Subcutaneously-every other day)	4	1.12
	ZIFRON® (IFN-β 1B 8MIU, Subcutaneously-every other day) *	34	9.5
	No medical treatment.	14	3.91
	Oral medications	50	13.97
	Injectable drugs other than IFN-β	62	17.32

* Local bio-similar Interferon β manufactured by CinnaGen Company.

Also, the discrete choice experiment was done by using the logit model in the software JMP® 10.0.0. Data extracted from 358 questionnaires were analysed by using the nominal logistic model in the form of binary choices. The results of the logit model are specified in Table 3. The probability level for all variables, other than the manufacturing country variable, is less than 0.05 and so it can be concluded that the null hypothesis, which suggests that the estimated coefficients are equal to zero for other variables of the model, is rejected. Thus, rejecting the null hypothesis, the variables are significant variables in the model and the only the hypothesis suggesting that the value of the manufacturing country variable is equal to zero cannot be rejected.

**Table 3:** Patient preference for INF-β for each attribute and level in DCE analysis.

Attributes	levels	Estimate	Lower 95%	Upper 95%	Std Error	L-R ChiSquare	DF	Prob>ChiSq
Manufacturing Country	Iran	-0.123	-0.303	0.056	0.0915	1.829	1	0.1763
	others							
Monthly costs	0–33$	-0.005	-0.006	-0.004	0.0005	100.346	1	< .0001*
	33–132$							
	132–231$							
Administration and frequency	Muscular- once a week	-0.395	-0.535	-0.257	0.0709	32.910	2	< .0001*
	Subcutaneously- 3times a week	0.218			0.0740			
	Subcutaneously-every other day							
Effectiveness	Moderate	1.138	0.074	0.364	0.1073	115.960	1	< .0001*
	High							
Side effects	Low	-0.929	0.929	1.349	0.0800	141.568	1	< .0001*
	Moderate							
Ease of injection	Easy	-0.694	-1.086	-0.773	0.0875	65.657	1	< .0001*
	Hard							

* statistically significant with 95% confidence

Logit models usually do not directly interpret coefficients, and the only interpretation indicates their significance and relative size. According to information obtained from the logit model, the biggest factor belongs to the efficacy variable. It means that patients with MS attach more importance to changes in the efficacy level than in other parameters. On the other hand, the significance of the symptoms of these attributes of these features is also one of the crucial points in the model analysis. In the obtained model, only the efficacy variable is positive, which means that with an increase in the efficacy of drug, the patient is more likely choose it. Also, the negativity of the price coefficient indicates that as the price increases, the utility of patients decreases and the drug is less likely to be selected by patients. Similarly, the increase in the side effects and difficult injection leads to a decrease in patient utility and reduces the probability of being selected by patients.

**Fig 1:**
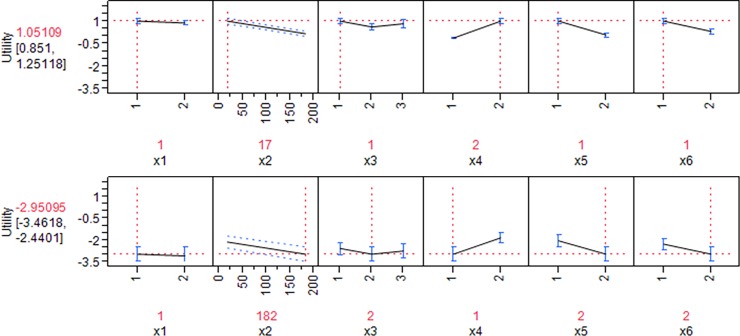
Relative importance of each attribute in the selection of the drug by the patients.

The relative importance of each attribute is obtained by dividing the utility range (the difference between the highest and lowest utility) for one attribute by the total differences of utility that is expressed as percentage.[22] The relative importance refers to the effect of each attribute on its probability to be selected by the patients. As has been shown in Fig 1, the highest relative importance was obtained for efficacy variable (20.91%), the manufacturing country (17.87%), and ease of injection (17.07%). In other words, the efficacy rate, the manufacturing country, and the ease of injection are respectively the most three important variables affecting patient preferences towards drugs. Notably, the relative importance of the manufacturing country is high, whereas this variable is not significant in the logit model. This means that despite patients’ attention to the manufacturing country in their choices generally, the utilities of Iranian and foreign drug were not significantly different from each other.

**Fig 2:**
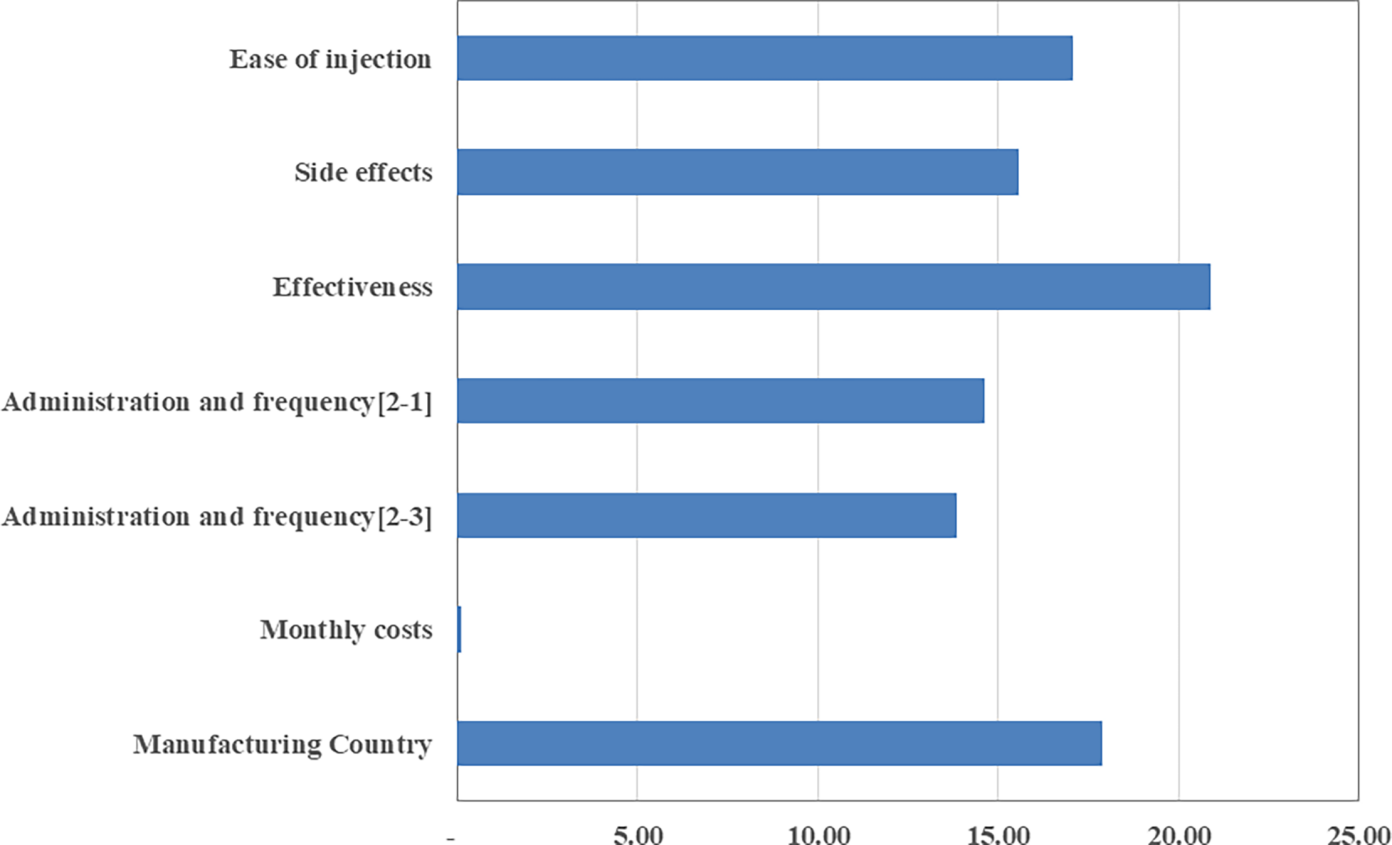
Minimum and maximum utility of MS patients for different attributes and their levels in two extreme states.

Generally, the greatest amount of utility in MS patients is obtained as follows: free IFN-β, muscular injection once a week, high efficacy, low side effects, and easy injection. As can be seen, this is the most ideal mode for a drug and utility obtained, which, in this case. is equivalent to 1.05 util. Unlike the maximum amount of utility, the minimum amount of utility in MS patients is obtained as follows: the highest price of the drug, subcutaneous injection three times per week, the lowest efficacy, the highest side effects and difficult injection—all these reduce patient utility by -2.95 util. These two limits are shown in the Fig 2. So, the change in utility can be predicted by considering changes in the levels of each variable.

Chi-square value and the resulting probability of the whole model test (271.0071 and <0001) reflect the overall significance of the whole estimated logit regression. The results of the lack of fit Test, in which the values are obtained for the chi-square and its likelihood (252.5827 and <0001), also showed that there are enough variables in the current model and there is no need to add new variables. In the Wald test, which was used to assess the significance of the coefficients in the logit model, probability value of less than 5% was obtained for all the coefficients, which indicates that the calculated value of chi-square statistics was placed on the rejection of the null hypothesis. Hence, it can be concluded that all the coefficients in the logit model are non-zero.

## Discussion

This study sought to evaluate and estimate the preferences of MS disease patients towards the attributes intended for IFN-β drugs by using the DCE method in Isfahan, Iran. Attributes such as the manufacturing country, monthly cost, administration and frequency of injection, efficacy, side effects and ease of use were analysed by using the logit model. In case of the manufacturing country, as mentioned in the results section, there was no significant difference between patient preferences for interferon produced by domestic and foreign pharmaceutical firms. But the remarkable point is that this attribute became significant when the recent treatment (IFN-β or other medicines) was entered into the model. It means that patients who currently use IFN-β, differentiate between drug made by Iranian and foreign companies in terms of desirability; but there was no significant difference for other patients. On the other hand, according to the findings obtained by Shah (2016), the manufacturing country was considered as a less important item compared quality, price, and availability of healthcare. In other words, the concept of ‘Made in’ was referred to as positive or negative effects on the customer's mind regarding the manufacturer or producer country and affects his/her decision-making process. It means that, the country of origin, as an external stimulus in the customer's mind, affects his/her perception of quality.[31] Therefore, the manufacturing country variable shows its effect while being used with other variables and is not significant by itself.

Monthly costs for the IFN-β drug have a negative impact on patient preferences. Obviously, the higher the prices, the more reduction in patient utility occurs. It should also be noted that considering the relative importance of a low coefficient of this variable, patients show more sensitivity to other aspects of this treatment such as efficacy and side effects than its price. The sign of price or cost is negative in studies[20,21,24], where they are considered as an attribute, which is consistent with our findings.

In most studies, the manner of administration refers to choose between oral and injectable medicines. The findings show that although oral administration of the drug is preferred over injection, the latter is preferred with increasing frequency of use of pills.[22] However, several relevant studies have shown that as frequency of injections decreases, patient utility also increases and the number of injections is considered as an important factor affecting patient preferences.[20–22] However, there has been no study that compares subcutaneous injection and muscular injections—especially in relation to IFN-β treatment.

Several studies have shown that when the efficacy variable exists among other attributes, this attribute will play a key role in patient preferences.[15] Similarly, the efficacy variable is of particular importance in preferences of MS patients.[24] On the contrary, side effects are also considered as other important attributes in the above studies and side effects, such as flu-like symptoms and skin reactions at the injection site, have been considered as the most common symptoms.[21] The findings of the present study and other similar studies [20–23,25] show that patients limit their preferences for the type and frequency of injections and prices, among others, by indicating the efficacy and side effects. It means that the efficacy and side effects play the most major role in increasing patient preferences, and other attributes are shown as significant and insignificant besides these two attributes.

With regard to the ease of injection, it can also be said that the injection device also plays an important role in patient preferences.[24] In other words, preparing syringes for IFN-β, instead of preparing it by the patient or a PWID prior to injection, leads to a higher level of utility. Generally, employing and paying attention to the attributes of the highest relative importance from the patient’s perspective is one of the major issues affecting healthcare—particularly medicine treatment. Therefore, attempts were made in the present study to identify and rank these attributes by using MS patient preferences. Considering that each study has its own limitations, this study’s limitations included access to MS patients, being honest responses, expose patients to various scenarios, among others. However, attempts were made to achieve even better possible answers by making close relationships with patients and spending more time with them. Also, in this study, relevant ethical considerations and the principle of confidentiality of information were considered, and the objectives of this project were explained to patients at the beginning of interviews and they participated in the study voluntarily and could withdraw from the study at the time of the interview.

## Conclusion

Evaluation of MS patient preferences on the IFN-β treatment will play a very important role in the treatment of patients because their acceptance of and adherence to the treatment procedure can lead to more effective treatment of chronic diseases. Although everybody recognizes the importance of attributes, such as efficacy and side effects of the treatment, quantifying such attributes and considering MS patient preferences on injection drugs can be effective and efficient for all stakeholders of the field, including manufacturers and importers of medicines, Food and Drug Administration, physicians, and insurance organizations.

## Supporting information

S1 Table The choice of MS patients. The choices between 12 scenarios by 358 Multiple Sclerosis patients.
(PDF)
