# Supplementary material for: Patient preferences for Interferon-beta in Iran: A discrete choice experiment
Source: PLoS One. 2018 Mar 28;13(3):e0193090. doi: 10.1371/journal.pone.0193090 (PMC5873931; doi:10.1371/journal.pone.0193090)
Supplement: S1 Table — The choices between 12 scenarios by 358 Multiple Sclerosis patients. (PDF) [file pone.0193090.s001.pdf]

Respondent	Choice Set	Response Indicator	X1	X2	X3	X4	X5	X6	Utility Formula
1	1	0	other	5.5	subcutances other days	high	low	hard	-0.005630885
1	1	1	other	0.5	mascular once a weak	medium	medium	hard	-0.900801637
1	2	0	other	0.5	subcutances three times a weak	medium	medium	hard	-1.116469982
1	2	1	other	5.5	mascular once a weak	high	low	easy	0.224832577
1	3	1	iran	5.5	mascular once a weak	medium	medium	hard	-0.878555459
1	3	0	iran	2.5	subcutances three times a weak	high	low	hard	-0.311436646
1	4	1	other	0.5	mascular once a weak	medium	low	hard	-0.384415273
1	4	0	other	2.5	subcutances three times a weak	high	medium	easy	-0.54728851
1	5	1	iran	0.5	subcutances other days	high	low	hard	-0.227088383
1	5	0	iran	2.5	mascular once a weak	medium	medium	easy	-0.737692999
1	6	0	other	2.5	subcutances other days	high	medium	hard	-0.562083627
1	6	1	iran	0.5	subcutances three times a weak	medium	low	easy	-0.518760439
1	7	0	other	0.5	mascular once a weak	high	medium	hard	-0.594334463
1	7	1	iran	5.5	subcutances other days	medium	low	easy	-0.230774879
1	8	1	iran	2.5	subcutances three times a weak	medium	low	hard	-0.617903819
1	8	0	iran	5.5	mascular once a weak	high	medium	easy	-0.391159447
1	9	1	iran	2.5	subcutances other days	medium	medium	hard	-0.96815646
1	9	0	other	0.5	subcutances three times a weak	high	low	hard	-0.293616445
1	10	1	iran	2.5	mascular once a weak	high	low	hard	-0.0957683
1	10	0	other	5.5	subcutances other days	medium	medium	easy	-0.647555583
1	11	1	other	5.5	subcutances three times a weak	medium	low	hard	-0.478231781
1	11	0	iran	0.5	subcutances other days	high	medium	easy	-0.562545908
1	12	0	iran	5.5	subcutances three times a weak	high	medium	hard	-0.787756632
1	12	1	other	2.5	subcutances other days	medium	low	hard	-0.352164436
2	1	0	other	5.5	subcutances other days	high	low	hard	-0.005630885
2	1	1	other	0.5	mascular once a weak	medium	medium	hard	-0.900801637
2	2	0	other	0.5	subcutances three times a weak	medium	medium	hard	-1.116469982
2	2	1	other	5.5	mascular once a weak	high	low	easy	0.224832577
2	3	1	iran	5.5	mascular once a weak	medium	medium	hard	-0.878555459
2	3	0	iran	2.5	subcutances three times a weak	high	low	hard	-0.311436646
2	4	1	other	0.5	mascular once a weak	medium	low	hard	-0.384415273
2	4	0	other	2.5	subcutances three times a weak	high	medium	easy	-0.54728851

2	5	1	iran	0.5	subcutances	other days	high	low	hard	-0.227088383
2	5	0	iran	2.5	mascular	once a weak	medium	medium	easy	-0.737692999
2	6	0	other	2.5	subcutances	other days	high	medium	hard	-0.562083627
2	6	1	iran	0.5	subcutances	three times a weak	medium	low	easy	-0.518760439
2	7	0	other	0.5	mascular	once a weak	high	medium	hard	-0.594334463
2	7	1	iran	5.5	subcutances	other days	medium	low	easy	-0.230774879
2	8	1	iran	2.5	subcutances	three times a weak	medium	low	hard	-0.617903819
2	8	0	iran	5.5	mascular	once a weak	high	medium	easy	-0.391159447
2	9	1	iran	2.5	subcutances	other days	medium	medium	hard	-0.96815646
2	9	0	other	0.5	subcutances	three times a weak	high	low	hard	-0.293616445
2	10	1	iran	2.5	mascular	once a weak	high	low	hard	-0.0957683
2	10	0	other	5.5	subcutances	other days	medium	medium	easy	-0.647555583
2	11	1	other	5.5	subcutances	three times a weak	medium	low	hard	-0.478231781
2	11	0	iran	0.5	subcutances	other days	high	medium	easy	-0.562545908
2	12	0	iran	5.5	subcutances	three times a weak	high	medium	hard	-0.787756632
2	12	1	other	2.5	subcutances	other days	medium	low	hard	-0.352164436
3	1	0	other	5.5	subcutances	other days	high	low	hard	-0.005630885
3	1	1	other	0.5	mascular	once a weak	medium	medium	hard	-0.900801637
3	2	0	other	0.5	subcutances	three times a weak	medium	medium	hard	-1.116469982
3	2	1	other	5.5	mascular	once a weak	high	low	easy	0.224832577
3	3	1	iran	5.5	mascular	once a weak	medium	medium	hard	-0.878555459
3	3	0	iran	2.5	subcutances	three times a weak	high	low	hard	-0.311436646
3	4	1	other	0.5	mascular	once a weak	medium	low	hard	-0.384415273
3	4	0	other	2.5	subcutances	three times a weak	high	medium	easy	-0.54728851
3	5	1	iran	0.5	subcutances	other days	high	low	hard	-0.227088383
3	5	0	iran	2.5	mascular	once a weak	medium	medium	easy	-0.737692999
3	6	1	other	2.5	subcutances	other days	high	medium	hard	-0.562083627
3	6	0	iran	0.5	subcutances	three times a weak	medium	low	easy	-0.518760439
3	7	0	other	0.5	mascular	once a weak	high	medium	hard	-0.594334463
3	7	1	iran	5.5	subcutances	other days	medium	low	easy	-0.230774879
3	8	1	iran	2.5	subcutances	three times a weak	medium	low	hard	-0.617903819
3	8	0	iran	5.5	mascular	once a weak	high	medium	easy	-0.391159447
3	9	0	iran	2.5	subcutances	other days	medium	medium	hard	-0.96815646
3	9	1	other	0.5	subcutances	three times a weak	high	low	hard	-0.293616445

3	10	1	iran	2.5	mascular once a weak	high	low	hard	-0.0957683
3	10	0	other	5.5	subcutances other days	medium	medium	easy	-0.647555583
3	11	1	other	5.5	subcutances three times a weak	medium	low	hard	-0.478231781
3	11	0	iran	0.5	subcutances other days	high	medium	easy	-0.562545908
3	12	1	iran	5.5	subcutances three times a weak	high	medium	hard	-0.787756632
3	12	0	other	2.5	subcutances other days	medium	low	hard	-0.352164436
4	1	0	other	5.5	subcutances other days	high	low	hard	-0.005630885
4	1	1	other	0.5	mascular once a weak	medium	medium	hard	-0.900801637
4	2	1	other	0.5	subcutances three times a weak	medium	medium	hard	-1.116469982
4	2	0	other	5.5	mascular once a weak	high	low	easy	0.224832577
4	3	0	iran	5.5	mascular once a weak	medium	medium	hard	-0.878555459
4	3	1	iran	2.5	subcutances three times a weak	high	low	hard	-0.311436646
4	4	1	other	0.5	mascular once a weak	medium	low	hard	-0.384415273
4	4	0	other	2.5	subcutances three times a weak	high	medium	easy	-0.54728851
4	5	1	iran	0.5	subcutances other days	high	low	hard	-0.227088383
4	5	0	iran	2.5	mascular once a weak	medium	medium	easy	-0.737692999
4	6	1	other	2.5	subcutances other days	high	medium	hard	-0.562083627
4	6	0	iran	0.5	subcutances three times a weak	medium	low	easy	-0.518760439
4	7	0	other	0.5	mascular once a weak	high	medium	hard	-0.594334463
4	7	1	iran	5.5	subcutances other days	medium	low	easy	-0.230774879
4	8	0	iran	2.5	subcutances three times a weak	medium	low	hard	-0.617903819
4	8	1	iran	5.5	mascular once a weak	high	medium	easy	-0.391159447
4	9	1	iran	2.5	subcutances other days	medium	medium	hard	-0.96815646
4	9	0	other	0.5	subcutances three times a weak	high	low	hard	-0.293616445
4	10	0	iran	2.5	mascular once a weak	high	low	hard	-0.0957683
4	10	1	other	5.5	subcutances other days	medium	medium	easy	-0.647555583
4	11	1	other	5.5	subcutances three times a weak	medium	low	hard	-0.478231781
4	11	0	iran	0.5	subcutances other days	high	medium	easy	-0.562545908
4	12	0	iran	5.5	subcutances three times a weak	high	medium	hard	-0.787756632
4	12	1	other	2.5	subcutances other days	medium	low	hard	-0.352164436
5	1	1	other	5.5	subcutances other days	high	low	hard	-0.005630885
5	1	0	other	0.5	mascular once a weak	medium	medium	hard	-0.900801637
5	2	0	other	0.5	subcutances three times a weak	medium	medium	hard	-1.116469982
5	2	1	other	5.5	mascular once a weak	high	low	easy	0.224832577

5	3	1	iran	5.5	mascular once a weak	medium	medium	hard	-0.878555459
5	3	0	iran	2.5	subcutances three times a weak	high	low	hard	-0.311436646
5	4	1	other	0.5	mascular once a weak	medium	low	hard	-0.384415273
5	4	0	other	2.5	subcutances three times a weak	high	medium	easy	-0.54728851
5	5	1	iran	0.5	subcutances other days	high	low	hard	-0.227088383
5	5	0	iran	2.5	mascular once a weak	medium	medium	easy	-0.737692999
5	6	0	other	2.5	subcutances other days	high	medium	hard	-0.562083627
5	6	1	iran	0.5	subcutances three times a weak	medium	low	easy	-0.518760439
5	7	1	other	0.5	mascular once a weak	high	medium	hard	-0.594334463
5	7	0	iran	5.5	subcutances other days	medium	low	easy	-0.230774879
5	8	1	iran	2.5	subcutances three times a weak	medium	low	hard	-0.617903819
5	8	0	iran	5.5	mascular once a weak	high	medium	easy	-0.391159447
5	9	0	iran	2.5	subcutances other days	medium	medium	hard	-0.96815646
5	9	1	other	0.5	subcutances three times a weak	high	low	hard	-0.293616445
5	10	1	iran	2.5	mascular once a weak	high	low	hard	-0.0957683
5	10	0	other	5.5	subcutances other days	medium	medium	easy	-0.647555583
5	11	0	other	5.5	subcutances three times a weak	medium	low	hard	-0.478231781
5	11	1	iran	0.5	subcutances other days	high	medium	easy	-0.562545908
5	12	1	iran	5.5	subcutances three times a weak	high	medium	hard	-0.787756632
5	12	0	other	2.5	subcutances other days	medium	low	hard	-0.352164436
6	1	0	other	5.5	subcutances other days	high	low	hard	-0.005630885
6	1	1	other	0.5	mascular once a weak	medium	medium	hard	-0.900801637
6	2	0	other	0.5	subcutances three times a weak	medium	medium	hard	-1.116469982
6	2	1	other	5.5	mascular once a weak	high	low	easy	0.224832577
6	3	0	iran	5.5	mascular once a weak	medium	medium	hard	-0.878555459
6	3	1	iran	2.5	subcutances three times a weak	high	low	hard	-0.311436646
6	4	1	other	0.5	mascular once a weak	medium	low	hard	-0.384415273
6	4	0	other	2.5	subcutances three times a weak	high	medium	easy	-0.54728851
6	5	1	iran	0.5	subcutances other days	high	low	hard	-0.227088383
6	5	0	iran	2.5	mascular once a weak	medium	medium	easy	-0.737692999
6	6	0	other	2.5	subcutances other days	high	medium	hard	-0.562083627
6	6	1	iran	0.5	subcutances three times a weak	medium	low	easy	-0.518760439
6	7	1	other	0.5	mascular once a weak	high	medium	hard	-0.594334463
6	7	0	iran	5.5	subcutances other days	medium	low	easy	-0.230774879

6	8	0	iran	2.5	subcutances three times a weak	medium	low	hard	-0.617903819
6	8	1	iran	5.5	mascular once a weak	high	medium	easy	-0.391159447
6	9	0	iran	2.5	subcutances other days	medium	medium	hard	-0.96815646
6	9	1	other	0.5	subcutances three times a weak	high	low	hard	-0.293616445
6	10	1	iran	2.5	mascular once a weak	high	low	hard	-0.0957683
6	10	0	other	5.5	subcutances other days	medium	medium	easy	-0.647555583
6	11	1	other	5.5	subcutances three times a weak	medium	low	hard	-0.478231781
6	11	0	iran	0.5	subcutances other days	high	medium	easy	-0.562545908
6	12	0	iran	5.5	subcutances three times a weak	high	medium	hard	-0.787756632
6	12	1	other	2.5	subcutances other days	medium	low	hard	-0.352164436
7	1	0	iran	5.5	mascular once a weak	high	medium	hard	-0.572088286
7	1	1	iran	0.5	subcutances three times a weak	high	medium	easy	-0.72867963
7	2	0	other	0.5	subcutances other days	high	medium	hard	-0.643869086
7	2	1	iran	5.5	subcutances other days	medium	medium	hard	-0.928090082
7	3	1	iran	5.5	mascular once a weak	high	medium	hard	-0.572088286
7	3	0	iran	2.5	subcutances three times a weak	high	medium	easy	-0.646894171
7	4	0	iran	0.5	subcutances three times a weak	medium	low	hard	-0.699689278
7	4	1	other	2.5	mascular once a weak	high	low	hard	0.00383736
7	5	1	iran	0.5	mascular once a weak	medium	low	easy	-0.303092094
7	5	0	other	2.5	subcutances three times a weak	high	low	easy	-0.030902146
7	6	0	other	2.5	subcutances three times a weak	high	low	easy	-0.030902146
7	6	1	other	0.5	subcutances other days	high	medium	easy	-0.462940247
7	7	0	other	0.5	mascular once a weak	medium	medium	hard	-0.900801637
7	7	1	other	5.5	subcutances other days	medium	low	hard	-0.312098058
7	8	0	iran	2.5	mascular once a weak	high	low	hard	-0.0957683
7	8	1	iran	5.5	subcutances three times a weak	high	low	easy	-0.090441429
7	9	1	iran	2.5	subcutances three times a weak	medium	low	hard	-0.617903819
7	9	0	other	0.5	subcutances other days	high	medium	hard	-0.643869086
7	10	1	other	2.5	mascular once a weak	high	low	hard	0.00383736
7	10	0	iran	5.5	mascular once a weak	medium	medium	easy	-0.697626621
7	11	0	other	5.5	subcutances other days	high	medium	easy	-0.34108841
7	11	1	iran	0.5	subcutances three times a weak	medium	low	hard	-0.699689278
7	12	0	iran	5.5	subcutances three times a weak	high	medium	hard	-0.787756632
7	12	1	other	2.5	subcutances other days	medium	low	hard	-0.352164436

8	1	1	iran	5.5	mascular once a weak	high	medium	hard	-0.572088286
8	1	0	iran	0.5	subcutances three times a weak	high	medium	easy	-0.72867963
8	2	1	other	0.5	subcutances other days	high	medium	hard	-0.643869086
8	2	0	iran	5.5	subcutances other days	medium	medium	hard	-0.928090082
8	3	1	iran	5.5	mascular once a weak	high	medium	hard	-0.572088286
8	3	0	iran	2.5	subcutances three times a weak	high	medium	easy	-0.646894171
8	4	0	iran	0.5	subcutances three times a weak	medium	low	hard	-0.699689278
8	4	1	other	2.5	mascular once a weak	high	low	hard	0.00383736
8	5	0	iran	0.5	mascular once a weak	medium	low	easy	-0.303092094
8	5	1	other	2.5	subcutances three times a weak	high	low	easy	-0.030902146
8	6	1	other	2.5	subcutances three times a weak	high	low	easy	-0.030902146
8	6	0	other	0.5	subcutances other days	high	medium	easy	-0.462940247
8	7	0	other	0.5	mascular once a weak	medium	medium	hard	-0.900801637
8	7	1	other	5.5	subcutances other days	medium	low	hard	-0.312098058
8	8	1	iran	2.5	mascular once a weak	high	low	hard	-0.0957683
8	8	0	iran	5.5	subcutances three times a weak	high	low	easy	-0.090441429
8	9	1	iran	2.5	subcutances three times a weak	medium	low	hard	-0.617903819
8	9	0	other	0.5	subcutances other days	high	medium	hard	-0.643869086
8	10	1	other	2.5	mascular once a weak	high	low	hard	0.00383736
8	10	0	iran	5.5	mascular once a weak	medium	medium	easy	-0.697626621
8	11	0	other	5.5	subcutances other days	high	medium	easy	-0.34108841
8	11	1	iran	0.5	subcutances three times a weak	medium	low	hard	-0.699689278
8	12	0	iran	5.5	subcutances three times a weak	high	medium	hard	-0.787756632
8	12	1	other	2.5	subcutances other days	medium	low	hard	-0.352164436
9	1	1	iran	5.5	mascular once a weak	high	medium	hard	-0.572088286
9	1	0	iran	0.5	subcutances three times a weak	high	medium	easy	-0.72867963
9	2	1	other	0.5	subcutances other days	high	medium	hard	-0.643869086
9	2	0	iran	5.5	subcutances other days	medium	medium	hard	-0.928090082
9	3	1	iran	5.5	mascular once a weak	high	medium	hard	-0.572088286
9	3	0	iran	2.5	subcutances three times a weak	high	medium	easy	-0.646894171
9	4	0	iran	0.5	subcutances three times a weak	medium	low	hard	-0.699689278
9	4	1	other	2.5	mascular once a weak	high	low	hard	0.00383736
9	5	0	iran	0.5	mascular once a weak	medium	low	easy	-0.303092094
9	5	1	other	2.5	subcutances three times a weak	high	low	easy	-0.030902146

9	6	0	other	2.5	subcutances three times a weak	high	low	easy	-0.030902146
9	6	1	other	0.5	subcutances other days	high	medium	easy	-0.462940247
9	7	0	other	0.5	mascular once a weak	medium	medium	hard	-0.900801637
9	7	1	other	5.5	subcutances other days	medium	low	hard	-0.312098058
9	8	0	iran	2.5	mascular once a weak	high	low	hard	-0.0957683
9	8	1	iran	5.5	subcutances three times a weak	high	low	easy	-0.090441429
9	9	0	iran	2.5	subcutances three times a weak	medium	low	hard	-0.617903819
9	9	1	other	0.5	subcutances other days	high	medium	hard	-0.643869086
9	10	1	other	2.5	mascular once a weak	high	low	hard	0.00383736
9	10	0	iran	5.5	mascular once a weak	medium	medium	easy	-0.697626621
9	11	0	other	5.5	subcutances other days	high	medium	easy	-0.34108841
9	11	1	iran	0.5	subcutances three times a weak	medium	low	hard	-0.699689278
9	12	0	iran	5.5	subcutances three times a weak	high	medium	hard	-0.787756632
9	12	1	other	2.5	subcutances other days	medium	low	hard	-0.352164436
10	1	1	iran	5.5	mascular once a weak	high	medium	hard	-0.572088286
10	1	0	iran	0.5	subcutances three times a weak	high	medium	easy	-0.72867963
10	2	1	other	0.5	subcutances other days	high	medium	hard	-0.643869086
10	2	0	iran	5.5	subcutances other days	medium	medium	hard	-0.928090082
10	3	1	iran	5.5	mascular once a weak	high	medium	hard	-0.572088286
10	3	0	iran	2.5	subcutances three times a weak	high	medium	easy	-0.646894171
10	4	0	iran	0.5	subcutances three times a weak	medium	low	hard	-0.699689278
10	4	1	other	2.5	mascular once a weak	high	low	hard	0.00383736
10	5	1	iran	0.5	mascular once a weak	medium	low	easy	-0.303092094
10	5	0	other	2.5	subcutances three times a weak	high	low	easy	-0.030902146
10	6	1	other	2.5	subcutances three times a weak	high	low	easy	-0.030902146
10	6	0	other	0.5	subcutances other days	high	medium	easy	-0.462940247
10	7	1	other	0.5	mascular once a weak	medium	medium	hard	-0.900801637
10	7	0	other	5.5	subcutances other days	medium	low	hard	-0.312098058
10	8	1	iran	2.5	mascular once a weak	high	low	hard	-0.0957683
10	8	0	iran	5.5	subcutances three times a weak	high	low	easy	-0.090441429
10	9	1	iran	2.5	subcutances three times a weak	medium	low	hard	-0.617903819
10	9	0	other	0.5	subcutances other days	high	medium	hard	-0.643869086
10	10	1	other	2.5	mascular once a weak	high	low	hard	0.00383736
10	10	0	iran	5.5	mascular once a weak	medium	medium	easy	-0.697626621

10	11	0	other	5.5	subcutances other days	high	medium	easy	-0.34108841
10	11	1	iran	0.5	subcutances three times a weak	medium	low	hard	-0.699689278
10	12	0	iran	5.5	subcutances three times a weak	high	medium	hard	-0.787756632
10	12	1	other	2.5	subcutances other days	medium	low	hard	-0.352164436
11	1	1	iran	5.5	mascular once a weak	high	medium	hard	-0.572088286
11	1	0	iran	0.5	subcutances three times a weak	high	medium	easy	-0.72867963
11	2	1	other	0.5	subcutances other days	high	medium	hard	-0.643869086
11	2	0	iran	5.5	subcutances other days	medium	medium	hard	-0.928090082
11	3	0	iran	5.5	mascular once a weak	high	medium	hard	-0.572088286
11	3	1	iran	2.5	subcutances three times a weak	high	medium	easy	-0.646894171
11	4	0	iran	0.5	subcutances three times a weak	medium	low	hard	-0.699689278
11	4	1	other	2.5	mascular once a weak	high	low	hard	0.00383736
11	5	0	iran	0.5	mascular once a weak	medium	low	easy	-0.303092094
11	5	1	other	2.5	subcutances three times a weak	high	low	easy	-0.030902146
11	6	0	other	2.5	subcutances three times a weak	high	low	easy	-0.030902146
11	6	1	other	0.5	subcutances other days	high	medium	easy	-0.462940247
11	7	0	other	0.5	mascular once a weak	medium	medium	hard	-0.900801637
11	7	1	other	5.5	subcutances other days	medium	low	hard	-0.312098058
11	8	1	iran	2.5	mascular once a weak	high	low	hard	-0.0957683
11	8	0	iran	5.5	subcutances three times a weak	high	low	easy	-0.090441429
11	9	0	iran	2.5	subcutances three times a weak	medium	low	hard	-0.617903819
11	9	1	other	0.5	subcutances other days	high	medium	hard	-0.643869086
11	10	1	other	2.5	mascular once a weak	high	low	hard	0.00383736
11	10	0	iran	5.5	mascular once a weak	medium	medium	easy	-0.697626621
11	11	1	other	5.5	subcutances other days	high	medium	easy	-0.34108841
11	11	0	iran	0.5	subcutances three times a weak	medium	low	hard	-0.699689278
11	12	1	iran	5.5	subcutances three times a weak	high	medium	hard	-0.787756632
11	12	0	other	2.5	subcutances other days	medium	low	hard	-0.352164436
12	1	1	iran	5.5	mascular once a weak	high	medium	hard	-0.572088286
12	1	0	iran	0.5	subcutances three times a weak	high	medium	easy	-0.72867963
12	2	0	other	0.5	subcutances other days	high	medium	hard	-0.643869086
12	2	1	iran	5.5	subcutances other days	medium	medium	hard	-0.928090082
12	3	0	iran	5.5	mascular once a weak	high	medium	hard	-0.572088286
12	3	1	iran	2.5	subcutances three times a weak	high	medium	easy	-0.646894171

12	4	1	iran	0.5	subcutances three times a weak	medium	low	hard	-0.699689278
12	4	0	other	2.5	mascular once a weak	high	low	hard	0.00383736
12	5	1	iran	0.5	mascular once a weak	medium	low	easy	-0.303092094
12	5	0	other	2.5	subcutances three times a weak	high	low	easy	-0.030902146
12	6	0	other	2.5	subcutances three times a weak	high	low	easy	-0.030902146
12	6	1	other	0.5	subcutances other days	high	medium	easy	-0.462940247
12	7	1	other	0.5	mascular once a weak	medium	medium	hard	-0.900801637
12	7	0	other	5.5	subcutances other days	medium	low	hard	-0.312098058
12	8	1	iran	2.5	mascular once a weak	high	low	hard	-0.0957683
12	8	0	iran	5.5	subcutances three times a weak	high	low	easy	-0.090441429
12	9	1	iran	2.5	subcutances three times a weak	medium	low	hard	-0.617903819
12	9	0	other	0.5	subcutances other days	high	medium	hard	-0.643869086
12	10	0	other	2.5	mascular once a weak	high	low	hard	0.00383736
12	10	1	iran	5.5	mascular once a weak	medium	medium	easy	-0.697626621
12	11	0	other	5.5	subcutances other days	high	medium	easy	-0.34108841
12	11	1	iran	0.5	subcutances three times a weak	medium	low	hard	-0.699689278
12	12	0	iran	5.5	subcutances three times a weak	high	medium	hard	-0.787756632
12	12	1	other	2.5	subcutances other days	medium	low	hard	-0.352164436
13	1	1	iran	5.5	mascular once a weak	high	medium	hard	-0.572088286
13	1	0	iran	0.5	subcutances three times a weak	high	medium	easy	-0.72867963
13	2	1	other	0.5	subcutances other days	high	medium	hard	-0.643869086
13	2	0	iran	5.5	subcutances other days	medium	medium	hard	-0.928090082
13	3	1	iran	5.5	mascular once a weak	high	medium	hard	-0.572088286
13	3	0	iran	2.5	subcutances three times a weak	high	medium	easy	-0.646894171
13	4	0	iran	0.5	subcutances three times a weak	medium	low	hard	-0.699689278
13	4	1	other	2.5	mascular once a weak	high	low	hard	0.00383736
13	5	1	iran	0.5	mascular once a weak	medium	low	easy	-0.303092094
13	5	0	other	2.5	subcutances three times a weak	high	low	easy	-0.030902146
13	6	1	other	2.5	subcutances three times a weak	high	low	easy	-0.030902146
13	6	0	other	0.5	subcutances other days	high	medium	easy	-0.462940247
13	7	1	other	0.5	mascular once a weak	medium	medium	hard	-0.900801637
13	7	0	other	5.5	subcutances other days	medium	low	hard	-0.312098058
13	8	1	iran	2.5	mascular once a weak	high	low	hard	-0.0957683
13	8	0	iran	5.5	subcutances three times a weak	high	low	easy	-0.090441429

13	9	1	iran	2.5	subcutances three times a weak	medium	low	hard	-0.617903819
13	9	0	other	0.5	subcutances other days	high	medium	hard	-0.643869086
13	10	0	other	2.5	mascular once a weak	high	low	hard	0.00383736
13	10	1	iran	5.5	mascular once a weak	medium	medium	easy	-0.697626621
13	11	1	other	5.5	subcutances other days	high	medium	easy	-0.34108841
13	11	0	iran	0.5	subcutances three times a weak	medium	low	hard	-0.699689278
13	12	0	iran	5.5	subcutances three times a weak	high	medium	hard	-0.787756632
13	12	1	other	2.5	subcutances other days	medium	low	hard	-0.352164436
14	1	1	iran	5.5	mascular once a weak	high	medium	hard	-0.572088286
14	1	0	iran	0.5	subcutances three times a weak	high	medium	easy	-0.72867963
14	2	1	other	0.5	subcutances other days	high	medium	hard	-0.643869086
14	2	0	iran	5.5	subcutances other days	medium	medium	hard	-0.928090082
14	3	0	iran	5.5	mascular once a weak	high	medium	hard	-0.572088286
14	3	1	iran	2.5	subcutances three times a weak	high	medium	easy	-0.646894171
14	4	0	iran	0.5	subcutances three times a weak	medium	low	hard	-0.699689278
14	4	1	other	2.5	mascular once a weak	high	low	hard	0.00383736
14	5	0	iran	0.5	mascular once a weak	medium	low	easy	-0.303092094
14	5	1	other	2.5	subcutances three times a weak	high	low	easy	-0.030902146
14	6	1	other	2.5	subcutances three times a weak	high	low	easy	-0.030902146
14	6	0	other	0.5	subcutances other days	high	medium	easy	-0.462940247
14	7	0	other	0.5	mascular once a weak	medium	medium	hard	-0.900801637
14	7	1	other	5.5	subcutances other days	medium	low	hard	-0.312098058
14	8	1	iran	2.5	mascular once a weak	high	low	hard	-0.0957683
14	8	0	iran	5.5	subcutances three times a weak	high	low	easy	-0.090441429
14	9	0	iran	2.5	subcutances three times a weak	medium	low	hard	-0.617903819
14	9	1	other	0.5	subcutances other days	high	medium	hard	-0.643869086
14	10	1	other	2.5	mascular once a weak	high	low	hard	0.00383736
14	10	0	iran	5.5	mascular once a weak	medium	medium	easy	-0.697626621
14	11	0	other	5.5	subcutances other days	high	medium	easy	-0.34108841
14	11	1	iran	0.5	subcutances three times a weak	medium	low	hard	-0.699689278
14	12	1	iran	5.5	subcutances three times a weak	high	medium	hard	-0.787756632
14	12	0	other	2.5	subcutances other days	medium	low	hard	-0.352164436
15	1	1	iran	5.5	mascular once a weak	high	medium	hard	-0.572088286
15	1	0	iran	0.5	subcutances three times a weak	high	medium	easy	-0.72867963

15	2	1	other	0.5	subcutances other days	high	medium	hard	-0.643869086
15	2	0	iran	5.5	subcutances other days	medium	medium	hard	-0.928090082
15	3	0	iran	5.5	mascular once a weak	high	medium	hard	-0.572088286
15	3	1	iran	2.5	subcutances three times a weak	high	medium	easy	-0.646894171
15	4	0	iran	0.5	subcutances three times a weak	medium	low	hard	-0.699689278
15	4	1	other	2.5	mascular once a weak	high	low	hard	0.00383736
15	5	0	iran	0.5	mascular once a weak	medium	low	easy	-0.303092094
15	5	1	other	2.5	subcutances three times a weak	high	low	easy	-0.030902146
15	6	1	other	2.5	subcutances three times a weak	high	low	easy	-0.030902146
15	6	0	other	0.5	subcutances other days	high	medium	easy	-0.462940247
15	7	0	other	0.5	mascular once a weak	medium	medium	hard	-0.900801637
15	7	1	other	5.5	subcutances other days	medium	low	hard	-0.312098058
15	8	1	iran	2.5	mascular once a weak	high	low	hard	-0.0957683
15	8	0	iran	5.5	subcutances three times a weak	high	low	easy	-0.090441429
15	9	1	iran	2.5	subcutances three times a weak	medium	low	hard	-0.617903819
15	9	0	other	0.5	subcutances other days	high	medium	hard	-0.643869086
15	10	0	other	2.5	mascular once a weak	high	low	hard	0.00383736
15	10	1	iran	5.5	mascular once a weak	medium	medium	easy	-0.697626621
15	11	1	other	5.5	subcutances other days	high	medium	easy	-0.34108841
15	11	0	iran	0.5	subcutances three times a weak	medium	low	hard	-0.699689278
15	12	0	iran	5.5	subcutances three times a weak	high	medium	hard	-0.787756632
15	12	1	other	2.5	subcutances other days	medium	low	hard	-0.352164436
16	1	0	iran	5.5	mascular once a weak	high	medium	hard	-0.572088286
16	1	1	iran	0.5	subcutances three times a weak	high	medium	easy	-0.72867963
16	2	1	other	0.5	subcutances other days	high	medium	hard	-0.643869086
16	2	0	iran	5.5	subcutances other days	medium	medium	hard	-0.928090082
16	3	0	iran	5.5	mascular once a weak	high	medium	hard	-0.572088286
16	3	1	iran	2.5	subcutances three times a weak	high	medium	easy	-0.646894171
16	4	1	iran	0.5	subcutances three times a weak	medium	low	hard	-0.699689278
16	4	0	other	2.5	mascular once a weak	high	low	hard	0.00383736
16	5	0	iran	0.5	mascular once a weak	medium	low	easy	-0.303092094
16	5	1	other	2.5	subcutances three times a weak	high	low	easy	-0.030902146
16	6	1	other	2.5	subcutances three times a weak	high	low	easy	-0.030902146
16	6	0	other	0.5	subcutances other days	high	medium	easy	-0.462940247

16	7	0	other	0.5	mascular once a weak	medium	medium	hard	-0.900801637
16	7	1	other	5.5	subcutances other days	medium	low	hard	-0.312098058
16	8	0	iran	2.5	mascular once a weak	high	low	hard	-0.0957683
16	8	1	iran	5.5	subcutances three times a weak	high	low	easy	-0.090441429
16	9	0	iran	2.5	subcutances three times a weak	medium	low	hard	-0.617903819
16	9	1	other	0.5	subcutances other days	high	medium	hard	-0.643869086
16	10	1	other	2.5	mascular once a weak	high	low	hard	0.00383736
16	10	0	iran	5.5	mascular once a weak	medium	medium	easy	-0.697626621
16	11	1	other	5.5	subcutances other days	high	medium	easy	-0.34108841
16	11	0	iran	0.5	subcutances three times a weak	medium	low	hard	-0.699689278
16	12	1	iran	5.5	subcutances three times a weak	high	medium	hard	-0.787756632
16	12	0	other	2.5	subcutances other days	medium	low	hard	-0.352164436
17	1	1	iran	5.5	mascular once a weak	high	medium	hard	-0.572088286
17	1	0	iran	0.5	subcutances three times a weak	high	medium	easy	-0.72867963
17	2	1	other	0.5	subcutances other days	high	medium	hard	-0.643869086
17	2	0	iran	5.5	subcutances other days	medium	medium	hard	-0.928090082
17	3	1	iran	5.5	mascular once a weak	high	medium	hard	-0.572088286
17	3	0	iran	2.5	subcutances three times a weak	high	medium	easy	-0.646894171
17	4	0	iran	0.5	subcutances three times a weak	medium	low	hard	-0.699689278
17	4	1	other	2.5	mascular once a weak	high	low	hard	0.00383736
17	5	1	iran	0.5	mascular once a weak	medium	low	easy	-0.303092094
17	5	0	other	2.5	subcutances three times a weak	high	low	easy	-0.030902146
17	6	1	other	2.5	subcutances three times a weak	high	low	easy	-0.030902146
17	6	0	other	0.5	subcutances other days	high	medium	easy	-0.462940247
17	7	1	other	0.5	mascular once a weak	medium	medium	hard	-0.900801637
17	7	0	other	5.5	subcutances other days	medium	low	hard	-0.312098058
17	8	1	iran	2.5	mascular once a weak	high	low	hard	-0.0957683
17	8	0	iran	5.5	subcutances three times a weak	high	low	easy	-0.090441429
17	9	1	iran	2.5	subcutances three times a weak	medium	low	hard	-0.617903819
17	9	0	other	0.5	subcutances other days	high	medium	hard	-0.643869086
17	10	1	other	2.5	mascular once a weak	high	low	hard	0.00383736
17	10	0	iran	5.5	mascular once a weak	medium	medium	easy	-0.697626621
17	11	0	other	5.5	subcutances other days	high	medium	easy	-0.34108841
17	11	1	iran	0.5	subcutances three times a weak	medium	low	hard	-0.699689278

17	12	0	iran	5.5	subcutances three times a weak	high	medium	hard	-0.787756632
17	12	1	other	2.5	subcutances other days	medium	low	hard	-0.352164436
18	1	1	iran	5.5	mascular once a weak	high	medium	hard	-0.572088286
18	1	0	iran	0.5	subcutances three times a weak	high	medium	easy	-0.72867963
18	2	1	other	0.5	subcutances other days	high	medium	hard	-0.643869086
18	2	0	iran	5.5	subcutances other days	medium	medium	hard	-0.928090082
18	3	0	iran	5.5	mascular once a weak	high	medium	hard	-0.572088286
18	3	1	iran	2.5	subcutances three times a weak	high	medium	easy	-0.646894171
18	4	0	iran	0.5	subcutances three times a weak	medium	low	hard	-0.699689278
18	4	1	other	2.5	mascular once a weak	high	low	hard	0.00383736
18	5	0	iran	0.5	mascular once a weak	medium	low	easy	-0.303092094
18	5	1	other	2.5	subcutances three times a weak	high	low	easy	-0.030902146
18	6	0	other	2.5	subcutances three times a weak	high	low	easy	-0.030902146
18	6	1	other	0.5	subcutances other days	high	medium	easy	-0.462940247
18	7	0	other	0.5	mascular once a weak	medium	medium	hard	-0.900801637
18	7	1	other	5.5	subcutances other days	medium	low	hard	-0.312098058
18	8	1	iran	2.5	mascular once a weak	high	low	hard	-0.0957683
18	8	0	iran	5.5	subcutances three times a weak	high	low	easy	-0.090441429
18	9	0	iran	2.5	subcutances three times a weak	medium	low	hard	-0.617903819
18	9	1	other	0.5	subcutances other days	high	medium	hard	-0.643869086
18	10	1	other	2.5	mascular once a weak	high	low	hard	0.00383736
18	10	0	iran	5.5	mascular once a weak	medium	medium	easy	-0.697626621
18	11	1	other	5.5	subcutances other days	high	medium	easy	-0.34108841
18	11	0	iran	0.5	subcutances three times a weak	medium	low	hard	-0.699689278
18	12	0	iran	5.5	subcutances three times a weak	high	medium	hard	-0.787756632
18	12	1	other	2.5	subcutances other days	medium	low	hard	-0.352164436
19	1	1	iran	5.5	mascular once a weak	high	medium	hard	-0.572088286
19	1	0	iran	0.5	subcutances three times a weak	high	medium	easy	-0.72867963
19	2	0	other	0.5	subcutances other days	high	medium	hard	-0.643869086
19	2	1	iran	5.5	subcutances other days	medium	medium	hard	-0.928090082
19	3	0	iran	5.5	mascular once a weak	high	medium	hard	-0.572088286
19	3	1	iran	2.5	subcutances three times a weak	high	medium	easy	-0.646894171
19	4	1	iran	0.5	subcutances three times a weak	medium	low	hard	-0.699689278
19	4	0	other	2.5	mascular once a weak	high	low	hard	0.00383736

19	5	1	iran	0.5	mascular once a weak	medium	low	easy	-0.303092094
19	5	0	other	2.5	subcutances three times a weak	high	low	easy	-0.030902146
19	6	0	other	2.5	subcutances three times a weak	high	low	easy	-0.030902146
19	6	1	other	0.5	subcutances other days	high	medium	easy	-0.462940247
19	7	1	other	0.5	mascular once a weak	medium	medium	hard	-0.900801637
19	7	0	other	5.5	subcutances other days	medium	low	hard	-0.312098058
19	8	1	iran	2.5	mascular once a weak	high	low	hard	-0.0957683
19	8	0	iran	5.5	subcutances three times a weak	high	low	easy	-0.090441429
19	9	1	iran	2.5	subcutances three times a weak	medium	low	hard	-0.617903819
19	9	0	other	0.5	subcutances other days	high	medium	hard	-0.643869086
19	10	0	other	2.5	mascular once a weak	high	low	hard	0.00383736
19	10	1	iran	5.5	mascular once a weak	medium	medium	easy	-0.697626621
19	11	0	other	5.5	subcutances other days	high	medium	easy	-0.34108841
19	11	1	iran	0.5	subcutances three times a weak	medium	low	hard	-0.699689278
19	12	0	iran	5.5	subcutances three times a weak	high	medium	hard	-0.787756632
19	12	1	other	2.5	subcutances other days	medium	low	hard	-0.352164436
20	1	1	iran	5.5	mascular once a weak	high	medium	hard	-0.572088286
20	1	0	iran	0.5	subcutances three times a weak	high	medium	easy	-0.72867963
20	2	1	other	0.5	subcutances other days	high	medium	hard	-0.643869086
20	2	0	iran	5.5	subcutances other days	medium	medium	hard	-0.928090082
20	3	1	iran	5.5	mascular once a weak	high	medium	hard	-0.572088286
20	3	0	iran	2.5	subcutances three times a weak	high	medium	easy	-0.646894171
20	4	0	iran	0.5	subcutances three times a weak	medium	low	hard	-0.699689278
20	4	1	other	2.5	mascular once a weak	high	low	hard	0.00383736
20	5	0	iran	0.5	mascular once a weak	medium	low	easy	-0.303092094
20	5	1	other	2.5	subcutances three times a weak	high	low	easy	-0.030902146
20	6	1	other	2.5	subcutances three times a weak	high	low	easy	-0.030902146
20	6	0	other	0.5	subcutances other days	high	medium	easy	-0.462940247
20	7	1	other	0.5	mascular once a weak	medium	medium	hard	-0.900801637
20	7	0	other	5.5	subcutances other days	medium	low	hard	-0.312098058
20	8	1	iran	2.5	mascular once a weak	high	low	hard	-0.0957683
20	8	0	iran	5.5	subcutances three times a weak	high	low	easy	-0.090441429
20	9	0	iran	2.5	subcutances three times a weak	medium	low	hard	-0.617903819
20	9	1	other	0.5	subcutances other days	high	medium	hard	-0.643869086

20	10	1	other	2.5	mascular once a weak	high	low	hard	0.00383736
20	10	0	iran	5.5	mascular once a weak	medium	medium	easy	-0.697626621
20	11	1	other	5.5	subcutances other days	high	medium	easy	-0.34108841
20	11	0	iran	0.5	subcutances three times a weak	medium	low	hard	-0.699689278
20	12	1	iran	5.5	subcutances three times a weak	high	medium	hard	-0.787756632
20	12	0	other	2.5	subcutances other days	medium	low	hard	-0.352164436
21	1	1	iran	5.5	mascular once a weak	high	medium	hard	-0.572088286
21	1	0	iran	0.5	subcutances three times a weak	high	medium	easy	-0.72867963
21	2	1	other	0.5	subcutances other days	high	medium	hard	-0.643869086
21	2	0	iran	5.5	subcutances other days	medium	medium	hard	-0.928090082
21	3	0	iran	5.5	mascular once a weak	high	medium	hard	-0.572088286
21	3	1	iran	2.5	subcutances three times a weak	high	medium	easy	-0.646894171
21	4	0	iran	0.5	subcutances three times a weak	medium	low	hard	-0.699689278
21	4	1	other	2.5	mascular once a weak	high	low	hard	0.00383736
21	5	0	iran	0.5	mascular once a weak	medium	low	easy	-0.303092094
21	5	1	other	2.5	subcutances three times a weak	high	low	easy	-0.030902146
21	6	0	other	2.5	subcutances three times a weak	high	low	easy	-0.030902146
21	6	1	other	0.5	subcutances other days	high	medium	easy	-0.462940247
21	7	0	other	0.5	mascular once a weak	medium	medium	hard	-0.900801637
21	7	1	other	5.5	subcutances other days	medium	low	hard	-0.312098058
21	8	1	iran	2.5	mascular once a weak	high	low	hard	-0.0957683
21	8	0	iran	5.5	subcutances three times a weak	high	low	easy	-0.090441429
21	9	1	iran	2.5	subcutances three times a weak	medium	low	hard	-0.617903819
21	9	0	other	0.5	subcutances other days	high	medium	hard	-0.643869086
21	10	1	other	2.5	mascular once a weak	high	low	hard	0.00383736
21	10	0	iran	5.5	mascular once a weak	medium	medium	easy	-0.697626621
21	11	0	other	5.5	subcutances other days	high	medium	easy	-0.34108841
21	11	1	iran	0.5	subcutances three times a weak	medium	low	hard	-0.699689278
21	12	0	iran	5.5	subcutances three times a weak	high	medium	hard	-0.787756632
21	12	1	other	2.5	subcutances other days	medium	low	hard	-0.352164436
22	1	0	iran	5.5	mascular once a weak	high	medium	hard	-0.572088286
22	1	1	iran	0.5	subcutances three times a weak	high	medium	easy	-0.72867963
22	2	1	other	0.5	subcutances other days	high	medium	hard	-0.643869086
22	2	0	iran	5.5	subcutances other days	medium	medium	hard	-0.928090082

22	3	1	iran	5.5	mascular once a weak	high	medium	hard	-0.572088286
22	3	0	iran	2.5	subcutances three times a weak	high	medium	easy	-0.646894171
22	4	0	iran	0.5	subcutances three times a weak	medium	low	hard	-0.699689278
22	4	1	other	2.5	mascular once a weak	high	low	hard	0.00383736
22	5	0	iran	0.5	mascular once a weak	medium	low	easy	-0.303092094
22	5	1	other	2.5	subcutances three times a weak	high	low	easy	-0.030902146
22	6	1	other	2.5	subcutances three times a weak	high	low	easy	-0.030902146
22	6	0	other	0.5	subcutances other days	high	medium	easy	-0.462940247
22	7	1	other	0.5	mascular once a weak	medium	medium	hard	-0.900801637
22	7	0	other	5.5	subcutances other days	medium	low	hard	-0.312098058
22	8	1	iran	2.5	mascular once a weak	high	low	hard	-0.0957683
22	8	0	iran	5.5	subcutances three times a weak	high	low	easy	-0.090441429
22	9	0	iran	2.5	subcutances three times a weak	medium	low	hard	-0.617903819
22	9	1	other	0.5	subcutances other days	high	medium	hard	-0.643869086
22	10	1	other	2.5	mascular once a weak	high	low	hard	0.00383736
22	10	0	iran	5.5	mascular once a weak	medium	medium	easy	-0.697626621
22	11	1	other	5.5	subcutances other days	high	medium	easy	-0.34108841
22	11	0	iran	0.5	subcutances three times a weak	medium	low	hard	-0.699689278
22	12	0	iran	5.5	subcutances three times a weak	high	medium	hard	-0.787756632
22	12	1	other	2.5	subcutances other days	medium	low	hard	-0.352164436
23	1	0	iran	5.5	mascular once a weak	high	medium	hard	-0.572088286
23	1	1	iran	0.5	subcutances three times a weak	high	medium	easy	-0.72867963
23	2	1	other	0.5	subcutances other days	high	medium	hard	-0.643869086
23	2	0	iran	5.5	subcutances other days	medium	medium	hard	-0.928090082
23	3	0	iran	5.5	mascular once a weak	high	medium	hard	-0.572088286
23	3	1	iran	2.5	subcutances three times a weak	high	medium	easy	-0.646894171
23	4	0	iran	0.5	subcutances three times a weak	medium	low	hard	-0.699689278
23	4	1	other	2.5	mascular once a weak	high	low	hard	0.00383736
23	5	0	iran	0.5	mascular once a weak	medium	low	easy	-0.303092094
23	5	1	other	2.5	subcutances three times a weak	high	low	easy	-0.030902146
23	6	1	other	2.5	subcutances three times a weak	high	low	easy	-0.030902146
23	6	0	other	0.5	subcutances other days	high	medium	easy	-0.462940247
23	7	0	other	0.5	mascular once a weak	medium	medium	hard	-0.900801637
23	7	1	other	5.5	subcutances other days	medium	low	hard	-0.312098058

23	8	0	iran	2.5	mascular once a weak	high	low	hard	-0.0957683
23	8	1	iran	5.5	subcutances three times a weak	high	low	easy	-0.090441429
23	9	0	iran	2.5	subcutances three times a weak	medium	low	hard	-0.617903819
23	9	1	other	0.5	subcutances other days	high	medium	hard	-0.643869086
23	10	1	other	2.5	mascular once a weak	high	low	hard	0.00383736
23	10	0	iran	5.5	mascular once a weak	medium	medium	easy	-0.697626621
23	11	1	other	5.5	subcutances other days	high	medium	easy	-0.34108841
23	11	0	iran	0.5	subcutances three times a weak	medium	low	hard	-0.699689278
23	12	1	iran	5.5	subcutances three times a weak	high	medium	hard	-0.787756632
23	12	0	other	2.5	subcutances other days	medium	low	hard	-0.352164436
24	1	1	iran	5.5	mascular once a weak	high	medium	hard	-0.572088286
24	1	0	iran	0.5	subcutances three times a weak	high	medium	easy	-0.72867963
24	2	1	other	0.5	subcutances other days	high	medium	hard	-0.643869086
24	2	0	iran	5.5	subcutances other days	medium	medium	hard	-0.928090082
24	3	1	iran	5.5	mascular once a weak	high	medium	hard	-0.572088286
24	3	0	iran	2.5	subcutances three times a weak	high	medium	easy	-0.646894171
24	4	0	iran	0.5	subcutances three times a weak	medium	low	hard	-0.699689278
24	4	1	other	2.5	mascular once a weak	high	low	hard	0.00383736
24	5	0	iran	0.5	mascular once a weak	medium	low	easy	-0.303092094
24	5	1	other	2.5	subcutances three times a weak	high	low	easy	-0.030902146
24	6	1	other	2.5	subcutances three times a weak	high	low	easy	-0.030902146
24	6	0	other	0.5	subcutances other days	high	medium	easy	-0.462940247
24	7	0	other	0.5	mascular once a weak	medium	medium	hard	-0.900801637
24	7	1	other	5.5	subcutances other days	medium	low	hard	-0.312098058
24	8	1	iran	2.5	mascular once a weak	high	low	hard	-0.0957683
24	8	0	iran	5.5	subcutances three times a weak	high	low	easy	-0.090441429
24	9	1	iran	2.5	subcutances three times a weak	medium	low	hard	-0.617903819
24	9	0	other	0.5	subcutances other days	high	medium	hard	-0.643869086
24	10	1	other	2.5	mascular once a weak	high	low	hard	0.00383736
24	10	0	iran	5.5	mascular once a weak	medium	medium	easy	-0.697626621
24	11	1	other	5.5	subcutances other days	high	medium	easy	-0.34108841
24	11	0	iran	0.5	subcutances three times a weak	medium	low	hard	-0.699689278
24	12	0	iran	5.5	subcutances three times a weak	high	medium	hard	-0.787756632
24	12	1	other	2.5	subcutances other days	medium	low	hard	-0.352164436

25	1	1	iran	5.5	mascular once a weak	high	medium	hard	-0.572088286
25	1	0	iran	0.5	subcutances three times a weak	high	medium	easy	-0.72867963
25	2	0	other	0.5	subcutances other days	high	medium	hard	-0.643869086
25	2	1	iran	5.5	subcutances other days	medium	medium	hard	-0.928090082
25	3	0	iran	5.5	mascular once a weak	high	medium	hard	-0.572088286
25	3	1	iran	2.5	subcutances three times a weak	high	medium	easy	-0.646894171
25	4	1	iran	0.5	subcutances three times a weak	medium	low	hard	-0.699689278
25	4	0	other	2.5	mascular once a weak	high	low	hard	0.00383736
25	5	1	iran	0.5	mascular once a weak	medium	low	easy	-0.303092094
25	5	0	other	2.5	subcutances three times a weak	high	low	easy	-0.030902146
25	6	0	other	2.5	subcutances three times a weak	high	low	easy	-0.030902146
25	6	1	other	0.5	subcutances other days	high	medium	easy	-0.462940247
25	7	0	other	0.5	mascular once a weak	medium	medium	hard	-0.900801637
25	7	1	other	5.5	subcutances other days	medium	low	hard	-0.312098058
25	8	1	iran	2.5	mascular once a weak	high	low	hard	-0.0957683
25	8	0	iran	5.5	subcutances three times a weak	high	low	easy	-0.090441429
25	9	1	iran	2.5	subcutances three times a weak	medium	low	hard	-0.617903819
25	9	0	other	0.5	subcutances other days	high	medium	hard	-0.643869086
25	10	0	other	2.5	mascular once a weak	high	low	hard	0.00383736
25	10	1	iran	5.5	mascular once a weak	medium	medium	easy	-0.697626621
25	11	0	other	5.5	subcutances other days	high	medium	easy	-0.34108841
25	11	1	iran	0.5	subcutances three times a weak	medium	low	hard	-0.699689278
25	12	0	iran	5.5	subcutances three times a weak	high	medium	hard	-0.787756632
25	12	1	other	2.5	subcutances other days	medium	low	hard	-0.352164436
26	1	1	iran	5.5	mascular once a weak	high	medium	hard	-0.572088286
26	1	0	iran	0.5	subcutances three times a weak	high	medium	easy	-0.72867963
26	2	1	other	0.5	subcutances other days	high	medium	hard	-0.643869086
26	2	0	iran	5.5	subcutances other days	medium	medium	hard	-0.928090082
26	3	1	iran	5.5	mascular once a weak	high	medium	hard	-0.572088286
26	3	0	iran	2.5	subcutances three times a weak	high	medium	easy	-0.646894171
26	4	0	iran	0.5	subcutances three times a weak	medium	low	hard	-0.699689278
26	4	1	other	2.5	mascular once a weak	high	low	hard	0.00383736
26	5	0	iran	0.5	mascular once a weak	medium	low	easy	-0.303092094
26	5	1	other	2.5	subcutances three times a weak	high	low	easy	-0.030902146

26	6	0	other	2.5	subcutances three times a weak	high	low	easy	-0.030902146
26	6	1	other	0.5	subcutances other days	high	medium	easy	-0.462940247
26	7	1	other	0.5	mascular once a weak	medium	medium	hard	-0.900801637
26	7	0	other	5.5	subcutances other days	medium	low	hard	-0.312098058
26	8	1	iran	2.5	mascular once a weak	high	low	hard	-0.0957683
26	8	0	iran	5.5	subcutances three times a weak	high	low	easy	-0.090441429
26	9	0	iran	2.5	subcutances three times a weak	medium	low	hard	-0.617903819
26	9	1	other	0.5	subcutances other days	high	medium	hard	-0.643869086
26	10	1	other	2.5	mascular once a weak	high	low	hard	0.00383736
26	10	0	iran	5.5	mascular once a weak	medium	medium	easy	-0.697626621
26	11	1	other	5.5	subcutances other days	high	medium	easy	-0.34108841
26	11	0	iran	0.5	subcutances three times a weak	medium	low	hard	-0.699689278
26	12	1	iran	5.5	subcutances three times a weak	high	medium	hard	-0.787756632
26	12	0	other	2.5	subcutances other days	medium	low	hard	-0.352164436
27	1	1	iran	5.5	mascular once a weak	high	medium	hard	-0.572088286
27	1	0	iran	0.5	subcutances three times a weak	high	medium	easy	-0.72867963
27	2	1	other	0.5	subcutances other days	high	medium	hard	-0.643869086
27	2	0	iran	5.5	subcutances other days	medium	medium	hard	-0.928090082
27	3	1	iran	5.5	mascular once a weak	high	medium	hard	-0.572088286
27	3	0	iran	2.5	subcutances three times a weak	high	medium	easy	-0.646894171
27	4	0	iran	0.5	subcutances three times a weak	medium	low	hard	-0.699689278
27	4	1	other	2.5	mascular once a weak	high	low	hard	0.00383736
27	5	1	iran	0.5	mascular once a weak	medium	low	easy	-0.303092094
27	5	0	other	2.5	subcutances three times a weak	high	low	easy	-0.030902146
27	6	1	other	2.5	subcutances three times a weak	high	low	easy	-0.030902146
27	6	0	other	0.5	subcutances other days	high	medium	easy	-0.462940247
27	7	1	other	0.5	mascular once a weak	medium	medium	hard	-0.900801637
27	7	0	other	5.5	subcutances other days	medium	low	hard	-0.312098058
27	8	1	iran	2.5	mascular once a weak	high	low	hard	-0.0957683
27	8	0	iran	5.5	subcutances three times a weak	high	low	easy	-0.090441429
27	9	0	iran	2.5	subcutances three times a weak	medium	low	hard	-0.617903819
27	9	1	other	0.5	subcutances other days	high	medium	hard	-0.643869086
27	10	1	other	2.5	mascular once a weak	high	low	hard	0.00383736
27	10	0	iran	5.5	mascular once a weak	medium	medium	easy	-0.697626621

27	11	1	other	5.5	subcutances other days	high	medium	easy	-0.34108841
27	11	0	iran	0.5	subcutances three times a weak	medium	low	hard	-0.699689278
27	12	1	iran	5.5	subcutances three times a weak	high	medium	hard	-0.787756632
27	12	0	other	2.5	subcutances other days	medium	low	hard	-0.352164436
28	1	0	iran	5.5	mascular once a weak	high	medium	hard	-0.572088286
28	1	1	iran	0.5	subcutances three times a weak	high	medium	easy	-0.72867963
28	2	1	other	0.5	subcutances other days	high	medium	hard	-0.643869086
28	2	0	iran	5.5	subcutances other days	medium	medium	hard	-0.928090082
28	3	0	iran	5.5	mascular once a weak	high	medium	hard	-0.572088286
28	3	1	iran	2.5	subcutances three times a weak	high	medium	easy	-0.646894171
28	4	0	iran	0.5	subcutances three times a weak	medium	low	hard	-0.699689278
28	4	1	other	2.5	mascular once a weak	high	low	hard	0.00383736
28	5	0	iran	0.5	mascular once a weak	medium	low	easy	-0.303092094
28	5	1	other	2.5	subcutances three times a weak	high	low	easy	-0.030902146
28	6	1	other	2.5	subcutances three times a weak	high	low	easy	-0.030902146
28	6	0	other	0.5	subcutances other days	high	medium	easy	-0.462940247
28	7	0	other	0.5	mascular once a weak	medium	medium	hard	-0.900801637
28	7	1	other	5.5	subcutances other days	medium	low	hard	-0.312098058
28	8	0	iran	2.5	mascular once a weak	high	low	hard	-0.0957683
28	8	1	iran	5.5	subcutances three times a weak	high	low	easy	-0.090441429
28	9	0	iran	2.5	subcutances three times a weak	medium	low	hard	-0.617903819
28	9	1	other	0.5	subcutances other days	high	medium	hard	-0.643869086
28	10	1	other	2.5	mascular once a weak	high	low	hard	0.00383736
28	10	0	iran	5.5	mascular once a weak	medium	medium	easy	-0.697626621
28	11	1	other	5.5	subcutances other days	high	medium	easy	-0.34108841
28	11	0	iran	0.5	subcutances three times a weak	medium	low	hard	-0.699689278
28	12	1	iran	5.5	subcutances three times a weak	high	medium	hard	-0.787756632
28	12	0	other	2.5	subcutances other days	medium	low	hard	-0.352164436
29	1	1	iran	5.5	mascular once a weak	high	medium	hard	-0.572088286
29	1	0	iran	0.5	subcutances three times a weak	high	medium	easy	-0.72867963
29	2	1	other	0.5	subcutances other days	high	medium	hard	-0.643869086
29	2	0	iran	5.5	subcutances other days	medium	medium	hard	-0.928090082
29	3	1	iran	5.5	mascular once a weak	high	medium	hard	-0.572088286
29	3	0	iran	2.5	subcutances three times a weak	high	medium	easy	-0.646894171

29	4	0	iran	0.5	subcutances	three times a weak	medium	low	hard	-0.699689278
29	4	1	other	2.5	mascular	once a weak	high	low	hard	0.00383736
29	5	0	iran	0.5	mascular	once a weak	medium	low	easy	-0.303092094
29	5	1	other	2.5	subcutances	three times a weak	high	low	easy	-0.030902146
29	6	1	other	2.5	subcutances	three times a weak	high	low	easy	-0.030902146
29	6	0	other	0.5	subcutances	other days	high	medium	easy	-0.462940247
29	7	0	other	0.5	mascular	once a weak	medium	medium	hard	-0.900801637
29	7	1	other	5.5	subcutances	other days	medium	low	hard	-0.312098058
29	8	0	iran	2.5	mascular	once a weak	high	low	hard	-0.0957683
29	8	1	iran	5.5	subcutances	three times a weak	high	low	easy	-0.090441429
29	9	0	iran	2.5	subcutances	three times a weak	medium	low	hard	-0.617903819
29	9	1	other	0.5	subcutances	other days	high	medium	hard	-0.643869086
29	10	1	other	2.5	mascular	once a weak	high	low	hard	0.00383736
29	10	0	iran	5.5	mascular	once a weak	medium	medium	easy	-0.697626621
29	11	1	other	5.5	subcutances	other days	high	medium	easy	-0.34108841
29	11	0	iran	0.5	subcutances	three times a weak	medium	low	hard	-0.699689278
29	12	1	iran	5.5	subcutances	three times a weak	high	medium	hard	-0.787756632
29	12	0	other	2.5	subcutances	other days	medium	low	hard	-0.352164436
30	1	1	iran	5.5	mascular	once a weak	high	medium	hard	-0.572088286
30	1	0	iran	0.5	subcutances	three times a weak	high	medium	easy	-0.72867963
30	2	1	other	0.5	subcutances	other days	high	medium	hard	-0.643869086
30	2	0	iran	5.5	subcutances	other days	medium	medium	hard	-0.928090082
30	3	1	iran	5.5	mascular	once a weak	high	medium	hard	-0.572088286
30	3	0	iran	2.5	subcutances	three times a weak	high	medium	easy	-0.646894171
30	4	0	iran	0.5	subcutances	three times a weak	medium	low	hard	-0.699689278
30	4	1	other	2.5	mascular	once a weak	high	low	hard	0.00383736
30	5	0	iran	0.5	mascular	once a weak	medium	low	easy	-0.303092094
30	5	1	other	2.5	subcutances	three times a weak	high	low	easy	-0.030902146
30	6	1	other	2.5	subcutances	three times a weak	high	low	easy	-0.030902146
30	6	0	other	0.5	subcutances	other days	high	medium	easy	-0.462940247
30	7	1	other	0.5	mascular	once a weak	medium	medium	hard	-0.900801637
30	7	0	other	5.5	subcutances	other days	medium	low	hard	-0.312098058
30	8	1	iran	2.5	mascular	once a weak	high	low	hard	-0.0957683
30	8	0	iran	5.5	subcutances	three times a weak	high	low	easy	-0.090441429

30	9	0	iran	2.5	subcutances three times a weak	medium	low	hard	-0.617903819
30	9	1	other	0.5	subcutances other days	high	medium	hard	-0.643869086
30	10	1	other	2.5	mascular once a weak	high	low	hard	0.00383736
30	10	0	iran	5.5	mascular once a weak	medium	medium	easy	-0.697626621
30	11	1	other	5.5	subcutances other days	high	medium	easy	-0.34108841
30	11	0	iran	0.5	subcutances three times a weak	medium	low	hard	-0.699689278
30	12	0	iran	5.5	subcutances three times a weak	high	medium	hard	-0.787756632
30	12	1	other	2.5	subcutances other days	medium	low	hard	-0.352164436
31	1	1	iran	5.5	mascular once a weak	high	medium	hard	-0.572088286
31	1	0	iran	0.5	subcutances three times a weak	high	medium	easy	-0.72867963
31	2	1	other	0.5	subcutances other days	high	medium	hard	-0.643869086
31	2	0	iran	5.5	subcutances other days	medium	medium	hard	-0.928090082
31	3	1	iran	5.5	mascular once a weak	high	medium	hard	-0.572088286
31	3	0	iran	2.5	subcutances three times a weak	high	medium	easy	-0.646894171
31	4	0	iran	0.5	subcutances three times a weak	medium	low	hard	-0.699689278
31	4	1	other	2.5	mascular once a weak	high	low	hard	0.00383736
31	5	0	iran	0.5	mascular once a weak	medium	low	easy	-0.303092094
31	5	1	other	2.5	subcutances three times a weak	high	low	easy	-0.030902146
31	6	1	other	2.5	subcutances three times a weak	high	low	easy	-0.030902146
31	6	0	other	0.5	subcutances other days	high	medium	easy	-0.462940247
31	7	0	other	0.5	mascular once a weak	medium	medium	hard	-0.900801637
31	7	1	other	5.5	subcutances other days	medium	low	hard	-0.312098058
31	8	1	iran	2.5	mascular once a weak	high	low	hard	-0.0957683
31	8	0	iran	5.5	subcutances three times a weak	high	low	easy	-0.090441429
31	9	1	iran	2.5	subcutances three times a weak	medium	low	hard	-0.617903819
31	9	0	other	0.5	subcutances other days	high	medium	hard	-0.643869086
31	10	1	other	2.5	mascular once a weak	high	low	hard	0.00383736
31	10	0	iran	5.5	mascular once a weak	medium	medium	easy	-0.697626621
31	11	1	other	5.5	subcutances other days	high	medium	easy	-0.34108841
31	11	0	iran	0.5	subcutances three times a weak	medium	low	hard	-0.699689278
31	12	1	iran	5.5	subcutances three times a weak	high	medium	hard	-0.787756632
31	12	0	other	2.5	subcutances other days	medium	low	hard	-0.352164436
32	1	0	iran	5.5	mascular once a weak	high	medium	hard	-0.572088286
32	1	1	iran	0.5	subcutances three times a weak	high	medium	easy	-0.72867963

32	2	1	other	0.5	subcutances	other days	high	medium	hard	-0.643869086
32	2	0	iran	5.5	subcutances	other days	medium	medium	hard	-0.928090082
32	3	0	iran	5.5	mascular	once a weak	high	medium	hard	-0.572088286
32	3	1	iran	2.5	subcutances	three times a weak	high	medium	easy	-0.646894171
32	4	0	iran	0.5	subcutances	three times a weak	medium	low	hard	-0.699689278
32	4	1	other	2.5	mascular	once a weak	high	low	hard	0.00383736
32	5	0	iran	0.5	mascular	once a weak	medium	low	easy	-0.303092094
32	5	1	other	2.5	subcutances	three times a weak	high	low	easy	-0.030902146
32	6	1	other	2.5	subcutances	three times a weak	high	low	easy	-0.030902146
32	6	0	other	0.5	subcutances	other days	high	medium	easy	-0.462940247
32	7	0	other	0.5	mascular	once a weak	medium	medium	hard	-0.900801637
32	7	1	other	5.5	subcutances	other days	medium	low	hard	-0.312098058
32	8	0	iran	2.5	mascular	once a weak	high	low	hard	-0.0957683
32	8	1	iran	5.5	subcutances	three times a weak	high	low	easy	-0.090441429
32	9	0	iran	2.5	subcutances	three times a weak	medium	low	hard	-0.617903819
32	9	1	other	0.5	subcutances	other days	high	medium	hard	-0.643869086
32	10	1	other	2.5	mascular	once a weak	high	low	hard	0.00383736
32	10	0	iran	5.5	mascular	once a weak	medium	medium	easy	-0.697626621
32	11	1	other	5.5	subcutances	other days	high	medium	easy	-0.34108841
32	11	0	iran	0.5	subcutances	three times a weak	medium	low	hard	-0.699689278
32	12	0	iran	5.5	subcutances	three times a weak	high	medium	hard	-0.787756632
32	12	1	other	2.5	subcutances	other days	medium	low	hard	-0.352164436
33	1	0	iran	5.5	mascular	once a weak	high	medium	hard	-0.572088286
33	1	1	iran	0.5	subcutances	three times a weak	high	medium	easy	-0.72867963
33	2	1	other	0.5	subcutances	other days	high	medium	hard	-0.643869086
33	2	0	iran	5.5	subcutances	other days	medium	medium	hard	-0.928090082
33	3	0	iran	5.5	mascular	once a weak	high	medium	hard	-0.572088286
33	3	1	iran	2.5	subcutances	three times a weak	high	medium	easy	-0.646894171
33	4	0	iran	0.5	subcutances	three times a weak	medium	low	hard	-0.699689278
33	4	1	other	2.5	mascular	once a weak	high	low	hard	0.00383736
33	5	0	iran	0.5	mascular	once a weak	medium	low	easy	-0.303092094
33	5	1	other	2.5	subcutances	three times a weak	high	low	easy	-0.030902146
33	6	1	other	2.5	subcutances	three times a weak	high	low	easy	-0.030902146
33	6	0	other	0.5	subcutances	other days	high	medium	easy	-0.462940247

33	7	0	other	0.5	mascular once a weak	medium	medium	hard	-0.900801637
33	7	1	other	5.5	subcutances other days	medium	low	hard	-0.312098058
33	8	0	iran	2.5	mascular once a weak	high	low	hard	-0.0957683
33	8	1	iran	5.5	subcutances three times a weak	high	low	easy	-0.090441429
33	9	1	iran	2.5	subcutances three times a weak	medium	low	hard	-0.617903819
33	9	0	other	0.5	subcutances other days	high	medium	hard	-0.643869086
33	10	1	other	2.5	mascular once a weak	high	low	hard	0.00383736
33	10	0	iran	5.5	mascular once a weak	medium	medium	easy	-0.697626621
33	11	1	other	5.5	subcutances other days	high	medium	easy	-0.34108841
33	11	0	iran	0.5	subcutances three times a weak	medium	low	hard	-0.699689278
33	12	1	iran	5.5	subcutances three times a weak	high	medium	hard	-0.787756632
33	12	0	other	2.5	subcutances other days	medium	low	hard	-0.352164436
34	1	1	iran	5.5	mascular once a weak	high	medium	hard	-0.572088286
34	1	0	iran	0.5	subcutances three times a weak	high	medium	easy	-0.72867963
34	2	1	other	0.5	subcutances other days	high	medium	hard	-0.643869086
34	2	0	iran	5.5	subcutances other days	medium	medium	hard	-0.928090082
34	3	1	iran	5.5	mascular once a weak	high	medium	hard	-0.572088286
34	3	0	iran	2.5	subcutances three times a weak	high	medium	easy	-0.646894171
34	4	0	iran	0.5	subcutances three times a weak	medium	low	hard	-0.699689278
34	4	1	other	2.5	mascular once a weak	high	low	hard	0.00383736
34	5	1	iran	0.5	mascular once a weak	medium	low	easy	-0.303092094
34	5	0	other	2.5	subcutances three times a weak	high	low	easy	-0.030902146
34	6	1	other	2.5	subcutances three times a weak	high	low	easy	-0.030902146
34	6	0	other	0.5	subcutances other days	high	medium	easy	-0.462940247
34	7	1	other	0.5	mascular once a weak	medium	medium	hard	-0.900801637
34	7	0	other	5.5	subcutances other days	medium	low	hard	-0.312098058
34	8	1	iran	2.5	mascular once a weak	high	low	hard	-0.0957683
34	8	0	iran	5.5	subcutances three times a weak	high	low	easy	-0.090441429
34	9	0	iran	2.5	subcutances three times a weak	medium	low	hard	-0.617903819
34	9	1	other	0.5	subcutances other days	high	medium	hard	-0.643869086
34	10	0	other	2.5	mascular once a weak	high	low	hard	0.00383736
34	10	1	iran	5.5	mascular once a weak	medium	medium	easy	-0.697626621
34	11	1	other	5.5	subcutances other days	high	medium	easy	-0.34108841
34	11	0	iran	0.5	subcutances three times a weak	medium	low	hard	-0.699689278

34	12	0	iran	5.5	subcutances three times a weak	high	medium	hard	-0.787756632
34	12	1	other	2.5	subcutances other days	medium	low	hard	-0.352164436
35	1	0	iran	5.5	mascular once a weak	high	medium	hard	-0.572088286
35	1	1	iran	0.5	subcutances three times a weak	high	medium	easy	-0.72867963
35	2	1	other	0.5	subcutances other days	high	medium	hard	-0.643869086
35	2	0	iran	5.5	subcutances other days	medium	medium	hard	-0.928090082
35	3	1	iran	5.5	mascular once a weak	high	medium	hard	-0.572088286
35	3	0	iran	2.5	subcutances three times a weak	high	medium	easy	-0.646894171
35	4	0	iran	0.5	subcutances three times a weak	medium	low	hard	-0.699689278
35	4	1	other	2.5	mascular once a weak	high	low	hard	0.00383736
35	5	0	iran	0.5	mascular once a weak	medium	low	easy	-0.303092094
35	5	1	other	2.5	subcutances three times a weak	high	low	easy	-0.030902146
35	6	1	other	2.5	subcutances three times a weak	high	low	easy	-0.030902146
35	6	0	other	0.5	subcutances other days	high	medium	easy	-0.462940247
35	7	0	other	0.5	mascular once a weak	medium	medium	hard	-0.900801637
35	7	1	other	5.5	subcutances other days	medium	low	hard	-0.312098058
35	8	0	iran	2.5	mascular once a weak	high	low	hard	-0.0957683
35	8	1	iran	5.5	subcutances three times a weak	high	low	easy	-0.090441429
35	9	0	iran	2.5	subcutances three times a weak	medium	low	hard	-0.617903819
35	9	1	other	0.5	subcutances other days	high	medium	hard	-0.643869086
35	10	1	other	2.5	mascular once a weak	high	low	hard	0.00383736
35	10	0	iran	5.5	mascular once a weak	medium	medium	easy	-0.697626621
35	11	0	other	5.5	subcutances other days	high	medium	easy	-0.34108841
35	11	1	iran	0.5	subcutances three times a weak	medium	low	hard	-0.699689278
35	12	1	iran	5.5	subcutances three times a weak	high	medium	hard	-0.787756632
35	12	0	other	2.5	subcutances other days	medium	low	hard	-0.352164436
36	1	1	iran	5.5	mascular once a weak	high	medium	hard	-0.572088286
36	1	0	iran	0.5	subcutances three times a weak	high	medium	easy	-0.72867963
36	2	1	other	0.5	subcutances other days	high	medium	hard	-0.643869086
36	2	0	iran	5.5	subcutances other days	medium	medium	hard	-0.928090082
36	3	0	iran	5.5	mascular once a weak	high	medium	hard	-0.572088286
36	3	1	iran	2.5	subcutances three times a weak	high	medium	easy	-0.646894171
36	4	0	iran	0.5	subcutances three times a weak	medium	low	hard	-0.699689278
36	4	1	other	2.5	mascular once a weak	high	low	hard	0.00383736

36	5	0	iran	0.5	mascular once a weak	medium	low	easy	-0.303092094
36	5	1	other	2.5	subcutances three times a weak	high	low	easy	-0.030902146
36	6	1	other	2.5	subcutances three times a weak	high	low	easy	-0.030902146
36	6	0	other	0.5	subcutances other days	high	medium	easy	-0.462940247
36	7	1	other	0.5	mascular once a weak	medium	medium	hard	-0.900801637
36	7	0	other	5.5	subcutances other days	medium	low	hard	-0.312098058
36	8	0	iran	2.5	mascular once a weak	high	low	hard	-0.0957683
36	8	1	iran	5.5	subcutances three times a weak	high	low	easy	-0.090441429
36	9	1	iran	2.5	subcutances three times a weak	medium	low	hard	-0.617903819
36	9	0	other	0.5	subcutances other days	high	medium	hard	-0.643869086
36	10	1	other	2.5	mascular once a weak	high	low	hard	0.00383736
36	10	0	iran	5.5	mascular once a weak	medium	medium	easy	-0.697626621
36	11	0	other	5.5	subcutances other days	high	medium	easy	-0.34108841
36	11	1	iran	0.5	subcutances three times a weak	medium	low	hard	-0.699689278
36	12	0	iran	5.5	subcutances three times a weak	high	medium	hard	-0.787756632
36	12	1	other	2.5	subcutances other days	medium	low	hard	-0.352164436
37	1	0	iran	5.5	mascular once a weak	high	medium	hard	-0.572088286
37	1	1	iran	0.5	subcutances three times a weak	high	medium	easy	-0.72867963
37	2	1	other	0.5	subcutances other days	high	medium	hard	-0.643869086
37	2	0	iran	5.5	subcutances other days	medium	medium	hard	-0.928090082
37	3	0	iran	5.5	mascular once a weak	high	medium	hard	-0.572088286
37	3	1	iran	2.5	subcutances three times a weak	high	medium	easy	-0.646894171
37	4	0	iran	0.5	subcutances three times a weak	medium	low	hard	-0.699689278
37	4	1	other	2.5	mascular once a weak	high	low	hard	0.00383736
37	5	0	iran	0.5	mascular once a weak	medium	low	easy	-0.303092094
37	5	1	other	2.5	subcutances three times a weak	high	low	easy	-0.030902146
37	6	1	other	2.5	subcutances three times a weak	high	low	easy	-0.030902146
37	6	0	other	0.5	subcutances other days	high	medium	easy	-0.462940247
37	7	0	other	0.5	mascular once a weak	medium	medium	hard	-0.900801637
37	7	1	other	5.5	subcutances other days	medium	low	hard	-0.312098058
37	8	0	iran	2.5	mascular once a weak	high	low	hard	-0.0957683
37	8	1	iran	5.5	subcutances three times a weak	high	low	easy	-0.090441429
37	9	1	iran	2.5	subcutances three times a weak	medium	low	hard	-0.617903819
37	9	0	other	0.5	subcutances other days	high	medium	hard	-0.643869086

37	10	1	other	2.5	mascular once a weak	high	low	hard	0.00383736
37	10	0	iran	5.5	mascular once a weak	medium	medium	easy	-0.697626621
37	11	1	other	5.5	subcutances other days	high	medium	easy	-0.34108841
37	11	0	iran	0.5	subcutances three times a weak	medium	low	hard	-0.699689278
37	12	1	iran	5.5	subcutances three times a weak	high	medium	hard	-0.787756632
37	12	0	other	2.5	subcutances other days	medium	low	hard	-0.352164436
38	1	1	iran	5.5	mascular once a weak	high	medium	hard	-0.572088286
38	1	0	iran	0.5	subcutances three times a weak	high	medium	easy	-0.72867963
38	2	1	other	0.5	subcutances other days	high	medium	hard	-0.643869086
38	2	0	iran	5.5	subcutances other days	medium	medium	hard	-0.928090082
38	3	1	iran	5.5	mascular once a weak	high	medium	hard	-0.572088286
38	3	0	iran	2.5	subcutances three times a weak	high	medium	easy	-0.646894171
38	4	0	iran	0.5	subcutances three times a weak	medium	low	hard	-0.699689278
38	4	1	other	2.5	mascular once a weak	high	low	hard	0.00383736
38	5	1	iran	0.5	mascular once a weak	medium	low	easy	-0.303092094
38	5	0	other	2.5	subcutances three times a weak	high	low	easy	-0.030902146
38	6	1	other	2.5	subcutances three times a weak	high	low	easy	-0.030902146
38	6	0	other	0.5	subcutances other days	high	medium	easy	-0.462940247
38	7	1	other	0.5	mascular once a weak	medium	medium	hard	-0.900801637
38	7	0	other	5.5	subcutances other days	medium	low	hard	-0.312098058
38	8	1	iran	2.5	mascular once a weak	high	low	hard	-0.0957683
38	8	0	iran	5.5	subcutances three times a weak	high	low	easy	-0.090441429
38	9	1	iran	2.5	subcutances three times a weak	medium	low	hard	-0.617903819
38	9	0	other	0.5	subcutances other days	high	medium	hard	-0.643869086
38	10	1	other	2.5	mascular once a weak	high	low	hard	0.00383736
38	10	0	iran	5.5	mascular once a weak	medium	medium	easy	-0.697626621
38	11	1	other	5.5	subcutances other days	high	medium	easy	-0.34108841
38	11	0	iran	0.5	subcutances three times a weak	medium	low	hard	-0.699689278
38	12	0	iran	5.5	subcutances three times a weak	high	medium	hard	-0.787756632
38	12	1	other	2.5	subcutances other days	medium	low	hard	-0.352164436
39	1	0	iran	5.5	mascular once a weak	high	medium	hard	-0.572088286
39	1	1	iran	0.5	subcutances three times a weak	high	medium	easy	-0.72867963
39	2	1	other	0.5	subcutances other days	high	medium	hard	-0.643869086
39	2	0	iran	5.5	subcutances other days	medium	medium	hard	-0.928090082

39	3	1	iran	5.5	mascular once a weak	high	medium	hard	-0.572088286
39	3	0	iran	2.5	subcutances three times a weak	high	medium	easy	-0.646894171
39	4	0	iran	0.5	subcutances three times a weak	medium	low	hard	-0.699689278
39	4	1	other	2.5	mascular once a weak	high	low	hard	0.00383736
39	5	0	iran	0.5	mascular once a weak	medium	low	easy	-0.303092094
39	5	1	other	2.5	subcutances three times a weak	high	low	easy	-0.030902146
39	6	1	other	2.5	subcutances three times a weak	high	low	easy	-0.030902146
39	6	0	other	0.5	subcutances other days	high	medium	easy	-0.462940247
39	7	0	other	0.5	mascular once a weak	medium	medium	hard	-0.900801637
39	7	1	other	5.5	subcutances other days	medium	low	hard	-0.312098058
39	8	0	iran	2.5	mascular once a weak	high	low	hard	-0.0957683
39	8	1	iran	5.5	subcutances three times a weak	high	low	easy	-0.090441429
39	9	0	iran	2.5	subcutances three times a weak	medium	low	hard	-0.617903819
39	9	1	other	0.5	subcutances other days	high	medium	hard	-0.643869086
39	10	1	other	2.5	mascular once a weak	high	low	hard	0.00383736
39	10	0	iran	5.5	mascular once a weak	medium	medium	easy	-0.697626621
39	11	1	other	5.5	subcutances other days	high	medium	easy	-0.34108841
39	11	0	iran	0.5	subcutances three times a weak	medium	low	hard	-0.699689278
39	12	1	iran	5.5	subcutances three times a weak	high	medium	hard	-0.787756632
39	12	0	other	2.5	subcutances other days	medium	low	hard	-0.352164436
40	1	1	iran	5.5	mascular once a weak	high	medium	hard	-0.572088286
40	1	0	iran	0.5	subcutances three times a weak	high	medium	easy	-0.72867963
40	2	1	other	0.5	subcutances other days	high	medium	hard	-0.643869086
40	2	0	iran	5.5	subcutances other days	medium	medium	hard	-0.928090082
40	3	0	iran	5.5	mascular once a weak	high	medium	hard	-0.572088286
40	3	1	iran	2.5	subcutances three times a weak	high	medium	easy	-0.646894171
40	4	0	iran	0.5	subcutances three times a weak	medium	low	hard	-0.699689278
40	4	1	other	2.5	mascular once a weak	high	low	hard	0.00383736
40	5	1	iran	0.5	mascular once a weak	medium	low	easy	-0.303092094
40	5	0	other	2.5	subcutances three times a weak	high	low	easy	-0.030902146
40	6	0	other	2.5	subcutances three times a weak	high	low	easy	-0.030902146
40	6	1	other	0.5	subcutances other days	high	medium	easy	-0.462940247
40	7	0	other	0.5	mascular once a weak	medium	medium	hard	-0.900801637
40	7	1	other	5.5	subcutances other days	medium	low	hard	-0.312098058

40	8	1	iran	2.5	mascular once a weak	high	low	hard	-0.0957683
40	8	0	iran	5.5	subcutances three times a weak	high	low	easy	-0.090441429
40	9	0	iran	2.5	subcutances three times a weak	medium	low	hard	-0.617903819
40	9	1	other	0.5	subcutances other days	high	medium	hard	-0.643869086
40	10	0	other	2.5	mascular once a weak	high	low	hard	0.00383736
40	10	1	iran	5.5	mascular once a weak	medium	medium	easy	-0.697626621
40	11	1	other	5.5	subcutances other days	high	medium	easy	-0.34108841
40	11	0	iran	0.5	subcutances three times a weak	medium	low	hard	-0.699689278
40	12	0	iran	5.5	subcutances three times a weak	high	medium	hard	-0.787756632
40	12	1	other	2.5	subcutances other days	medium	low	hard	-0.352164436
41	1	1	iran	5.5	mascular once a weak	high	medium	hard	-0.572088286
41	1	0	iran	0.5	subcutances three times a weak	high	medium	easy	-0.72867963
41	2	0	other	0.5	subcutances other days	high	medium	hard	-0.643869086
41	2	1	iran	5.5	subcutances other days	medium	medium	hard	-0.928090082
41	3	0	iran	5.5	mascular once a weak	high	medium	hard	-0.572088286
41	3	1	iran	2.5	subcutances three times a weak	high	medium	easy	-0.646894171
41	4	1	iran	0.5	subcutances three times a weak	medium	low	hard	-0.699689278
41	4	0	other	2.5	mascular once a weak	high	low	hard	0.00383736
41	5	1	iran	0.5	mascular once a weak	medium	low	easy	-0.303092094
41	5	0	other	2.5	subcutances three times a weak	high	low	easy	-0.030902146
41	6	0	other	2.5	subcutances three times a weak	high	low	easy	-0.030902146
41	6	1	other	0.5	subcutances other days	high	medium	easy	-0.462940247
41	7	0	other	0.5	mascular once a weak	medium	medium	hard	-0.900801637
41	7	1	other	5.5	subcutances other days	medium	low	hard	-0.312098058
41	8	1	iran	2.5	mascular once a weak	high	low	hard	-0.0957683
41	8	0	iran	5.5	subcutances three times a weak	high	low	easy	-0.090441429
41	9	1	iran	2.5	subcutances three times a weak	medium	low	hard	-0.617903819
41	9	0	other	0.5	subcutances other days	high	medium	hard	-0.643869086
41	10	0	other	2.5	mascular once a weak	high	low	hard	0.00383736
41	10	1	iran	5.5	mascular once a weak	medium	medium	easy	-0.697626621
41	11	0	other	5.5	subcutances other days	high	medium	easy	-0.34108841
41	11	1	iran	0.5	subcutances three times a weak	medium	low	hard	-0.699689278
41	12	0	iran	5.5	subcutances three times a weak	high	medium	hard	-0.787756632
41	12	1	other	2.5	subcutances other days	medium	low	hard	-0.352164436

42	1	1	iran	5.5	mascular once a weak	high	medium	hard	-0.572088286
42	1	0	iran	0.5	subcutances three times a weak	high	medium	easy	-0.72867963
42	2	0	other	0.5	subcutances other days	high	medium	hard	-0.643869086
42	2	1	iran	5.5	subcutances other days	medium	medium	hard	-0.928090082
42	3	0	iran	5.5	mascular once a weak	high	medium	hard	-0.572088286
42	3	1	iran	2.5	subcutances three times a weak	high	medium	easy	-0.646894171
42	4	0	iran	0.5	subcutances three times a weak	medium	low	hard	-0.699689278
42	4	1	other	2.5	mascular once a weak	high	low	hard	0.00383736
42	5	1	iran	0.5	mascular once a weak	medium	low	easy	-0.303092094
42	5	0	other	2.5	subcutances three times a weak	high	low	easy	-0.030902146
42	6	0	other	2.5	subcutances three times a weak	high	low	easy	-0.030902146
42	6	1	other	0.5	subcutances other days	high	medium	easy	-0.462940247
42	7	1	other	0.5	mascular once a weak	medium	medium	hard	-0.900801637
42	7	0	other	5.5	subcutances other days	medium	low	hard	-0.312098058
42	8	1	iran	2.5	mascular once a weak	high	low	hard	-0.0957683
42	8	0	iran	5.5	subcutances three times a weak	high	low	easy	-0.090441429
42	9	1	iran	2.5	subcutances three times a weak	medium	low	hard	-0.617903819
42	9	0	other	0.5	subcutances other days	high	medium	hard	-0.643869086
42	10	0	other	2.5	mascular once a weak	high	low	hard	0.00383736
42	10	1	iran	5.5	mascular once a weak	medium	medium	easy	-0.697626621
42	11	0	other	5.5	subcutances other days	high	medium	easy	-0.34108841
42	11	1	iran	0.5	subcutances three times a weak	medium	low	hard	-0.699689278
42	12	1	iran	5.5	subcutances three times a weak	high	medium	hard	-0.787756632
42	12	0	other	2.5	subcutances other days	medium	low	hard	-0.352164436
43	1	1	iran	5.5	mascular once a weak	high	medium	hard	-0.572088286
43	1	0	iran	0.5	subcutances three times a weak	high	medium	easy	-0.72867963
43	2	1	other	0.5	subcutances other days	high	medium	hard	-0.643869086
43	2	0	iran	5.5	subcutances other days	medium	medium	hard	-0.928090082
43	3	0	iran	5.5	mascular once a weak	high	medium	hard	-0.572088286
43	3	1	iran	2.5	subcutances three times a weak	high	medium	easy	-0.646894171
43	4	0	iran	0.5	subcutances three times a weak	medium	low	hard	-0.699689278
43	4	1	other	2.5	mascular once a weak	high	low	hard	0.00383736
43	5	0	iran	0.5	mascular once a weak	medium	low	easy	-0.303092094
43	5	1	other	2.5	subcutances three times a weak	high	low	easy	-0.030902146

43	6	0	other	2.5	subcutances three times a weak	high	low	easy	-0.030902146
43	6	1	other	0.5	subcutances other days	high	medium	easy	-0.462940247
43	7	0	other	0.5	mascular once a weak	medium	medium	hard	-0.900801637
43	7	1	other	5.5	subcutances other days	medium	low	hard	-0.312098058
43	8	1	iran	2.5	mascular once a weak	high	low	hard	-0.0957683
43	8	0	iran	5.5	subcutances three times a weak	high	low	easy	-0.090441429
43	9	0	iran	2.5	subcutances three times a weak	medium	low	hard	-0.617903819
43	9	1	other	0.5	subcutances other days	high	medium	hard	-0.643869086
43	10	1	other	2.5	mascular once a weak	high	low	hard	0.00383736
43	10	0	iran	5.5	mascular once a weak	medium	medium	easy	-0.697626621
43	11	1	other	5.5	subcutances other days	high	medium	easy	-0.34108841
43	11	0	iran	0.5	subcutances three times a weak	medium	low	hard	-0.699689278
43	12	1	iran	5.5	subcutances three times a weak	high	medium	hard	-0.787756632
43	12	0	other	2.5	subcutances other days	medium	low	hard	-0.352164436
44	1	1	iran	5.5	mascular once a weak	high	medium	hard	-0.572088286
44	1	0	iran	0.5	subcutances three times a weak	high	medium	easy	-0.72867963
44	2	0	other	0.5	subcutances other days	high	medium	hard	-0.643869086
44	2	1	iran	5.5	subcutances other days	medium	medium	hard	-0.928090082
44	3	0	iran	5.5	mascular once a weak	high	medium	hard	-0.572088286
44	3	1	iran	2.5	subcutances three times a weak	high	medium	easy	-0.646894171
44	4	0	iran	0.5	subcutances three times a weak	medium	low	hard	-0.699689278
44	4	1	other	2.5	mascular once a weak	high	low	hard	0.00383736
44	5	1	iran	0.5	mascular once a weak	medium	low	easy	-0.303092094
44	5	0	other	2.5	subcutances three times a weak	high	low	easy	-0.030902146
44	6	1	other	2.5	subcutances three times a weak	high	low	easy	-0.030902146
44	6	0	other	0.5	subcutances other days	high	medium	easy	-0.462940247
44	7	1	other	0.5	mascular once a weak	medium	medium	hard	-0.900801637
44	7	0	other	5.5	subcutances other days	medium	low	hard	-0.312098058
44	8	1	iran	2.5	mascular once a weak	high	low	hard	-0.0957683
44	8	0	iran	5.5	subcutances three times a weak	high	low	easy	-0.090441429
44	9	0	iran	2.5	subcutances three times a weak	medium	low	hard	-0.617903819
44	9	1	other	0.5	subcutances other days	high	medium	hard	-0.643869086
44	10	1	other	2.5	mascular once a weak	high	low	hard	0.00383736
44	10	0	iran	5.5	mascular once a weak	medium	medium	easy	-0.697626621

44	11	1	other	5.5	subcutances other days	high	medium	easy	-0.34108841
44	11	0	iran	0.5	subcutances three times a weak	medium	low	hard	-0.699689278
44	12	1	iran	5.5	subcutances three times a weak	high	medium	hard	-0.787756632
44	12	0	other	2.5	subcutances other days	medium	low	hard	-0.352164436
45	1	1	iran	5.5	mascular once a weak	high	medium	hard	-0.572088286
45	1	0	iran	0.5	subcutances three times a weak	high	medium	easy	-0.72867963
45	2	1	other	0.5	subcutances other days	high	medium	hard	-0.643869086
45	2	0	iran	5.5	subcutances other days	medium	medium	hard	-0.928090082
45	3	0	iran	5.5	mascular once a weak	high	medium	hard	-0.572088286
45	3	1	iran	2.5	subcutances three times a weak	high	medium	easy	-0.646894171
45	4	0	iran	0.5	subcutances three times a weak	medium	low	hard	-0.699689278
45	4	1	other	2.5	mascular once a weak	high	low	hard	0.00383736
45	5	1	iran	0.5	mascular once a weak	medium	low	easy	-0.303092094
45	5	0	other	2.5	subcutances three times a weak	high	low	easy	-0.030902146
45	6	1	other	2.5	subcutances three times a weak	high	low	easy	-0.030902146
45	6	0	other	0.5	subcutances other days	high	medium	easy	-0.462940247
45	7	0	other	0.5	mascular once a weak	medium	medium	hard	-0.900801637
45	7	1	other	5.5	subcutances other days	medium	low	hard	-0.312098058
45	8	1	iran	2.5	mascular once a weak	high	low	hard	-0.0957683
45	8	0	iran	5.5	subcutances three times a weak	high	low	easy	-0.090441429
45	9	1	iran	2.5	subcutances three times a weak	medium	low	hard	-0.617903819
45	9	0	other	0.5	subcutances other days	high	medium	hard	-0.643869086
45	10	1	other	2.5	mascular once a weak	high	low	hard	0.00383736
45	10	0	iran	5.5	mascular once a weak	medium	medium	easy	-0.697626621
45	11	1	other	5.5	subcutances other days	high	medium	easy	-0.34108841
45	11	0	iran	0.5	subcutances three times a weak	medium	low	hard	-0.699689278
45	12	1	iran	5.5	subcutances three times a weak	high	medium	hard	-0.787756632
45	12	0	other	2.5	subcutances other days	medium	low	hard	-0.352164436
46	1	1	iran	5.5	mascular once a weak	high	medium	hard	-0.572088286
46	1	0	iran	0.5	subcutances three times a weak	high	medium	easy	-0.72867963
46	2	0	other	0.5	subcutances other days	high	medium	hard	-0.643869086
46	2	1	iran	5.5	subcutances other days	medium	medium	hard	-0.928090082
46	3	0	iran	5.5	mascular once a weak	high	medium	hard	-0.572088286
46	3	1	iran	2.5	subcutances three times a weak	high	medium	easy	-0.646894171

46	4	1	iran	0.5	subcutances three times a weak	medium	low	hard	-0.699689278
46	4	0	other	2.5	mascular once a weak	high	low	hard	0.00383736
46	5	1	iran	0.5	mascular once a weak	medium	low	easy	-0.303092094
46	5	0	other	2.5	subcutances three times a weak	high	low	easy	-0.030902146
46	6	0	other	2.5	subcutances three times a weak	high	low	easy	-0.030902146
46	6	1	other	0.5	subcutances other days	high	medium	easy	-0.462940247
46	7	1	other	0.5	mascular once a weak	medium	medium	hard	-0.900801637
46	7	0	other	5.5	subcutances other days	medium	low	hard	-0.312098058
46	8	1	iran	2.5	mascular once a weak	high	low	hard	-0.0957683
46	8	0	iran	5.5	subcutances three times a weak	high	low	easy	-0.090441429
46	9	1	iran	2.5	subcutances three times a weak	medium	low	hard	-0.617903819
46	9	0	other	0.5	subcutances other days	high	medium	hard	-0.643869086
46	10	0	other	2.5	mascular once a weak	high	low	hard	0.00383736
46	10	1	iran	5.5	mascular once a weak	medium	medium	easy	-0.697626621
46	11	0	other	5.5	subcutances other days	high	medium	easy	-0.34108841
46	11	1	iran	0.5	subcutances three times a weak	medium	low	hard	-0.699689278
46	12	0	iran	5.5	subcutances three times a weak	high	medium	hard	-0.787756632
46	12	1	other	2.5	subcutances other days	medium	low	hard	-0.352164436
47	1	1	iran	5.5	mascular once a weak	high	medium	hard	-0.572088286
47	1	0	iran	0.5	subcutances three times a weak	high	medium	easy	-0.72867963
47	2	0	other	0.5	subcutances other days	high	medium	hard	-0.643869086
47	2	1	iran	5.5	subcutances other days	medium	medium	hard	-0.928090082
47	3	0	iran	5.5	mascular once a weak	high	medium	hard	-0.572088286
47	3	1	iran	2.5	subcutances three times a weak	high	medium	easy	-0.646894171
47	4	1	iran	0.5	subcutances three times a weak	medium	low	hard	-0.699689278
47	4	0	other	2.5	mascular once a weak	high	low	hard	0.00383736
47	5	1	iran	0.5	mascular once a weak	medium	low	easy	-0.303092094
47	5	0	other	2.5	subcutances three times a weak	high	low	easy	-0.030902146
47	6	0	other	2.5	subcutances three times a weak	high	low	easy	-0.030902146
47	6	1	other	0.5	subcutances other days	high	medium	easy	-0.462940247
47	7	0	other	0.5	mascular once a weak	medium	medium	hard	-0.900801637
47	7	1	other	5.5	subcutances other days	medium	low	hard	-0.312098058
47	8	1	iran	2.5	mascular once a weak	high	low	hard	-0.0957683
47	8	0	iran	5.5	subcutances three times a weak	high	low	easy	-0.090441429

47	9	1	iran	2.5	subcutances three times a weak	medium	low	hard	-0.617903819
47	9	0	other	0.5	subcutances other days	high	medium	hard	-0.643869086
47	10	0	other	2.5	mascular once a weak	high	low	hard	0.00383736
47	10	1	iran	5.5	mascular once a weak	medium	medium	easy	-0.697626621
47	11	0	other	5.5	subcutances other days	high	medium	easy	-0.34108841
47	11	1	iran	0.5	subcutances three times a weak	medium	low	hard	-0.699689278
47	12	0	iran	5.5	subcutances three times a weak	high	medium	hard	-0.787756632
47	12	1	other	2.5	subcutances other days	medium	low	hard	-0.352164436
48	1	1	iran	5.5	mascular once a weak	high	medium	hard	-0.572088286
48	1	0	iran	0.5	subcutances three times a weak	high	medium	easy	-0.72867963
48	2	0	other	0.5	subcutances other days	high	medium	hard	-0.643869086
48	2	1	iran	5.5	subcutances other days	medium	medium	hard	-0.928090082
48	3	0	iran	5.5	mascular once a weak	high	medium	hard	-0.572088286
48	3	1	iran	2.5	subcutances three times a weak	high	medium	easy	-0.646894171
48	4	1	iran	0.5	subcutances three times a weak	medium	low	hard	-0.699689278
48	4	0	other	2.5	mascular once a weak	high	low	hard	0.00383736
48	5	1	iran	0.5	mascular once a weak	medium	low	easy	-0.303092094
48	5	0	other	2.5	subcutances three times a weak	high	low	easy	-0.030902146
48	6	0	other	2.5	subcutances three times a weak	high	low	easy	-0.030902146
48	6	1	other	0.5	subcutances other days	high	medium	easy	-0.462940247
48	7	1	other	0.5	mascular once a weak	medium	medium	hard	-0.900801637
48	7	0	other	5.5	subcutances other days	medium	low	hard	-0.312098058
48	8	1	iran	2.5	mascular once a weak	high	low	hard	-0.0957683
48	8	0	iran	5.5	subcutances three times a weak	high	low	easy	-0.090441429
48	9	1	iran	2.5	subcutances three times a weak	medium	low	hard	-0.617903819
48	9	0	other	0.5	subcutances other days	high	medium	hard	-0.643869086
48	10	0	other	2.5	mascular once a weak	high	low	hard	0.00383736
48	10	1	iran	5.5	mascular once a weak	medium	medium	easy	-0.697626621
48	11	0	other	5.5	subcutances other days	high	medium	easy	-0.34108841
48	11	1	iran	0.5	subcutances three times a weak	medium	low	hard	-0.699689278
48	12	0	iran	5.5	subcutances three times a weak	high	medium	hard	-0.787756632
48	12	1	other	2.5	subcutances other days	medium	low	hard	-0.352164436
49	1	1	iran	5.5	mascular once a weak	high	medium	hard	-0.572088286
49	1	0	iran	0.5	subcutances three times a weak	high	medium	easy	-0.72867963

49	2	0	other	0.5	subcutances other days	high	medium	hard	-0.643869086
49	2	1	iran	5.5	subcutances other days	medium	medium	hard	-0.928090082
49	3	1	iran	5.5	mascular once a weak	high	medium	hard	-0.572088286
49	3	0	iran	2.5	subcutances three times a weak	high	medium	easy	-0.646894171
49	4	0	iran	0.5	subcutances three times a weak	medium	low	hard	-0.699689278
49	4	1	other	2.5	mascular once a weak	high	low	hard	0.00383736
49	5	1	iran	0.5	mascular once a weak	medium	low	easy	-0.303092094
49	5	0	other	2.5	subcutances three times a weak	high	low	easy	-0.030902146
49	6	1	other	2.5	subcutances three times a weak	high	low	easy	-0.030902146
49	6	0	other	0.5	subcutances other days	high	medium	easy	-0.462940247
49	7	1	other	0.5	mascular once a weak	medium	medium	hard	-0.900801637
49	7	0	other	5.5	subcutances other days	medium	low	hard	-0.312098058
49	8	1	iran	2.5	mascular once a weak	high	low	hard	-0.0957683
49	8	0	iran	5.5	subcutances three times a weak	high	low	easy	-0.090441429
49	9	1	iran	2.5	subcutances three times a weak	medium	low	hard	-0.617903819
49	9	0	other	0.5	subcutances other days	high	medium	hard	-0.643869086
49	10	1	other	2.5	mascular once a weak	high	low	hard	0.00383736
49	10	0	iran	5.5	mascular once a weak	medium	medium	easy	-0.697626621
49	11	0	other	5.5	subcutances other days	high	medium	easy	-0.34108841
49	11	1	iran	0.5	subcutances three times a weak	medium	low	hard	-0.699689278
49	12	0	iran	5.5	subcutances three times a weak	high	medium	hard	-0.787756632
49	12	1	other	2.5	subcutances other days	medium	low	hard	-0.352164436
50	1	0	iran	5.5	mascular once a weak	high	medium	hard	-0.572088286
50	1	1	iran	0.5	subcutances three times a weak	high	medium	easy	-0.72867963
50	2	0	other	0.5	subcutances other days	high	medium	hard	-0.643869086
50	2	1	iran	5.5	subcutances other days	medium	medium	hard	-0.928090082
50	3	1	iran	5.5	mascular once a weak	high	medium	hard	-0.572088286
50	3	0	iran	2.5	subcutances three times a weak	high	medium	easy	-0.646894171
50	4	1	iran	0.5	subcutances three times a weak	medium	low	hard	-0.699689278
50	4	0	other	2.5	mascular once a weak	high	low	hard	0.00383736
50	5	1	iran	0.5	mascular once a weak	medium	low	easy	-0.303092094
50	5	0	other	2.5	subcutances three times a weak	high	low	easy	-0.030902146
50	6	0	other	2.5	subcutances three times a weak	high	low	easy	-0.030902146
50	6	1	other	0.5	subcutances other days	high	medium	easy	-0.462940247

50	7	1	other	0.5	mascular once a weak	medium	medium	hard	-0.900801637
50	7	0	other	5.5	subcutances other days	medium	low	hard	-0.312098058
50	8	0	iran	2.5	mascular once a weak	high	low	hard	-0.0957683
50	8	1	iran	5.5	subcutances three times a weak	high	low	easy	-0.090441429
50	9	1	iran	2.5	subcutances three times a weak	medium	low	hard	-0.617903819
50	9	0	other	0.5	subcutances other days	high	medium	hard	-0.643869086
50	10	1	other	2.5	mascular once a weak	high	low	hard	0.00383736
50	10	0	iran	5.5	mascular once a weak	medium	medium	easy	-0.697626621
50	11	1	other	5.5	subcutances other days	high	medium	easy	-0.34108841
50	11	0	iran	0.5	subcutances three times a weak	medium	low	hard	-0.699689278
50	12	1	iran	5.5	subcutances three times a weak	high	medium	hard	-0.787756632
50	12	0	other	2.5	subcutances other days	medium	low	hard	-0.352164436
51	1	1	iran	5.5	mascular once a weak	high	medium	hard	-0.572088286
51	1	0	iran	0.5	subcutances three times a weak	high	medium	easy	-0.72867963
51	2	1	other	0.5	subcutances other days	high	medium	hard	-0.643869086
51	2	0	iran	5.5	subcutances other days	medium	medium	hard	-0.928090082
51	3	0	iran	5.5	mascular once a weak	high	medium	hard	-0.572088286
51	3	1	iran	2.5	subcutances three times a weak	high	medium	easy	-0.646894171
51	4	0	iran	0.5	subcutances three times a weak	medium	low	hard	-0.699689278
51	4	1	other	2.5	mascular once a weak	high	low	hard	0.00383736
51	5	1	iran	0.5	mascular once a weak	medium	low	easy	-0.303092094
51	5	0	other	2.5	subcutances three times a weak	high	low	easy	-0.030902146
51	6	0	other	2.5	subcutances three times a weak	high	low	easy	-0.030902146
51	6	1	other	0.5	subcutances other days	high	medium	easy	-0.462940247
51	7	0	other	0.5	mascular once a weak	medium	medium	hard	-0.900801637
51	7	1	other	5.5	subcutances other days	medium	low	hard	-0.312098058
51	8	1	iran	2.5	mascular once a weak	high	low	hard	-0.0957683
51	8	0	iran	5.5	subcutances three times a weak	high	low	easy	-0.090441429
51	9	0	iran	2.5	subcutances three times a weak	medium	low	hard	-0.617903819
51	9	1	other	0.5	subcutances other days	high	medium	hard	-0.643869086
51	10	1	other	2.5	mascular once a weak	high	low	hard	0.00383736
51	10	0	iran	5.5	mascular once a weak	medium	medium	easy	-0.697626621
51	11	0	other	5.5	subcutances other days	high	medium	easy	-0.34108841
51	11	1	iran	0.5	subcutances three times a weak	medium	low	hard	-0.699689278

51	12	0	iran	5.5	subcutances three times a weak	high	medium	hard	-0.787756632
51	12	1	other	2.5	subcutances other days	medium	low	hard	-0.352164436
52	1	1	iran	5.5	mascular once a weak	high	medium	hard	-0.572088286
52	1	0	iran	0.5	subcutances three times a weak	high	medium	easy	-0.72867963
52	2	1	other	0.5	subcutances other days	high	medium	hard	-0.643869086
52	2	0	iran	5.5	subcutances other days	medium	medium	hard	-0.928090082
52	3	1	iran	5.5	mascular once a weak	high	medium	hard	-0.572088286
52	3	0	iran	2.5	subcutances three times a weak	high	medium	easy	-0.646894171
52	4	0	iran	0.5	subcutances three times a weak	medium	low	hard	-0.699689278
52	4	1	other	2.5	mascular once a weak	high	low	hard	0.00383736
52	5	1	iran	0.5	mascular once a weak	medium	low	easy	-0.303092094
52	5	0	other	2.5	subcutances three times a weak	high	low	easy	-0.030902146
52	6	1	other	2.5	subcutances three times a weak	high	low	easy	-0.030902146
52	6	0	other	0.5	subcutances other days	high	medium	easy	-0.462940247
52	7	0	other	0.5	mascular once a weak	medium	medium	hard	-0.900801637
52	7	1	other	5.5	subcutances other days	medium	low	hard	-0.312098058
52	8	1	iran	2.5	mascular once a weak	high	low	hard	-0.0957683
52	8	0	iran	5.5	subcutances three times a weak	high	low	easy	-0.090441429
52	9	1	iran	2.5	subcutances three times a weak	medium	low	hard	-0.617903819
52	9	0	other	0.5	subcutances other days	high	medium	hard	-0.643869086
52	10	1	other	2.5	mascular once a weak	high	low	hard	0.00383736
52	10	0	iran	5.5	mascular once a weak	medium	medium	easy	-0.697626621
52	11	0	other	5.5	subcutances other days	high	medium	easy	-0.34108841
52	11	1	iran	0.5	subcutances three times a weak	medium	low	hard	-0.699689278
52	12	0	iran	5.5	subcutances three times a weak	high	medium	hard	-0.787756632
52	12	1	other	2.5	subcutances other days	medium	low	hard	-0.352164436
53	1	0	iran	5.5	mascular once a weak	high	medium	hard	-0.572088286
53	1	1	iran	0.5	subcutances three times a weak	high	medium	easy	-0.72867963
53	2	0	other	0.5	subcutances other days	high	medium	hard	-0.643869086
53	2	1	iran	5.5	subcutances other days	medium	medium	hard	-0.928090082
53	3	0	iran	5.5	mascular once a weak	high	medium	hard	-0.572088286
53	3	1	iran	2.5	subcutances three times a weak	high	medium	easy	-0.646894171
53	4	1	iran	0.5	subcutances three times a weak	medium	low	hard	-0.699689278
53	4	0	other	2.5	mascular once a weak	high	low	hard	0.00383736

53	5	0	iran	0.5	mascular once a weak	medium	low	easy	-0.303092094
53	5	1	other	2.5	subcutances three times a weak	high	low	easy	-0.030902146
53	6	0	other	2.5	subcutances three times a weak	high	low	easy	-0.030902146
53	6	1	other	0.5	subcutances other days	high	medium	easy	-0.462940247
53	7	0	other	0.5	mascular once a weak	medium	medium	hard	-0.900801637
53	7	1	other	5.5	subcutances other days	medium	low	hard	-0.312098058
53	8	1	iran	2.5	mascular once a weak	high	low	hard	-0.0957683
53	8	0	iran	5.5	subcutances three times a weak	high	low	easy	-0.090441429
53	9	0	iran	2.5	subcutances three times a weak	medium	low	hard	-0.617903819
53	9	1	other	0.5	subcutances other days	high	medium	hard	-0.643869086
53	10	0	other	2.5	mascular once a weak	high	low	hard	0.00383736
53	10	1	iran	5.5	mascular once a weak	medium	medium	easy	-0.697626621
53	11	1	other	5.5	subcutances other days	high	medium	easy	-0.34108841
53	11	0	iran	0.5	subcutances three times a weak	medium	low	hard	-0.699689278
53	12	0	iran	5.5	subcutances three times a weak	high	medium	hard	-0.787756632
53	12	1	other	2.5	subcutances other days	medium	low	hard	-0.352164436
54	1	0	iran	5.5	mascular once a weak	high	medium	hard	-0.572088286
54	1	1	iran	0.5	subcutances three times a weak	high	medium	easy	-0.72867963
54	2	1	other	0.5	subcutances other days	high	medium	hard	-0.643869086
54	2	0	iran	5.5	subcutances other days	medium	medium	hard	-0.928090082
54	3	0	iran	5.5	mascular once a weak	high	medium	hard	-0.572088286
54	3	1	iran	2.5	subcutances three times a weak	high	medium	easy	-0.646894171
54	4	1	iran	0.5	subcutances three times a weak	medium	low	hard	-0.699689278
54	4	0	other	2.5	mascular once a weak	high	low	hard	0.00383736
54	5	1	iran	0.5	mascular once a weak	medium	low	easy	-0.303092094
54	5	0	other	2.5	subcutances three times a weak	high	low	easy	-0.030902146
54	6	0	other	2.5	subcutances three times a weak	high	low	easy	-0.030902146
54	6	1	other	0.5	subcutances other days	high	medium	easy	-0.462940247
54	7	0	other	0.5	mascular once a weak	medium	medium	hard	-0.900801637
54	7	1	other	5.5	subcutances other days	medium	low	hard	-0.312098058
54	8	0	iran	2.5	mascular once a weak	high	low	hard	-0.0957683
54	8	1	iran	5.5	subcutances three times a weak	high	low	easy	-0.090441429
54	9	0	iran	2.5	subcutances three times a weak	medium	low	hard	-0.617903819
54	9	1	other	0.5	subcutances other days	high	medium	hard	-0.643869086

54	10	1	other	2.5	mascular once a weak	high	low	hard	0.00383736
54	10	0	iran	5.5	mascular once a weak	medium	medium	easy	-0.697626621
54	11	1	other	5.5	subcutances other days	high	medium	easy	-0.34108841
54	11	0	iran	0.5	subcutances three times a weak	medium	low	hard	-0.699689278
54	12	1	iran	5.5	subcutances three times a weak	high	medium	hard	-0.787756632
54	12	0	other	2.5	subcutances other days	medium	low	hard	-0.352164436
55	1	1	iran	5.5	mascular once a weak	high	medium	hard	-0.572088286
55	1	0	iran	0.5	subcutances three times a weak	high	medium	easy	-0.72867963
55	2	0	other	0.5	subcutances other days	high	medium	hard	-0.643869086
55	2	1	iran	5.5	subcutances other days	medium	medium	hard	-0.928090082
55	3	0	iran	5.5	mascular once a weak	high	medium	hard	-0.572088286
55	3	1	iran	2.5	subcutances three times a weak	high	medium	easy	-0.646894171
55	4	0	iran	0.5	subcutances three times a weak	medium	low	hard	-0.699689278
55	4	1	other	2.5	mascular once a weak	high	low	hard	0.00383736
55	5	0	iran	0.5	mascular once a weak	medium	low	easy	-0.303092094
55	5	1	other	2.5	subcutances three times a weak	high	low	easy	-0.030902146
55	6	0	other	2.5	subcutances three times a weak	high	low	easy	-0.030902146
55	6	1	other	0.5	subcutances other days	high	medium	easy	-0.462940247
55	7	0	other	0.5	mascular once a weak	medium	medium	hard	-0.900801637
55	7	1	other	5.5	subcutances other days	medium	low	hard	-0.312098058
55	8	1	iran	2.5	mascular once a weak	high	low	hard	-0.0957683
55	8	0	iran	5.5	subcutances three times a weak	high	low	easy	-0.090441429
55	9	1	iran	2.5	subcutances three times a weak	medium	low	hard	-0.617903819
55	9	0	other	0.5	subcutances other days	high	medium	hard	-0.643869086
55	10	0	other	2.5	mascular once a weak	high	low	hard	0.00383736
55	10	1	iran	5.5	mascular once a weak	medium	medium	easy	-0.697626621
55	11	0	other	5.5	subcutances other days	high	medium	easy	-0.34108841
55	11	1	iran	0.5	subcutances three times a weak	medium	low	hard	-0.699689278
55	12	0	iran	5.5	subcutances three times a weak	high	medium	hard	-0.787756632
55	12	1	other	2.5	subcutances other days	medium	low	hard	-0.352164436
56	1	0	iran	5.5	mascular once a weak	high	medium	hard	-0.572088286
56	1	1	iran	0.5	subcutances three times a weak	high	medium	easy	-0.72867963
56	2	1	other	0.5	subcutances other days	high	medium	hard	-0.643869086
56	2	0	iran	5.5	subcutances other days	medium	medium	hard	-0.928090082

56	3	0	iran	5.5	mascular once a weak	high	medium	hard	-0.572088286
56	3	1	iran	2.5	subcutances three times a weak	high	medium	easy	-0.646894171
56	4	0	iran	0.5	subcutances three times a weak	medium	low	hard	-0.699689278
56	4	1	other	2.5	mascular once a weak	high	low	hard	0.00383736
56	5	0	iran	0.5	mascular once a weak	medium	low	easy	-0.303092094
56	5	1	other	2.5	subcutances three times a weak	high	low	easy	-0.030902146
56	6	1	other	2.5	subcutances three times a weak	high	low	easy	-0.030902146
56	6	0	other	0.5	subcutances other days	high	medium	easy	-0.462940247
56	7	0	other	0.5	mascular once a weak	medium	medium	hard	-0.900801637
56	7	1	other	5.5	subcutances other days	medium	low	hard	-0.312098058
56	8	0	iran	2.5	mascular once a weak	high	low	hard	-0.0957683
56	8	1	iran	5.5	subcutances three times a weak	high	low	easy	-0.090441429
56	9	1	iran	2.5	subcutances three times a weak	medium	low	hard	-0.617903819
56	9	0	other	0.5	subcutances other days	high	medium	hard	-0.643869086
56	10	1	other	2.5	mascular once a weak	high	low	hard	0.00383736
56	10	0	iran	5.5	mascular once a weak	medium	medium	easy	-0.697626621
56	11	1	other	5.5	subcutances other days	high	medium	easy	-0.34108841
56	11	0	iran	0.5	subcutances three times a weak	medium	low	hard	-0.699689278
56	12	1	iran	5.5	subcutances three times a weak	high	medium	hard	-0.787756632
56	12	0	other	2.5	subcutances other days	medium	low	hard	-0.352164436
57	1	0	iran	5.5	mascular once a weak	high	medium	hard	-0.572088286
57	1	1	iran	0.5	subcutances three times a weak	high	medium	easy	-0.72867963
57	2	1	other	0.5	subcutances other days	high	medium	hard	-0.643869086
57	2	0	iran	5.5	subcutances other days	medium	medium	hard	-0.928090082
57	3	0	iran	5.5	mascular once a weak	high	medium	hard	-0.572088286
57	3	1	iran	2.5	subcutances three times a weak	high	medium	easy	-0.646894171
57	4	0	iran	0.5	subcutances three times a weak	medium	low	hard	-0.699689278
57	4	1	other	2.5	mascular once a weak	high	low	hard	0.00383736
57	5	0	iran	0.5	mascular once a weak	medium	low	easy	-0.303092094
57	5	1	other	2.5	subcutances three times a weak	high	low	easy	-0.030902146
57	6	1	other	2.5	subcutances three times a weak	high	low	easy	-0.030902146
57	6	0	other	0.5	subcutances other days	high	medium	easy	-0.462940247
57	7	0	other	0.5	mascular once a weak	medium	medium	hard	-0.900801637
57	7	1	other	5.5	subcutances other days	medium	low	hard	-0.312098058

57	8	0	iran	2.5	mascular once a weak	high	low	hard	-0.0957683
57	8	1	iran	5.5	subcutances three times a weak	high	low	easy	-0.090441429
57	9	1	iran	2.5	subcutances three times a weak	medium	low	hard	-0.617903819
57	9	0	other	0.5	subcutances other days	high	medium	hard	-0.643869086
57	10	1	other	2.5	mascular once a weak	high	low	hard	0.00383736
57	10	0	iran	5.5	mascular once a weak	medium	medium	easy	-0.697626621
57	11	0	other	5.5	subcutances other days	high	medium	easy	-0.34108841
57	11	1	iran	0.5	subcutances three times a weak	medium	low	hard	-0.699689278
57	12	0	iran	5.5	subcutances three times a weak	high	medium	hard	-0.787756632
57	12	1	other	2.5	subcutances other days	medium	low	hard	-0.352164436
58	1	0	iran	5.5	mascular once a weak	high	medium	hard	-0.572088286
58	1	1	iran	0.5	subcutances three times a weak	high	medium	easy	-0.72867963
58	2	1	other	0.5	subcutances other days	high	medium	hard	-0.643869086
58	2	0	iran	5.5	subcutances other days	medium	medium	hard	-0.928090082
58	3	0	iran	5.5	mascular once a weak	high	medium	hard	-0.572088286
58	3	1	iran	2.5	subcutances three times a weak	high	medium	easy	-0.646894171
58	4	0	iran	0.5	subcutances three times a weak	medium	low	hard	-0.699689278
58	4	1	other	2.5	mascular once a weak	high	low	hard	0.00383736
58	5	0	iran	0.5	mascular once a weak	medium	low	easy	-0.303092094
58	5	1	other	2.5	subcutances three times a weak	high	low	easy	-0.030902146
58	6	1	other	2.5	subcutances three times a weak	high	low	easy	-0.030902146
58	6	0	other	0.5	subcutances other days	high	medium	easy	-0.462940247
58	7	0	other	0.5	mascular once a weak	medium	medium	hard	-0.900801637
58	7	1	other	5.5	subcutances other days	medium	low	hard	-0.312098058
58	8	0	iran	2.5	mascular once a weak	high	low	hard	-0.0957683
58	8	1	iran	5.5	subcutances three times a weak	high	low	easy	-0.090441429
58	9	0	iran	2.5	subcutances three times a weak	medium	low	hard	-0.617903819
58	9	1	other	0.5	subcutances other days	high	medium	hard	-0.643869086
58	10	1	other	2.5	mascular once a weak	high	low	hard	0.00383736
58	10	0	iran	5.5	mascular once a weak	medium	medium	easy	-0.697626621
58	11	1	other	5.5	subcutances other days	high	medium	easy	-0.34108841
58	11	0	iran	0.5	subcutances three times a weak	medium	low	hard	-0.699689278
58	12	1	iran	5.5	subcutances three times a weak	high	medium	hard	-0.787756632
58	12	0	other	2.5	subcutances other days	medium	low	hard	-0.352164436

59	1	1	iran	5.5	mascular once a weak	high	medium	hard	-0.572088286
59	1	0	iran	0.5	subcutances three times a weak	high	medium	easy	-0.72867963
59	2	1	other	0.5	subcutances other days	high	medium	hard	-0.643869086
59	2	0	iran	5.5	subcutances other days	medium	medium	hard	-0.928090082
59	3	0	iran	5.5	mascular once a weak	high	medium	hard	-0.572088286
59	3	1	iran	2.5	subcutances three times a weak	high	medium	easy	-0.646894171
59	4	0	iran	0.5	subcutances three times a weak	medium	low	hard	-0.699689278
59	4	1	other	2.5	mascular once a weak	high	low	hard	0.00383736
59	5	0	iran	0.5	mascular once a weak	medium	low	easy	-0.303092094
59	5	1	other	2.5	subcutances three times a weak	high	low	easy	-0.030902146
59	6	0	other	2.5	subcutances three times a weak	high	low	easy	-0.030902146
59	6	1	other	0.5	subcutances other days	high	medium	easy	-0.462940247
59	7	0	other	0.5	mascular once a weak	medium	medium	hard	-0.900801637
59	7	1	other	5.5	subcutances other days	medium	low	hard	-0.312098058
59	8	0	iran	2.5	mascular once a weak	high	low	hard	-0.0957683
59	8	1	iran	5.5	subcutances three times a weak	high	low	easy	-0.090441429
59	9	0	iran	2.5	subcutances three times a weak	medium	low	hard	-0.617903819
59	9	1	other	0.5	subcutances other days	high	medium	hard	-0.643869086
59	10	1	other	2.5	mascular once a weak	high	low	hard	0.00383736
59	10	0	iran	5.5	mascular once a weak	medium	medium	easy	-0.697626621
59	11	1	other	5.5	subcutances other days	high	medium	easy	-0.34108841
59	11	0	iran	0.5	subcutances three times a weak	medium	low	hard	-0.699689278
59	12	1	iran	5.5	subcutances three times a weak	high	medium	hard	-0.787756632
59	12	0	other	2.5	subcutances other days	medium	low	hard	-0.352164436
60	1	1	iran	5.5	mascular once a weak	high	medium	hard	-0.572088286
60	1	0	iran	0.5	subcutances three times a weak	high	medium	easy	-0.72867963
60	2	0	other	0.5	subcutances other days	high	medium	hard	-0.643869086
60	2	1	iran	5.5	subcutances other days	medium	medium	hard	-0.928090082
60	3	0	iran	5.5	mascular once a weak	high	medium	hard	-0.572088286
60	3	1	iran	2.5	subcutances three times a weak	high	medium	easy	-0.646894171
60	4	1	iran	0.5	subcutances three times a weak	medium	low	hard	-0.699689278
60	4	0	other	2.5	mascular once a weak	high	low	hard	0.00383736
60	5	1	iran	0.5	mascular once a weak	medium	low	easy	-0.303092094
60	5	0	other	2.5	subcutances three times a weak	high	low	easy	-0.030902146

60	6	0	other	2.5	subcutances three times a weak	high	low	easy	-0.030902146
60	6	1	other	0.5	subcutances other days	high	medium	easy	-0.462940247
60	7	1	other	0.5	mascular once a weak	medium	medium	hard	-0.900801637
60	7	0	other	5.5	subcutances other days	medium	low	hard	-0.312098058
60	8	1	iran	2.5	mascular once a weak	high	low	hard	-0.0957683
60	8	0	iran	5.5	subcutances three times a weak	high	low	easy	-0.090441429
60	9	1	iran	2.5	subcutances three times a weak	medium	low	hard	-0.617903819
60	9	0	other	0.5	subcutances other days	high	medium	hard	-0.643869086
60	10	0	other	2.5	mascular once a weak	high	low	hard	0.00383736
60	10	1	iran	5.5	mascular once a weak	medium	medium	easy	-0.697626621
60	11	0	other	5.5	subcutances other days	high	medium	easy	-0.34108841
60	11	1	iran	0.5	subcutances three times a weak	medium	low	hard	-0.699689278
60	12	0	iran	5.5	subcutances three times a weak	high	medium	hard	-0.787756632
60	12	1	other	2.5	subcutances other days	medium	low	hard	-0.352164436
61	1	1	iran	5.5	mascular once a weak	high	medium	hard	-0.572088286
61	1	0	iran	0.5	subcutances three times a weak	high	medium	easy	-0.72867963
61	2	1	other	0.5	subcutances other days	high	medium	hard	-0.643869086
61	2	0	iran	5.5	subcutances other days	medium	medium	hard	-0.928090082
61	3	1	iran	5.5	mascular once a weak	high	medium	hard	-0.572088286
61	3	0	iran	2.5	subcutances three times a weak	high	medium	easy	-0.646894171
61	4	0	iran	0.5	subcutances three times a weak	medium	low	hard	-0.699689278
61	4	1	other	2.5	mascular once a weak	high	low	hard	0.00383736
61	5	1	iran	0.5	mascular once a weak	medium	low	easy	-0.303092094
61	5	0	other	2.5	subcutances three times a weak	high	low	easy	-0.030902146
61	6	1	other	2.5	subcutances three times a weak	high	low	easy	-0.030902146
61	6	0	other	0.5	subcutances other days	high	medium	easy	-0.462940247
61	7	1	other	0.5	mascular once a weak	medium	medium	hard	-0.900801637
61	7	0	other	5.5	subcutances other days	medium	low	hard	-0.312098058
61	8	1	iran	2.5	mascular once a weak	high	low	hard	-0.0957683
61	8	0	iran	5.5	subcutances three times a weak	high	low	easy	-0.090441429
61	9	0	iran	2.5	subcutances three times a weak	medium	low	hard	-0.617903819
61	9	1	other	0.5	subcutances other days	high	medium	hard	-0.643869086
61	10	0	other	2.5	mascular once a weak	high	low	hard	0.00383736
61	10	1	iran	5.5	mascular once a weak	medium	medium	easy	-0.697626621

61	11	1	other	5.5	subcutances other days	high	medium	easy	-0.34108841
61	11	0	iran	0.5	subcutances three times a weak	medium	low	hard	-0.699689278
61	12	0	iran	5.5	subcutances three times a weak	high	medium	hard	-0.787756632
61	12	1	other	2.5	subcutances other days	medium	low	hard	-0.352164436
62	1	0	iran	5.5	mascular once a weak	high	medium	hard	-0.572088286
62	1	1	iran	0.5	subcutances three times a weak	high	medium	easy	-0.72867963
62	2	1	other	0.5	subcutances other days	high	medium	hard	-0.643869086
62	2	0	iran	5.5	subcutances other days	medium	medium	hard	-0.928090082
62	3	1	iran	5.5	mascular once a weak	high	medium	hard	-0.572088286
62	3	0	iran	2.5	subcutances three times a weak	high	medium	easy	-0.646894171
62	4	0	iran	0.5	subcutances three times a weak	medium	low	hard	-0.699689278
62	4	1	other	2.5	mascular once a weak	high	low	hard	0.00383736
62	5	0	iran	0.5	mascular once a weak	medium	low	easy	-0.303092094
62	5	1	other	2.5	subcutances three times a weak	high	low	easy	-0.030902146
62	6	1	other	2.5	subcutances three times a weak	high	low	easy	-0.030902146
62	6	0	other	0.5	subcutances other days	high	medium	easy	-0.462940247
62	7	0	other	0.5	mascular once a weak	medium	medium	hard	-0.900801637
62	7	1	other	5.5	subcutances other days	medium	low	hard	-0.312098058
62	8	0	iran	2.5	mascular once a weak	high	low	hard	-0.0957683
62	8	1	iran	5.5	subcutances three times a weak	high	low	easy	-0.090441429
62	9	0	iran	2.5	subcutances three times a weak	medium	low	hard	-0.617903819
62	9	1	other	0.5	subcutances other days	high	medium	hard	-0.643869086
62	10	1	other	2.5	mascular once a weak	high	low	hard	0.00383736
62	10	0	iran	5.5	mascular once a weak	medium	medium	easy	-0.697626621
62	11	1	other	5.5	subcutances other days	high	medium	easy	-0.34108841
62	11	0	iran	0.5	subcutances three times a weak	medium	low	hard	-0.699689278
62	12	0	iran	5.5	subcutances three times a weak	high	medium	hard	-0.787756632
62	12	1	other	2.5	subcutances other days	medium	low	hard	-0.352164436
63	1	0	iran	5.5	mascular once a weak	high	medium	hard	-0.572088286
63	1	1	iran	0.5	subcutances three times a weak	high	medium	easy	-0.72867963
63	2	1	other	0.5	subcutances other days	high	medium	hard	-0.643869086
63	2	0	iran	5.5	subcutances other days	medium	medium	hard	-0.928090082
63	3	0	iran	5.5	mascular once a weak	high	medium	hard	-0.572088286
63	3	1	iran	2.5	subcutances three times a weak	high	medium	easy	-0.646894171

63	4	0	iran	0.5	subcutances three times a weak	medium	low	hard	-0.699689278
63	4	1	other	2.5	mascular once a weak	high	low	hard	0.00383736
63	5	0	iran	0.5	mascular once a weak	medium	low	easy	-0.303092094
63	5	1	other	2.5	subcutances three times a weak	high	low	easy	-0.030902146
63	6	1	other	2.5	subcutances three times a weak	high	low	easy	-0.030902146
63	6	0	other	0.5	subcutances other days	high	medium	easy	-0.462940247
63	7	0	other	0.5	mascular once a weak	medium	medium	hard	-0.900801637
63	7	1	other	5.5	subcutances other days	medium	low	hard	-0.312098058
63	8	0	iran	2.5	mascular once a weak	high	low	hard	-0.0957683
63	8	1	iran	5.5	subcutances three times a weak	high	low	easy	-0.090441429
63	9	1	iran	2.5	subcutances three times a weak	medium	low	hard	-0.617903819
63	9	0	other	0.5	subcutances other days	high	medium	hard	-0.643869086
63	10	1	other	2.5	mascular once a weak	high	low	hard	0.00383736
63	10	0	iran	5.5	mascular once a weak	medium	medium	easy	-0.697626621
63	11	1	other	5.5	subcutances other days	high	medium	easy	-0.34108841
63	11	0	iran	0.5	subcutances three times a weak	medium	low	hard	-0.699689278
63	12	1	iran	5.5	subcutances three times a weak	high	medium	hard	-0.787756632
63	12	0	other	2.5	subcutances other days	medium	low	hard	-0.352164436
64	1	0	iran	5.5	mascular once a weak	high	medium	hard	-0.572088286
64	1	1	iran	0.5	subcutances three times a weak	high	medium	easy	-0.72867963
64	2	1	other	0.5	subcutances other days	high	medium	hard	-0.643869086
64	2	0	iran	5.5	subcutances other days	medium	medium	hard	-0.928090082
64	3	0	iran	5.5	mascular once a weak	high	medium	hard	-0.572088286
64	3	1	iran	2.5	subcutances three times a weak	high	medium	easy	-0.646894171
64	4	0	iran	0.5	subcutances three times a weak	medium	low	hard	-0.699689278
64	4	1	other	2.5	mascular once a weak	high	low	hard	0.00383736
64	5	0	iran	0.5	mascular once a weak	medium	low	easy	-0.303092094
64	5	1	other	2.5	subcutances three times a weak	high	low	easy	-0.030902146
64	6	1	other	2.5	subcutances three times a weak	high	low	easy	-0.030902146
64	6	0	other	0.5	subcutances other days	high	medium	easy	-0.462940247
64	7	0	other	0.5	mascular once a weak	medium	medium	hard	-0.900801637
64	7	1	other	5.5	subcutances other days	medium	low	hard	-0.312098058
64	8	0	iran	2.5	mascular once a weak	high	low	hard	-0.0957683
64	8	1	iran	5.5	subcutances three times a weak	high	low	easy	-0.090441429

64	9	0	iran	2.5	subcutances three times a weak	medium	low	hard	-0.617903819
64	9	1	other	0.5	subcutances other days	high	medium	hard	-0.643869086
64	10	1	other	2.5	mascular once a weak	high	low	hard	0.00383736
64	10	0	iran	5.5	mascular once a weak	medium	medium	easy	-0.697626621
64	11	1	other	5.5	subcutances other days	high	medium	easy	-0.34108841
64	11	0	iran	0.5	subcutances three times a weak	medium	low	hard	-0.699689278
64	12	1	iran	5.5	subcutances three times a weak	high	medium	hard	-0.787756632
64	12	0	other	2.5	subcutances other days	medium	low	hard	-0.352164436
65	1	0	iran	5.5	mascular once a weak	high	medium	hard	-0.572088286
65	1	1	iran	0.5	subcutances three times a weak	high	medium	easy	-0.72867963
65	2	0	other	0.5	subcutances other days	high	medium	hard	-0.643869086
65	2	1	iran	5.5	subcutances other days	medium	medium	hard	-0.928090082
65	3	0	iran	5.5	mascular once a weak	high	medium	hard	-0.572088286
65	3	1	iran	2.5	subcutances three times a weak	high	medium	easy	-0.646894171
65	4	0	iran	0.5	subcutances three times a weak	medium	low	hard	-0.699689278
65	4	1	other	2.5	mascular once a weak	high	low	hard	0.00383736
65	5	1	iran	0.5	mascular once a weak	medium	low	easy	-0.303092094
65	5	0	other	2.5	subcutances three times a weak	high	low	easy	-0.030902146
65	6	1	other	2.5	subcutances three times a weak	high	low	easy	-0.030902146
65	6	0	other	0.5	subcutances other days	high	medium	easy	-0.462940247
65	7	0	other	0.5	mascular once a weak	medium	medium	hard	-0.900801637
65	7	1	other	5.5	subcutances other days	medium	low	hard	-0.312098058
65	8	0	iran	2.5	mascular once a weak	high	low	hard	-0.0957683
65	8	1	iran	5.5	subcutances three times a weak	high	low	easy	-0.090441429
65	9	1	iran	2.5	subcutances three times a weak	medium	low	hard	-0.617903819
65	9	0	other	0.5	subcutances other days	high	medium	hard	-0.643869086
65	10	1	other	2.5	mascular once a weak	high	low	hard	0.00383736
65	10	0	iran	5.5	mascular once a weak	medium	medium	easy	-0.697626621
65	11	0	other	5.5	subcutances other days	high	medium	easy	-0.34108841
65	11	1	iran	0.5	subcutances three times a weak	medium	low	hard	-0.699689278
65	12	1	iran	5.5	subcutances three times a weak	high	medium	hard	-0.787756632
65	12	0	other	2.5	subcutances other days	medium	low	hard	-0.352164436
66	1	1	iran	5.5	mascular once a weak	high	medium	hard	-0.572088286
66	1	0	iran	0.5	subcutances three times a weak	high	medium	easy	-0.72867963

66	2	1	other	0.5	subcutances	other days	high	medium	hard	-0.643869086
66	2	0	iran	5.5	subcutances	other days	medium	medium	hard	-0.928090082
66	3	1	iran	5.5	mascular	once a weak	high	medium	hard	-0.572088286
66	3	0	iran	2.5	subcutances	three times a weak	high	medium	easy	-0.646894171
66	4	0	iran	0.5	subcutances	three times a weak	medium	low	hard	-0.699689278
66	4	1	other	2.5	mascular	once a weak	high	low	hard	0.00383736
66	5	0	iran	0.5	mascular	once a weak	medium	low	easy	-0.303092094
66	5	1	other	2.5	subcutances	three times a weak	high	low	easy	-0.030902146
66	6	1	other	2.5	subcutances	three times a weak	high	low	easy	-0.030902146
66	6	0	other	0.5	subcutances	other days	high	medium	easy	-0.462940247
66	7	0	other	0.5	mascular	once a weak	medium	medium	hard	-0.900801637
66	7	1	other	5.5	subcutances	other days	medium	low	hard	-0.312098058
66	8	1	iran	2.5	mascular	once a weak	high	low	hard	-0.0957683
66	8	0	iran	5.5	subcutances	three times a weak	high	low	easy	-0.090441429
66	9	0	iran	2.5	subcutances	three times a weak	medium	low	hard	-0.617903819
66	9	1	other	0.5	subcutances	other days	high	medium	hard	-0.643869086
66	10	1	other	2.5	mascular	once a weak	high	low	hard	0.00383736
66	10	0	iran	5.5	mascular	once a weak	medium	medium	easy	-0.697626621
66	11	1	other	5.5	subcutances	other days	high	medium	easy	-0.34108841
66	11	0	iran	0.5	subcutances	three times a weak	medium	low	hard	-0.699689278
66	12	0	iran	5.5	subcutances	three times a weak	high	medium	hard	-0.787756632
66	12	1	other	2.5	subcutances	other days	medium	low	hard	-0.352164436
67	1	1	iran	5.5	mascular	once a weak	high	medium	hard	-0.572088286
67	1	0	iran	0.5	subcutances	three times a weak	high	medium	easy	-0.72867963
67	2	1	other	0.5	subcutances	other days	high	medium	hard	-0.643869086
67	2	0	iran	5.5	subcutances	other days	medium	medium	hard	-0.928090082
67	3	0	iran	5.5	mascular	once a weak	high	medium	hard	-0.572088286
67	3	1	iran	2.5	subcutances	three times a weak	high	medium	easy	-0.646894171
67	4	0	iran	0.5	subcutances	three times a weak	medium	low	hard	-0.699689278
67	4	1	other	2.5	mascular	once a weak	high	low	hard	0.00383736
67	5	0	iran	0.5	mascular	once a weak	medium	low	easy	-0.303092094
67	5	1	other	2.5	subcutances	three times a weak	high	low	easy	-0.030902146
67	6	0	other	2.5	subcutances	three times a weak	high	low	easy	-0.030902146
67	6	1	other	0.5	subcutances	other days	high	medium	easy	-0.462940247

67	7	0	other	0.5	mascular once a weak	medium	medium	hard	-0.900801637
67	7	1	other	5.5	subcutances other days	medium	low	hard	-0.312098058
67	8	1	iran	2.5	mascular once a weak	high	low	hard	-0.0957683
67	8	0	iran	5.5	subcutances three times a weak	high	low	easy	-0.090441429
67	9	0	iran	2.5	subcutances three times a weak	medium	low	hard	-0.617903819
67	9	1	other	0.5	subcutances other days	high	medium	hard	-0.643869086
67	10	1	other	2.5	mascular once a weak	high	low	hard	0.00383736
67	10	0	iran	5.5	mascular once a weak	medium	medium	easy	-0.697626621
67	11	1	other	5.5	subcutances other days	high	medium	easy	-0.34108841
67	11	0	iran	0.5	subcutances three times a weak	medium	low	hard	-0.699689278
67	12	1	iran	5.5	subcutances three times a weak	high	medium	hard	-0.787756632
67	12	0	other	2.5	subcutances other days	medium	low	hard	-0.352164436
68	1	1	iran	5.5	mascular once a weak	high	medium	hard	-0.572088286
68	1	0	iran	0.5	subcutances three times a weak	high	medium	easy	-0.72867963
68	2	1	other	0.5	subcutances other days	high	medium	hard	-0.643869086
68	2	0	iran	5.5	subcutances other days	medium	medium	hard	-0.928090082
68	3	1	iran	5.5	mascular once a weak	high	medium	hard	-0.572088286
68	3	0	iran	2.5	subcutances three times a weak	high	medium	easy	-0.646894171
68	4	0	iran	0.5	subcutances three times a weak	medium	low	hard	-0.699689278
68	4	1	other	2.5	mascular once a weak	high	low	hard	0.00383736
68	5	1	iran	0.5	mascular once a weak	medium	low	easy	-0.303092094
68	5	0	other	2.5	subcutances three times a weak	high	low	easy	-0.030902146
68	6	1	other	2.5	subcutances three times a weak	high	low	easy	-0.030902146
68	6	0	other	0.5	subcutances other days	high	medium	easy	-0.462940247
68	7	1	other	0.5	mascular once a weak	medium	medium	hard	-0.900801637
68	7	0	other	5.5	subcutances other days	medium	low	hard	-0.312098058
68	8	1	iran	2.5	mascular once a weak	high	low	hard	-0.0957683
68	8	0	iran	5.5	subcutances three times a weak	high	low	easy	-0.090441429
68	9	0	iran	2.5	subcutances three times a weak	medium	low	hard	-0.617903819
68	9	1	other	0.5	subcutances other days	high	medium	hard	-0.643869086
68	10	1	other	2.5	mascular once a weak	high	low	hard	0.00383736
68	10	0	iran	5.5	mascular once a weak	medium	medium	easy	-0.697626621
68	11	1	other	5.5	subcutances other days	high	medium	easy	-0.34108841
68	11	0	iran	0.5	subcutances three times a weak	medium	low	hard	-0.699689278

68	12	0	iran	5.5	subcutances	three times a weak	high	medium	hard	-0.787756632
68	12	1	other	2.5	subcutances	other days	medium	low	hard	-0.352164436
69	1	1	iran	5.5	mascular	once a weak	high	medium	hard	-0.572088286
69	1	0	iran	0.5	subcutances	three times a weak	high	medium	easy	-0.72867963
69	2	1	other	0.5	subcutances	other days	high	medium	hard	-0.643869086
69	2	0	iran	5.5	subcutances	other days	medium	medium	hard	-0.928090082
69	3	1	iran	5.5	mascular	once a weak	high	medium	hard	-0.572088286
69	3	0	iran	2.5	subcutances	three times a weak	high	medium	easy	-0.646894171
69	4	0	iran	0.5	subcutances	three times a weak	medium	low	hard	-0.699689278
69	4	1	other	2.5	mascular	once a weak	high	low	hard	0.00383736
69	5	0	iran	0.5	mascular	once a weak	medium	low	easy	-0.303092094
69	5	1	other	2.5	subcutances	three times a weak	high	low	easy	-0.030902146
69	6	1	other	2.5	subcutances	three times a weak	high	low	easy	-0.030902146
69	6	0	other	0.5	subcutances	other days	high	medium	easy	-0.462940247
69	7	1	other	0.5	mascular	once a weak	medium	medium	hard	-0.900801637
69	7	0	other	5.5	subcutances	other days	medium	low	hard	-0.312098058
69	8	1	iran	2.5	mascular	once a weak	high	low	hard	-0.0957683
69	8	0	iran	5.5	subcutances	three times a weak	high	low	easy	-0.090441429
69	9	0	iran	2.5	subcutances	three times a weak	medium	low	hard	-0.617903819
69	9	1	other	0.5	subcutances	other days	high	medium	hard	-0.643869086
69	10	1	other	2.5	mascular	once a weak	high	low	hard	0.00383736
69	10	0	iran	5.5	mascular	once a weak	medium	medium	easy	-0.697626621
69	11	1	other	5.5	subcutances	other days	high	medium	easy	-0.34108841
69	11	0	iran	0.5	subcutances	three times a weak	medium	low	hard	-0.699689278
69	12	0	iran	5.5	subcutances	three times a weak	high	medium	hard	-0.787756632
69	12	1	other	2.5	subcutances	other days	medium	low	hard	-0.352164436
70	1	1	iran	5.5	mascular	once a weak	high	medium	hard	-0.572088286
70	1	0	iran	0.5	subcutances	three times a weak	high	medium	easy	-0.72867963
70	2	0	other	0.5	subcutances	other days	high	medium	hard	-0.643869086
70	2	1	iran	5.5	subcutances	other days	medium	medium	hard	-0.928090082
70	3	1	iran	5.5	mascular	once a weak	high	medium	hard	-0.572088286
70	3	0	iran	2.5	subcutances	three times a weak	high	medium	easy	-0.646894171
70	4	0	iran	0.5	subcutances	three times a weak	medium	low	hard	-0.699689278
70	4	1	other	2.5	mascular	once a weak	high	low	hard	0.00383736

70	5	1	iran	0.5	mascular once a weak	medium	low	easy	-0.303092094
70	5	0	other	2.5	subcutances three times a weak	high	low	easy	-0.030902146
70	6	1	other	2.5	subcutances three times a weak	high	low	easy	-0.030902146
70	6	0	other	0.5	subcutances other days	high	medium	easy	-0.462940247
70	7	1	other	0.5	mascular once a weak	medium	medium	hard	-0.900801637
70	7	0	other	5.5	subcutances other days	medium	low	hard	-0.312098058
70	8	1	iran	2.5	mascular once a weak	high	low	hard	-0.0957683
70	8	0	iran	5.5	subcutances three times a weak	high	low	easy	-0.090441429
70	9	1	iran	2.5	subcutances three times a weak	medium	low	hard	-0.617903819
70	9	0	other	0.5	subcutances other days	high	medium	hard	-0.643869086
70	10	1	other	2.5	mascular once a weak	high	low	hard	0.00383736
70	10	0	iran	5.5	mascular once a weak	medium	medium	easy	-0.697626621
70	11	0	other	5.5	subcutances other days	high	medium	easy	-0.34108841
70	11	1	iran	0.5	subcutances three times a weak	medium	low	hard	-0.699689278
70	12	1	iran	5.5	subcutances three times a weak	high	medium	hard	-0.787756632
70	12	0	other	2.5	subcutances other days	medium	low	hard	-0.352164436
71	1	0	iran	5.5	mascular once a weak	high	medium	hard	-0.572088286
71	1	1	iran	0.5	subcutances three times a weak	high	medium	easy	-0.72867963
71	2	1	other	0.5	subcutances other days	high	medium	hard	-0.643869086
71	2	0	iran	5.5	subcutances other days	medium	medium	hard	-0.928090082
71	3	0	iran	5.5	mascular once a weak	high	medium	hard	-0.572088286
71	3	1	iran	2.5	subcutances three times a weak	high	medium	easy	-0.646894171
71	4	0	iran	0.5	subcutances three times a weak	medium	low	hard	-0.699689278
71	4	1	other	2.5	mascular once a weak	high	low	hard	0.00383736
71	5	1	iran	0.5	mascular once a weak	medium	low	easy	-0.303092094
71	5	0	other	2.5	subcutances three times a weak	high	low	easy	-0.030902146
71	6	0	other	2.5	subcutances three times a weak	high	low	easy	-0.030902146
71	6	1	other	0.5	subcutances other days	high	medium	easy	-0.462940247
71	7	1	other	0.5	mascular once a weak	medium	medium	hard	-0.900801637
71	7	0	other	5.5	subcutances other days	medium	low	hard	-0.312098058
71	8	0	iran	2.5	mascular once a weak	high	low	hard	-0.0957683
71	8	1	iran	5.5	subcutances three times a weak	high	low	easy	-0.090441429
71	9	1	iran	2.5	subcutances three times a weak	medium	low	hard	-0.617903819
71	9	0	other	0.5	subcutances other days	high	medium	hard	-0.643869086

71	10	0	other	2.5	mascular once a weak	high	low	hard	0.00383736
71	10	1	iran	5.5	mascular once a weak	medium	medium	easy	-0.697626621
71	11	1	other	5.5	subcutances other days	high	medium	easy	-0.34108841
71	11	0	iran	0.5	subcutances three times a weak	medium	low	hard	-0.699689278
71	12	1	iran	5.5	subcutances three times a weak	high	medium	hard	-0.787756632
71	12	0	other	2.5	subcutances other days	medium	low	hard	-0.352164436
72	1	0	iran	5.5	mascular once a weak	high	medium	hard	-0.572088286
72	1	1	iran	0.5	subcutances three times a weak	high	medium	easy	-0.72867963
72	2	1	other	0.5	subcutances other days	high	medium	hard	-0.643869086
72	2	0	iran	5.5	subcutances other days	medium	medium	hard	-0.928090082
72	3	0	iran	5.5	mascular once a weak	high	medium	hard	-0.572088286
72	3	1	iran	2.5	subcutances three times a weak	high	medium	easy	-0.646894171
72	4	0	iran	0.5	subcutances three times a weak	medium	low	hard	-0.699689278
72	4	1	other	2.5	mascular once a weak	high	low	hard	0.00383736
72	5	0	iran	0.5	mascular once a weak	medium	low	easy	-0.303092094
72	5	1	other	2.5	subcutances three times a weak	high	low	easy	-0.030902146
72	6	1	other	2.5	subcutances three times a weak	high	low	easy	-0.030902146
72	6	0	other	0.5	subcutances other days	high	medium	easy	-0.462940247
72	7	0	other	0.5	mascular once a weak	medium	medium	hard	-0.900801637
72	7	1	other	5.5	subcutances other days	medium	low	hard	-0.312098058
72	8	0	iran	2.5	mascular once a weak	high	low	hard	-0.0957683
72	8	1	iran	5.5	subcutances three times a weak	high	low	easy	-0.090441429
72	9	0	iran	2.5	subcutances three times a weak	medium	low	hard	-0.617903819
72	9	1	other	0.5	subcutances other days	high	medium	hard	-0.643869086
72	10	1	other	2.5	mascular once a weak	high	low	hard	0.00383736
72	10	0	iran	5.5	mascular once a weak	medium	medium	easy	-0.697626621
72	11	1	other	5.5	subcutances other days	high	medium	easy	-0.34108841
72	11	0	iran	0.5	subcutances three times a weak	medium	low	hard	-0.699689278
72	12	1	iran	5.5	subcutances three times a weak	high	medium	hard	-0.787756632
72	12	0	other	2.5	subcutances other days	medium	low	hard	-0.352164436
73	1	1	iran	5.5	mascular once a weak	high	medium	hard	-0.572088286
73	1	0	iran	0.5	subcutances three times a weak	high	medium	easy	-0.72867963
73	2	0	other	0.5	subcutances other days	high	medium	hard	-0.643869086
73	2	1	iran	5.5	subcutances other days	medium	medium	hard	-0.928090082

73	3	0	iran	5.5	mascular once a weak	high	medium	hard	-0.572088286
73	3	1	iran	2.5	subcutances three times a weak	high	medium	easy	-0.646894171
73	4	1	iran	0.5	subcutances three times a weak	medium	low	hard	-0.699689278
73	4	0	other	2.5	mascular once a weak	high	low	hard	0.00383736
73	5	1	iran	0.5	mascular once a weak	medium	low	easy	-0.303092094
73	5	0	other	2.5	subcutances three times a weak	high	low	easy	-0.030902146
73	6	0	other	2.5	subcutances three times a weak	high	low	easy	-0.030902146
73	6	1	other	0.5	subcutances other days	high	medium	easy	-0.462940247
73	7	1	other	0.5	mascular once a weak	medium	medium	hard	-0.900801637
73	7	0	other	5.5	subcutances other days	medium	low	hard	-0.312098058
73	8	1	iran	2.5	mascular once a weak	high	low	hard	-0.0957683
73	8	0	iran	5.5	subcutances three times a weak	high	low	easy	-0.090441429
73	9	1	iran	2.5	subcutances three times a weak	medium	low	hard	-0.617903819
73	9	0	other	0.5	subcutances other days	high	medium	hard	-0.643869086
73	10	0	other	2.5	mascular once a weak	high	low	hard	0.00383736
73	10	1	iran	5.5	mascular once a weak	medium	medium	easy	-0.697626621
73	11	0	other	5.5	subcutances other days	high	medium	easy	-0.34108841
73	11	1	iran	0.5	subcutances three times a weak	medium	low	hard	-0.699689278
73	12	0	iran	5.5	subcutances three times a weak	high	medium	hard	-0.787756632
73	12	1	other	2.5	subcutances other days	medium	low	hard	-0.352164436
74	1	1	iran	5.5	mascular once a weak	high	medium	hard	-0.572088286
74	1	0	iran	0.5	subcutances three times a weak	high	medium	easy	-0.72867963
74	2	1	other	0.5	subcutances other days	high	medium	hard	-0.643869086
74	2	0	iran	5.5	subcutances other days	medium	medium	hard	-0.928090082
74	3	1	iran	5.5	mascular once a weak	high	medium	hard	-0.572088286
74	3	0	iran	2.5	subcutances three times a weak	high	medium	easy	-0.646894171
74	4	0	iran	0.5	subcutances three times a weak	medium	low	hard	-0.699689278
74	4	1	other	2.5	mascular once a weak	high	low	hard	0.00383736
74	5	0	iran	0.5	mascular once a weak	medium	low	easy	-0.303092094
74	5	1	other	2.5	subcutances three times a weak	high	low	easy	-0.030902146
74	6	0	other	2.5	subcutances three times a weak	high	low	easy	-0.030902146
74	6	1	other	0.5	subcutances other days	high	medium	easy	-0.462940247
74	7	1	other	0.5	mascular once a weak	medium	medium	hard	-0.900801637
74	7	0	other	5.5	subcutances other days	medium	low	hard	-0.312098058

74	8	1	iran	2.5	mascular once a weak	high	low	hard	-0.0957683
74	8	0	iran	5.5	subcutances three times a weak	high	low	easy	-0.090441429
74	9	1	iran	2.5	subcutances three times a weak	medium	low	hard	-0.617903819
74	9	0	other	0.5	subcutances other days	high	medium	hard	-0.643869086
74	10	1	other	2.5	mascular once a weak	high	low	hard	0.00383736
74	10	0	iran	5.5	mascular once a weak	medium	medium	easy	-0.697626621
74	11	0	other	5.5	subcutances other days	high	medium	easy	-0.34108841
74	11	1	iran	0.5	subcutances three times a weak	medium	low	hard	-0.699689278
74	12	0	iran	5.5	subcutances three times a weak	high	medium	hard	-0.787756632
74	12	1	other	2.5	subcutances other days	medium	low	hard	-0.352164436
75	1	0	iran	5.5	mascular once a weak	high	medium	hard	-0.572088286
75	1	1	iran	0.5	subcutances three times a weak	high	medium	easy	-0.72867963
75	2	1	other	0.5	subcutances other days	high	medium	hard	-0.643869086
75	2	0	iran	5.5	subcutances other days	medium	medium	hard	-0.928090082
75	3	0	iran	5.5	mascular once a weak	high	medium	hard	-0.572088286
75	3	1	iran	2.5	subcutances three times a weak	high	medium	easy	-0.646894171
75	4	0	iran	0.5	subcutances three times a weak	medium	low	hard	-0.699689278
75	4	1	other	2.5	mascular once a weak	high	low	hard	0.00383736
75	5	0	iran	0.5	mascular once a weak	medium	low	easy	-0.303092094
75	5	1	other	2.5	subcutances three times a weak	high	low	easy	-0.030902146
75	6	0	other	2.5	subcutances three times a weak	high	low	easy	-0.030902146
75	6	1	other	0.5	subcutances other days	high	medium	easy	-0.462940247
75	7	0	other	0.5	mascular once a weak	medium	medium	hard	-0.900801637
75	7	1	other	5.5	subcutances other days	medium	low	hard	-0.312098058
75	8	0	iran	2.5	mascular once a weak	high	low	hard	-0.0957683
75	8	1	iran	5.5	subcutances three times a weak	high	low	easy	-0.090441429
75	9	0	iran	2.5	subcutances three times a weak	medium	low	hard	-0.617903819
75	9	1	other	0.5	subcutances other days	high	medium	hard	-0.643869086
75	10	0	other	2.5	mascular once a weak	high	low	hard	0.00383736
75	10	1	iran	5.5	mascular once a weak	medium	medium	easy	-0.697626621
75	11	1	other	5.5	subcutances other days	high	medium	easy	-0.34108841
75	11	0	iran	0.5	subcutances three times a weak	medium	low	hard	-0.699689278
75	12	1	iran	5.5	subcutances three times a weak	high	medium	hard	-0.787756632
75	12	0	other	2.5	subcutances other days	medium	low	hard	-0.352164436

76	1	0	iran	5.5	mascular once a weak	high	medium	hard	-0.572088286
76	1	1	iran	0.5	subcutances three times a weak	high	medium	easy	-0.72867963
76	2	1	other	0.5	subcutances other days	high	medium	hard	-0.643869086
76	2	0	iran	5.5	subcutances other days	medium	medium	hard	-0.928090082
76	3	0	iran	5.5	mascular once a weak	high	medium	hard	-0.572088286
76	3	1	iran	2.5	subcutances three times a weak	high	medium	easy	-0.646894171
76	4	0	iran	0.5	subcutances three times a weak	medium	low	hard	-0.699689278
76	4	1	other	2.5	mascular once a weak	high	low	hard	0.00383736
76	5	0	iran	0.5	mascular once a weak	medium	low	easy	-0.303092094
76	5	1	other	2.5	subcutances three times a weak	high	low	easy	-0.030902146
76	6	0	other	2.5	subcutances three times a weak	high	low	easy	-0.030902146
76	6	1	other	0.5	subcutances other days	high	medium	easy	-0.462940247
76	7	0	other	0.5	mascular once a weak	medium	medium	hard	-0.900801637
76	7	1	other	5.5	subcutances other days	medium	low	hard	-0.312098058
76	8	0	iran	2.5	mascular once a weak	high	low	hard	-0.0957683
76	8	1	iran	5.5	subcutances three times a weak	high	low	easy	-0.090441429
76	9	0	iran	2.5	subcutances three times a weak	medium	low	hard	-0.617903819
76	9	1	other	0.5	subcutances other days	high	medium	hard	-0.643869086
76	10	1	other	2.5	mascular once a weak	high	low	hard	0.00383736
76	10	0	iran	5.5	mascular once a weak	medium	medium	easy	-0.697626621
76	11	1	other	5.5	subcutances other days	high	medium	easy	-0.34108841
76	11	0	iran	0.5	subcutances three times a weak	medium	low	hard	-0.699689278
76	12	1	iran	5.5	subcutances three times a weak	high	medium	hard	-0.787756632
76	12	0	other	2.5	subcutances other days	medium	low	hard	-0.352164436
77	1	0	iran	5.5	mascular once a weak	high	medium	hard	-0.572088286
77	1	1	iran	0.5	subcutances three times a weak	high	medium	easy	-0.72867963
77	2	0	other	0.5	subcutances other days	high	medium	hard	-0.643869086
77	2	1	iran	5.5	subcutances other days	medium	medium	hard	-0.928090082
77	3	0	iran	5.5	mascular once a weak	high	medium	hard	-0.572088286
77	3	1	iran	2.5	subcutances three times a weak	high	medium	easy	-0.646894171
77	4	1	iran	0.5	subcutances three times a weak	medium	low	hard	-0.699689278
77	4	0	other	2.5	mascular once a weak	high	low	hard	0.00383736
77	5	0	iran	0.5	mascular once a weak	medium	low	easy	-0.303092094
77	5	1	other	2.5	subcutances three times a weak	high	low	easy	-0.030902146

77	6	0	other	2.5	subcutances three times a weak	high	low	easy	-0.030902146
77	6	1	other	0.5	subcutances other days	high	medium	easy	-0.462940247
77	7	0	other	0.5	mascular once a weak	medium	medium	hard	-0.900801637
77	7	1	other	5.5	subcutances other days	medium	low	hard	-0.312098058
77	8	0	iran	2.5	mascular once a weak	high	low	hard	-0.0957683
77	8	1	iran	5.5	subcutances three times a weak	high	low	easy	-0.090441429
77	9	1	iran	2.5	subcutances three times a weak	medium	low	hard	-0.617903819
77	9	0	other	0.5	subcutances other days	high	medium	hard	-0.643869086
77	10	1	other	2.5	mascular once a weak	high	low	hard	0.00383736
77	10	0	iran	5.5	mascular once a weak	medium	medium	easy	-0.697626621
77	11	0	other	5.5	subcutances other days	high	medium	easy	-0.34108841
77	11	1	iran	0.5	subcutances three times a weak	medium	low	hard	-0.699689278
77	12	1	iran	5.5	subcutances three times a weak	high	medium	hard	-0.787756632
77	12	0	other	2.5	subcutances other days	medium	low	hard	-0.352164436
78	1	1	iran	5.5	mascular once a weak	high	medium	hard	-0.572088286
78	1	0	iran	0.5	subcutances three times a weak	high	medium	easy	-0.72867963
78	2	1	other	0.5	subcutances other days	high	medium	hard	-0.643869086
78	2	0	iran	5.5	subcutances other days	medium	medium	hard	-0.928090082
78	3	1	iran	5.5	mascular once a weak	high	medium	hard	-0.572088286
78	3	0	iran	2.5	subcutances three times a weak	high	medium	easy	-0.646894171
78	4	0	iran	0.5	subcutances three times a weak	medium	low	hard	-0.699689278
78	4	1	other	2.5	mascular once a weak	high	low	hard	0.00383736
78	5	0	iran	0.5	mascular once a weak	medium	low	easy	-0.303092094
78	5	1	other	2.5	subcutances three times a weak	high	low	easy	-0.030902146
78	6	1	other	2.5	subcutances three times a weak	high	low	easy	-0.030902146
78	6	0	other	0.5	subcutances other days	high	medium	easy	-0.462940247
78	7	0	other	0.5	mascular once a weak	medium	medium	hard	-0.900801637
78	7	1	other	5.5	subcutances other days	medium	low	hard	-0.312098058
78	8	0	iran	2.5	mascular once a weak	high	low	hard	-0.0957683
78	8	1	iran	5.5	subcutances three times a weak	high	low	easy	-0.090441429
78	9	0	iran	2.5	subcutances three times a weak	medium	low	hard	-0.617903819
78	9	1	other	0.5	subcutances other days	high	medium	hard	-0.643869086
78	10	1	other	2.5	mascular once a weak	high	low	hard	0.00383736
78	10	0	iran	5.5	mascular once a weak	medium	medium	easy	-0.697626621

78	11	1	other	5.5	subcutances other days	high	medium	easy	-0.34108841
78	11	0	iran	0.5	subcutances three times a weak	medium	low	hard	-0.699689278
78	12	0	iran	5.5	subcutances three times a weak	high	medium	hard	-0.787756632
78	12	1	other	2.5	subcutances other days	medium	low	hard	-0.352164436
79	1	0	iran	5.5	mascular once a weak	high	medium	hard	-0.572088286
79	1	1	iran	0.5	subcutances three times a weak	high	medium	easy	-0.72867963
79	2	1	other	0.5	subcutances other days	high	medium	hard	-0.643869086
79	2	0	iran	5.5	subcutances other days	medium	medium	hard	-0.928090082
79	3	0	iran	5.5	mascular once a weak	high	medium	hard	-0.572088286
79	3	1	iran	2.5	subcutances three times a weak	high	medium	easy	-0.646894171
79	4	1	iran	0.5	subcutances three times a weak	medium	low	hard	-0.699689278
79	4	0	other	2.5	mascular once a weak	high	low	hard	0.00383736
79	5	0	iran	0.5	mascular once a weak	medium	low	easy	-0.303092094
79	5	1	other	2.5	subcutances three times a weak	high	low	easy	-0.030902146
79	6	1	other	2.5	subcutances three times a weak	high	low	easy	-0.030902146
79	6	0	other	0.5	subcutances other days	high	medium	easy	-0.462940247
79	7	0	other	0.5	mascular once a weak	medium	medium	hard	-0.900801637
79	7	1	other	5.5	subcutances other days	medium	low	hard	-0.312098058
79	8	0	iran	2.5	mascular once a weak	high	low	hard	-0.0957683
79	8	1	iran	5.5	subcutances three times a weak	high	low	easy	-0.090441429
79	9	1	iran	2.5	subcutances three times a weak	medium	low	hard	-0.617903819
79	9	0	other	0.5	subcutances other days	high	medium	hard	-0.643869086
79	10	1	other	2.5	mascular once a weak	high	low	hard	0.00383736
79	10	0	iran	5.5	mascular once a weak	medium	medium	easy	-0.697626621
79	11	1	other	5.5	subcutances other days	high	medium	easy	-0.34108841
79	11	0	iran	0.5	subcutances three times a weak	medium	low	hard	-0.699689278
79	12	1	iran	5.5	subcutances three times a weak	high	medium	hard	-0.787756632
79	12	0	other	2.5	subcutances other days	medium	low	hard	-0.352164436
80	1	0	iran	5.5	mascular once a weak	high	medium	hard	-0.572088286
80	1	1	iran	0.5	subcutances three times a weak	high	medium	easy	-0.72867963
80	2	1	other	0.5	subcutances other days	high	medium	hard	-0.643869086
80	2	0	iran	5.5	subcutances other days	medium	medium	hard	-0.928090082
80	3	0	iran	5.5	mascular once a weak	high	medium	hard	-0.572088286
80	3	0	iran	2.5	subcutances three times a weak	high	medium	easy	-0.646894171

80	4	0	iran	0.5	subcutances three times a weak	medium	low	hard	-0.699689278
80	4	1	other	2.5	mascular once a weak	high	low	hard	0.00383736
80	5	0	iran	0.5	mascular once a weak	medium	low	easy	-0.303092094
80	5	1	other	2.5	subcutances three times a weak	high	low	easy	-0.030902146
80	6	1	other	2.5	subcutances three times a weak	high	low	easy	-0.030902146
80	6	0	other	0.5	subcutances other days	high	medium	easy	-0.462940247
80	7	1	other	0.5	mascular once a weak	medium	medium	hard	-0.900801637
80	7	0	other	5.5	subcutances other days	medium	low	hard	-0.312098058
80	8	1	iran	2.5	mascular once a weak	high	low	hard	-0.0957683
80	8	0	iran	5.5	subcutances three times a weak	high	low	easy	-0.090441429
80	9	0	iran	2.5	subcutances three times a weak	medium	low	hard	-0.617903819
80	9	1	other	0.5	subcutances other days	high	medium	hard	-0.643869086
80	10	1	other	2.5	mascular once a weak	high	low	hard	0.00383736
80	10	0	iran	5.5	mascular once a weak	medium	medium	easy	-0.697626621
80	11	1	other	5.5	subcutances other days	high	medium	easy	-0.34108841
80	11	0	iran	0.5	subcutances three times a weak	medium	low	hard	-0.699689278
80	12	0	iran	5.5	subcutances three times a weak	high	medium	hard	-0.787756632
80	12	1	other	2.5	subcutances other days	medium	low	hard	-0.352164436
81	1	1	iran	5.5	mascular once a weak	high	medium	hard	-0.572088286
81	1	0	iran	0.5	subcutances three times a weak	high	medium	easy	-0.72867963
81	2	0	other	0.5	subcutances other days	high	medium	hard	-0.643869086
81	2	1	iran	5.5	subcutances other days	medium	medium	hard	-0.928090082
81	3	1	iran	5.5	mascular once a weak	high	medium	hard	-0.572088286
81	3	0	iran	2.5	subcutances three times a weak	high	medium	easy	-0.646894171
81	4	0	iran	0.5	subcutances three times a weak	medium	low	hard	-0.699689278
81	4	1	other	2.5	mascular once a weak	high	low	hard	0.00383736
81	5	1	iran	0.5	mascular once a weak	medium	low	easy	-0.303092094
81	5	0	other	2.5	subcutances three times a weak	high	low	easy	-0.030902146
81	6	0	other	2.5	subcutances three times a weak	high	low	easy	-0.030902146
81	6	1	other	0.5	subcutances other days	high	medium	easy	-0.462940247
81	7	1	other	0.5	mascular once a weak	medium	medium	hard	-0.900801637
81	7	0	other	5.5	subcutances other days	medium	low	hard	-0.312098058
81	8	1	iran	2.5	mascular once a weak	high	low	hard	-0.0957683
81	8	0	iran	5.5	subcutances three times a weak	high	low	easy	-0.090441429

81	9	0	iran	2.5	subcutances three times a weak	medium	low	hard	-0.617903819
81	9	1	other	0.5	subcutances other days	high	medium	hard	-0.643869086
81	10	1	other	2.5	mascular once a weak	high	low	hard	0.00383736
81	10	0	iran	5.5	mascular once a weak	medium	medium	easy	-0.697626621
81	11	0	other	5.5	subcutances other days	high	medium	easy	-0.34108841
81	11	1	iran	0.5	subcutances three times a weak	medium	low	hard	-0.699689278
81	12	1	iran	5.5	subcutances three times a weak	high	medium	hard	-0.787756632
81	12	0	other	2.5	subcutances other days	medium	low	hard	-0.352164436
82	1	1	iran	5.5	mascular once a weak	high	medium	hard	-0.572088286
82	1	0	iran	0.5	subcutances three times a weak	high	medium	easy	-0.72867963
82	2	1	other	0.5	subcutances other days	high	medium	hard	-0.643869086
82	2	0	iran	5.5	subcutances other days	medium	medium	hard	-0.928090082
82	3	1	iran	5.5	mascular once a weak	high	medium	hard	-0.572088286
82	3	0	iran	2.5	subcutances three times a weak	high	medium	easy	-0.646894171
82	4	0	iran	0.5	subcutances three times a weak	medium	low	hard	-0.699689278
82	4	1	other	2.5	mascular once a weak	high	low	hard	0.00383736
82	5	0	iran	0.5	mascular once a weak	medium	low	easy	-0.303092094
82	5	1	other	2.5	subcutances three times a weak	high	low	easy	-0.030902146
82	6	1	other	2.5	subcutances three times a weak	high	low	easy	-0.030902146
82	6	0	other	0.5	subcutances other days	high	medium	easy	-0.462940247
82	7	1	other	0.5	mascular once a weak	medium	medium	hard	-0.900801637
82	7	0	other	5.5	subcutances other days	medium	low	hard	-0.312098058
82	8	1	iran	2.5	mascular once a weak	high	low	hard	-0.0957683
82	8	0	iran	5.5	subcutances three times a weak	high	low	easy	-0.090441429
82	9	1	iran	2.5	subcutances three times a weak	medium	low	hard	-0.617903819
82	9	0	other	0.5	subcutances other days	high	medium	hard	-0.643869086
82	10	1	other	2.5	mascular once a weak	high	low	hard	0.00383736
82	10	0	iran	5.5	mascular once a weak	medium	medium	easy	-0.697626621
82	11	0	other	5.5	subcutances other days	high	medium	easy	-0.34108841
82	11	1	iran	0.5	subcutances three times a weak	medium	low	hard	-0.699689278
82	12	0	iran	5.5	subcutances three times a weak	high	medium	hard	-0.787756632
82	12	1	other	2.5	subcutances other days	medium	low	hard	-0.352164436
83	1	0	iran	5.5	mascular once a weak	high	medium	hard	-0.572088286
83	1	1	iran	0.5	subcutances three times a weak	high	medium	easy	-0.72867963

83	2	1	other	0.5	subcutances other days	high	medium	hard	-0.643869086
83	2	0	iran	5.5	subcutances other days	medium	medium	hard	-0.928090082
83	3	1	iran	5.5	mascular once a weak	high	medium	hard	-0.572088286
83	3	0	iran	2.5	subcutances three times a weak	high	medium	easy	-0.646894171
83	4	0	iran	0.5	subcutances three times a weak	medium	low	hard	-0.699689278
83	4	1	other	2.5	mascular once a weak	high	low	hard	0.00383736
83	5	0	iran	0.5	mascular once a weak	medium	low	easy	-0.303092094
83	5	1	other	2.5	subcutances three times a weak	high	low	easy	-0.030902146
83	6	1	other	2.5	subcutances three times a weak	high	low	easy	-0.030902146
83	6	0	other	0.5	subcutances other days	high	medium	easy	-0.462940247
83	7	0	other	0.5	mascular once a weak	medium	medium	hard	-0.900801637
83	7	1	other	5.5	subcutances other days	medium	low	hard	-0.312098058
83	8	1	iran	2.5	mascular once a weak	high	low	hard	-0.0957683
83	8	0	iran	5.5	subcutances three times a weak	high	low	easy	-0.090441429
83	9	0	iran	2.5	subcutances three times a weak	medium	low	hard	-0.617903819
83	9	1	other	0.5	subcutances other days	high	medium	hard	-0.643869086
83	10	1	other	2.5	mascular once a weak	high	low	hard	0.00383736
83	10	0	iran	5.5	mascular once a weak	medium	medium	easy	-0.697626621
83	11	1	other	5.5	subcutances other days	high	medium	easy	-0.34108841
83	11	0	iran	0.5	subcutances three times a weak	medium	low	hard	-0.699689278
83	12	1	iran	5.5	subcutances three times a weak	high	medium	hard	-0.787756632
83	12	0	other	2.5	subcutances other days	medium	low	hard	-0.352164436
84	1	1	iran	5.5	mascular once a weak	high	medium	hard	-0.572088286
84	1	0	iran	0.5	subcutances three times a weak	high	medium	easy	-0.72867963
84	2	0	other	0.5	subcutances other days	high	medium	hard	-0.643869086
84	2	1	iran	5.5	subcutances other days	medium	medium	hard	-0.928090082
84	3	0	iran	5.5	mascular once a weak	high	medium	hard	-0.572088286
84	3	1	iran	2.5	subcutances three times a weak	high	medium	easy	-0.646894171
84	4	1	iran	0.5	subcutances three times a weak	medium	low	hard	-0.699689278
84	4	0	other	2.5	mascular once a weak	high	low	hard	0.00383736
84	5	1	iran	0.5	mascular once a weak	medium	low	easy	-0.303092094
84	5	0	other	2.5	subcutances three times a weak	high	low	easy	-0.030902146
84	6	1	other	2.5	subcutances three times a weak	high	low	easy	-0.030902146
84	6	0	other	0.5	subcutances other days	high	medium	easy	-0.462940247

84	7	1	other	0.5	mascular once a weak	medium	medium	hard	-0.900801637
84	7	0	other	5.5	subcutances other days	medium	low	hard	-0.312098058
84	8	1	iran	2.5	mascular once a weak	high	low	hard	-0.0957683
84	8	0	iran	5.5	subcutances three times a weak	high	low	easy	-0.090441429
84	9	0	iran	2.5	subcutances three times a weak	medium	low	hard	-0.617903819
84	9	1	other	0.5	subcutances other days	high	medium	hard	-0.643869086
84	10	1	other	2.5	mascular once a weak	high	low	hard	0.00383736
84	10	0	iran	5.5	mascular once a weak	medium	medium	easy	-0.697626621
84	11	1	other	5.5	subcutances other days	high	medium	easy	-0.34108841
84	11	0	iran	0.5	subcutances three times a weak	medium	low	hard	-0.699689278
84	12	0	iran	5.5	subcutances three times a weak	high	medium	hard	-0.787756632
84	12	1	other	2.5	subcutances other days	medium	low	hard	-0.352164436
85	1	1	iran	5.5	mascular once a weak	high	medium	hard	-0.572088286
85	1	0	iran	0.5	subcutances three times a weak	high	medium	easy	-0.72867963
85	2	1	other	0.5	subcutances other days	high	medium	hard	-0.643869086
85	2	0	iran	5.5	subcutances other days	medium	medium	hard	-0.928090082
85	3	1	iran	5.5	mascular once a weak	high	medium	hard	-0.572088286
85	3	0	iran	2.5	subcutances three times a weak	high	medium	easy	-0.646894171
85	4	0	iran	0.5	subcutances three times a weak	medium	low	hard	-0.699689278
85	4	1	other	2.5	mascular once a weak	high	low	hard	0.00383736
85	5	1	iran	0.5	mascular once a weak	medium	low	easy	-0.303092094
85	5	0	other	2.5	subcutances three times a weak	high	low	easy	-0.030902146
85	6	1	other	2.5	subcutances three times a weak	high	low	easy	-0.030902146
85	6	0	other	0.5	subcutances other days	high	medium	easy	-0.462940247
85	7	0	other	0.5	mascular once a weak	medium	medium	hard	-0.900801637
85	7	1	other	5.5	subcutances other days	medium	low	hard	-0.312098058
85	8	1	iran	2.5	mascular once a weak	high	low	hard	-0.0957683
85	8	0	iran	5.5	subcutances three times a weak	high	low	easy	-0.090441429
85	9	0	iran	2.5	subcutances three times a weak	medium	low	hard	-0.617903819
85	9	1	other	0.5	subcutances other days	high	medium	hard	-0.643869086
85	10	1	other	2.5	mascular once a weak	high	low	hard	0.00383736
85	10	0	iran	5.5	mascular once a weak	medium	medium	easy	-0.697626621
85	11	1	other	5.5	subcutances other days	high	medium	easy	-0.34108841
85	11	0	iran	0.5	subcutances three times a weak	medium	low	hard	-0.699689278

85	12	0	iran	5.5	subcutances three times a weak	high	medium	hard	-0.787756632
85	12	1	other	2.5	subcutances other days	medium	low	hard	-0.352164436
86	1	1	iran	5.5	mascular once a weak	high	medium	hard	-0.572088286
86	1	0	iran	0.5	subcutances three times a weak	high	medium	easy	-0.72867963
86	2	0	other	0.5	subcutances other days	high	medium	hard	-0.643869086
86	2	1	iran	5.5	subcutances other days	medium	medium	hard	-0.928090082
86	3	0	iran	5.5	mascular once a weak	high	medium	hard	-0.572088286
86	3	1	iran	2.5	subcutances three times a weak	high	medium	easy	-0.646894171
86	4	1	iran	0.5	subcutances three times a weak	medium	low	hard	-0.699689278
86	4	0	other	2.5	mascular once a weak	high	low	hard	0.00383736
86	5	1	iran	0.5	mascular once a weak	medium	low	easy	-0.303092094
86	5	0	other	2.5	subcutances three times a weak	high	low	easy	-0.030902146
86	6	1	other	2.5	subcutances three times a weak	high	low	easy	-0.030902146
86	6	0	other	0.5	subcutances other days	high	medium	easy	-0.462940247
86	7	0	other	0.5	mascular once a weak	medium	medium	hard	-0.900801637
86	7	1	other	5.5	subcutances other days	medium	low	hard	-0.312098058
86	8	1	iran	2.5	mascular once a weak	high	low	hard	-0.0957683
86	8	0	iran	5.5	subcutances three times a weak	high	low	easy	-0.090441429
86	9	1	iran	2.5	subcutances three times a weak	medium	low	hard	-0.617903819
86	9	0	other	0.5	subcutances other days	high	medium	hard	-0.643869086
86	10	1	other	2.5	mascular once a weak	high	low	hard	0.00383736
86	10	0	iran	5.5	mascular once a weak	medium	medium	easy	-0.697626621
86	11	0	other	5.5	subcutances other days	high	medium	easy	-0.34108841
86	11	1	iran	0.5	subcutances three times a weak	medium	low	hard	-0.699689278
86	12	0	iran	5.5	subcutances three times a weak	high	medium	hard	-0.787756632
86	12	1	other	2.5	subcutances other days	medium	low	hard	-0.352164436
87	1	1	iran	5.5	mascular once a weak	high	medium	hard	-0.572088286
87	1	0	iran	0.5	subcutances three times a weak	high	medium	easy	-0.72867963
87	2	0	other	0.5	subcutances other days	high	medium	hard	-0.643869086
87	2	1	iran	5.5	subcutances other days	medium	medium	hard	-0.928090082
87	3	0	iran	5.5	mascular once a weak	high	medium	hard	-0.572088286
87	3	1	iran	2.5	subcutances three times a weak	high	medium	easy	-0.646894171
87	4	1	iran	0.5	subcutances three times a weak	medium	low	hard	-0.699689278
87	4	0	other	2.5	mascular once a weak	high	low	hard	0.00383736

87	5	1	iran	0.5	mascular once a weak	medium	low	easy	-0.303092094
87	5	0	other	2.5	subcutances three times a weak	high	low	easy	-0.030902146
87	6	1	other	2.5	subcutances three times a weak	high	low	easy	-0.030902146
87	6	0	other	0.5	subcutances other days	high	medium	easy	-0.462940247
87	7	1	other	0.5	mascular once a weak	medium	medium	hard	-0.900801637
87	7	0	other	5.5	subcutances other days	medium	low	hard	-0.312098058
87	8	1	iran	2.5	mascular once a weak	high	low	hard	-0.0957683
87	8	0	iran	5.5	subcutances three times a weak	high	low	easy	-0.090441429
87	9	1	iran	2.5	subcutances three times a weak	medium	low	hard	-0.617903819
87	9	0	other	0.5	subcutances other days	high	medium	hard	-0.643869086
87	10	0	other	2.5	mascular once a weak	high	low	hard	0.00383736
87	10	1	iran	5.5	mascular once a weak	medium	medium	easy	-0.697626621
87	11	0	other	5.5	subcutances other days	high	medium	easy	-0.34108841
87	11	1	iran	0.5	subcutances three times a weak	medium	low	hard	-0.699689278
87	12	1	iran	5.5	subcutances three times a weak	high	medium	hard	-0.787756632
87	12	0	other	2.5	subcutances other days	medium	low	hard	-0.352164436
88	1	0	iran	5.5	mascular once a weak	high	medium	hard	-0.572088286
88	1	1	iran	0.5	subcutances three times a weak	high	medium	easy	-0.72867963
88	2	0	other	0.5	subcutances other days	high	medium	hard	-0.643869086
88	2	1	iran	5.5	subcutances other days	medium	medium	hard	-0.928090082
88	3	0	iran	5.5	mascular once a weak	high	medium	hard	-0.572088286
88	3	1	iran	2.5	subcutances three times a weak	high	medium	easy	-0.646894171
88	4	1	iran	0.5	subcutances three times a weak	medium	low	hard	-0.699689278
88	4	0	other	2.5	mascular once a weak	high	low	hard	0.00383736
88	5	1	iran	0.5	mascular once a weak	medium	low	easy	-0.303092094
88	5	0	other	2.5	subcutances three times a weak	high	low	easy	-0.030902146
88	6	0	other	2.5	subcutances three times a weak	high	low	easy	-0.030902146
88	6	1	other	0.5	subcutances other days	high	medium	easy	-0.462940247
88	7	0	other	0.5	mascular once a weak	medium	medium	hard	-0.900801637
88	7	1	other	5.5	subcutances other days	medium	low	hard	-0.312098058
88	8	1	iran	2.5	mascular once a weak	high	low	hard	-0.0957683
88	8	0	iran	5.5	subcutances three times a weak	high	low	easy	-0.090441429
88	9	1	iran	2.5	subcutances three times a weak	medium	low	hard	-0.617903819
88	9	0	other	0.5	subcutances other days	high	medium	hard	-0.643869086

88	10	0	other	2.5	mascular once a weak	high	low	hard	0.00383736
88	10	1	iran	5.5	mascular once a weak	medium	medium	easy	-0.697626621
88	11	0	other	5.5	subcutances other days	high	medium	easy	-0.34108841
88	11	1	iran	0.5	subcutances three times a weak	medium	low	hard	-0.699689278
88	12	1	iran	5.5	subcutances three times a weak	high	medium	hard	-0.787756632
88	12	0	other	2.5	subcutances other days	medium	low	hard	-0.352164436
89	1	1	iran	5.5	mascular once a weak	high	medium	hard	-0.572088286
89	1	0	iran	0.5	subcutances three times a weak	high	medium	easy	-0.72867963
89	2	1	other	0.5	subcutances other days	high	medium	hard	-0.643869086
89	2	0	iran	5.5	subcutances other days	medium	medium	hard	-0.928090082
89	3	1	iran	5.5	mascular once a weak	high	medium	hard	-0.572088286
89	3	0	iran	2.5	subcutances three times a weak	high	medium	easy	-0.646894171
89	4	0	iran	0.5	subcutances three times a weak	medium	low	hard	-0.699689278
89	4	1	other	2.5	mascular once a weak	high	low	hard	0.00383736
89	5	1	iran	0.5	mascular once a weak	medium	low	easy	-0.303092094
89	5	0	other	2.5	subcutances three times a weak	high	low	easy	-0.030902146
89	6	0	other	2.5	subcutances three times a weak	high	low	easy	-0.030902146
89	6	0	other	0.5	subcutances other days	high	medium	easy	-0.462940247
89	7	1	other	0.5	mascular once a weak	medium	medium	hard	-0.900801637
89	7	0	other	5.5	subcutances other days	medium	low	hard	-0.312098058
89	8	1	iran	2.5	mascular once a weak	high	low	hard	-0.0957683
89	8	0	iran	5.5	subcutances three times a weak	high	low	easy	-0.090441429
89	9	0	iran	2.5	subcutances three times a weak	medium	low	hard	-0.617903819
89	9	0	other	0.5	subcutances other days	high	medium	hard	-0.643869086
89	10	1	other	2.5	mascular once a weak	high	low	hard	0.00383736
89	10	0	iran	5.5	mascular once a weak	medium	medium	easy	-0.697626621
89	11	0	other	5.5	subcutances other days	high	medium	easy	-0.34108841
89	11	0	iran	0.5	subcutances three times a weak	medium	low	hard	-0.699689278
89	12	0	iran	5.5	subcutances three times a weak	high	medium	hard	-0.787756632
89	12	0	other	2.5	subcutances other days	medium	low	hard	-0.352164436
90	1	1	iran	5.5	mascular once a weak	high	medium	hard	-0.572088286
90	1	0	iran	0.5	subcutances three times a weak	high	medium	easy	-0.72867963
90	2	0	other	0.5	subcutances other days	high	medium	hard	-0.643869086
90	2	1	iran	5.5	subcutances other days	medium	medium	hard	-0.928090082

90	3	0	iran	5.5	mascular once a weak	high	medium	hard	-0.572088286
90	3	1	iran	2.5	subcutances three times a weak	high	medium	easy	-0.646894171
90	4	0	iran	0.5	subcutances three times a weak	medium	low	hard	-0.699689278
90	4	1	other	2.5	mascular once a weak	high	low	hard	0.00383736
90	5	0	iran	0.5	mascular once a weak	medium	low	easy	-0.303092094
90	5	1	other	2.5	subcutances three times a weak	high	low	easy	-0.030902146
90	6	0	other	2.5	subcutances three times a weak	high	low	easy	-0.030902146
90	6	1	other	0.5	subcutances other days	high	medium	easy	-0.462940247
90	7	1	other	0.5	mascular once a weak	medium	medium	hard	-0.900801637
90	7	0	other	5.5	subcutances other days	medium	low	hard	-0.312098058
90	8	1	iran	2.5	mascular once a weak	high	low	hard	-0.0957683
90	8	0	iran	5.5	subcutances three times a weak	high	low	easy	-0.090441429
90	9	1	iran	2.5	subcutances three times a weak	medium	low	hard	-0.617903819
90	9	0	other	0.5	subcutances other days	high	medium	hard	-0.643869086
90	10	0	other	2.5	mascular once a weak	high	low	hard	0.00383736
90	10	1	iran	5.5	mascular once a weak	medium	medium	easy	-0.697626621
90	11	0	other	5.5	subcutances other days	high	medium	easy	-0.34108841
90	11	1	iran	0.5	subcutances three times a weak	medium	low	hard	-0.699689278
90	12	0	iran	5.5	subcutances three times a weak	high	medium	hard	-0.787756632
90	12	1	other	2.5	subcutances other days	medium	low	hard	-0.352164436
91	1	0	iran	5.5	mascular once a weak	high	medium	hard	-0.572088286
91	1	1	iran	0.5	subcutances three times a weak	high	medium	easy	-0.72867963
91	2	1	other	0.5	subcutances other days	high	medium	hard	-0.643869086
91	2	0	iran	5.5	subcutances other days	medium	medium	hard	-0.928090082
91	3	0	iran	5.5	mascular once a weak	high	medium	hard	-0.572088286
91	3	1	iran	2.5	subcutances three times a weak	high	medium	easy	-0.646894171
91	4	0	iran	0.5	subcutances three times a weak	medium	low	hard	-0.699689278
91	4	1	other	2.5	mascular once a weak	high	low	hard	0.00383736
91	5	0	iran	0.5	mascular once a weak	medium	low	easy	-0.303092094
91	5	1	other	2.5	subcutances three times a weak	high	low	easy	-0.030902146
91	6	1	other	2.5	subcutances three times a weak	high	low	easy	-0.030902146
91	6	0	other	0.5	subcutances other days	high	medium	easy	-0.462940247
91	7	0	other	0.5	mascular once a weak	medium	medium	hard	-0.900801637
91	7	1	other	5.5	subcutances other days	medium	low	hard	-0.312098058

91	8	0	iran	2.5	mascular once a weak	high	low	hard	-0.0957683
91	8	1	iran	5.5	subcutances three times a weak	high	low	easy	-0.090441429
91	9	0	iran	2.5	subcutances three times a weak	medium	low	hard	-0.617903819
91	9	1	other	0.5	subcutances other days	high	medium	hard	-0.643869086
91	10	1	other	2.5	mascular once a weak	high	low	hard	0.00383736
91	10	0	iran	5.5	mascular once a weak	medium	medium	easy	-0.697626621
91	11	1	other	5.5	subcutances other days	high	medium	easy	-0.34108841
91	11	0	iran	0.5	subcutances three times a weak	medium	low	hard	-0.699689278
91	12	1	iran	5.5	subcutances three times a weak	high	medium	hard	-0.787756632
91	12	0	other	2.5	subcutances other days	medium	low	hard	-0.352164436
92	1	1	iran	5.5	mascular once a weak	high	medium	hard	-0.572088286
92	1	0	iran	0.5	subcutances three times a weak	high	medium	easy	-0.72867963
92	2	1	other	0.5	subcutances other days	high	medium	hard	-0.643869086
92	2	0	iran	5.5	subcutances other days	medium	medium	hard	-0.928090082
92	3	1	iran	5.5	mascular once a weak	high	medium	hard	-0.572088286
92	3	0	iran	2.5	subcutances three times a weak	high	medium	easy	-0.646894171
92	4	0	iran	0.5	subcutances three times a weak	medium	low	hard	-0.699689278
92	4	1	other	2.5	mascular once a weak	high	low	hard	0.00383736
92	5	0	iran	0.5	mascular once a weak	medium	low	easy	-0.303092094
92	5	1	other	2.5	subcutances three times a weak	high	low	easy	-0.030902146
92	6	1	other	2.5	subcutances three times a weak	high	low	easy	-0.030902146
92	6	0	other	0.5	subcutances other days	high	medium	easy	-0.462940247
92	7	0	other	0.5	mascular once a weak	medium	medium	hard	-0.900801637
92	7	1	other	5.5	subcutances other days	medium	low	hard	-0.312098058
92	8	1	iran	2.5	mascular once a weak	high	low	hard	-0.0957683
92	8	0	iran	5.5	subcutances three times a weak	high	low	easy	-0.090441429
92	9	1	iran	2.5	subcutances three times a weak	medium	low	hard	-0.617903819
92	9	0	other	0.5	subcutances other days	high	medium	hard	-0.643869086
92	10	1	other	2.5	mascular once a weak	high	low	hard	0.00383736
92	10	0	iran	5.5	mascular once a weak	medium	medium	easy	-0.697626621
92	11	1	other	5.5	subcutances other days	high	medium	easy	-0.34108841
92	11	0	iran	0.5	subcutances three times a weak	medium	low	hard	-0.699689278
92	12	0	iran	5.5	subcutances three times a weak	high	medium	hard	-0.787756632
92	12	1	other	2.5	subcutances other days	medium	low	hard	-0.352164436

93	1	0	iran	5.5	mascular once a weak	high	medium	hard	-0.572088286
93	1	1	iran	0.5	subcutances three times a weak	high	medium	easy	-0.72867963
93	2	0	other	0.5	subcutances other days	high	medium	hard	-0.643869086
93	2	1	iran	5.5	subcutances other days	medium	medium	hard	-0.928090082
93	3	1	iran	5.5	mascular once a weak	high	medium	hard	-0.572088286
93	3	0	iran	2.5	subcutances three times a weak	high	medium	easy	-0.646894171
93	4	0	iran	0.5	subcutances three times a weak	medium	low	hard	-0.699689278
93	4	1	other	2.5	mascular once a weak	high	low	hard	0.00383736
93	5	0	iran	0.5	mascular once a weak	medium	low	easy	-0.303092094
93	5	1	other	2.5	subcutances three times a weak	high	low	easy	-0.030902146
93	6	1	other	2.5	subcutances three times a weak	high	low	easy	-0.030902146
93	6	0	other	0.5	subcutances other days	high	medium	easy	-0.462940247
93	7	1	other	0.5	mascular once a weak	medium	medium	hard	-0.900801637
93	7	0	other	5.5	subcutances other days	medium	low	hard	-0.312098058
93	8	1	iran	2.5	mascular once a weak	high	low	hard	-0.0957683
93	8	0	iran	5.5	subcutances three times a weak	high	low	easy	-0.090441429
93	9	1	iran	2.5	subcutances three times a weak	medium	low	hard	-0.617903819
93	9	0	other	0.5	subcutances other days	high	medium	hard	-0.643869086
93	10	0	other	2.5	mascular once a weak	high	low	hard	0.00383736
93	10	1	iran	5.5	mascular once a weak	medium	medium	easy	-0.697626621
93	11	1	other	5.5	subcutances other days	high	medium	easy	-0.34108841
93	11	0	iran	0.5	subcutances three times a weak	medium	low	hard	-0.699689278
93	12	0	iran	5.5	subcutances three times a weak	high	medium	hard	-0.787756632
93	12	1	other	2.5	subcutances other days	medium	low	hard	-0.352164436
94	1	1	iran	5.5	mascular once a weak	high	medium	hard	-0.572088286
94	1	0	iran	0.5	subcutances three times a weak	high	medium	easy	-0.72867963
94	2	0	other	0.5	subcutances other days	high	medium	hard	-0.643869086
94	2	0	iran	5.5	subcutances other days	medium	medium	hard	-0.928090082
94	3	1	iran	5.5	mascular once a weak	high	medium	hard	-0.572088286
94	3	0	iran	2.5	subcutances three times a weak	high	medium	easy	-0.646894171
94	4	0	iran	0.5	subcutances three times a weak	medium	low	hard	-0.699689278
94	4	1	other	2.5	mascular once a weak	high	low	hard	0.00383736
94	5	1	iran	0.5	mascular once a weak	medium	low	easy	-0.303092094
94	5	0	other	2.5	subcutances three times a weak	high	low	easy	-0.030902146

94	6	0	other	2.5	subcutances three times a weak	high	low	easy	-0.030902146
94	6	0	other	0.5	subcutances other days	high	medium	easy	-0.462940247
94	7	1	other	0.5	mascular once a weak	medium	medium	hard	-0.900801637
94	7	0	other	5.5	subcutances other days	medium	low	hard	-0.312098058
94	8	1	iran	2.5	mascular once a weak	high	low	hard	-0.0957683
94	8	0	iran	5.5	subcutances three times a weak	high	low	easy	-0.090441429
94	9	0	iran	2.5	subcutances three times a weak	medium	low	hard	-0.617903819
94	9	0	other	0.5	subcutances other days	high	medium	hard	-0.643869086
94	10	1	other	2.5	mascular once a weak	high	low	hard	0.00383736
94	10	0	iran	5.5	mascular once a weak	medium	medium	easy	-0.697626621
94	11	0	other	5.5	subcutances other days	high	medium	easy	-0.34108841
94	11	0	iran	0.5	subcutances three times a weak	medium	low	hard	-0.699689278
94	12	0	iran	5.5	subcutances three times a weak	high	medium	hard	-0.787756632
94	12	0	other	2.5	subcutances other days	medium	low	hard	-0.352164436
95	1	1	iran	5.5	mascular once a weak	high	medium	hard	-0.572088286
95	1	0	iran	0.5	subcutances three times a weak	high	medium	easy	-0.72867963
95	2	1	other	0.5	subcutances other days	high	medium	hard	-0.643869086
95	2	0	iran	5.5	subcutances other days	medium	medium	hard	-0.928090082
95	3	1	iran	5.5	mascular once a weak	high	medium	hard	-0.572088286
95	3	0	iran	2.5	subcutances three times a weak	high	medium	easy	-0.646894171
95	4	0	iran	0.5	subcutances three times a weak	medium	low	hard	-0.699689278
95	4	1	other	2.5	mascular once a weak	high	low	hard	0.00383736
95	5	0	iran	0.5	mascular once a weak	medium	low	easy	-0.303092094
95	5	1	other	2.5	subcutances three times a weak	high	low	easy	-0.030902146
95	6	1	other	2.5	subcutances three times a weak	high	low	easy	-0.030902146
95	6	0	other	0.5	subcutances other days	high	medium	easy	-0.462940247
95	7	1	other	0.5	mascular once a weak	medium	medium	hard	-0.900801637
95	7	0	other	5.5	subcutances other days	medium	low	hard	-0.312098058
95	8	0	iran	2.5	mascular once a weak	high	low	hard	-0.0957683
95	8	1	iran	5.5	subcutances three times a weak	high	low	easy	-0.090441429
95	9	1	iran	2.5	subcutances three times a weak	medium	low	hard	-0.617903819
95	9	0	other	0.5	subcutances other days	high	medium	hard	-0.643869086
95	10	1	other	2.5	mascular once a weak	high	low	hard	0.00383736
95	10	0	iran	5.5	mascular once a weak	medium	medium	easy	-0.697626621

95	11	1	other	5.5	subcutances other days	high	medium	easy	-0.34108841
95	11	0	iran	0.5	subcutances three times a weak	medium	low	hard	-0.699689278
95	12	1	iran	5.5	subcutances three times a weak	high	medium	hard	-0.787756632
95	12	0	other	2.5	subcutances other days	medium	low	hard	-0.352164436
96	1	0	iran	5.5	mascular once a weak	high	medium	hard	-0.572088286
96	1	1	iran	0.5	subcutances three times a weak	high	medium	easy	-0.72867963
96	2	1	other	0.5	subcutances other days	high	medium	hard	-0.643869086
96	2	0	iran	5.5	subcutances other days	medium	medium	hard	-0.928090082
96	3	0	iran	5.5	mascular once a weak	high	medium	hard	-0.572088286
96	3	1	iran	2.5	subcutances three times a weak	high	medium	easy	-0.646894171
96	4	0	iran	0.5	subcutances three times a weak	medium	low	hard	-0.699689278
96	4	1	other	2.5	mascular once a weak	high	low	hard	0.00383736
96	5	0	iran	0.5	mascular once a weak	medium	low	easy	-0.303092094
96	5	1	other	2.5	subcutances three times a weak	high	low	easy	-0.030902146
96	6	0	other	2.5	subcutances three times a weak	high	low	easy	-0.030902146
96	6	1	other	0.5	subcutances other days	high	medium	easy	-0.462940247
96	7	1	other	0.5	mascular once a weak	medium	medium	hard	-0.900801637
96	7	0	other	5.5	subcutances other days	medium	low	hard	-0.312098058
96	8	0	iran	2.5	mascular once a weak	high	low	hard	-0.0957683
96	8	1	iran	5.5	subcutances three times a weak	high	low	easy	-0.090441429
96	9	0	iran	2.5	subcutances three times a weak	medium	low	hard	-0.617903819
96	9	1	other	0.5	subcutances other days	high	medium	hard	-0.643869086
96	10	0	other	2.5	mascular once a weak	high	low	hard	0.00383736
96	10	1	iran	5.5	mascular once a weak	medium	medium	easy	-0.697626621
96	11	1	other	5.5	subcutances other days	high	medium	easy	-0.34108841
96	11	0	iran	0.5	subcutances three times a weak	medium	low	hard	-0.699689278
96	12	1	iran	5.5	subcutances three times a weak	high	medium	hard	-0.787756632
96	12	0	other	2.5	subcutances other days	medium	low	hard	-0.352164436
97	1	1	iran	5.5	mascular once a weak	high	medium	hard	-0.572088286
97	1	0	iran	0.5	subcutances three times a weak	high	medium	easy	-0.72867963
97	2	0	other	0.5	subcutances other days	high	medium	hard	-0.643869086
97	2	1	iran	5.5	subcutances other days	medium	medium	hard	-0.928090082
97	3	0	iran	5.5	mascular once a weak	high	medium	hard	-0.572088286
97	3	1	iran	2.5	subcutances three times a weak	high	medium	easy	-0.646894171

97	4	1	iran	0.5	subcutances three times a weak	medium	low	hard	-0.699689278
97	4	0	other	2.5	mascular once a weak	high	low	hard	0.00383736
97	5	1	iran	0.5	mascular once a weak	medium	low	easy	-0.303092094
97	5	0	other	2.5	subcutances three times a weak	high	low	easy	-0.030902146
97	6	0	other	2.5	subcutances three times a weak	high	low	easy	-0.030902146
97	6	1	other	0.5	subcutances other days	high	medium	easy	-0.462940247
97	7	1	other	0.5	mascular once a weak	medium	medium	hard	-0.900801637
97	7	0	other	5.5	subcutances other days	medium	low	hard	-0.312098058
97	8	1	iran	2.5	mascular once a weak	high	low	hard	-0.0957683
97	8	0	iran	5.5	subcutances three times a weak	high	low	easy	-0.090441429
97	9	1	iran	2.5	subcutances three times a weak	medium	low	hard	-0.617903819
97	9	0	other	0.5	subcutances other days	high	medium	hard	-0.643869086
97	10	0	other	2.5	mascular once a weak	high	low	hard	0.00383736
97	10	1	iran	5.5	mascular once a weak	medium	medium	easy	-0.697626621
97	11	0	other	5.5	subcutances other days	high	medium	easy	-0.34108841
97	11	1	iran	0.5	subcutances three times a weak	medium	low	hard	-0.699689278
97	12	0	iran	5.5	subcutances three times a weak	high	medium	hard	-0.787756632
97	12	1	other	2.5	subcutances other days	medium	low	hard	-0.352164436
98	1	1	iran	5.5	mascular once a weak	high	medium	hard	-0.572088286
98	1	0	iran	0.5	subcutances three times a weak	high	medium	easy	-0.72867963
98	2	1	other	0.5	subcutances other days	high	medium	hard	-0.643869086
98	2	0	iran	5.5	subcutances other days	medium	medium	hard	-0.928090082
98	3	0	iran	5.5	mascular once a weak	high	medium	hard	-0.572088286
98	3	1	iran	2.5	subcutances three times a weak	high	medium	easy	-0.646894171
98	4	0	iran	0.5	subcutances three times a weak	medium	low	hard	-0.699689278
98	4	1	other	2.5	mascular once a weak	high	low	hard	0.00383736
98	5	0	iran	0.5	mascular once a weak	medium	low	easy	-0.303092094
98	5	1	other	2.5	subcutances three times a weak	high	low	easy	-0.030902146
98	6	0	other	2.5	subcutances three times a weak	high	low	easy	-0.030902146
98	6	1	other	0.5	subcutances other days	high	medium	easy	-0.462940247
98	7	0	other	0.5	mascular once a weak	medium	medium	hard	-0.900801637
98	7	1	other	5.5	subcutances other days	medium	low	hard	-0.312098058
98	8	0	iran	2.5	mascular once a weak	high	low	hard	-0.0957683
98	8	1	iran	5.5	subcutances three times a weak	high	low	easy	-0.090441429

98	9	0	iran	2.5	subcutances three times a weak	medium	low	hard	-0.617903819
98	9	1	other	0.5	subcutances other days	high	medium	hard	-0.643869086
98	10	1	other	2.5	mascular once a weak	high	low	hard	0.00383736
98	10	0	iran	5.5	mascular once a weak	medium	medium	easy	-0.697626621
98	11	1	other	5.5	subcutances other days	high	medium	easy	-0.34108841
98	11	0	iran	0.5	subcutances three times a weak	medium	low	hard	-0.699689278
98	12	1	iran	5.5	subcutances three times a weak	high	medium	hard	-0.787756632
98	12	0	other	2.5	subcutances other days	medium	low	hard	-0.352164436
99	1	1	iran	5.5	mascular once a weak	high	medium	hard	-0.572088286
99	1	0	iran	0.5	subcutances three times a weak	high	medium	easy	-0.72867963
99	2	1	other	0.5	subcutances other days	high	medium	hard	-0.643869086
99	2	0	iran	5.5	subcutances other days	medium	medium	hard	-0.928090082
99	3	1	iran	5.5	mascular once a weak	high	medium	hard	-0.572088286
99	3	0	iran	2.5	subcutances three times a weak	high	medium	easy	-0.646894171
99	4	0	iran	0.5	subcutances three times a weak	medium	low	hard	-0.699689278
99	4	1	other	2.5	mascular once a weak	high	low	hard	0.00383736
99	5	1	iran	0.5	mascular once a weak	medium	low	easy	-0.303092094
99	5	0	other	2.5	subcutances three times a weak	high	low	easy	-0.030902146
99	6	1	other	2.5	subcutances three times a weak	high	low	easy	-0.030902146
99	6	0	other	0.5	subcutances other days	high	medium	easy	-0.462940247
99	7	1	other	0.5	mascular once a weak	medium	medium	hard	-0.900801637
99	7	0	other	5.5	subcutances other days	medium	low	hard	-0.312098058
99	8	1	iran	2.5	mascular once a weak	high	low	hard	-0.0957683
99	8	0	iran	5.5	subcutances three times a weak	high	low	easy	-0.090441429
99	9	0	iran	2.5	subcutances three times a weak	medium	low	hard	-0.617903819
99	9	1	other	0.5	subcutances other days	high	medium	hard	-0.643869086
99	10	1	other	2.5	mascular once a weak	high	low	hard	0.00383736
99	10	0	iran	5.5	mascular once a weak	medium	medium	easy	-0.697626621
99	11	1	other	5.5	subcutances other days	high	medium	easy	-0.34108841
99	11	0	iran	0.5	subcutances three times a weak	medium	low	hard	-0.699689278
99	12	1	iran	5.5	subcutances three times a weak	high	medium	hard	-0.787756632
99	12	0	other	2.5	subcutances other days	medium	low	hard	-0.352164436
100	1	0	iran	5.5	mascular once a weak	high	medium	hard	-0.572088286
100	1	1	iran	0.5	subcutances three times a weak	high	medium	easy	-0.72867963

100	2	1	other	0.5	subcutances	other days	high	medium	hard	-0.643869086
100	2	0	iran	5.5	subcutances	other days	medium	medium	hard	-0.928090082
100	3	0	iran	5.5	mascular	once a weak	high	medium	hard	-0.572088286
100	3	1	iran	2.5	subcutances	three times a weak	high	medium	easy	-0.646894171
100	4	0	iran	0.5	subcutances	three times a weak	medium	low	hard	-0.699689278
100	4	1	other	2.5	mascular	once a weak	high	low	hard	0.00383736
100	5	0	iran	0.5	mascular	once a weak	medium	low	easy	-0.303092094
100	5	1	other	2.5	subcutances	three times a weak	high	low	easy	-0.030902146
100	6	1	other	2.5	subcutances	three times a weak	high	low	easy	-0.030902146
100	6	0	other	0.5	subcutances	other days	high	medium	easy	-0.462940247
100	7	0	other	0.5	mascular	once a weak	medium	medium	hard	-0.900801637
100	7	1	other	5.5	subcutances	other days	medium	low	hard	-0.312098058
100	8	0	iran	2.5	mascular	once a weak	high	low	hard	-0.0957683
100	8	1	iran	5.5	subcutances	three times a weak	high	low	easy	-0.090441429
100	9	1	iran	2.5	subcutances	three times a weak	medium	low	hard	-0.617903819
100	9	0	other	0.5	subcutances	other days	high	medium	hard	-0.643869086
100	10	1	other	2.5	mascular	once a weak	high	low	hard	0.00383736
100	10	0	iran	5.5	mascular	once a weak	medium	medium	easy	-0.697626621
100	11	1	other	5.5	subcutances	other days	high	medium	easy	-0.34108841
100	11	0	iran	0.5	subcutances	three times a weak	medium	low	hard	-0.699689278
100	12	0	iran	5.5	subcutances	three times a weak	high	medium	hard	-0.787756632
100	12	1	other	2.5	subcutances	other days	medium	low	hard	-0.352164436
101	1	1	iran	5.5	mascular	once a weak	high	medium	hard	-0.572088286
101	1	0	iran	0.5	subcutances	three times a weak	high	medium	easy	-0.72867963
101	2	1	other	0.5	subcutances	other days	high	medium	hard	-0.643869086
101	2	0	iran	5.5	subcutances	other days	medium	medium	hard	-0.928090082
101	3	0	iran	5.5	mascular	once a weak	high	medium	hard	-0.572088286
101	3	1	iran	2.5	subcutances	three times a weak	high	medium	easy	-0.646894171
101	4	0	iran	0.5	subcutances	three times a weak	medium	low	hard	-0.699689278
101	4	1	other	2.5	mascular	once a weak	high	low	hard	0.00383736
101	5	0	iran	0.5	mascular	once a weak	medium	low	easy	-0.303092094
101	5	1	other	2.5	subcutances	three times a weak	high	low	easy	-0.030902146
101	6	1	other	2.5	subcutances	three times a weak	high	low	easy	-0.030902146
101	6	0	other	0.5	subcutances	other days	high	medium	easy	-0.462940247

101	7	0	other	0.5	mascular once a weak	medium	medium	hard	-0.900801637
101	7	1	other	5.5	subcutances other days	medium	low	hard	-0.312098058
101	8	0	iran	2.5	mascular once a weak	high	low	hard	-0.0957683
101	8	1	iran	5.5	subcutances three times a weak	high	low	easy	-0.090441429
101	9	1	iran	2.5	subcutances three times a weak	medium	low	hard	-0.617903819
101	9	0	other	0.5	subcutances other days	high	medium	hard	-0.643869086
101	10	1	other	2.5	mascular once a weak	high	low	hard	0.00383736
101	10	0	iran	5.5	mascular once a weak	medium	medium	easy	-0.697626621
101	11	1	other	5.5	subcutances other days	high	medium	easy	-0.34108841
101	11	0	iran	0.5	subcutances three times a weak	medium	low	hard	-0.699689278
101	12	1	iran	5.5	subcutances three times a weak	high	medium	hard	-0.787756632
101	12	0	other	2.5	subcutances other days	medium	low	hard	-0.352164436
102	1	0	iran	5.5	mascular once a weak	high	medium	hard	-0.572088286
102	1	1	iran	0.5	subcutances three times a weak	high	medium	easy	-0.72867963
102	2	1	other	0.5	subcutances other days	high	medium	hard	-0.643869086
102	2	0	iran	5.5	subcutances other days	medium	medium	hard	-0.928090082
102	3	0	iran	5.5	mascular once a weak	high	medium	hard	-0.572088286
102	3	1	iran	2.5	subcutances three times a weak	high	medium	easy	-0.646894171
102	4	0	iran	0.5	subcutances three times a weak	medium	low	hard	-0.699689278
102	4	1	other	2.5	mascular once a weak	high	low	hard	0.00383736
102	5	0	iran	0.5	mascular once a weak	medium	low	easy	-0.303092094
102	5	1	other	2.5	subcutances three times a weak	high	low	easy	-0.030902146
102	6	1	other	2.5	subcutances three times a weak	high	low	easy	-0.030902146
102	6	0	other	0.5	subcutances other days	high	medium	easy	-0.462940247
102	7	0	other	0.5	mascular once a weak	medium	medium	hard	-0.900801637
102	7	1	other	5.5	subcutances other days	medium	low	hard	-0.312098058
102	8	0	iran	2.5	mascular once a weak	high	low	hard	-0.0957683
102	8	1	iran	5.5	subcutances three times a weak	high	low	easy	-0.090441429
102	9	1	iran	2.5	subcutances three times a weak	medium	low	hard	-0.617903819
102	9	0	other	0.5	subcutances other days	high	medium	hard	-0.643869086
102	10	0	other	2.5	mascular once a weak	high	low	hard	0.00383736
102	10	1	iran	5.5	mascular once a weak	medium	medium	easy	-0.697626621
102	11	0	other	5.5	subcutances other days	high	medium	easy	-0.34108841
102	11	1	iran	0.5	subcutances three times a weak	medium	low	hard	-0.699689278

102	12	0	iran	5.5	subcutances three times a weak	high	medium	hard	-0.787756632
102	12	1	other	2.5	subcutances other days	medium	low	hard	-0.352164436
103	1	0	iran	5.5	mascular once a weak	high	medium	hard	-0.572088286
103	1	1	iran	0.5	subcutances three times a weak	high	medium	easy	-0.72867963
103	2	1	other	0.5	subcutances other days	high	medium	hard	-0.643869086
103	2	0	iran	5.5	subcutances other days	medium	medium	hard	-0.928090082
103	3	0	iran	5.5	mascular once a weak	high	medium	hard	-0.572088286
103	3	1	iran	2.5	subcutances three times a weak	high	medium	easy	-0.646894171
103	4	0	iran	0.5	subcutances three times a weak	medium	low	hard	-0.699689278
103	4	1	other	2.5	mascular once a weak	high	low	hard	0.00383736
103	5	0	iran	0.5	mascular once a weak	medium	low	easy	-0.303092094
103	5	1	other	2.5	subcutances three times a weak	high	low	easy	-0.030902146
103	6	1	other	2.5	subcutances three times a weak	high	low	easy	-0.030902146
103	6	0	other	0.5	subcutances other days	high	medium	easy	-0.462940247
103	7	0	other	0.5	mascular once a weak	medium	medium	hard	-0.900801637
103	7	1	other	5.5	subcutances other days	medium	low	hard	-0.312098058
103	8	0	iran	2.5	mascular once a weak	high	low	hard	-0.0957683
103	8	1	iran	5.5	subcutances three times a weak	high	low	easy	-0.090441429
103	9	1	iran	2.5	subcutances three times a weak	medium	low	hard	-0.617903819
103	9	0	other	0.5	subcutances other days	high	medium	hard	-0.643869086
103	10	0	other	2.5	mascular once a weak	high	low	hard	0.00383736
103	10	1	iran	5.5	mascular once a weak	medium	medium	easy	-0.697626621
103	11	1	other	5.5	subcutances other days	high	medium	easy	-0.34108841
103	11	0	iran	0.5	subcutances three times a weak	medium	low	hard	-0.699689278
103	12	0	iran	5.5	subcutances three times a weak	high	medium	hard	-0.787756632
103	12	1	other	2.5	subcutances other days	medium	low	hard	-0.352164436
104	1	1	iran	5.5	mascular once a weak	high	medium	hard	-0.572088286
104	1	0	iran	0.5	subcutances three times a weak	high	medium	easy	-0.72867963
104	2	0	other	0.5	subcutances other days	high	medium	hard	-0.643869086
104	2	1	iran	5.5	subcutances other days	medium	medium	hard	-0.928090082
104	3	0	iran	5.5	mascular once a weak	high	medium	hard	-0.572088286
104	3	1	iran	2.5	subcutances three times a weak	high	medium	easy	-0.646894171
104	4	1	iran	0.5	subcutances three times a weak	medium	low	hard	-0.699689278
104	4	0	other	2.5	mascular once a weak	high	low	hard	0.00383736

104	5	1	iran	0.5	mascular once a weak	medium	low	easy	-0.303092094
104	5	0	other	2.5	subcutances three times a weak	high	low	easy	-0.030902146
104	6	0	other	2.5	subcutances three times a weak	high	low	easy	-0.030902146
104	6	1	other	0.5	subcutances other days	high	medium	easy	-0.462940247
104	7	0	other	0.5	mascular once a weak	medium	medium	hard	-0.900801637
104	7	1	other	5.5	subcutances other days	medium	low	hard	-0.312098058
104	8	1	iran	2.5	mascular once a weak	high	low	hard	-0.0957683
104	8	0	iran	5.5	subcutances three times a weak	high	low	easy	-0.090441429
104	9	1	iran	2.5	subcutances three times a weak	medium	low	hard	-0.617903819
104	9	0	other	0.5	subcutances other days	high	medium	hard	-0.643869086
104	10	0	other	2.5	mascular once a weak	high	low	hard	0.00383736
104	10	1	iran	5.5	mascular once a weak	medium	medium	easy	-0.697626621
104	11	0	other	5.5	subcutances other days	high	medium	easy	-0.34108841
104	11	1	iran	0.5	subcutances three times a weak	medium	low	hard	-0.699689278
104	12	0	iran	5.5	subcutances three times a weak	high	medium	hard	-0.787756632
104	12	1	other	2.5	subcutances other days	medium	low	hard	-0.352164436
105	1	1	iran	5.5	mascular once a weak	high	medium	hard	-0.572088286
105	1	0	iran	0.5	subcutances three times a weak	high	medium	easy	-0.72867963
105	2	0	other	0.5	subcutances other days	high	medium	hard	-0.643869086
105	2	1	iran	5.5	subcutances other days	medium	medium	hard	-0.928090082
105	3	0	iran	5.5	mascular once a weak	high	medium	hard	-0.572088286
105	3	1	iran	2.5	subcutances three times a weak	high	medium	easy	-0.646894171
105	4	0	iran	0.5	subcutances three times a weak	medium	low	hard	-0.699689278
105	4	1	other	2.5	mascular once a weak	high	low	hard	0.00383736
105	5	0	iran	0.5	mascular once a weak	medium	low	easy	-0.303092094
105	5	1	other	2.5	subcutances three times a weak	high	low	easy	-0.030902146
105	6	0	other	2.5	subcutances three times a weak	high	low	easy	-0.030902146
105	6	1	other	0.5	subcutances other days	high	medium	easy	-0.462940247
105	7	1	other	0.5	mascular once a weak	medium	medium	hard	-0.900801637
105	7	0	other	5.5	subcutances other days	medium	low	hard	-0.312098058
105	8	1	iran	2.5	mascular once a weak	high	low	hard	-0.0957683
105	8	0	iran	5.5	subcutances three times a weak	high	low	easy	-0.090441429
105	9	1	iran	2.5	subcutances three times a weak	medium	low	hard	-0.617903819
105	9	0	other	0.5	subcutances other days	high	medium	hard	-0.643869086

105	10	0	other	2.5	mascular once a weak	high	low	hard	0.00383736
105	10	1	iran	5.5	mascular once a weak	medium	medium	easy	-0.697626621
105	11	0	other	5.5	subcutances other days	high	medium	easy	-0.34108841
105	11	1	iran	0.5	subcutances three times a weak	medium	low	hard	-0.699689278
105	12	0	iran	5.5	subcutances three times a weak	high	medium	hard	-0.787756632
105	12	1	other	2.5	subcutances other days	medium	low	hard	-0.352164436
106	1	1	iran	5.5	mascular once a weak	high	medium	hard	-0.572088286
106	1	0	iran	0.5	subcutances three times a weak	high	medium	easy	-0.72867963
106	2	0	other	0.5	subcutances other days	high	medium	hard	-0.643869086
106	2	1	iran	5.5	subcutances other days	medium	medium	hard	-0.928090082
106	3	0	iran	5.5	mascular once a weak	high	medium	hard	-0.572088286
106	3	1	iran	2.5	subcutances three times a weak	high	medium	easy	-0.646894171
106	4	1	iran	0.5	subcutances three times a weak	medium	low	hard	-0.699689278
106	4	0	other	2.5	mascular once a weak	high	low	hard	0.00383736
106	5	1	iran	0.5	mascular once a weak	medium	low	easy	-0.303092094
106	5	0	other	2.5	subcutances three times a weak	high	low	easy	-0.030902146
106	6	0	other	2.5	subcutances three times a weak	high	low	easy	-0.030902146
106	6	1	other	0.5	subcutances other days	high	medium	easy	-0.462940247
106	7	0	other	0.5	mascular once a weak	medium	medium	hard	-0.900801637
106	7	1	other	5.5	subcutances other days	medium	low	hard	-0.312098058
106	8	1	iran	2.5	mascular once a weak	high	low	hard	-0.0957683
106	8	0	iran	5.5	subcutances three times a weak	high	low	easy	-0.090441429
106	9	1	iran	2.5	subcutances three times a weak	medium	low	hard	-0.617903819
106	9	0	other	0.5	subcutances other days	high	medium	hard	-0.643869086
106	10	0	other	2.5	mascular once a weak	high	low	hard	0.00383736
106	10	1	iran	5.5	mascular once a weak	medium	medium	easy	-0.697626621
106	11	0	other	5.5	subcutances other days	high	medium	easy	-0.34108841
106	11	1	iran	0.5	subcutances three times a weak	medium	low	hard	-0.699689278
106	12	0	iran	5.5	subcutances three times a weak	high	medium	hard	-0.787756632
106	12	1	other	2.5	subcutances other days	medium	low	hard	-0.352164436
107	1	1	iran	5.5	mascular once a weak	high	medium	hard	-0.572088286
107	1	0	iran	0.5	subcutances three times a weak	high	medium	easy	-0.72867963
107	2	0	other	0.5	subcutances other days	high	medium	hard	-0.643869086
107	2	1	iran	5.5	subcutances other days	medium	medium	hard	-0.928090082

107	3	1	iran	5.5	mascular once a weak	high	medium	hard	-0.572088286
107	3	0	iran	2.5	subcutances three times a weak	high	medium	easy	-0.646894171
107	4	1	iran	0.5	subcutances three times a weak	medium	low	hard	-0.699689278
107	4	0	other	2.5	mascular once a weak	high	low	hard	0.00383736
107	5	1	iran	0.5	mascular once a weak	medium	low	easy	-0.303092094
107	5	0	other	2.5	subcutances three times a weak	high	low	easy	-0.030902146
107	6	0	other	2.5	subcutances three times a weak	high	low	easy	-0.030902146
107	6	1	other	0.5	subcutances other days	high	medium	easy	-0.462940247
107	7	0	other	0.5	mascular once a weak	medium	medium	hard	-0.900801637
107	7	1	other	5.5	subcutances other days	medium	low	hard	-0.312098058
107	8	1	iran	2.5	mascular once a weak	high	low	hard	-0.0957683
107	8	0	iran	5.5	subcutances three times a weak	high	low	easy	-0.090441429
107	9	1	iran	2.5	subcutances three times a weak	medium	low	hard	-0.617903819
107	9	0	other	0.5	subcutances other days	high	medium	hard	-0.643869086
107	10	0	other	2.5	mascular once a weak	high	low	hard	0.00383736
107	10	1	iran	5.5	mascular once a weak	medium	medium	easy	-0.697626621
107	11	0	other	5.5	subcutances other days	high	medium	easy	-0.34108841
107	11	1	iran	0.5	subcutances three times a weak	medium	low	hard	-0.699689278
107	12	0	iran	5.5	subcutances three times a weak	high	medium	hard	-0.787756632
107	12	1	other	2.5	subcutances other days	medium	low	hard	-0.352164436
108	1	0	iran	5.5	mascular once a weak	high	medium	hard	-0.572088286
108	1	1	iran	0.5	subcutances three times a weak	high	medium	easy	-0.72867963
108	2	1	other	0.5	subcutances other days	high	medium	hard	-0.643869086
108	2	0	iran	5.5	subcutances other days	medium	medium	hard	-0.928090082
108	3	0	iran	5.5	mascular once a weak	high	medium	hard	-0.572088286
108	3	0	iran	2.5	subcutances three times a weak	high	medium	easy	-0.646894171
108	4	0	iran	0.5	subcutances three times a weak	medium	low	hard	-0.699689278
108	4	1	other	2.5	mascular once a weak	high	low	hard	0.00383736
108	5	0	iran	0.5	mascular once a weak	medium	low	easy	-0.303092094
108	5	1	other	2.5	subcutances three times a weak	high	low	easy	-0.030902146
108	6	1	other	2.5	subcutances three times a weak	high	low	easy	-0.030902146
108	6	0	other	0.5	subcutances other days	high	medium	easy	-0.462940247
108	7	0	other	0.5	mascular once a weak	medium	medium	hard	-0.900801637
108	7	1	other	5.5	subcutances other days	medium	low	hard	-0.312098058

108	8	0	iran	2.5	mascular once a weak	high	low	hard	-0.0957683
108	8	1	iran	5.5	subcutances three times a weak	high	low	easy	-0.090441429
108	9	0	iran	2.5	subcutances three times a weak	medium	low	hard	-0.617903819
108	9	1	other	0.5	subcutances other days	high	medium	hard	-0.643869086
108	10	1	other	2.5	mascular once a weak	high	low	hard	0.00383736
108	10	0	iran	5.5	mascular once a weak	medium	medium	easy	-0.697626621
108	11	1	other	5.5	subcutances other days	high	medium	easy	-0.34108841
108	11	0	iran	0.5	subcutances three times a weak	medium	low	hard	-0.699689278
108	12	0	iran	5.5	subcutances three times a weak	high	medium	hard	-0.787756632
108	12	1	other	2.5	subcutances other days	medium	low	hard	-0.352164436
109	1	1	iran	5.5	mascular once a weak	high	medium	hard	-0.572088286
109	1	0	iran	0.5	subcutances three times a weak	high	medium	easy	-0.72867963
109	2	0	other	0.5	subcutances other days	high	medium	hard	-0.643869086
109	2	1	iran	5.5	subcutances other days	medium	medium	hard	-0.928090082
109	3	0	iran	5.5	mascular once a weak	high	medium	hard	-0.572088286
109	3	1	iran	2.5	subcutances three times a weak	high	medium	easy	-0.646894171
109	4	1	iran	0.5	subcutances three times a weak	medium	low	hard	-0.699689278
109	4	0	other	2.5	mascular once a weak	high	low	hard	0.00383736
109	5	1	iran	0.5	mascular once a weak	medium	low	easy	-0.303092094
109	5	0	other	2.5	subcutances three times a weak	high	low	easy	-0.030902146
109	6	0	other	2.5	subcutances three times a weak	high	low	easy	-0.030902146
109	6	1	other	0.5	subcutances other days	high	medium	easy	-0.462940247
109	7	0	other	0.5	mascular once a weak	medium	medium	hard	-0.900801637
109	7	1	other	5.5	subcutances other days	medium	low	hard	-0.312098058
109	8	1	iran	2.5	mascular once a weak	high	low	hard	-0.0957683
109	8	0	iran	5.5	subcutances three times a weak	high	low	easy	-0.090441429
109	9	1	iran	2.5	subcutances three times a weak	medium	low	hard	-0.617903819
109	9	0	other	0.5	subcutances other days	high	medium	hard	-0.643869086
109	10	0	other	2.5	mascular once a weak	high	low	hard	0.00383736
109	10	1	iran	5.5	mascular once a weak	medium	medium	easy	-0.697626621
109	11	0	other	5.5	subcutances other days	high	medium	easy	-0.34108841
109	11	1	iran	0.5	subcutances three times a weak	medium	low	hard	-0.699689278
109	12	0	iran	5.5	subcutances three times a weak	high	medium	hard	-0.787756632
109	12	1	other	2.5	subcutances other days	medium	low	hard	-0.352164436

110	1	1	iran	5.5	mascular once a weak	high	medium	hard	-0.572088286
110	1	0	iran	0.5	subcutances three times a weak	high	medium	easy	-0.72867963
110	2	0	other	0.5	subcutances other days	high	medium	hard	-0.643869086
110	2	1	iran	5.5	subcutances other days	medium	medium	hard	-0.928090082
110	3	1	iran	5.5	mascular once a weak	high	medium	hard	-0.572088286
110	3	0	iran	2.5	subcutances three times a weak	high	medium	easy	-0.646894171
110	4	0	iran	0.5	subcutances three times a weak	medium	low	hard	-0.699689278
110	4	1	other	2.5	mascular once a weak	high	low	hard	0.00383736
110	5	0	iran	0.5	mascular once a weak	medium	low	easy	-0.303092094
110	5	1	other	2.5	subcutances three times a weak	high	low	easy	-0.030902146
110	6	1	other	2.5	subcutances three times a weak	high	low	easy	-0.030902146
110	6	0	other	0.5	subcutances other days	high	medium	easy	-0.462940247
110	7	1	other	0.5	mascular once a weak	medium	medium	hard	-0.900801637
110	7	0	other	5.5	subcutances other days	medium	low	hard	-0.312098058
110	8	1	iran	2.5	mascular once a weak	high	low	hard	-0.0957683
110	8	0	iran	5.5	subcutances three times a weak	high	low	easy	-0.090441429
110	9	0	iran	2.5	subcutances three times a weak	medium	low	hard	-0.617903819
110	9	1	other	0.5	subcutances other days	high	medium	hard	-0.643869086
110	10	1	other	2.5	mascular once a weak	high	low	hard	0.00383736
110	10	0	iran	5.5	mascular once a weak	medium	medium	easy	-0.697626621
110	11	1	other	5.5	subcutances other days	high	medium	easy	-0.34108841
110	11	0	iran	0.5	subcutances three times a weak	medium	low	hard	-0.699689278
110	12	1	iran	5.5	subcutances three times a weak	high	medium	hard	-0.787756632
110	12	0	other	2.5	subcutances other days	medium	low	hard	-0.352164436
111	1	1	iran	5.5	mascular once a weak	high	medium	hard	-0.572088286
111	1	0	iran	0.5	subcutances three times a weak	high	medium	easy	-0.72867963
111	2	0	other	0.5	subcutances other days	high	medium	hard	-0.643869086
111	2	1	iran	5.5	subcutances other days	medium	medium	hard	-0.928090082
111	3	0	iran	5.5	mascular once a weak	high	medium	hard	-0.572088286
111	3	1	iran	2.5	subcutances three times a weak	high	medium	easy	-0.646894171
111	4	1	iran	0.5	subcutances three times a weak	medium	low	hard	-0.699689278
111	4	0	other	2.5	mascular once a weak	high	low	hard	0.00383736
111	5	1	iran	0.5	mascular once a weak	medium	low	easy	-0.303092094
111	5	0	other	2.5	subcutances three times a weak	high	low	easy	-0.030902146

111	6	0	other	2.5	subcutances three times a weak	high	low	easy	-0.030902146
111	6	1	other	0.5	subcutances other days	high	medium	easy	-0.462940247
111	7	0	other	0.5	mascular once a weak	medium	medium	hard	-0.900801637
111	7	1	other	5.5	subcutances other days	medium	low	hard	-0.312098058
111	8	1	iran	2.5	mascular once a weak	high	low	hard	-0.0957683
111	8	0	iran	5.5	subcutances three times a weak	high	low	easy	-0.090441429
111	9	1	iran	2.5	subcutances three times a weak	medium	low	hard	-0.617903819
111	9	0	other	0.5	subcutances other days	high	medium	hard	-0.643869086
111	10	0	other	2.5	mascular once a weak	high	low	hard	0.00383736
111	10	1	iran	5.5	mascular once a weak	medium	medium	easy	-0.697626621
111	11	0	other	5.5	subcutances other days	high	medium	easy	-0.34108841
111	11	1	iran	0.5	subcutances three times a weak	medium	low	hard	-0.699689278
111	12	0	iran	5.5	subcutances three times a weak	high	medium	hard	-0.787756632
111	12	1	other	2.5	subcutances other days	medium	low	hard	-0.352164436
112	1	1	iran	5.5	mascular once a weak	high	medium	hard	-0.572088286
112	1	0	iran	0.5	subcutances three times a weak	high	medium	easy	-0.72867963
112	2	0	other	0.5	subcutances other days	high	medium	hard	-0.643869086
112	2	1	iran	5.5	subcutances other days	medium	medium	hard	-0.928090082
112	3	0	iran	5.5	mascular once a weak	high	medium	hard	-0.572088286
112	3	1	iran	2.5	subcutances three times a weak	high	medium	easy	-0.646894171
112	4	1	iran	0.5	subcutances three times a weak	medium	low	hard	-0.699689278
112	4	0	other	2.5	mascular once a weak	high	low	hard	0.00383736
112	5	1	iran	0.5	mascular once a weak	medium	low	easy	-0.303092094
112	5	0	other	2.5	subcutances three times a weak	high	low	easy	-0.030902146
112	6	0	other	2.5	subcutances three times a weak	high	low	easy	-0.030902146
112	6	1	other	0.5	subcutances other days	high	medium	easy	-0.462940247
112	7	0	other	0.5	mascular once a weak	medium	medium	hard	-0.900801637
112	7	1	other	5.5	subcutances other days	medium	low	hard	-0.312098058
112	8	1	iran	2.5	mascular once a weak	high	low	hard	-0.0957683
112	8	0	iran	5.5	subcutances three times a weak	high	low	easy	-0.090441429
112	9	1	iran	2.5	subcutances three times a weak	medium	low	hard	-0.617903819
112	9	0	other	0.5	subcutances other days	high	medium	hard	-0.643869086
112	10	0	other	2.5	mascular once a weak	high	low	hard	0.00383736
112	10	1	iran	5.5	mascular once a weak	medium	medium	easy	-0.697626621

112	11	0	other	5.5	subcutances other days	high	medium	easy	-0.34108841
112	11	1	iran	0.5	subcutances three times a weak	medium	low	hard	-0.699689278
112	12	0	iran	5.5	subcutances three times a weak	high	medium	hard	-0.787756632
112	12	1	other	2.5	subcutances other days	medium	low	hard	-0.352164436
113	1	1	iran	5.5	mascular once a weak	high	medium	hard	-0.572088286
113	1	0	iran	0.5	subcutances three times a weak	high	medium	easy	-0.72867963
113	2	0	other	0.5	subcutances other days	high	medium	hard	-0.643869086
113	2	1	iran	5.5	subcutances other days	medium	medium	hard	-0.928090082
113	3	1	iran	5.5	mascular once a weak	high	medium	hard	-0.572088286
113	3	0	iran	2.5	subcutances three times a weak	high	medium	easy	-0.646894171
113	4	0	iran	0.5	subcutances three times a weak	medium	low	hard	-0.699689278
113	4	1	other	2.5	mascular once a weak	high	low	hard	0.00383736
113	5	1	iran	0.5	mascular once a weak	medium	low	easy	-0.303092094
113	5	0	other	2.5	subcutances three times a weak	high	low	easy	-0.030902146
113	6	0	other	2.5	subcutances three times a weak	high	low	easy	-0.030902146
113	6	1	other	0.5	subcutances other days	high	medium	easy	-0.462940247
113	7	1	other	0.5	mascular once a weak	medium	medium	hard	-0.900801637
113	7	0	other	5.5	subcutances other days	medium	low	hard	-0.312098058
113	8	1	iran	2.5	mascular once a weak	high	low	hard	-0.0957683
113	8	0	iran	5.5	subcutances three times a weak	high	low	easy	-0.090441429
113	9	1	iran	2.5	subcutances three times a weak	medium	low	hard	-0.617903819
113	9	0	other	0.5	subcutances other days	high	medium	hard	-0.643869086
113	10	1	other	2.5	mascular once a weak	high	low	hard	0.00383736
113	10	0	iran	5.5	mascular once a weak	medium	medium	easy	-0.697626621
113	11	0	other	5.5	subcutances other days	high	medium	easy	-0.34108841
113	11	1	iran	0.5	subcutances three times a weak	medium	low	hard	-0.699689278
113	12	0	iran	5.5	subcutances three times a weak	high	medium	hard	-0.787756632
113	12	1	other	2.5	subcutances other days	medium	low	hard	-0.352164436
114	1	0	iran	5.5	mascular once a weak	high	medium	hard	-0.572088286
114	1	1	iran	0.5	subcutances three times a weak	high	medium	easy	-0.72867963
114	2	1	other	0.5	subcutances other days	high	medium	hard	-0.643869086
114	2	0	iran	5.5	subcutances other days	medium	medium	hard	-0.928090082
114	3	1	iran	5.5	mascular once a weak	high	medium	hard	-0.572088286
114	3	0	iran	2.5	subcutances three times a weak	high	medium	easy	-0.646894171

114	4	0	iran	0.5	subcutances three times a weak	medium	low	hard	-0.699689278
114	4	1	other	2.5	mascular once a weak	high	low	hard	0.00383736
114	5	0	iran	0.5	mascular once a weak	medium	low	easy	-0.303092094
114	5	1	other	2.5	subcutances three times a weak	high	low	easy	-0.030902146
114	6	0	other	2.5	subcutances three times a weak	high	low	easy	-0.030902146
114	6	1	other	0.5	subcutances other days	high	medium	easy	-0.462940247
114	7	1	other	0.5	mascular once a weak	medium	medium	hard	-0.900801637
114	7	0	other	5.5	subcutances other days	medium	low	hard	-0.312098058
114	8	1	iran	2.5	mascular once a weak	high	low	hard	-0.0957683
114	8	0	iran	5.5	subcutances three times a weak	high	low	easy	-0.090441429
114	9	0	iran	2.5	subcutances three times a weak	medium	low	hard	-0.617903819
114	9	1	other	0.5	subcutances other days	high	medium	hard	-0.643869086
114	10	1	other	2.5	mascular once a weak	high	low	hard	0.00383736
114	10	0	iran	5.5	mascular once a weak	medium	medium	easy	-0.697626621
114	11	1	other	5.5	subcutances other days	high	medium	easy	-0.34108841
114	11	0	iran	0.5	subcutances three times a weak	medium	low	hard	-0.699689278
114	12	0	iran	5.5	subcutances three times a weak	high	medium	hard	-0.787756632
114	12	1	other	2.5	subcutances other days	medium	low	hard	-0.352164436
115	1	1	iran	5.5	mascular once a weak	high	medium	hard	-0.572088286
115	1	0	iran	0.5	subcutances three times a weak	high	medium	easy	-0.72867963
115	2	0	other	0.5	subcutances other days	high	medium	hard	-0.643869086
115	2	1	iran	5.5	subcutances other days	medium	medium	hard	-0.928090082
115	3	0	iran	5.5	mascular once a weak	high	medium	hard	-0.572088286
115	3	1	iran	2.5	subcutances three times a weak	high	medium	easy	-0.646894171
115	4	1	iran	0.5	subcutances three times a weak	medium	low	hard	-0.699689278
115	4	0	other	2.5	mascular once a weak	high	low	hard	0.00383736
115	5	1	iran	0.5	mascular once a weak	medium	low	easy	-0.303092094
115	5	0	other	2.5	subcutances three times a weak	high	low	easy	-0.030902146
115	6	1	other	2.5	subcutances three times a weak	high	low	easy	-0.030902146
115	6	0	other	0.5	subcutances other days	high	medium	easy	-0.462940247
115	7	0	other	0.5	mascular once a weak	medium	medium	hard	-0.900801637
115	7	1	other	5.5	subcutances other days	medium	low	hard	-0.312098058
115	8	1	iran	2.5	mascular once a weak	high	low	hard	-0.0957683
115	8	0	iran	5.5	subcutances three times a weak	high	low	easy	-0.090441429

115	9	1	iran	2.5	subcutances three times a weak	medium	low	hard	-0.617903819
115	9	0	other	0.5	subcutances other days	high	medium	hard	-0.643869086
115	10	1	other	2.5	mascular once a weak	high	low	hard	0.00383736
115	10	0	iran	5.5	mascular once a weak	medium	medium	easy	-0.697626621
115	11	0	other	5.5	subcutances other days	high	medium	easy	-0.34108841
115	11	1	iran	0.5	subcutances three times a weak	medium	low	hard	-0.699689278
115	12	0	iran	5.5	subcutances three times a weak	high	medium	hard	-0.787756632
115	12	1	other	2.5	subcutances other days	medium	low	hard	-0.352164436
116	1	1	iran	5.5	mascular once a weak	high	medium	hard	-0.572088286
116	1	0	iran	0.5	subcutances three times a weak	high	medium	easy	-0.72867963
116	2	0	other	0.5	subcutances other days	high	medium	hard	-0.643869086
116	2	1	iran	5.5	subcutances other days	medium	medium	hard	-0.928090082
116	3	0	iran	5.5	mascular once a weak	high	medium	hard	-0.572088286
116	3	1	iran	2.5	subcutances three times a weak	high	medium	easy	-0.646894171
116	4	1	iran	0.5	subcutances three times a weak	medium	low	hard	-0.699689278
116	4	0	other	2.5	mascular once a weak	high	low	hard	0.00383736
116	5	1	iran	0.5	mascular once a weak	medium	low	easy	-0.303092094
116	5	0	other	2.5	subcutances three times a weak	high	low	easy	-0.030902146
116	6	0	other	2.5	subcutances three times a weak	high	low	easy	-0.030902146
116	6	1	other	0.5	subcutances other days	high	medium	easy	-0.462940247
116	7	0	other	0.5	mascular once a weak	medium	medium	hard	-0.900801637
116	7	1	other	5.5	subcutances other days	medium	low	hard	-0.312098058
116	8	1	iran	2.5	mascular once a weak	high	low	hard	-0.0957683
116	8	0	iran	5.5	subcutances three times a weak	high	low	easy	-0.090441429
116	9	1	iran	2.5	subcutances three times a weak	medium	low	hard	-0.617903819
116	9	0	other	0.5	subcutances other days	high	medium	hard	-0.643869086
116	10	0	other	2.5	mascular once a weak	high	low	hard	0.00383736
116	10	1	iran	5.5	mascular once a weak	medium	medium	easy	-0.697626621
116	11	0	other	5.5	subcutances other days	high	medium	easy	-0.34108841
116	11	1	iran	0.5	subcutances three times a weak	medium	low	hard	-0.699689278
116	12	0	iran	5.5	subcutances three times a weak	high	medium	hard	-0.787756632
116	12	1	other	2.5	subcutances other days	medium	low	hard	-0.352164436
117	1	1	iran	5.5	mascular once a weak	high	medium	hard	-0.572088286
117	1	0	iran	0.5	subcutances three times a weak	high	medium	easy	-0.72867963

117	2	0	other	0.5	subcutances	other days	high	medium	hard	-0.643869086
117	2	1	iran	5.5	subcutances	other days	medium	medium	hard	-0.928090082
117	3	0	iran	5.5	mascular	once a weak	high	medium	hard	-0.572088286
117	3	1	iran	2.5	subcutances	three times a weak	high	medium	easy	-0.646894171
117	4	1	iran	0.5	subcutances	three times a weak	medium	low	hard	-0.699689278
117	4	0	other	2.5	mascular	once a weak	high	low	hard	0.00383736
117	5	1	iran	0.5	mascular	once a weak	medium	low	easy	-0.303092094
117	5	0	other	2.5	subcutances	three times a weak	high	low	easy	-0.030902146
117	6	0	other	2.5	subcutances	three times a weak	high	low	easy	-0.030902146
117	6	1	other	0.5	subcutances	other days	high	medium	easy	-0.462940247
117	7	0	other	0.5	mascular	once a weak	medium	medium	hard	-0.900801637
117	7	1	other	5.5	subcutances	other days	medium	low	hard	-0.312098058
117	8	1	iran	2.5	mascular	once a weak	high	low	hard	-0.0957683
117	8	0	iran	5.5	subcutances	three times a weak	high	low	easy	-0.090441429
117	9	1	iran	2.5	subcutances	three times a weak	medium	low	hard	-0.617903819
117	9	0	other	0.5	subcutances	other days	high	medium	hard	-0.643869086
117	10	0	other	2.5	mascular	once a weak	high	low	hard	0.00383736
117	10	1	iran	5.5	mascular	once a weak	medium	medium	easy	-0.697626621
117	11	0	other	5.5	subcutances	other days	high	medium	easy	-0.34108841
117	11	1	iran	0.5	subcutances	three times a weak	medium	low	hard	-0.699689278
117	12	0	iran	5.5	subcutances	three times a weak	high	medium	hard	-0.787756632
117	12	1	other	2.5	subcutances	other days	medium	low	hard	-0.352164436
118	1	0	iran	5.5	mascular	once a weak	high	medium	hard	-0.572088286
118	1	1	iran	0.5	subcutances	three times a weak	high	medium	easy	-0.72867963
118	2	1	other	0.5	subcutances	other days	high	medium	hard	-0.643869086
118	2	0	iran	5.5	subcutances	other days	medium	medium	hard	-0.928090082
118	3	0	iran	5.5	mascular	once a weak	high	medium	hard	-0.572088286
118	3	1	iran	2.5	subcutances	three times a weak	high	medium	easy	-0.646894171
118	4	0	iran	0.5	subcutances	three times a weak	medium	low	hard	-0.699689278
118	4	1	other	2.5	mascular	once a weak	high	low	hard	0.00383736
118	5	1	iran	0.5	mascular	once a weak	medium	low	easy	-0.303092094
118	5	0	other	2.5	subcutances	three times a weak	high	low	easy	-0.030902146
118	6	1	other	2.5	subcutances	three times a weak	high	low	easy	-0.030902146
118	6	0	other	0.5	subcutances	other days	high	medium	easy	-0.462940247

118	7	1	other	0.5	mascular once a weak	medium	medium	hard	-0.900801637
118	7	0	other	5.5	subcutances other days	medium	low	hard	-0.312098058
118	8	0	iran	2.5	mascular once a weak	high	low	hard	-0.0957683
118	8	1	iran	5.5	subcutances three times a weak	high	low	easy	-0.090441429
118	9	1	iran	2.5	subcutances three times a weak	medium	low	hard	-0.617903819
118	9	0	other	0.5	subcutances other days	high	medium	hard	-0.643869086
118	10	0	other	2.5	mascular once a weak	high	low	hard	0.00383736
118	10	1	iran	5.5	mascular once a weak	medium	medium	easy	-0.697626621
118	11	1	other	5.5	subcutances other days	high	medium	easy	-0.34108841
118	11	0	iran	0.5	subcutances three times a weak	medium	low	hard	-0.699689278
118	12	1	iran	5.5	subcutances three times a weak	high	medium	hard	-0.787756632
118	12	0	other	2.5	subcutances other days	medium	low	hard	-0.352164436
119	1	1	iran	5.5	mascular once a weak	high	medium	hard	-0.572088286
119	1	0	iran	0.5	subcutances three times a weak	high	medium	easy	-0.72867963
119	2	0	other	0.5	subcutances other days	high	medium	hard	-0.643869086
119	2	1	iran	5.5	subcutances other days	medium	medium	hard	-0.928090082
119	3	0	iran	5.5	mascular once a weak	high	medium	hard	-0.572088286
119	3	1	iran	2.5	subcutances three times a weak	high	medium	easy	-0.646894171
119	4	1	iran	0.5	subcutances three times a weak	medium	low	hard	-0.699689278
119	4	0	other	2.5	mascular once a weak	high	low	hard	0.00383736
119	5	1	iran	0.5	mascular once a weak	medium	low	easy	-0.303092094
119	5	0	other	2.5	subcutances three times a weak	high	low	easy	-0.030902146
119	6	0	other	2.5	subcutances three times a weak	high	low	easy	-0.030902146
119	6	1	other	0.5	subcutances other days	high	medium	easy	-0.462940247
119	7	0	other	0.5	mascular once a weak	medium	medium	hard	-0.900801637
119	7	1	other	5.5	subcutances other days	medium	low	hard	-0.312098058
119	8	1	iran	2.5	mascular once a weak	high	low	hard	-0.0957683
119	8	0	iran	5.5	subcutances three times a weak	high	low	easy	-0.090441429
119	9	1	iran	2.5	subcutances three times a weak	medium	low	hard	-0.617903819
119	9	0	other	0.5	subcutances other days	high	medium	hard	-0.643869086
119	10	0	other	2.5	mascular once a weak	high	low	hard	0.00383736
119	10	1	iran	5.5	mascular once a weak	medium	medium	easy	-0.697626621
119	11	0	other	5.5	subcutances other days	high	medium	easy	-0.34108841
119	11	1	iran	0.5	subcutances three times a weak	medium	low	hard	-0.699689278

119	12	0	iran	5.5	subcutances three times a weak	high	medium	hard	-0.787756632
119	12	1	other	2.5	subcutances other days	medium	low	hard	-0.352164436
120	1	0	iran	5.5	mascular once a weak	high	medium	hard	-0.572088286
120	1	1	iran	0.5	subcutances three times a weak	high	medium	easy	-0.72867963
120	2	1	other	0.5	subcutances other days	high	medium	hard	-0.643869086
120	2	0	iran	5.5	subcutances other days	medium	medium	hard	-0.928090082
120	3	0	iran	5.5	mascular once a weak	high	medium	hard	-0.572088286
120	3	1	iran	2.5	subcutances three times a weak	high	medium	easy	-0.646894171
120	4	0	iran	0.5	subcutances three times a weak	medium	low	hard	-0.699689278
120	4	1	other	2.5	mascular once a weak	high	low	hard	0.00383736
120	5	0	iran	0.5	mascular once a weak	medium	low	easy	-0.303092094
120	5	1	other	2.5	subcutances three times a weak	high	low	easy	-0.030902146
120	6	1	other	2.5	subcutances three times a weak	high	low	easy	-0.030902146
120	6	0	other	0.5	subcutances other days	high	medium	easy	-0.462940247
120	7	0	other	0.5	mascular once a weak	medium	medium	hard	-0.900801637
120	7	1	other	5.5	subcutances other days	medium	low	hard	-0.312098058
120	8	1	iran	2.5	mascular once a weak	high	low	hard	-0.0957683
120	8	0	iran	5.5	subcutances three times a weak	high	low	easy	-0.090441429
120	9	0	iran	2.5	subcutances three times a weak	medium	low	hard	-0.617903819
120	9	1	other	0.5	subcutances other days	high	medium	hard	-0.643869086
120	10	1	other	2.5	mascular once a weak	high	low	hard	0.00383736
120	10	0	iran	5.5	mascular once a weak	medium	medium	easy	-0.697626621
120	11	1	other	5.5	subcutances other days	high	medium	easy	-0.34108841
120	11	0	iran	0.5	subcutances three times a weak	medium	low	hard	-0.699689278
120	12	1	iran	5.5	subcutances three times a weak	high	medium	hard	-0.787756632
120	12	0	other	2.5	subcutances other days	medium	low	hard	-0.352164436
121	1	1	iran	5.5	mascular once a weak	high	medium	hard	-0.572088286
121	1	0	iran	0.5	subcutances three times a weak	high	medium	easy	-0.72867963
121	2	0	other	0.5	subcutances other days	high	medium	hard	-0.643869086
121	2	1	iran	5.5	subcutances other days	medium	medium	hard	-0.928090082
121	3	0	iran	5.5	mascular once a weak	high	medium	hard	-0.572088286
121	3	1	iran	2.5	subcutances three times a weak	high	medium	easy	-0.646894171
121	4	0	iran	0.5	subcutances three times a weak	medium	low	hard	-0.699689278
121	4	1	other	2.5	mascular once a weak	high	low	hard	0.00383736

121	5	1	iran	0.5	mascular once a weak	medium	low	easy	-0.303092094
121	5	0	other	2.5	subcutances three times a weak	high	low	easy	-0.030902146
121	6	0	other	2.5	subcutances three times a weak	high	low	easy	-0.030902146
121	6	1	other	0.5	subcutances other days	high	medium	easy	-0.462940247
121	7	1	other	0.5	mascular once a weak	medium	medium	hard	-0.900801637
121	7	0	other	5.5	subcutances other days	medium	low	hard	-0.312098058
121	8	1	iran	2.5	mascular once a weak	high	low	hard	-0.0957683
121	8	0	iran	5.5	subcutances three times a weak	high	low	easy	-0.090441429
121	9	1	iran	2.5	subcutances three times a weak	medium	low	hard	-0.617903819
121	9	0	other	0.5	subcutances other days	high	medium	hard	-0.643869086
121	10	0	other	2.5	mascular once a weak	high	low	hard	0.00383736
121	10	1	iran	5.5	mascular once a weak	medium	medium	easy	-0.697626621
121	11	0	other	5.5	subcutances other days	high	medium	easy	-0.34108841
121	11	1	iran	0.5	subcutances three times a weak	medium	low	hard	-0.699689278
121	12	1	iran	5.5	subcutances three times a weak	high	medium	hard	-0.787756632
121	12	0	other	2.5	subcutances other days	medium	low	hard	-0.352164436
122	1	1	iran	5.5	mascular once a weak	high	medium	hard	-0.572088286
122	1	0	iran	0.5	subcutances three times a weak	high	medium	easy	-0.72867963
122	2	0	other	0.5	subcutances other days	high	medium	hard	-0.643869086
122	2	1	iran	5.5	subcutances other days	medium	medium	hard	-0.928090082
122	3	0	iran	5.5	mascular once a weak	high	medium	hard	-0.572088286
122	3	1	iran	2.5	subcutances three times a weak	high	medium	easy	-0.646894171
122	4	1	iran	0.5	subcutances three times a weak	medium	low	hard	-0.699689278
122	4	0	other	2.5	mascular once a weak	high	low	hard	0.00383736
122	5	1	iran	0.5	mascular once a weak	medium	low	easy	-0.303092094
122	5	0	other	2.5	subcutances three times a weak	high	low	easy	-0.030902146
122	6	0	other	2.5	subcutances three times a weak	high	low	easy	-0.030902146
122	6	1	other	0.5	subcutances other days	high	medium	easy	-0.462940247
122	7	1	other	0.5	mascular once a weak	medium	medium	hard	-0.900801637
122	7	0	other	5.5	subcutances other days	medium	low	hard	-0.312098058
122	8	1	iran	2.5	mascular once a weak	high	low	hard	-0.0957683
122	8	0	iran	5.5	subcutances three times a weak	high	low	easy	-0.090441429
122	9	1	iran	2.5	subcutances three times a weak	medium	low	hard	-0.617903819
122	9	0	other	0.5	subcutances other days	high	medium	hard	-0.643869086

122	10	0	other	2.5	mascular once a weak	high	low	hard	0.00383736
122	10	1	iran	5.5	mascular once a weak	medium	medium	easy	-0.697626621
122	11	0	other	5.5	subcutances other days	high	medium	easy	-0.34108841
122	11	1	iran	0.5	subcutances three times a weak	medium	low	hard	-0.699689278
122	12	0	iran	5.5	subcutances three times a weak	high	medium	hard	-0.787756632
122	12	1	other	2.5	subcutances other days	medium	low	hard	-0.352164436
123	1	0	iran	5.5	mascular once a weak	high	medium	hard	-0.572088286
123	1	1	iran	0.5	subcutances three times a weak	high	medium	easy	-0.72867963
123	2	1	other	0.5	subcutances other days	high	medium	hard	-0.643869086
123	2	0	iran	5.5	subcutances other days	medium	medium	hard	-0.928090082
123	3	0	iran	5.5	mascular once a weak	high	medium	hard	-0.572088286
123	3	1	iran	2.5	subcutances three times a weak	high	medium	easy	-0.646894171
123	4	0	iran	0.5	subcutances three times a weak	medium	low	hard	-0.699689278
123	4	1	other	2.5	mascular once a weak	high	low	hard	0.00383736
123	5	0	iran	0.5	mascular once a weak	medium	low	easy	-0.303092094
123	5	1	other	2.5	subcutances three times a weak	high	low	easy	-0.030902146
123	6	1	other	2.5	subcutances three times a weak	high	low	easy	-0.030902146
123	6	0	other	0.5	subcutances other days	high	medium	easy	-0.462940247
123	7	0	other	0.5	mascular once a weak	medium	medium	hard	-0.900801637
123	7	1	other	5.5	subcutances other days	medium	low	hard	-0.312098058
123	8	0	iran	2.5	mascular once a weak	high	low	hard	-0.0957683
123	8	1	iran	5.5	subcutances three times a weak	high	low	easy	-0.090441429
123	9	0	iran	2.5	subcutances three times a weak	medium	low	hard	-0.617903819
123	9	1	other	0.5	subcutances other days	high	medium	hard	-0.643869086
123	10	1	other	2.5	mascular once a weak	high	low	hard	0.00383736
123	10	0	iran	5.5	mascular once a weak	medium	medium	easy	-0.697626621
123	11	1	other	5.5	subcutances other days	high	medium	easy	-0.34108841
123	11	0	iran	0.5	subcutances three times a weak	medium	low	hard	-0.699689278
123	12	1	iran	5.5	subcutances three times a weak	high	medium	hard	-0.787756632
123	12	0	other	2.5	subcutances other days	medium	low	hard	-0.352164436
124	1	0	iran	5.5	mascular once a weak	high	medium	hard	-0.572088286
124	1	0	iran	0.5	subcutances three times a weak	high	medium	easy	-0.72867963
124	2	1	other	0.5	subcutances other days	high	medium	hard	-0.643869086
124	2	0	iran	5.5	subcutances other days	medium	medium	hard	-0.928090082

124	3	0	iran	5.5	mascular once a weak	high	medium	hard	-0.572088286
124	3	1	iran	2.5	subcutances three times a weak	high	medium	easy	-0.646894171
124	4	0	iran	0.5	subcutances three times a weak	medium	low	hard	-0.699689278
124	4	1	other	2.5	mascular once a weak	high	low	hard	0.00383736
124	5	0	iran	0.5	mascular once a weak	medium	low	easy	-0.303092094
124	5	1	other	2.5	subcutances three times a weak	high	low	easy	-0.030902146
124	6	1	other	2.5	subcutances three times a weak	high	low	easy	-0.030902146
124	6	0	other	0.5	subcutances other days	high	medium	easy	-0.462940247
124	7	0	other	0.5	mascular once a weak	medium	medium	hard	-0.900801637
124	7	1	other	5.5	subcutances other days	medium	low	hard	-0.312098058
124	8	0	iran	2.5	mascular once a weak	high	low	hard	-0.0957683
124	8	1	iran	5.5	subcutances three times a weak	high	low	easy	-0.090441429
124	9	1	iran	2.5	subcutances three times a weak	medium	low	hard	-0.617903819
124	9	0	other	0.5	subcutances other days	high	medium	hard	-0.643869086
124	10	0	other	2.5	mascular once a weak	high	low	hard	0.00383736
124	10	1	iran	5.5	mascular once a weak	medium	medium	easy	-0.697626621
124	11	1	other	5.5	subcutances other days	high	medium	easy	-0.34108841
124	11	0	iran	0.5	subcutances three times a weak	medium	low	hard	-0.699689278
124	12	0	iran	5.5	subcutances three times a weak	high	medium	hard	-0.787756632
124	12	1	other	2.5	subcutances other days	medium	low	hard	-0.352164436
125	1	0	iran	5.5	mascular once a weak	high	medium	hard	-0.572088286
125	1	1	iran	0.5	subcutances three times a weak	high	medium	easy	-0.72867963
125	2	1	other	0.5	subcutances other days	high	medium	hard	-0.643869086
125	2	0	iran	5.5	subcutances other days	medium	medium	hard	-0.928090082
125	3	0	iran	5.5	mascular once a weak	high	medium	hard	-0.572088286
125	3	1	iran	2.5	subcutances three times a weak	high	medium	easy	-0.646894171
125	4	0	iran	0.5	subcutances three times a weak	medium	low	hard	-0.699689278
125	4	1	other	2.5	mascular once a weak	high	low	hard	0.00383736
125	5	0	iran	0.5	mascular once a weak	medium	low	easy	-0.303092094
125	5	1	other	2.5	subcutances three times a weak	high	low	easy	-0.030902146
125	6	1	other	2.5	subcutances three times a weak	high	low	easy	-0.030902146
125	6	0	other	0.5	subcutances other days	high	medium	easy	-0.462940247
125	7	0	other	0.5	mascular once a weak	medium	medium	hard	-0.900801637
125	7	1	other	5.5	subcutances other days	medium	low	hard	-0.312098058

125	8	0	iran	2.5	mascular once a weak	high	low	hard	-0.0957683
125	8	1	iran	5.5	subcutances three times a weak	high	low	easy	-0.090441429
125	9	0	iran	2.5	subcutances three times a weak	medium	low	hard	-0.617903819
125	9	1	other	0.5	subcutances other days	high	medium	hard	-0.643869086
125	10	1	other	2.5	mascular once a weak	high	low	hard	0.00383736
125	10	0	iran	5.5	mascular once a weak	medium	medium	easy	-0.697626621
125	11	1	other	5.5	subcutances other days	high	medium	easy	-0.34108841
125	11	0	iran	0.5	subcutances three times a weak	medium	low	hard	-0.699689278
125	12	1	iran	5.5	subcutances three times a weak	high	medium	hard	-0.787756632
125	12	0	other	2.5	subcutances other days	medium	low	hard	-0.352164436
126	1	1	iran	5.5	mascular once a weak	high	medium	hard	-0.572088286
126	1	0	iran	0.5	subcutances three times a weak	high	medium	easy	-0.72867963
126	2	0	other	0.5	subcutances other days	high	medium	hard	-0.643869086
126	2	1	iran	5.5	subcutances other days	medium	medium	hard	-0.928090082
126	3	0	iran	5.5	mascular once a weak	high	medium	hard	-0.572088286
126	3	1	iran	2.5	subcutances three times a weak	high	medium	easy	-0.646894171
126	4	1	iran	0.5	subcutances three times a weak	medium	low	hard	-0.699689278
126	4	0	other	2.5	mascular once a weak	high	low	hard	0.00383736
126	5	1	iran	0.5	mascular once a weak	medium	low	easy	-0.303092094
126	5	0	other	2.5	subcutances three times a weak	high	low	easy	-0.030902146
126	6	0	other	2.5	subcutances three times a weak	high	low	easy	-0.030902146
126	6	1	other	0.5	subcutances other days	high	medium	easy	-0.462940247
126	7	0	other	0.5	mascular once a weak	medium	medium	hard	-0.900801637
126	7	0	other	5.5	subcutances other days	medium	low	hard	-0.312098058
126	8	1	iran	2.5	mascular once a weak	high	low	hard	-0.0957683
126	8	0	iran	5.5	subcutances three times a weak	high	low	easy	-0.090441429
126	9	1	iran	2.5	subcutances three times a weak	medium	low	hard	-0.617903819
126	9	0	other	0.5	subcutances other days	high	medium	hard	-0.643869086
126	10	0	other	2.5	mascular once a weak	high	low	hard	0.00383736
126	10	1	iran	5.5	mascular once a weak	medium	medium	easy	-0.697626621
126	11	0	other	5.5	subcutances other days	high	medium	easy	-0.34108841
126	11	1	iran	0.5	subcutances three times a weak	medium	low	hard	-0.699689278
126	12	0	iran	5.5	subcutances three times a weak	high	medium	hard	-0.787756632
126	12	1	other	2.5	subcutances other days	medium	low	hard	-0.352164436

127	1	1	iran	5.5	mascular once a weak	high	medium	hard	-0.572088286
127	1	0	iran	0.5	subcutances three times a weak	high	medium	easy	-0.72867963
127	2	0	other	0.5	subcutances other days	high	medium	hard	-0.643869086
127	2	1	iran	5.5	subcutances other days	medium	medium	hard	-0.928090082
127	3	0	iran	5.5	mascular once a weak	high	medium	hard	-0.572088286
127	3	1	iran	2.5	subcutances three times a weak	high	medium	easy	-0.646894171
127	4	1	iran	0.5	subcutances three times a weak	medium	low	hard	-0.699689278
127	4	0	other	2.5	mascular once a weak	high	low	hard	0.00383736
127	5	1	iran	0.5	mascular once a weak	medium	low	easy	-0.303092094
127	5	0	other	2.5	subcutances three times a weak	high	low	easy	-0.030902146
127	6	0	other	2.5	subcutances three times a weak	high	low	easy	-0.030902146
127	6	1	other	0.5	subcutances other days	high	medium	easy	-0.462940247
127	7	0	other	0.5	mascular once a weak	medium	medium	hard	-0.900801637
127	7	1	other	5.5	subcutances other days	medium	low	hard	-0.312098058
127	8	1	iran	2.5	mascular once a weak	high	low	hard	-0.0957683
127	8	0	iran	5.5	subcutances three times a weak	high	low	easy	-0.090441429
127	9	1	iran	2.5	subcutances three times a weak	medium	low	hard	-0.617903819
127	9	0	other	0.5	subcutances other days	high	medium	hard	-0.643869086
127	10	0	other	2.5	mascular once a weak	high	low	hard	0.00383736
127	10	1	iran	5.5	mascular once a weak	medium	medium	easy	-0.697626621
127	11	0	other	5.5	subcutances other days	high	medium	easy	-0.34108841
127	11	1	iran	0.5	subcutances three times a weak	medium	low	hard	-0.699689278
127	12	0	iran	5.5	subcutances three times a weak	high	medium	hard	-0.787756632
127	12	1	other	2.5	subcutances other days	medium	low	hard	-0.352164436
128	1	1	iran	5.5	mascular once a weak	high	medium	hard	-0.572088286
128	1	0	iran	0.5	subcutances three times a weak	high	medium	easy	-0.72867963
128	2	0	other	0.5	subcutances other days	high	medium	hard	-0.643869086
128	2	1	iran	5.5	subcutances other days	medium	medium	hard	-0.928090082
128	3	0	iran	5.5	mascular once a weak	high	medium	hard	-0.572088286
128	3	1	iran	2.5	subcutances three times a weak	high	medium	easy	-0.646894171
128	4	1	iran	0.5	subcutances three times a weak	medium	low	hard	-0.699689278
128	4	0	other	2.5	mascular once a weak	high	low	hard	0.00383736
128	5	1	iran	0.5	mascular once a weak	medium	low	easy	-0.303092094
128	5	0	other	2.5	subcutances three times a weak	high	low	easy	-0.030902146

128	6	0	other	2.5	subcutances three times a weak	high	low	easy	-0.030902146
128	6	1	other	0.5	subcutances other days	high	medium	easy	-0.462940247
128	7	0	other	0.5	mascular once a weak	medium	medium	hard	-0.900801637
128	7	0	other	5.5	subcutances other days	medium	low	hard	-0.312098058
128	8	1	iran	2.5	mascular once a weak	high	low	hard	-0.0957683
128	8	0	iran	5.5	subcutances three times a weak	high	low	easy	-0.090441429
128	9	1	iran	2.5	subcutances three times a weak	medium	low	hard	-0.617903819
128	9	0	other	0.5	subcutances other days	high	medium	hard	-0.643869086
128	10	0	other	2.5	mascular once a weak	high	low	hard	0.00383736
128	10	1	iran	5.5	mascular once a weak	medium	medium	easy	-0.697626621
128	11	0	other	5.5	subcutances other days	high	medium	easy	-0.34108841
128	11	1	iran	0.5	subcutances three times a weak	medium	low	hard	-0.699689278
128	12	0	iran	5.5	subcutances three times a weak	high	medium	hard	-0.787756632
128	12	1	other	2.5	subcutances other days	medium	low	hard	-0.352164436
129	1	1	iran	5.5	mascular once a weak	high	medium	hard	-0.572088286
129	1	0	iran	0.5	subcutances three times a weak	high	medium	easy	-0.72867963
129	2	0	other	0.5	subcutances other days	high	medium	hard	-0.643869086
129	2	1	iran	5.5	subcutances other days	medium	medium	hard	-0.928090082
129	3	1	iran	5.5	mascular once a weak	high	medium	hard	-0.572088286
129	3	0	iran	2.5	subcutances three times a weak	high	medium	easy	-0.646894171
129	4	0	iran	0.5	subcutances three times a weak	medium	low	hard	-0.699689278
129	4	1	other	2.5	mascular once a weak	high	low	hard	0.00383736
129	5	1	iran	0.5	mascular once a weak	medium	low	easy	-0.303092094
129	5	0	other	2.5	subcutances three times a weak	high	low	easy	-0.030902146
129	6	0	other	2.5	subcutances three times a weak	high	low	easy	-0.030902146
129	6	1	other	0.5	subcutances other days	high	medium	easy	-0.462940247
129	7	1	other	0.5	mascular once a weak	medium	medium	hard	-0.900801637
129	7	0	other	5.5	subcutances other days	medium	low	hard	-0.312098058
129	8	1	iran	2.5	mascular once a weak	high	low	hard	-0.0957683
129	8	0	iran	5.5	subcutances three times a weak	high	low	easy	-0.090441429
129	9	1	iran	2.5	subcutances three times a weak	medium	low	hard	-0.617903819
129	9	0	other	0.5	subcutances other days	high	medium	hard	-0.643869086
129	10	1	other	2.5	mascular once a weak	high	low	hard	0.00383736
129	10	0	iran	5.5	mascular once a weak	medium	medium	easy	-0.697626621

129	11	1	other	5.5	subcutances other days	high	medium	easy	-0.34108841
129	11	0	iran	0.5	subcutances three times a weak	medium	low	hard	-0.699689278
129	12	0	iran	5.5	subcutances three times a weak	high	medium	hard	-0.787756632
129	12	1	other	2.5	subcutances other days	medium	low	hard	-0.352164436
130	1	1	iran	5.5	mascular once a weak	high	medium	hard	-0.572088286
130	1	0	iran	0.5	subcutances three times a weak	high	medium	easy	-0.72867963
130	2	1	other	0.5	subcutances other days	high	medium	hard	-0.643869086
130	2	0	iran	5.5	subcutances other days	medium	medium	hard	-0.928090082
130	3	0	iran	5.5	mascular once a weak	high	medium	hard	-0.572088286
130	3	1	iran	2.5	subcutances three times a weak	high	medium	easy	-0.646894171
130	4	0	iran	0.5	subcutances three times a weak	medium	low	hard	-0.699689278
130	4	1	other	2.5	mascular once a weak	high	low	hard	0.00383736
130	5	1	iran	0.5	mascular once a weak	medium	low	easy	-0.303092094
130	5	0	other	2.5	subcutances three times a weak	high	low	easy	-0.030902146
130	6	1	other	2.5	subcutances three times a weak	high	low	easy	-0.030902146
130	6	0	other	0.5	subcutances other days	high	medium	easy	-0.462940247
130	7	0	other	0.5	mascular once a weak	medium	medium	hard	-0.900801637
130	7	1	other	5.5	subcutances other days	medium	low	hard	-0.312098058
130	8	1	iran	2.5	mascular once a weak	high	low	hard	-0.0957683
130	8	0	iran	5.5	subcutances three times a weak	high	low	easy	-0.090441429
130	9	0	iran	2.5	subcutances three times a weak	medium	low	hard	-0.617903819
130	9	1	other	0.5	subcutances other days	high	medium	hard	-0.643869086
130	10	1	other	2.5	mascular once a weak	high	low	hard	0.00383736
130	10	0	iran	5.5	mascular once a weak	medium	medium	easy	-0.697626621
130	11	1	other	5.5	subcutances other days	high	medium	easy	-0.34108841
130	11	0	iran	0.5	subcutances three times a weak	medium	low	hard	-0.699689278
130	12	0	iran	5.5	subcutances three times a weak	high	medium	hard	-0.787756632
130	12	1	other	2.5	subcutances other days	medium	low	hard	-0.352164436
131	1	0	iran	5.5	mascular once a weak	high	medium	hard	-0.572088286
131	1	1	iran	0.5	subcutances three times a weak	high	medium	easy	-0.72867963
131	2	0	other	0.5	subcutances other days	high	medium	hard	-0.643869086
131	2	1	iran	5.5	subcutances other days	medium	medium	hard	-0.928090082
131	3	0	iran	5.5	mascular once a weak	high	medium	hard	-0.572088286
131	3	1	iran	2.5	subcutances three times a weak	high	medium	easy	-0.646894171

131	4	1	iran	0.5	subcutances three times a weak	medium	low	hard	-0.699689278
131	4	0	other	2.5	mascular once a weak	high	low	hard	0.00383736
131	5	0	iran	0.5	mascular once a weak	medium	low	easy	-0.303092094
131	5	1	other	2.5	subcutances three times a weak	high	low	easy	-0.030902146
131	6	0	other	2.5	subcutances three times a weak	high	low	easy	-0.030902146
131	6	1	other	0.5	subcutances other days	high	medium	easy	-0.462940247
131	7	0	other	0.5	mascular once a weak	medium	medium	hard	-0.900801637
131	7	1	other	5.5	subcutances other days	medium	low	hard	-0.312098058
131	8	1	iran	2.5	mascular once a weak	high	low	hard	-0.0957683
131	8	0	iran	5.5	subcutances three times a weak	high	low	easy	-0.090441429
131	9	1	iran	2.5	subcutances three times a weak	medium	low	hard	-0.617903819
131	9	0	other	0.5	subcutances other days	high	medium	hard	-0.643869086
131	10	0	other	2.5	mascular once a weak	high	low	hard	0.00383736
131	10	1	iran	5.5	mascular once a weak	medium	medium	easy	-0.697626621
131	11	0	other	5.5	subcutances other days	high	medium	easy	-0.34108841
131	11	1	iran	0.5	subcutances three times a weak	medium	low	hard	-0.699689278
131	12	0	iran	5.5	subcutances three times a weak	high	medium	hard	-0.787756632
131	12	1	other	2.5	subcutances other days	medium	low	hard	-0.352164436
132	1	1	iran	5.5	mascular once a weak	high	medium	hard	-0.572088286
132	1	0	iran	0.5	subcutances three times a weak	high	medium	easy	-0.72867963
132	2	1	other	0.5	subcutances other days	high	medium	hard	-0.643869086
132	2	0	iran	5.5	subcutances other days	medium	medium	hard	-0.928090082
132	3	1	iran	5.5	mascular once a weak	high	medium	hard	-0.572088286
132	3	0	iran	2.5	subcutances three times a weak	high	medium	easy	-0.646894171
132	4	0	iran	0.5	subcutances three times a weak	medium	low	hard	-0.699689278
132	4	1	other	2.5	mascular once a weak	high	low	hard	0.00383736
132	5	0	iran	0.5	mascular once a weak	medium	low	easy	-0.303092094
132	5	1	other	2.5	subcutances three times a weak	high	low	easy	-0.030902146
132	6	1	other	2.5	subcutances three times a weak	high	low	easy	-0.030902146
132	6	0	other	0.5	subcutances other days	high	medium	easy	-0.462940247
132	7	0	other	0.5	mascular once a weak	medium	medium	hard	-0.900801637
132	7	1	other	5.5	subcutances other days	medium	low	hard	-0.312098058
132	8	1	iran	2.5	mascular once a weak	high	low	hard	-0.0957683
132	8	0	iran	5.5	subcutances three times a weak	high	low	easy	-0.090441429

132	9	0	iran	2.5	subcutances three times a weak	medium	low	hard	-0.617903819
132	9	1	other	0.5	subcutances other days	high	medium	hard	-0.643869086
132	10	1	other	2.5	mascular once a weak	high	low	hard	0.00383736
132	10	0	iran	5.5	mascular once a weak	medium	medium	easy	-0.697626621
132	11	1	other	5.5	subcutances other days	high	medium	easy	-0.34108841
132	11	0	iran	0.5	subcutances three times a weak	medium	low	hard	-0.699689278
132	12	1	iran	5.5	subcutances three times a weak	high	medium	hard	-0.787756632
132	12	0	other	2.5	subcutances other days	medium	low	hard	-0.352164436
133	1	1	iran	5.5	mascular once a weak	high	medium	hard	-0.572088286
133	1	0	iran	0.5	subcutances three times a weak	high	medium	easy	-0.72867963
133	2	1	other	0.5	subcutances other days	high	medium	hard	-0.643869086
133	2	0	iran	5.5	subcutances other days	medium	medium	hard	-0.928090082
133	3	1	iran	5.5	mascular once a weak	high	medium	hard	-0.572088286
133	3	0	iran	2.5	subcutances three times a weak	high	medium	easy	-0.646894171
133	4	0	iran	0.5	subcutances three times a weak	medium	low	hard	-0.699689278
133	4	1	other	2.5	mascular once a weak	high	low	hard	0.00383736
133	5	0	iran	0.5	mascular once a weak	medium	low	easy	-0.303092094
133	5	1	other	2.5	subcutances three times a weak	high	low	easy	-0.030902146
133	6	1	other	2.5	subcutances three times a weak	high	low	easy	-0.030902146
133	6	0	other	0.5	subcutances other days	high	medium	easy	-0.462940247
133	7	0	other	0.5	mascular once a weak	medium	medium	hard	-0.900801637
133	7	1	other	5.5	subcutances other days	medium	low	hard	-0.312098058
133	8	1	iran	2.5	mascular once a weak	high	low	hard	-0.0957683
133	8	0	iran	5.5	subcutances three times a weak	high	low	easy	-0.090441429
133	9	0	iran	2.5	subcutances three times a weak	medium	low	hard	-0.617903819
133	9	1	other	0.5	subcutances other days	high	medium	hard	-0.643869086
133	10	1	other	2.5	mascular once a weak	high	low	hard	0.00383736
133	10	0	iran	5.5	mascular once a weak	medium	medium	easy	-0.697626621
133	11	1	other	5.5	subcutances other days	high	medium	easy	-0.34108841
133	11	0	iran	0.5	subcutances three times a weak	medium	low	hard	-0.699689278
133	12	1	iran	5.5	subcutances three times a weak	high	medium	hard	-0.787756632
133	12	0	other	2.5	subcutances other days	medium	low	hard	-0.352164436
134	1	0	iran	5.5	mascular once a weak	high	medium	hard	-0.572088286
134	1	1	iran	0.5	subcutances three times a weak	high	medium	easy	-0.72867963

134	2	1	other	0.5	subcutances other days	high	medium	hard	-0.643869086
134	2	0	iran	5.5	subcutances other days	medium	medium	hard	-0.928090082
134	3	0	iran	5.5	mascular once a weak	high	medium	hard	-0.572088286
134	3	1	iran	2.5	subcutances three times a weak	high	medium	easy	-0.646894171
134	4	0	iran	0.5	subcutances three times a weak	medium	low	hard	-0.699689278
134	4	1	other	2.5	mascular once a weak	high	low	hard	0.00383736
134	5	0	iran	0.5	mascular once a weak	medium	low	easy	-0.303092094
134	5	1	other	2.5	subcutances three times a weak	high	low	easy	-0.030902146
134	6	1	other	2.5	subcutances three times a weak	high	low	easy	-0.030902146
134	6	0	other	0.5	subcutances other days	high	medium	easy	-0.462940247
134	7	0	other	0.5	mascular once a weak	medium	medium	hard	-0.900801637
134	7	1	other	5.5	subcutances other days	medium	low	hard	-0.312098058
134	8	0	iran	2.5	mascular once a weak	high	low	hard	-0.0957683
134	8	1	iran	5.5	subcutances three times a weak	high	low	easy	-0.090441429
134	9	1	iran	2.5	subcutances three times a weak	medium	low	hard	-0.617903819
134	9	0	other	0.5	subcutances other days	high	medium	hard	-0.643869086
134	10	0	other	2.5	mascular once a weak	high	low	hard	0.00383736
134	10	1	iran	5.5	mascular once a weak	medium	medium	easy	-0.697626621
134	11	1	other	5.5	subcutances other days	high	medium	easy	-0.34108841
134	11	0	iran	0.5	subcutances three times a weak	medium	low	hard	-0.699689278
134	12	0	iran	5.5	subcutances three times a weak	high	medium	hard	-0.787756632
134	12	1	other	2.5	subcutances other days	medium	low	hard	-0.352164436
135	1	1	iran	5.5	mascular once a weak	high	medium	hard	-0.572088286
135	1	0	iran	0.5	subcutances three times a weak	high	medium	easy	-0.72867963
135	2	0	other	0.5	subcutances other days	high	medium	hard	-0.643869086
135	2	1	iran	5.5	subcutances other days	medium	medium	hard	-0.928090082
135	3	0	iran	5.5	mascular once a weak	high	medium	hard	-0.572088286
135	3	1	iran	2.5	subcutances three times a weak	high	medium	easy	-0.646894171
135	4	1	iran	0.5	subcutances three times a weak	medium	low	hard	-0.699689278
135	4	0	other	2.5	mascular once a weak	high	low	hard	0.00383736
135	5	1	iran	0.5	mascular once a weak	medium	low	easy	-0.303092094
135	5	0	other	2.5	subcutances three times a weak	high	low	easy	-0.030902146
135	6	0	other	2.5	subcutances three times a weak	high	low	easy	-0.030902146
135	6	1	other	0.5	subcutances other days	high	medium	easy	-0.462940247

135	7	1	other	0.5	mascular once a weak	medium	medium	hard	-0.900801637
135	7	0	other	5.5	subcutances other days	medium	low	hard	-0.312098058
135	8	1	iran	2.5	mascular once a weak	high	low	hard	-0.0957683
135	8	0	iran	5.5	subcutances three times a weak	high	low	easy	-0.090441429
135	9	1	iran	2.5	subcutances three times a weak	medium	low	hard	-0.617903819
135	9	0	other	0.5	subcutances other days	high	medium	hard	-0.643869086
135	10	0	other	2.5	mascular once a weak	high	low	hard	0.00383736
135	10	1	iran	5.5	mascular once a weak	medium	medium	easy	-0.697626621
135	11	0	other	5.5	subcutances other days	high	medium	easy	-0.34108841
135	11	1	iran	0.5	subcutances three times a weak	medium	low	hard	-0.699689278
135	12	0	iran	5.5	subcutances three times a weak	high	medium	hard	-0.787756632
135	12	1	other	2.5	subcutances other days	medium	low	hard	-0.352164436
136	1	1	iran	5.5	mascular once a weak	high	medium	hard	-0.572088286
136	1	0	iran	0.5	subcutances three times a weak	high	medium	easy	-0.72867963
136	2	1	other	0.5	subcutances other days	high	medium	hard	-0.643869086
136	2	0	iran	5.5	subcutances other days	medium	medium	hard	-0.928090082
136	3	0	iran	5.5	mascular once a weak	high	medium	hard	-0.572088286
136	3	0	iran	2.5	subcutances three times a weak	high	medium	easy	-0.646894171
136	4	0	iran	0.5	subcutances three times a weak	medium	low	hard	-0.699689278
136	4	1	other	2.5	mascular once a weak	high	low	hard	0.00383736
136	5	0	iran	0.5	mascular once a weak	medium	low	easy	-0.303092094
136	5	1	other	2.5	subcutances three times a weak	high	low	easy	-0.030902146
136	6	1	other	2.5	subcutances three times a weak	high	low	easy	-0.030902146
136	6	0	other	0.5	subcutances other days	high	medium	easy	-0.462940247
136	7	1	other	0.5	mascular once a weak	medium	medium	hard	-0.900801637
136	7	0	other	5.5	subcutances other days	medium	low	hard	-0.312098058
136	8	0	iran	2.5	mascular once a weak	high	low	hard	-0.0957683
136	8	1	iran	5.5	subcutances three times a weak	high	low	easy	-0.090441429
136	9	0	iran	2.5	subcutances three times a weak	medium	low	hard	-0.617903819
136	9	1	other	0.5	subcutances other days	high	medium	hard	-0.643869086
136	10	0	other	2.5	mascular once a weak	high	low	hard	0.00383736
136	10	1	iran	5.5	mascular once a weak	medium	medium	easy	-0.697626621
136	11	1	other	5.5	subcutances other days	high	medium	easy	-0.34108841
136	11	0	iran	0.5	subcutances three times a weak	medium	low	hard	-0.699689278

136	12	0	iran	5.5	subcutances three times a weak	high	medium	hard	-0.787756632
136	12	1	other	2.5	subcutances other days	medium	low	hard	-0.352164436
137	1	0	iran	5.5	mascular once a weak	high	medium	hard	-0.572088286
137	1	1	iran	0.5	subcutances three times a weak	high	medium	easy	-0.72867963
137	2	0	other	0.5	subcutances other days	high	medium	hard	-0.643869086
137	2	1	iran	5.5	subcutances other days	medium	medium	hard	-0.928090082
137	3	0	iran	5.5	mascular once a weak	high	medium	hard	-0.572088286
137	3	1	iran	2.5	subcutances three times a weak	high	medium	easy	-0.646894171
137	4	0	iran	0.5	subcutances three times a weak	medium	low	hard	-0.699689278
137	4	1	other	2.5	mascular once a weak	high	low	hard	0.00383736
137	5	0	iran	0.5	mascular once a weak	medium	low	easy	-0.303092094
137	5	1	other	2.5	subcutances three times a weak	high	low	easy	-0.030902146
137	6	0	other	2.5	subcutances three times a weak	high	low	easy	-0.030902146
137	6	1	other	0.5	subcutances other days	high	medium	easy	-0.462940247
137	7	1	other	0.5	mascular once a weak	medium	medium	hard	-0.900801637
137	7	0	other	5.5	subcutances other days	medium	low	hard	-0.312098058
137	8	1	iran	2.5	mascular once a weak	high	low	hard	-0.0957683
137	8	0	iran	5.5	subcutances three times a weak	high	low	easy	-0.090441429
137	9	1	iran	2.5	subcutances three times a weak	medium	low	hard	-0.617903819
137	9	0	other	0.5	subcutances other days	high	medium	hard	-0.643869086
137	10	0	other	2.5	mascular once a weak	high	low	hard	0.00383736
137	10	1	iran	5.5	mascular once a weak	medium	medium	easy	-0.697626621
137	11	0	other	5.5	subcutances other days	high	medium	easy	-0.34108841
137	11	1	iran	0.5	subcutances three times a weak	medium	low	hard	-0.699689278
137	12	0	iran	5.5	subcutances three times a weak	high	medium	hard	-0.787756632
137	12	1	other	2.5	subcutances other days	medium	low	hard	-0.352164436
138	1	0	iran	5.5	mascular once a weak	high	medium	hard	-0.572088286
138	1	1	iran	0.5	subcutances three times a weak	high	medium	easy	-0.72867963
138	2	1	other	0.5	subcutances other days	high	medium	hard	-0.643869086
138	2	0	iran	5.5	subcutances other days	medium	medium	hard	-0.928090082
138	3	0	iran	5.5	mascular once a weak	high	medium	hard	-0.572088286
138	3	1	iran	2.5	subcutances three times a weak	high	medium	easy	-0.646894171
138	4	0	iran	0.5	subcutances three times a weak	medium	low	hard	-0.699689278
138	4	1	other	2.5	mascular once a weak	high	low	hard	0.00383736

138	5	0	iran	0.5	mascular once a weak	medium	low	easy	-0.303092094
138	5	1	other	2.5	subcutances three times a weak	high	low	easy	-0.030902146
138	6	1	other	2.5	subcutances three times a weak	high	low	easy	-0.030902146
138	6	0	other	0.5	subcutances other days	high	medium	easy	-0.462940247
138	7	0	other	0.5	mascular once a weak	medium	medium	hard	-0.900801637
138	7	1	other	5.5	subcutances other days	medium	low	hard	-0.312098058
138	8	1	iran	2.5	mascular once a weak	high	low	hard	-0.0957683
138	8	0	iran	5.5	subcutances three times a weak	high	low	easy	-0.090441429
138	9	0	iran	2.5	subcutances three times a weak	medium	low	hard	-0.617903819
138	9	1	other	0.5	subcutances other days	high	medium	hard	-0.643869086
138	10	1	other	2.5	mascular once a weak	high	low	hard	0.00383736
138	10	0	iran	5.5	mascular once a weak	medium	medium	easy	-0.697626621
138	11	1	other	5.5	subcutances other days	high	medium	easy	-0.34108841
138	11	0	iran	0.5	subcutances three times a weak	medium	low	hard	-0.699689278
138	12	1	iran	5.5	subcutances three times a weak	high	medium	hard	-0.787756632
138	12	0	other	2.5	subcutances other days	medium	low	hard	-0.352164436
139	1	1	iran	5.5	mascular once a weak	high	medium	hard	-0.572088286
139	1	0	iran	0.5	subcutances three times a weak	high	medium	easy	-0.72867963
139	2	1	other	0.5	subcutances other days	high	medium	hard	-0.643869086
139	2	0	iran	5.5	subcutances other days	medium	medium	hard	-0.928090082
139	3	0	iran	5.5	mascular once a weak	high	medium	hard	-0.572088286
139	3	1	iran	2.5	subcutances three times a weak	high	medium	easy	-0.646894171
139	4	0	iran	0.5	subcutances three times a weak	medium	low	hard	-0.699689278
139	4	1	other	2.5	mascular once a weak	high	low	hard	0.00383736
139	5	0	iran	0.5	mascular once a weak	medium	low	easy	-0.303092094
139	5	1	other	2.5	subcutances three times a weak	high	low	easy	-0.030902146
139	6	1	other	2.5	subcutances three times a weak	high	low	easy	-0.030902146
139	6	0	other	0.5	subcutances other days	high	medium	easy	-0.462940247
139	7	0	other	0.5	mascular once a weak	medium	medium	hard	-0.900801637
139	7	1	other	5.5	subcutances other days	medium	low	hard	-0.312098058
139	8	0	iran	2.5	mascular once a weak	high	low	hard	-0.0957683
139	8	1	iran	5.5	subcutances three times a weak	high	low	easy	-0.090441429
139	9	0	iran	2.5	subcutances three times a weak	medium	low	hard	-0.617903819
139	9	1	other	0.5	subcutances other days	high	medium	hard	-0.643869086

139	10	1	other	2.5	mascular once a weak	high	low	hard	0.00383736
139	10	0	iran	5.5	mascular once a weak	medium	medium	easy	-0.697626621
139	11	0	other	5.5	subcutances other days	high	medium	easy	-0.34108841
139	11	1	iran	0.5	subcutances three times a weak	medium	low	hard	-0.699689278
139	12	1	iran	5.5	subcutances three times a weak	high	medium	hard	-0.787756632
139	12	0	other	2.5	subcutances other days	medium	low	hard	-0.352164436
140	1	0	iran	5.5	mascular once a weak	high	medium	hard	-0.572088286
140	1	1	iran	0.5	subcutances three times a weak	high	medium	easy	-0.72867963
140	2	1	other	0.5	subcutances other days	high	medium	hard	-0.643869086
140	2	0	iran	5.5	subcutances other days	medium	medium	hard	-0.928090082
140	3	0	iran	5.5	mascular once a weak	high	medium	hard	-0.572088286
140	3	1	iran	2.5	subcutances three times a weak	high	medium	easy	-0.646894171
140	4	0	iran	0.5	subcutances three times a weak	medium	low	hard	-0.699689278
140	4	1	other	2.5	mascular once a weak	high	low	hard	0.00383736
140	5	0	iran	0.5	mascular once a weak	medium	low	easy	-0.303092094
140	5	1	other	2.5	subcutances three times a weak	high	low	easy	-0.030902146
140	6	1	other	2.5	subcutances three times a weak	high	low	easy	-0.030902146
140	6	0	other	0.5	subcutances other days	high	medium	easy	-0.462940247
140	7	0	other	0.5	mascular once a weak	medium	medium	hard	-0.900801637
140	7	1	other	5.5	subcutances other days	medium	low	hard	-0.312098058
140	8	0	iran	2.5	mascular once a weak	high	low	hard	-0.0957683
140	8	1	iran	5.5	subcutances three times a weak	high	low	easy	-0.090441429
140	9	0	iran	2.5	subcutances three times a weak	medium	low	hard	-0.617903819
140	9	1	other	0.5	subcutances other days	high	medium	hard	-0.643869086
140	10	1	other	2.5	mascular once a weak	high	low	hard	0.00383736
140	10	0	iran	5.5	mascular once a weak	medium	medium	easy	-0.697626621
140	11	1	other	5.5	subcutances other days	high	medium	easy	-0.34108841
140	11	0	iran	0.5	subcutances three times a weak	medium	low	hard	-0.699689278
140	12	1	iran	5.5	subcutances three times a weak	high	medium	hard	-0.787756632
140	12	0	other	2.5	subcutances other days	medium	low	hard	-0.352164436
141	1	1	iran	5.5	mascular once a weak	high	medium	hard	-0.572088286
141	1	0	iran	0.5	subcutances three times a weak	high	medium	easy	-0.72867963
141	2	0	other	0.5	subcutances other days	high	medium	hard	-0.643869086
141	2	1	iran	5.5	subcutances other days	medium	medium	hard	-0.928090082

141	3	0	iran	5.5	mascular once a weak	high	medium	hard	-0.572088286
141	3	1	iran	2.5	subcutances three times a weak	high	medium	easy	-0.646894171
141	4	1	iran	0.5	subcutances three times a weak	medium	low	hard	-0.699689278
141	4	0	other	2.5	mascular once a weak	high	low	hard	0.00383736
141	5	1	iran	0.5	mascular once a weak	medium	low	easy	-0.303092094
141	5	0	other	2.5	subcutances three times a weak	high	low	easy	-0.030902146
141	6	0	other	2.5	subcutances three times a weak	high	low	easy	-0.030902146
141	6	1	other	0.5	subcutances other days	high	medium	easy	-0.462940247
141	7	0	other	0.5	mascular once a weak	medium	medium	hard	-0.900801637
141	7	1	other	5.5	subcutances other days	medium	low	hard	-0.312098058
141	8	1	iran	2.5	mascular once a weak	high	low	hard	-0.0957683
141	8	0	iran	5.5	subcutances three times a weak	high	low	easy	-0.090441429
141	9	1	iran	2.5	subcutances three times a weak	medium	low	hard	-0.617903819
141	9	0	other	0.5	subcutances other days	high	medium	hard	-0.643869086
141	10	0	other	2.5	mascular once a weak	high	low	hard	0.00383736
141	10	1	iran	5.5	mascular once a weak	medium	medium	easy	-0.697626621
141	11	0	other	5.5	subcutances other days	high	medium	easy	-0.34108841
141	11	1	iran	0.5	subcutances three times a weak	medium	low	hard	-0.699689278
141	12	0	iran	5.5	subcutances three times a weak	high	medium	hard	-0.787756632
141	12	1	other	2.5	subcutances other days	medium	low	hard	-0.352164436
142	1	1	iran	5.5	mascular once a weak	high	medium	hard	-0.572088286
142	1	0	iran	0.5	subcutances three times a weak	high	medium	easy	-0.72867963
142	2	1	other	0.5	subcutances other days	high	medium	hard	-0.643869086
142	2	0	iran	5.5	subcutances other days	medium	medium	hard	-0.928090082
142	3	1	iran	5.5	mascular once a weak	high	medium	hard	-0.572088286
142	3	0	iran	2.5	subcutances three times a weak	high	medium	easy	-0.646894171
142	4	0	iran	0.5	subcutances three times a weak	medium	low	hard	-0.699689278
142	4	1	other	2.5	mascular once a weak	high	low	hard	0.00383736
142	5	1	iran	0.5	mascular once a weak	medium	low	easy	-0.303092094
142	5	0	other	2.5	subcutances three times a weak	high	low	easy	-0.030902146
142	6	1	other	2.5	subcutances three times a weak	high	low	easy	-0.030902146
142	6	0	other	0.5	subcutances other days	high	medium	easy	-0.462940247
142	7	1	other	0.5	mascular once a weak	medium	medium	hard	-0.900801637
142	7	0	other	5.5	subcutances other days	medium	low	hard	-0.312098058

142	8	1	iran	2.5	mascular once a weak	high	low	hard	-0.0957683
142	8	0	iran	5.5	subcutances three times a weak	high	low	easy	-0.090441429
142	9	0	iran	2.5	subcutances three times a weak	medium	low	hard	-0.617903819
142	9	1	other	0.5	subcutances other days	high	medium	hard	-0.643869086
142	10	1	other	2.5	mascular once a weak	high	low	hard	0.00383736
142	10	0	iran	5.5	mascular once a weak	medium	medium	easy	-0.697626621
142	11	1	other	5.5	subcutances other days	high	medium	easy	-0.34108841
142	11	0	iran	0.5	subcutances three times a weak	medium	low	hard	-0.699689278
142	12	1	iran	5.5	subcutances three times a weak	high	medium	hard	-0.787756632
142	12	0	other	2.5	subcutances other days	medium	low	hard	-0.352164436
143	1	0	iran	5.5	mascular once a weak	high	medium	hard	-0.572088286
143	1	1	iran	0.5	subcutances three times a weak	high	medium	easy	-0.72867963
143	2	1	other	0.5	subcutances other days	high	medium	hard	-0.643869086
143	2	0	iran	5.5	subcutances other days	medium	medium	hard	-0.928090082
143	3	1	iran	5.5	mascular once a weak	high	medium	hard	-0.572088286
143	3	0	iran	2.5	subcutances three times a weak	high	medium	easy	-0.646894171
143	4	1	iran	0.5	subcutances three times a weak	medium	low	hard	-0.699689278
143	4	0	other	2.5	mascular once a weak	high	low	hard	0.00383736
143	5	0	iran	0.5	mascular once a weak	medium	low	easy	-0.303092094
143	5	1	other	2.5	subcutances three times a weak	high	low	easy	-0.030902146
143	6	1	other	2.5	subcutances three times a weak	high	low	easy	-0.030902146
143	6	0	other	0.5	subcutances other days	high	medium	easy	-0.462940247
143	7	0	other	0.5	mascular once a weak	medium	medium	hard	-0.900801637
143	7	1	other	5.5	subcutances other days	medium	low	hard	-0.312098058
143	8	1	iran	2.5	mascular once a weak	high	low	hard	-0.0957683
143	8	0	iran	5.5	subcutances three times a weak	high	low	easy	-0.090441429
143	9	0	iran	2.5	subcutances three times a weak	medium	low	hard	-0.617903819
143	9	1	other	0.5	subcutances other days	high	medium	hard	-0.643869086
143	10	1	other	2.5	mascular once a weak	high	low	hard	0.00383736
143	10	0	iran	5.5	mascular once a weak	medium	medium	easy	-0.697626621
143	11	1	other	5.5	subcutances other days	high	medium	easy	-0.34108841
143	11	0	iran	0.5	subcutances three times a weak	medium	low	hard	-0.699689278
143	12	1	iran	5.5	subcutances three times a weak	high	medium	hard	-0.787756632
143	12	0	other	2.5	subcutances other days	medium	low	hard	-0.352164436

144	1	1	iran	5.5	mascular once a weak	high	medium	hard	-0.572088286
144	1	0	iran	0.5	subcutances three times a weak	high	medium	easy	-0.72867963
144	2	0	other	0.5	subcutances other days	high	medium	hard	-0.643869086
144	2	1	iran	5.5	subcutances other days	medium	medium	hard	-0.928090082
144	3	0	iran	5.5	mascular once a weak	high	medium	hard	-0.572088286
144	3	1	iran	2.5	subcutances three times a weak	high	medium	easy	-0.646894171
144	4	0	iran	0.5	subcutances three times a weak	medium	low	hard	-0.699689278
144	4	1	other	2.5	mascular once a weak	high	low	hard	0.00383736
144	5	0	iran	0.5	mascular once a weak	medium	low	easy	-0.303092094
144	5	1	other	2.5	subcutances three times a weak	high	low	easy	-0.030902146
144	6	0	other	2.5	subcutances three times a weak	high	low	easy	-0.030902146
144	6	1	other	0.5	subcutances other days	high	medium	easy	-0.462940247
144	7	0	other	0.5	mascular once a weak	medium	medium	hard	-0.900801637
144	7	1	other	5.5	subcutances other days	medium	low	hard	-0.312098058
144	8	1	iran	2.5	mascular once a weak	high	low	hard	-0.0957683
144	8	0	iran	5.5	subcutances three times a weak	high	low	easy	-0.090441429
144	9	0	iran	2.5	subcutances three times a weak	medium	low	hard	-0.617903819
144	9	1	other	0.5	subcutances other days	high	medium	hard	-0.643869086
144	10	0	other	2.5	mascular once a weak	high	low	hard	0.00383736
144	10	1	iran	5.5	mascular once a weak	medium	medium	easy	-0.697626621
144	11	0	other	5.5	subcutances other days	high	medium	easy	-0.34108841
144	11	1	iran	0.5	subcutances three times a weak	medium	low	hard	-0.699689278
144	12	0	iran	5.5	subcutances three times a weak	high	medium	hard	-0.787756632
144	12	1	other	2.5	subcutances other days	medium	low	hard	-0.352164436
145	1	1	iran	5.5	mascular once a weak	high	medium	hard	-0.572088286
145	1	0	iran	0.5	subcutances three times a weak	high	medium	easy	-0.72867963
145	2	1	other	0.5	subcutances other days	high	medium	hard	-0.643869086
145	2	0	iran	5.5	subcutances other days	medium	medium	hard	-0.928090082
145	3	0	iran	5.5	mascular once a weak	high	medium	hard	-0.572088286
145	3	1	iran	2.5	subcutances three times a weak	high	medium	easy	-0.646894171
145	4	0	iran	0.5	subcutances three times a weak	medium	low	hard	-0.699689278
145	4	1	other	2.5	mascular once a weak	high	low	hard	0.00383736
145	5	0	iran	0.5	mascular once a weak	medium	low	easy	-0.303092094
145	5	1	other	2.5	subcutances three times a weak	high	low	easy	-0.030902146

145	6	1	other	2.5	subcutances three times a weak	high	low	easy	-0.030902146
145	6	0	other	0.5	subcutances other days	high	medium	easy	-0.462940247
145	7	0	other	0.5	mascular once a weak	medium	medium	hard	-0.900801637
145	7	1	other	5.5	subcutances other days	medium	low	hard	-0.312098058
145	8	0	iran	2.5	mascular once a weak	high	low	hard	-0.0957683
145	8	1	iran	5.5	subcutances three times a weak	high	low	easy	-0.090441429
145	9	1	iran	2.5	subcutances three times a weak	medium	low	hard	-0.617903819
145	9	0	other	0.5	subcutances other days	high	medium	hard	-0.643869086
145	10	0	other	2.5	mascular once a weak	high	low	hard	0.00383736
145	10	1	iran	5.5	mascular once a weak	medium	medium	easy	-0.697626621
145	11	1	other	5.5	subcutances other days	high	medium	easy	-0.34108841
145	11	0	iran	0.5	subcutances three times a weak	medium	low	hard	-0.699689278
145	12	0	iran	5.5	subcutances three times a weak	high	medium	hard	-0.787756632
145	12	1	other	2.5	subcutances other days	medium	low	hard	-0.352164436
146	1	1	iran	5.5	mascular once a weak	high	medium	hard	-0.572088286
146	1	0	iran	0.5	subcutances three times a weak	high	medium	easy	-0.72867963
146	2	0	other	0.5	subcutances other days	high	medium	hard	-0.643869086
146	2	1	iran	5.5	subcutances other days	medium	medium	hard	-0.928090082
146	3	0	iran	5.5	mascular once a weak	high	medium	hard	-0.572088286
146	3	1	iran	2.5	subcutances three times a weak	high	medium	easy	-0.646894171
146	4	1	iran	0.5	subcutances three times a weak	medium	low	hard	-0.699689278
146	4	0	other	2.5	mascular once a weak	high	low	hard	0.00383736
146	5	1	iran	0.5	mascular once a weak	medium	low	easy	-0.303092094
146	5	0	other	2.5	subcutances three times a weak	high	low	easy	-0.030902146
146	6	0	other	2.5	subcutances three times a weak	high	low	easy	-0.030902146
146	6	1	other	0.5	subcutances other days	high	medium	easy	-0.462940247
146	7	0	other	0.5	mascular once a weak	medium	medium	hard	-0.900801637
146	7	1	other	5.5	subcutances other days	medium	low	hard	-0.312098058
146	8	1	iran	2.5	mascular once a weak	high	low	hard	-0.0957683
146	8	0	iran	5.5	subcutances three times a weak	high	low	easy	-0.090441429
146	9	1	iran	2.5	subcutances three times a weak	medium	low	hard	-0.617903819
146	9	0	other	0.5	subcutances other days	high	medium	hard	-0.643869086
146	10	0	other	2.5	mascular once a weak	high	low	hard	0.00383736
146	10	1	iran	5.5	mascular once a weak	medium	medium	easy	-0.697626621

146	11	0	other	5.5	subcutances other days	high	medium	easy	-0.34108841
146	11	1	iran	0.5	subcutances three times a weak	medium	low	hard	-0.699689278
146	12	0	iran	5.5	subcutances three times a weak	high	medium	hard	-0.787756632
146	12	1	other	2.5	subcutances other days	medium	low	hard	-0.352164436
147	1	1	iran	5.5	mascular once a weak	high	medium	hard	-0.572088286
147	1	0	iran	0.5	subcutances three times a weak	high	medium	easy	-0.72867963
147	2	0	other	0.5	subcutances other days	high	medium	hard	-0.643869086
147	2	1	iran	5.5	subcutances other days	medium	medium	hard	-0.928090082
147	3	0	iran	5.5	mascular once a weak	high	medium	hard	-0.572088286
147	3	1	iran	2.5	subcutances three times a weak	high	medium	easy	-0.646894171
147	4	0	iran	0.5	subcutances three times a weak	medium	low	hard	-0.699689278
147	4	1	other	2.5	mascular once a weak	high	low	hard	0.00383736
147	5	0	iran	0.5	mascular once a weak	medium	low	easy	-0.303092094
147	5	1	other	2.5	subcutances three times a weak	high	low	easy	-0.030902146
147	6	0	other	2.5	subcutances three times a weak	high	low	easy	-0.030902146
147	6	1	other	0.5	subcutances other days	high	medium	easy	-0.462940247
147	7	0	other	0.5	mascular once a weak	medium	medium	hard	-0.900801637
147	7	1	other	5.5	subcutances other days	medium	low	hard	-0.312098058
147	8	1	iran	2.5	mascular once a weak	high	low	hard	-0.0957683
147	8	0	iran	5.5	subcutances three times a weak	high	low	easy	-0.090441429
147	9	1	iran	2.5	subcutances three times a weak	medium	low	hard	-0.617903819
147	9	0	other	0.5	subcutances other days	high	medium	hard	-0.643869086
147	10	0	other	2.5	mascular once a weak	high	low	hard	0.00383736
147	10	1	iran	5.5	mascular once a weak	medium	medium	easy	-0.697626621
147	11	0	other	5.5	subcutances other days	high	medium	easy	-0.34108841
147	11	1	iran	0.5	subcutances three times a weak	medium	low	hard	-0.699689278
147	12	0	iran	5.5	subcutances three times a weak	high	medium	hard	-0.787756632
147	12	1	other	2.5	subcutances other days	medium	low	hard	-0.352164436
148	1	1	iran	5.5	mascular once a weak	high	medium	hard	-0.572088286
148	1	0	iran	0.5	subcutances three times a weak	high	medium	easy	-0.72867963
148	2	1	other	0.5	subcutances other days	high	medium	hard	-0.643869086
148	2	0	iran	5.5	subcutances other days	medium	medium	hard	-0.928090082
148	3	0	iran	5.5	mascular once a weak	high	medium	hard	-0.572088286
148	3	1	iran	2.5	subcutances three times a weak	high	medium	easy	-0.646894171

148	4	0	iran	0.5	subcutances three times a weak	medium	low	hard	-0.699689278
148	4	1	other	2.5	mascular once a weak	high	low	hard	0.00383736
148	5	0	iran	0.5	mascular once a weak	medium	low	easy	-0.303092094
148	5	1	other	2.5	subcutances three times a weak	high	low	easy	-0.030902146
148	6	1	other	2.5	subcutances three times a weak	high	low	easy	-0.030902146
148	6	0	other	0.5	subcutances other days	high	medium	easy	-0.462940247
148	7	1	other	0.5	mascular once a weak	medium	medium	hard	-0.900801637
148	7	0	other	5.5	subcutances other days	medium	low	hard	-0.312098058
148	8	0	iran	2.5	mascular once a weak	high	low	hard	-0.0957683
148	8	1	iran	5.5	subcutances three times a weak	high	low	easy	-0.090441429
148	9	0	iran	2.5	subcutances three times a weak	medium	low	hard	-0.617903819
148	9	1	other	0.5	subcutances other days	high	medium	hard	-0.643869086
148	10	1	other	2.5	mascular once a weak	high	low	hard	0.00383736
148	10	0	iran	5.5	mascular once a weak	medium	medium	easy	-0.697626621
148	11	1	other	5.5	subcutances other days	high	medium	easy	-0.34108841
148	11	0	iran	0.5	subcutances three times a weak	medium	low	hard	-0.699689278
148	12	0	iran	5.5	subcutances three times a weak	high	medium	hard	-0.787756632
148	12	1	other	2.5	subcutances other days	medium	low	hard	-0.352164436
149	1	1	iran	5.5	mascular once a weak	high	medium	hard	-0.572088286
149	1	0	iran	0.5	subcutances three times a weak	high	medium	easy	-0.72867963
149	2	1	other	0.5	subcutances other days	high	medium	hard	-0.643869086
149	2	0	iran	5.5	subcutances other days	medium	medium	hard	-0.928090082
149	3	1	iran	5.5	mascular once a weak	high	medium	hard	-0.572088286
149	3	0	iran	2.5	subcutances three times a weak	high	medium	easy	-0.646894171
149	4	0	iran	0.5	subcutances three times a weak	medium	low	hard	-0.699689278
149	4	1	other	2.5	mascular once a weak	high	low	hard	0.00383736
149	5	1	iran	0.5	mascular once a weak	medium	low	easy	-0.303092094
149	5	0	other	2.5	subcutances three times a weak	high	low	easy	-0.030902146
149	6	1	other	2.5	subcutances three times a weak	high	low	easy	-0.030902146
149	6	0	other	0.5	subcutances other days	high	medium	easy	-0.462940247
149	7	1	other	0.5	mascular once a weak	medium	medium	hard	-0.900801637
149	7	0	other	5.5	subcutances other days	medium	low	hard	-0.312098058
149	8	1	iran	2.5	mascular once a weak	high	low	hard	-0.0957683
149	8	0	iran	5.5	subcutances three times a weak	high	low	easy	-0.090441429

149	9	0	iran	2.5	subcutances three times a weak	medium	low	hard	-0.617903819
149	9	1	other	0.5	subcutances other days	high	medium	hard	-0.643869086
149	10	1	other	2.5	mascular once a weak	high	low	hard	0.00383736
149	10	0	iran	5.5	mascular once a weak	medium	medium	easy	-0.697626621
149	11	1	other	5.5	subcutances other days	high	medium	easy	-0.34108841
149	11	0	iran	0.5	subcutances three times a weak	medium	low	hard	-0.699689278
149	12	1	iran	5.5	subcutances three times a weak	high	medium	hard	-0.787756632
149	12	0	other	2.5	subcutances other days	medium	low	hard	-0.352164436
150	1	0	iran	5.5	mascular once a weak	high	medium	hard	-0.572088286
150	1	1	iran	0.5	subcutances three times a weak	high	medium	easy	-0.72867963
150	2	1	other	0.5	subcutances other days	high	medium	hard	-0.643869086
150	2	0	iran	5.5	subcutances other days	medium	medium	hard	-0.928090082
150	3	0	iran	5.5	mascular once a weak	high	medium	hard	-0.572088286
150	3	1	iran	2.5	subcutances three times a weak	high	medium	easy	-0.646894171
150	4	0	iran	0.5	subcutances three times a weak	medium	low	hard	-0.699689278
150	4	1	other	2.5	mascular once a weak	high	low	hard	0.00383736
150	5	0	iran	0.5	mascular once a weak	medium	low	easy	-0.303092094
150	5	1	other	2.5	subcutances three times a weak	high	low	easy	-0.030902146
150	6	1	other	2.5	subcutances three times a weak	high	low	easy	-0.030902146
150	6	0	other	0.5	subcutances other days	high	medium	easy	-0.462940247
150	7	0	other	0.5	mascular once a weak	medium	medium	hard	-0.900801637
150	7	1	other	5.5	subcutances other days	medium	low	hard	-0.312098058
150	8	0	iran	2.5	mascular once a weak	high	low	hard	-0.0957683
150	8	1	iran	5.5	subcutances three times a weak	high	low	easy	-0.090441429
150	9	0	iran	2.5	subcutances three times a weak	medium	low	hard	-0.617903819
150	9	1	other	0.5	subcutances other days	high	medium	hard	-0.643869086
150	10	1	other	2.5	mascular once a weak	high	low	hard	0.00383736
150	10	0	iran	5.5	mascular once a weak	medium	medium	easy	-0.697626621
150	11	1	other	5.5	subcutances other days	high	medium	easy	-0.34108841
150	11	0	iran	0.5	subcutances three times a weak	medium	low	hard	-0.699689278
150	12	0	iran	5.5	subcutances three times a weak	high	medium	hard	-0.787756632
150	12	1	other	2.5	subcutances other days	medium	low	hard	-0.352164436
151	1	0	iran	5.5	mascular once a weak	high	medium	hard	-0.572088286
151	1	1	iran	0.5	subcutances three times a weak	high	medium	easy	-0.72867963

151	2	1	other	0.5	subcutances other days	high	medium	hard	-0.643869086
151	2	0	iran	5.5	subcutances other days	medium	medium	hard	-0.928090082
151	3	0	iran	5.5	mascular once a weak	high	medium	hard	-0.572088286
151	3	1	iran	2.5	subcutances three times a weak	high	medium	easy	-0.646894171
151	4	0	iran	0.5	subcutances three times a weak	medium	low	hard	-0.699689278
151	4	1	other	2.5	mascular once a weak	high	low	hard	0.00383736
151	5	0	iran	0.5	mascular once a weak	medium	low	easy	-0.303092094
151	5	1	other	2.5	subcutances three times a weak	high	low	easy	-0.030902146
151	6	1	other	2.5	subcutances three times a weak	high	low	easy	-0.030902146
151	6	0	other	0.5	subcutances other days	high	medium	easy	-0.462940247
151	7	0	other	0.5	mascular once a weak	medium	medium	hard	-0.900801637
151	7	1	other	5.5	subcutances other days	medium	low	hard	-0.312098058
151	8	0	iran	2.5	mascular once a weak	high	low	hard	-0.0957683
151	8	1	iran	5.5	subcutances three times a weak	high	low	easy	-0.090441429
151	9	0	iran	2.5	subcutances three times a weak	medium	low	hard	-0.617903819
151	9	1	other	0.5	subcutances other days	high	medium	hard	-0.643869086
151	10	1	other	2.5	mascular once a weak	high	low	hard	0.00383736
151	10	0	iran	5.5	mascular once a weak	medium	medium	easy	-0.697626621
151	11	1	other	5.5	subcutances other days	high	medium	easy	-0.34108841
151	11	0	iran	0.5	subcutances three times a weak	medium	low	hard	-0.699689278
151	12	1	iran	5.5	subcutances three times a weak	high	medium	hard	-0.787756632
151	12	0	other	2.5	subcutances other days	medium	low	hard	-0.352164436
152	1	0	iran	5.5	mascular once a weak	high	medium	hard	-0.572088286
152	1	1	iran	0.5	subcutances three times a weak	high	medium	easy	-0.72867963
152	2	1	other	0.5	subcutances other days	high	medium	hard	-0.643869086
152	2	0	iran	5.5	subcutances other days	medium	medium	hard	-0.928090082
152	3	0	iran	5.5	mascular once a weak	high	medium	hard	-0.572088286
152	3	1	iran	2.5	subcutances three times a weak	high	medium	easy	-0.646894171
152	4	0	iran	0.5	subcutances three times a weak	medium	low	hard	-0.699689278
152	4	1	other	2.5	mascular once a weak	high	low	hard	0.00383736
152	5	0	iran	0.5	mascular once a weak	medium	low	easy	-0.303092094
152	5	1	other	2.5	subcutances three times a weak	high	low	easy	-0.030902146
152	6	1	other	2.5	subcutances three times a weak	high	low	easy	-0.030902146
152	6	0	other	0.5	subcutances other days	high	medium	easy	-0.462940247

152	7	0	other	0.5	mascular once a weak	medium	medium	hard	-0.900801637
152	7	1	other	5.5	subcutances other days	medium	low	hard	-0.312098058
152	8	0	iran	2.5	mascular once a weak	high	low	hard	-0.0957683
152	8	1	iran	5.5	subcutances three times a weak	high	low	easy	-0.090441429
152	9	1	iran	2.5	subcutances three times a weak	medium	low	hard	-0.617903819
152	9	0	other	0.5	subcutances other days	high	medium	hard	-0.643869086
152	10	1	other	2.5	mascular once a weak	high	low	hard	0.00383736
152	10	0	iran	5.5	mascular once a weak	medium	medium	easy	-0.697626621
152	11	0	other	5.5	subcutances other days	high	medium	easy	-0.34108841
152	11	1	iran	0.5	subcutances three times a weak	medium	low	hard	-0.699689278
152	12	0	iran	5.5	subcutances three times a weak	high	medium	hard	-0.787756632
152	12	1	other	2.5	subcutances other days	medium	low	hard	-0.352164436
153	1	0	iran	5.5	mascular once a weak	high	medium	hard	-0.572088286
153	1	1	iran	0.5	subcutances three times a weak	high	medium	easy	-0.72867963
153	2	0	other	0.5	subcutances other days	high	medium	hard	-0.643869086
153	2	1	iran	5.5	subcutances other days	medium	medium	hard	-0.928090082
153	3	0	iran	5.5	mascular once a weak	high	medium	hard	-0.572088286
153	3	1	iran	2.5	subcutances three times a weak	high	medium	easy	-0.646894171
153	4	1	iran	0.5	subcutances three times a weak	medium	low	hard	-0.699689278
153	4	0	other	2.5	mascular once a weak	high	low	hard	0.00383736
153	5	0	iran	0.5	mascular once a weak	medium	low	easy	-0.303092094
153	5	1	other	2.5	subcutances three times a weak	high	low	easy	-0.030902146
153	6	0	other	2.5	subcutances three times a weak	high	low	easy	-0.030902146
153	6	0	other	0.5	subcutances other days	high	medium	easy	-0.462940247
153	7	0	other	0.5	mascular once a weak	medium	medium	hard	-0.900801637
153	7	1	other	5.5	subcutances other days	medium	low	hard	-0.312098058
153	8	0	iran	2.5	mascular once a weak	high	low	hard	-0.0957683
153	8	1	iran	5.5	subcutances three times a weak	high	low	easy	-0.090441429
153	9	1	iran	2.5	subcutances three times a weak	medium	low	hard	-0.617903819
153	9	0	other	0.5	subcutances other days	high	medium	hard	-0.643869086
153	10	0	other	2.5	mascular once a weak	high	low	hard	0.00383736
153	10	0	iran	5.5	mascular once a weak	medium	medium	easy	-0.697626621
153	11	0	other	5.5	subcutances other days	high	medium	easy	-0.34108841
153	11	1	iran	0.5	subcutances three times a weak	medium	low	hard	-0.699689278

153	12	0	iran	5.5	subcutances three times a weak	high	medium	hard	-0.787756632
153	12	1	other	2.5	subcutances other days	medium	low	hard	-0.352164436
154	1	1	iran	5.5	mascular once a weak	high	medium	hard	-0.572088286
154	1	0	iran	0.5	subcutances three times a weak	high	medium	easy	-0.72867963
154	2	1	other	0.5	subcutances other days	high	medium	hard	-0.643869086
154	2	0	iran	5.5	subcutances other days	medium	medium	hard	-0.928090082
154	3	0	iran	5.5	mascular once a weak	high	medium	hard	-0.572088286
154	3	1	iran	2.5	subcutances three times a weak	high	medium	easy	-0.646894171
154	4	0	iran	0.5	subcutances three times a weak	medium	low	hard	-0.699689278
154	4	1	other	2.5	mascular once a weak	high	low	hard	0.00383736
154	5	0	iran	0.5	mascular once a weak	medium	low	easy	-0.303092094
154	5	1	other	2.5	subcutances three times a weak	high	low	easy	-0.030902146
154	6	0	other	2.5	subcutances three times a weak	high	low	easy	-0.030902146
154	6	1	other	0.5	subcutances other days	high	medium	easy	-0.462940247
154	7	0	other	0.5	mascular once a weak	medium	medium	hard	-0.900801637
154	7	1	other	5.5	subcutances other days	medium	low	hard	-0.312098058
154	8	1	iran	2.5	mascular once a weak	high	low	hard	-0.0957683
154	8	0	iran	5.5	subcutances three times a weak	high	low	easy	-0.090441429
154	9	0	iran	2.5	subcutances three times a weak	medium	low	hard	-0.617903819
154	9	1	other	0.5	subcutances other days	high	medium	hard	-0.643869086
154	10	1	other	2.5	mascular once a weak	high	low	hard	0.00383736
154	10	0	iran	5.5	mascular once a weak	medium	medium	easy	-0.697626621
154	11	1	other	5.5	subcutances other days	high	medium	easy	-0.34108841
154	11	0	iran	0.5	subcutances three times a weak	medium	low	hard	-0.699689278
154	12	1	iran	5.5	subcutances three times a weak	high	medium	hard	-0.787756632
154	12	0	other	2.5	subcutances other days	medium	low	hard	-0.352164436
155	1	1	iran	5.5	mascular once a weak	high	medium	hard	-0.572088286
155	1	0	iran	0.5	subcutances three times a weak	high	medium	easy	-0.72867963
155	2	1	other	0.5	subcutances other days	high	medium	hard	-0.643869086
155	2	0	iran	5.5	subcutances other days	medium	medium	hard	-0.928090082
155	3	1	iran	5.5	mascular once a weak	high	medium	hard	-0.572088286
155	3	0	iran	2.5	subcutances three times a weak	high	medium	easy	-0.646894171
155	4	0	iran	0.5	subcutances three times a weak	medium	low	hard	-0.699689278
155	4	1	other	2.5	mascular once a weak	high	low	hard	0.00383736

155	5	0	iran	0.5	mascular once a weak	medium	low	easy	-0.303092094
155	5	1	other	2.5	subcutances three times a weak	high	low	easy	-0.030902146
155	6	1	other	2.5	subcutances three times a weak	high	low	easy	-0.030902146
155	6	0	other	0.5	subcutances other days	high	medium	easy	-0.462940247
155	7	0	other	0.5	mascular once a weak	medium	medium	hard	-0.900801637
155	7	1	other	5.5	subcutances other days	medium	low	hard	-0.312098058
155	8	1	iran	2.5	mascular once a weak	high	low	hard	-0.0957683
155	8	0	iran	5.5	subcutances three times a weak	high	low	easy	-0.090441429
155	9	0	iran	2.5	subcutances three times a weak	medium	low	hard	-0.617903819
155	9	1	other	0.5	subcutances other days	high	medium	hard	-0.643869086
155	10	1	other	2.5	mascular once a weak	high	low	hard	0.00383736
155	10	0	iran	5.5	mascular once a weak	medium	medium	easy	-0.697626621
155	11	1	other	5.5	subcutances other days	high	medium	easy	-0.34108841
155	11	0	iran	0.5	subcutances three times a weak	medium	low	hard	-0.699689278
155	12	1	iran	5.5	subcutances three times a weak	high	medium	hard	-0.787756632
155	12	0	other	2.5	subcutances other days	medium	low	hard	-0.352164436
156	1	1	iran	5.5	mascular once a weak	high	medium	hard	-0.572088286
156	1	0	iran	0.5	subcutances three times a weak	high	medium	easy	-0.72867963
156	2	1	other	0.5	subcutances other days	high	medium	hard	-0.643869086
156	2	0	iran	5.5	subcutances other days	medium	medium	hard	-0.928090082
156	3	1	iran	5.5	mascular once a weak	high	medium	hard	-0.572088286
156	3	0	iran	2.5	subcutances three times a weak	high	medium	easy	-0.646894171
156	4	0	iran	0.5	subcutances three times a weak	medium	low	hard	-0.699689278
156	4	1	other	2.5	mascular once a weak	high	low	hard	0.00383736
156	5	0	iran	0.5	mascular once a weak	medium	low	easy	-0.303092094
156	5	1	other	2.5	subcutances three times a weak	high	low	easy	-0.030902146
156	6	0	other	2.5	subcutances three times a weak	high	low	easy	-0.030902146
156	6	1	other	0.5	subcutances other days	high	medium	easy	-0.462940247
156	7	1	other	0.5	mascular once a weak	medium	medium	hard	-0.900801637
156	7	0	other	5.5	subcutances other days	medium	low	hard	-0.312098058
156	8	1	iran	2.5	mascular once a weak	high	low	hard	-0.0957683
156	8	0	iran	5.5	subcutances three times a weak	high	low	easy	-0.090441429
156	9	0	iran	2.5	subcutances three times a weak	medium	low	hard	-0.617903819
156	9	1	other	0.5	subcutances other days	high	medium	hard	-0.643869086

156	10	1	other	2.5	mascular once a weak	high	low	hard	0.00383736
156	10	0	iran	5.5	mascular once a weak	medium	medium	easy	-0.697626621
156	11	1	other	5.5	subcutances other days	high	medium	easy	-0.34108841
156	11	0	iran	0.5	subcutances three times a weak	medium	low	hard	-0.699689278
156	12	0	iran	5.5	subcutances three times a weak	high	medium	hard	-0.787756632
156	12	1	other	2.5	subcutances other days	medium	low	hard	-0.352164436
157	1	0	iran	5.5	mascular once a weak	high	medium	hard	-0.572088286
157	1	1	iran	0.5	subcutances three times a weak	high	medium	easy	-0.72867963
157	2	1	other	0.5	subcutances other days	high	medium	hard	-0.643869086
157	2	0	iran	5.5	subcutances other days	medium	medium	hard	-0.928090082
157	3	0	iran	5.5	mascular once a weak	high	medium	hard	-0.572088286
157	3	1	iran	2.5	subcutances three times a weak	high	medium	easy	-0.646894171
157	4	0	iran	0.5	subcutances three times a weak	medium	low	hard	-0.699689278
157	4	1	other	2.5	mascular once a weak	high	low	hard	0.00383736
157	5	0	iran	0.5	mascular once a weak	medium	low	easy	-0.303092094
157	5	1	other	2.5	subcutances three times a weak	high	low	easy	-0.030902146
157	6	1	other	2.5	subcutances three times a weak	high	low	easy	-0.030902146
157	6	0	other	0.5	subcutances other days	high	medium	easy	-0.462940247
157	7	0	other	0.5	mascular once a weak	medium	medium	hard	-0.900801637
157	7	1	other	5.5	subcutances other days	medium	low	hard	-0.312098058
157	8	0	iran	2.5	mascular once a weak	high	low	hard	-0.0957683
157	8	1	iran	5.5	subcutances three times a weak	high	low	easy	-0.090441429
157	9	1	iran	2.5	subcutances three times a weak	medium	low	hard	-0.617903819
157	9	0	other	0.5	subcutances other days	high	medium	hard	-0.643869086
157	10	1	other	2.5	mascular once a weak	high	low	hard	0.00383736
157	10	0	iran	5.5	mascular once a weak	medium	medium	easy	-0.697626621
157	11	1	other	5.5	subcutances other days	high	medium	easy	-0.34108841
157	11	0	iran	0.5	subcutances three times a weak	medium	low	hard	-0.699689278
157	12	0	iran	5.5	subcutances three times a weak	high	medium	hard	-0.787756632
157	12	1	other	2.5	subcutances other days	medium	low	hard	-0.352164436
158	1	1	iran	5.5	mascular once a weak	high	medium	hard	-0.572088286
158	1	0	iran	0.5	subcutances three times a weak	high	medium	easy	-0.72867963
158	2	0	other	0.5	subcutances other days	high	medium	hard	-0.643869086
158	2	1	iran	5.5	subcutances other days	medium	medium	hard	-0.928090082

158	3	0	iran	5.5	mascular once a weak	high	medium	hard	-0.572088286
158	3	1	iran	2.5	subcutances three times a weak	high	medium	easy	-0.646894171
158	4	1	iran	0.5	subcutances three times a weak	medium	low	hard	-0.699689278
158	4	0	other	2.5	mascular once a weak	high	low	hard	0.00383736
158	5	1	iran	0.5	mascular once a weak	medium	low	easy	-0.303092094
158	5	0	other	2.5	subcutances three times a weak	high	low	easy	-0.030902146
158	6	0	other	2.5	subcutances three times a weak	high	low	easy	-0.030902146
158	6	1	other	0.5	subcutances other days	high	medium	easy	-0.462940247
158	7	0	other	0.5	mascular once a weak	medium	medium	hard	-0.900801637
158	7	1	other	5.5	subcutances other days	medium	low	hard	-0.312098058
158	8	1	iran	2.5	mascular once a weak	high	low	hard	-0.0957683
158	8	0	iran	5.5	subcutances three times a weak	high	low	easy	-0.090441429
158	9	1	iran	2.5	subcutances three times a weak	medium	low	hard	-0.617903819
158	9	0	other	0.5	subcutances other days	high	medium	hard	-0.643869086
158	10	0	other	2.5	mascular once a weak	high	low	hard	0.00383736
158	10	1	iran	5.5	mascular once a weak	medium	medium	easy	-0.697626621
158	11	0	other	5.5	subcutances other days	high	medium	easy	-0.34108841
158	11	1	iran	0.5	subcutances three times a weak	medium	low	hard	-0.699689278
158	12	0	iran	5.5	subcutances three times a weak	high	medium	hard	-0.787756632
158	12	1	other	2.5	subcutances other days	medium	low	hard	-0.352164436
159	1	1	iran	5.5	mascular once a weak	high	medium	hard	-0.572088286
159	1	0	iran	0.5	subcutances three times a weak	high	medium	easy	-0.72867963
159	2	1	other	0.5	subcutances other days	high	medium	hard	-0.643869086
159	2	0	iran	5.5	subcutances other days	medium	medium	hard	-0.928090082
159	3	0	iran	5.5	mascular once a weak	high	medium	hard	-0.572088286
159	3	1	iran	2.5	subcutances three times a weak	high	medium	easy	-0.646894171
159	4	0	iran	0.5	subcutances three times a weak	medium	low	hard	-0.699689278
159	4	1	other	2.5	mascular once a weak	high	low	hard	0.00383736
159	5	1	iran	0.5	mascular once a weak	medium	low	easy	-0.303092094
159	5	0	other	2.5	subcutances three times a weak	high	low	easy	-0.030902146
159	6	0	other	2.5	subcutances three times a weak	high	low	easy	-0.030902146
159	6	1	other	0.5	subcutances other days	high	medium	easy	-0.462940247
159	7	0	other	0.5	mascular once a weak	medium	medium	hard	-0.900801637
159	7	1	other	5.5	subcutances other days	medium	low	hard	-0.312098058

159	8	1	iran	2.5	mascular once a weak	high	low	hard	-0.0957683
159	8	0	iran	5.5	subcutances three times a weak	high	low	easy	-0.090441429
159	9	1	iran	2.5	subcutances three times a weak	medium	low	hard	-0.617903819
159	9	0	other	0.5	subcutances other days	high	medium	hard	-0.643869086
159	10	1	other	2.5	mascular once a weak	high	low	hard	0.00383736
159	10	0	iran	5.5	mascular once a weak	medium	medium	easy	-0.697626621
159	11	0	other	5.5	subcutances other days	high	medium	easy	-0.34108841
159	11	1	iran	0.5	subcutances three times a weak	medium	low	hard	-0.699689278
159	12	0	iran	5.5	subcutances three times a weak	high	medium	hard	-0.787756632
159	12	1	other	2.5	subcutances other days	medium	low	hard	-0.352164436
160	1	1	iran	5.5	mascular once a weak	high	medium	hard	-0.572088286
160	1	0	iran	0.5	subcutances three times a weak	high	medium	easy	-0.72867963
160	2	0	other	0.5	subcutances other days	high	medium	hard	-0.643869086
160	2	1	iran	5.5	subcutances other days	medium	medium	hard	-0.928090082
160	3	0	iran	5.5	mascular once a weak	high	medium	hard	-0.572088286
160	3	1	iran	2.5	subcutances three times a weak	high	medium	easy	-0.646894171
160	4	1	iran	0.5	subcutances three times a weak	medium	low	hard	-0.699689278
160	4	0	other	2.5	mascular once a weak	high	low	hard	0.00383736
160	5	1	iran	0.5	mascular once a weak	medium	low	easy	-0.303092094
160	5	0	other	2.5	subcutances three times a weak	high	low	easy	-0.030902146
160	6	0	other	2.5	subcutances three times a weak	high	low	easy	-0.030902146
160	6	1	other	0.5	subcutances other days	high	medium	easy	-0.462940247
160	7	0	other	0.5	mascular once a weak	medium	medium	hard	-0.900801637
160	7	1	other	5.5	subcutances other days	medium	low	hard	-0.312098058
160	8	1	iran	2.5	mascular once a weak	high	low	hard	-0.0957683
160	8	0	iran	5.5	subcutances three times a weak	high	low	easy	-0.090441429
160	9	1	iran	2.5	subcutances three times a weak	medium	low	hard	-0.617903819
160	9	0	other	0.5	subcutances other days	high	medium	hard	-0.643869086
160	10	0	other	2.5	mascular once a weak	high	low	hard	0.00383736
160	10	1	iran	5.5	mascular once a weak	medium	medium	easy	-0.697626621
160	11	0	other	5.5	subcutances other days	high	medium	easy	-0.34108841
160	11	1	iran	0.5	subcutances three times a weak	medium	low	hard	-0.699689278
160	12	0	iran	5.5	subcutances three times a weak	high	medium	hard	-0.787756632
160	12	1	other	2.5	subcutances other days	medium	low	hard	-0.352164436

161	1	0	iran	5.5	mascular once a weak	high	medium	hard	-0.572088286
161	1	1	iran	0.5	subcutances three times a weak	high	medium	easy	-0.72867963
161	2	1	other	0.5	subcutances other days	high	medium	hard	-0.643869086
161	2	0	iran	5.5	subcutances other days	medium	medium	hard	-0.928090082
161	3	0	iran	5.5	mascular once a weak	high	medium	hard	-0.572088286
161	3	0	iran	2.5	subcutances three times a weak	high	medium	easy	-0.646894171
161	4	0	iran	0.5	subcutances three times a weak	medium	low	hard	-0.699689278
161	4	1	other	2.5	mascular once a weak	high	low	hard	0.00383736
161	5	0	iran	0.5	mascular once a weak	medium	low	easy	-0.303092094
161	5	1	other	2.5	subcutances three times a weak	high	low	easy	-0.030902146
161	6	0	other	2.5	subcutances three times a weak	high	low	easy	-0.030902146
161	6	1	other	0.5	subcutances other days	high	medium	easy	-0.462940247
161	7	0	other	0.5	mascular once a weak	medium	medium	hard	-0.900801637
161	7	1	other	5.5	subcutances other days	medium	low	hard	-0.312098058
161	8	0	iran	2.5	mascular once a weak	high	low	hard	-0.0957683
161	8	0	iran	5.5	subcutances three times a weak	high	low	easy	-0.090441429
161	9	0	iran	2.5	subcutances three times a weak	medium	low	hard	-0.617903819
161	9	1	other	0.5	subcutances other days	high	medium	hard	-0.643869086
161	10	1	other	2.5	mascular once a weak	high	low	hard	0.00383736
161	10	0	iran	5.5	mascular once a weak	medium	medium	easy	-0.697626621
161	11	1	other	5.5	subcutances other days	high	medium	easy	-0.34108841
161	11	0	iran	0.5	subcutances three times a weak	medium	low	hard	-0.699689278
161	12	0	iran	5.5	subcutances three times a weak	high	medium	hard	-0.787756632
161	12	1	other	2.5	subcutances other days	medium	low	hard	-0.352164436
162	1	1	iran	5.5	mascular once a weak	high	medium	hard	-0.572088286
162	1	0	iran	0.5	subcutances three times a weak	high	medium	easy	-0.72867963
162	2	0	other	0.5	subcutances other days	high	medium	hard	-0.643869086
162	2	1	iran	5.5	subcutances other days	medium	medium	hard	-0.928090082
162	3	0	iran	5.5	mascular once a weak	high	medium	hard	-0.572088286
162	3	1	iran	2.5	subcutances three times a weak	high	medium	easy	-0.646894171
162	4	1	iran	0.5	subcutances three times a weak	medium	low	hard	-0.699689278
162	4	0	other	2.5	mascular once a weak	high	low	hard	0.00383736
162	5	1	iran	0.5	mascular once a weak	medium	low	easy	-0.303092094
162	5	0	other	2.5	subcutances three times a weak	high	low	easy	-0.030902146

162	6	0	other	2.5	subcutances three times a weak	high	low	easy	-0.030902146
162	6	1	other	0.5	subcutances other days	high	medium	easy	-0.462940247
162	7	0	other	0.5	mascular once a weak	medium	medium	hard	-0.900801637
162	7	1	other	5.5	subcutances other days	medium	low	hard	-0.312098058
162	8	1	iran	2.5	mascular once a weak	high	low	hard	-0.0957683
162	8	0	iran	5.5	subcutances three times a weak	high	low	easy	-0.090441429
162	9	1	iran	2.5	subcutances three times a weak	medium	low	hard	-0.617903819
162	9	0	other	0.5	subcutances other days	high	medium	hard	-0.643869086
162	10	0	other	2.5	mascular once a weak	high	low	hard	0.00383736
162	10	1	iran	5.5	mascular once a weak	medium	medium	easy	-0.697626621
162	11	0	other	5.5	subcutances other days	high	medium	easy	-0.34108841
162	11	1	iran	0.5	subcutances three times a weak	medium	low	hard	-0.699689278
162	12	0	iran	5.5	subcutances three times a weak	high	medium	hard	-0.787756632
162	12	1	other	2.5	subcutances other days	medium	low	hard	-0.352164436
163	1	1	iran	5.5	mascular once a weak	high	medium	hard	-0.572088286
163	1	0	iran	0.5	subcutances three times a weak	high	medium	easy	-0.72867963
163	2	0	other	0.5	subcutances other days	high	medium	hard	-0.643869086
163	2	1	iran	5.5	subcutances other days	medium	medium	hard	-0.928090082
163	3	0	iran	5.5	mascular once a weak	high	medium	hard	-0.572088286
163	3	1	iran	2.5	subcutances three times a weak	high	medium	easy	-0.646894171
163	4	0	iran	0.5	subcutances three times a weak	medium	low	hard	-0.699689278
163	4	1	other	2.5	mascular once a weak	high	low	hard	0.00383736
163	5	1	iran	0.5	mascular once a weak	medium	low	easy	-0.303092094
163	5	0	other	2.5	subcutances three times a weak	high	low	easy	-0.030902146
163	6	0	other	2.5	subcutances three times a weak	high	low	easy	-0.030902146
163	6	1	other	0.5	subcutances other days	high	medium	easy	-0.462940247
163	7	1	other	0.5	mascular once a weak	medium	medium	hard	-0.900801637
163	7	0	other	5.5	subcutances other days	medium	low	hard	-0.312098058
163	8	1	iran	2.5	mascular once a weak	high	low	hard	-0.0957683
163	8	0	iran	5.5	subcutances three times a weak	high	low	easy	-0.090441429
163	9	1	iran	2.5	subcutances three times a weak	medium	low	hard	-0.617903819
163	9	0	other	0.5	subcutances other days	high	medium	hard	-0.643869086
163	10	0	other	2.5	mascular once a weak	high	low	hard	0.00383736
163	10	1	iran	5.5	mascular once a weak	medium	medium	easy	-0.697626621

163	11	0	other	5.5	subcutances other days	high	medium	easy	-0.34108841
163	11	1	iran	0.5	subcutances three times a weak	medium	low	hard	-0.699689278
163	12	1	iran	5.5	subcutances three times a weak	high	medium	hard	-0.787756632
163	12	0	other	2.5	subcutances other days	medium	low	hard	-0.352164436
164	1	1	iran	5.5	mascular once a weak	high	medium	hard	-0.572088286
164	1	0	iran	0.5	subcutances three times a weak	high	medium	easy	-0.72867963
164	2	0	other	0.5	subcutances other days	high	medium	hard	-0.643869086
164	2	1	iran	5.5	subcutances other days	medium	medium	hard	-0.928090082
164	3	0	iran	5.5	mascular once a weak	high	medium	hard	-0.572088286
164	3	1	iran	2.5	subcutances three times a weak	high	medium	easy	-0.646894171
164	4	1	iran	0.5	subcutances three times a weak	medium	low	hard	-0.699689278
164	4	0	other	2.5	mascular once a weak	high	low	hard	0.00383736
164	5	1	iran	0.5	mascular once a weak	medium	low	easy	-0.303092094
164	5	0	other	2.5	subcutances three times a weak	high	low	easy	-0.030902146
164	6	0	other	2.5	subcutances three times a weak	high	low	easy	-0.030902146
164	6	1	other	0.5	subcutances other days	high	medium	easy	-0.462940247
164	7	0	other	0.5	mascular once a weak	medium	medium	hard	-0.900801637
164	7	0	other	5.5	subcutances other days	medium	low	hard	-0.312098058
164	8	1	iran	2.5	mascular once a weak	high	low	hard	-0.0957683
164	8	0	iran	5.5	subcutances three times a weak	high	low	easy	-0.090441429
164	9	1	iran	2.5	subcutances three times a weak	medium	low	hard	-0.617903819
164	9	0	other	0.5	subcutances other days	high	medium	hard	-0.643869086
164	10	0	other	2.5	mascular once a weak	high	low	hard	0.00383736
164	10	1	iran	5.5	mascular once a weak	medium	medium	easy	-0.697626621
164	11	0	other	5.5	subcutances other days	high	medium	easy	-0.34108841
164	11	1	iran	0.5	subcutances three times a weak	medium	low	hard	-0.699689278
164	12	0	iran	5.5	subcutances three times a weak	high	medium	hard	-0.787756632
164	12	1	other	2.5	subcutances other days	medium	low	hard	-0.352164436
165	1	1	iran	5.5	mascular once a weak	high	medium	hard	-0.572088286
165	1	0	iran	0.5	subcutances three times a weak	high	medium	easy	-0.72867963
165	2	0	other	0.5	subcutances other days	high	medium	hard	-0.643869086
165	2	1	iran	5.5	subcutances other days	medium	medium	hard	-0.928090082
165	3	0	iran	5.5	mascular once a weak	high	medium	hard	-0.572088286
165	3	1	iran	2.5	subcutances three times a weak	high	medium	easy	-0.646894171

165	4	0	iran	0.5	subcutances three times a weak	medium	low	hard	-0.699689278
165	4	1	other	2.5	mascular once a weak	high	low	hard	0.00383736
165	5	1	iran	0.5	mascular once a weak	medium	low	easy	-0.303092094
165	5	0	other	2.5	subcutances three times a weak	high	low	easy	-0.030902146
165	6	0	other	2.5	subcutances three times a weak	high	low	easy	-0.030902146
165	6	1	other	0.5	subcutances other days	high	medium	easy	-0.462940247
165	7	0	other	0.5	mascular once a weak	medium	medium	hard	-0.900801637
165	7	1	other	5.5	subcutances other days	medium	low	hard	-0.312098058
165	8	1	iran	2.5	mascular once a weak	high	low	hard	-0.0957683
165	8	0	iran	5.5	subcutances three times a weak	high	low	easy	-0.090441429
165	9	1	iran	2.5	subcutances three times a weak	medium	low	hard	-0.617903819
165	9	0	other	0.5	subcutances other days	high	medium	hard	-0.643869086
165	10	0	other	2.5	mascular once a weak	high	low	hard	0.00383736
165	10	1	iran	5.5	mascular once a weak	medium	medium	easy	-0.697626621
165	11	1	other	5.5	subcutances other days	high	medium	easy	-0.34108841
165	11	0	iran	0.5	subcutances three times a weak	medium	low	hard	-0.699689278
165	12	0	iran	5.5	subcutances three times a weak	high	medium	hard	-0.787756632
165	12	1	other	2.5	subcutances other days	medium	low	hard	-0.352164436
166	1	1	iran	5.5	mascular once a weak	high	medium	hard	-0.572088286
166	1	0	iran	0.5	subcutances three times a weak	high	medium	easy	-0.72867963
166	2	0	other	0.5	subcutances other days	high	medium	hard	-0.643869086
166	2	1	iran	5.5	subcutances other days	medium	medium	hard	-0.928090082
166	3	1	iran	5.5	mascular once a weak	high	medium	hard	-0.572088286
166	3	0	iran	2.5	subcutances three times a weak	high	medium	easy	-0.646894171
166	4	0	iran	0.5	subcutances three times a weak	medium	low	hard	-0.699689278
166	4	1	other	2.5	mascular once a weak	high	low	hard	0.00383736
166	5	1	iran	0.5	mascular once a weak	medium	low	easy	-0.303092094
166	5	0	other	2.5	subcutances three times a weak	high	low	easy	-0.030902146
166	6	1	other	2.5	subcutances three times a weak	high	low	easy	-0.030902146
166	6	0	other	0.5	subcutances other days	high	medium	easy	-0.462940247
166	7	1	other	0.5	mascular once a weak	medium	medium	hard	-0.900801637
166	7	0	other	5.5	subcutances other days	medium	low	hard	-0.312098058
166	8	1	iran	2.5	mascular once a weak	high	low	hard	-0.0957683
166	8	0	iran	5.5	subcutances three times a weak	high	low	easy	-0.090441429

166	9	1	iran	2.5	subcutances three times a weak	medium	low	hard	-0.617903819
166	9	0	other	0.5	subcutances other days	high	medium	hard	-0.643869086
166	10	0	other	2.5	mascular once a weak	high	low	hard	0.00383736
166	10	1	iran	5.5	mascular once a weak	medium	medium	easy	-0.697626621
166	11	1	other	5.5	subcutances other days	high	medium	easy	-0.34108841
166	11	0	iran	0.5	subcutances three times a weak	medium	low	hard	-0.699689278
166	12	1	iran	5.5	subcutances three times a weak	high	medium	hard	-0.787756632
166	12	0	other	2.5	subcutances other days	medium	low	hard	-0.352164436
167	1	1	iran	5.5	mascular once a weak	high	medium	hard	-0.572088286
167	1	0	iran	0.5	subcutances three times a weak	high	medium	easy	-0.72867963
167	2	0	other	0.5	subcutances other days	high	medium	hard	-0.643869086
167	2	1	iran	5.5	subcutances other days	medium	medium	hard	-0.928090082
167	3	0	iran	5.5	mascular once a weak	high	medium	hard	-0.572088286
167	3	1	iran	2.5	subcutances three times a weak	high	medium	easy	-0.646894171
167	4	1	iran	0.5	subcutances three times a weak	medium	low	hard	-0.699689278
167	4	0	other	2.5	mascular once a weak	high	low	hard	0.00383736
167	5	1	iran	0.5	mascular once a weak	medium	low	easy	-0.303092094
167	5	0	other	2.5	subcutances three times a weak	high	low	easy	-0.030902146
167	6	0	other	2.5	subcutances three times a weak	high	low	easy	-0.030902146
167	6	1	other	0.5	subcutances other days	high	medium	easy	-0.462940247
167	7	0	other	0.5	mascular once a weak	medium	medium	hard	-0.900801637
167	7	0	other	5.5	subcutances other days	medium	low	hard	-0.312098058
167	8	1	iran	2.5	mascular once a weak	high	low	hard	-0.0957683
167	8	0	iran	5.5	subcutances three times a weak	high	low	easy	-0.090441429
167	9	1	iran	2.5	subcutances three times a weak	medium	low	hard	-0.617903819
167	9	0	other	0.5	subcutances other days	high	medium	hard	-0.643869086
167	10	0	other	2.5	mascular once a weak	high	low	hard	0.00383736
167	10	1	iran	5.5	mascular once a weak	medium	medium	easy	-0.697626621
167	11	0	other	5.5	subcutances other days	high	medium	easy	-0.34108841
167	11	1	iran	0.5	subcutances three times a weak	medium	low	hard	-0.699689278
167	12	0	iran	5.5	subcutances three times a weak	high	medium	hard	-0.787756632
167	12	1	other	2.5	subcutances other days	medium	low	hard	-0.352164436
168	1	0	iran	5.5	mascular once a weak	high	medium	hard	-0.572088286
168	1	1	iran	0.5	subcutances three times a weak	high	medium	easy	-0.72867963

168	2	1	other	0.5	subcutances other days	high	medium	hard	-0.643869086
168	2	0	iran	5.5	subcutances other days	medium	medium	hard	-0.928090082
168	3	0	iran	5.5	mascular once a weak	high	medium	hard	-0.572088286
168	3	1	iran	2.5	subcutances three times a weak	high	medium	easy	-0.646894171
168	4	0	iran	0.5	subcutances three times a weak	medium	low	hard	-0.699689278
168	4	1	other	2.5	mascular once a weak	high	low	hard	0.00383736
168	5	0	iran	0.5	mascular once a weak	medium	low	easy	-0.303092094
168	5	1	other	2.5	subcutances three times a weak	high	low	easy	-0.030902146
168	6	1	other	2.5	subcutances three times a weak	high	low	easy	-0.030902146
168	6	0	other	0.5	subcutances other days	high	medium	easy	-0.462940247
168	7	0	other	0.5	mascular once a weak	medium	medium	hard	-0.900801637
168	7	1	other	5.5	subcutances other days	medium	low	hard	-0.312098058
168	8	0	iran	2.5	mascular once a weak	high	low	hard	-0.0957683
168	8	1	iran	5.5	subcutances three times a weak	high	low	easy	-0.090441429
168	9	0	iran	2.5	subcutances three times a weak	medium	low	hard	-0.617903819
168	9	1	other	0.5	subcutances other days	high	medium	hard	-0.643869086
168	10	1	other	2.5	mascular once a weak	high	low	hard	0.00383736
168	10	0	iran	5.5	mascular once a weak	medium	medium	easy	-0.697626621
168	11	1	other	5.5	subcutances other days	high	medium	easy	-0.34108841
168	11	0	iran	0.5	subcutances three times a weak	medium	low	hard	-0.699689278
168	12	1	iran	5.5	subcutances three times a weak	high	medium	hard	-0.787756632
168	12	0	other	2.5	subcutances other days	medium	low	hard	-0.352164436
169	1	0	iran	5.5	mascular once a weak	high	medium	hard	-0.572088286
169	1	1	iran	0.5	subcutances three times a weak	high	medium	easy	-0.72867963
169	2	0	other	0.5	subcutances other days	high	medium	hard	-0.643869086
169	2	1	iran	5.5	subcutances other days	medium	medium	hard	-0.928090082
169	3	0	iran	5.5	mascular once a weak	high	medium	hard	-0.572088286
169	3	1	iran	2.5	subcutances three times a weak	high	medium	easy	-0.646894171
169	4	1	iran	0.5	subcutances three times a weak	medium	low	hard	-0.699689278
169	4	0	other	2.5	mascular once a weak	high	low	hard	0.00383736
169	5	1	iran	0.5	mascular once a weak	medium	low	easy	-0.303092094
169	5	0	other	2.5	subcutances three times a weak	high	low	easy	-0.030902146
169	6	0	other	2.5	subcutances three times a weak	high	low	easy	-0.030902146
169	6	1	other	0.5	subcutances other days	high	medium	easy	-0.462940247

169	7	0	other	0.5	mascular once a weak	medium	medium	hard	-0.900801637
169	7	0	other	5.5	subcutances other days	medium	low	hard	-0.312098058
169	8	1	iran	2.5	mascular once a weak	high	low	hard	-0.0957683
169	8	0	iran	5.5	subcutances three times a weak	high	low	easy	-0.090441429
169	9	1	iran	2.5	subcutances three times a weak	medium	low	hard	-0.617903819
169	9	0	other	0.5	subcutances other days	high	medium	hard	-0.643869086
169	10	0	other	2.5	mascular once a weak	high	low	hard	0.00383736
169	10	1	iran	5.5	mascular once a weak	medium	medium	easy	-0.697626621
169	11	0	other	5.5	subcutances other days	high	medium	easy	-0.34108841
169	11	1	iran	0.5	subcutances three times a weak	medium	low	hard	-0.699689278
169	12	0	iran	5.5	subcutances three times a weak	high	medium	hard	-0.787756632
169	12	1	other	2.5	subcutances other days	medium	low	hard	-0.352164436
170	1	1	iran	5.5	mascular once a weak	high	medium	hard	-0.572088286
170	1	0	iran	0.5	subcutances three times a weak	high	medium	easy	-0.72867963
170	2	0	other	0.5	subcutances other days	high	medium	hard	-0.643869086
170	2	1	iran	5.5	subcutances other days	medium	medium	hard	-0.928090082
170	3	0	iran	5.5	mascular once a weak	high	medium	hard	-0.572088286
170	3	1	iran	2.5	subcutances three times a weak	high	medium	easy	-0.646894171
170	4	1	iran	0.5	subcutances three times a weak	medium	low	hard	-0.699689278
170	4	0	other	2.5	mascular once a weak	high	low	hard	0.00383736
170	5	1	iran	0.5	mascular once a weak	medium	low	easy	-0.303092094
170	5	0	other	2.5	subcutances three times a weak	high	low	easy	-0.030902146
170	6	0	other	2.5	subcutances three times a weak	high	low	easy	-0.030902146
170	6	1	other	0.5	subcutances other days	high	medium	easy	-0.462940247
170	7	0	other	0.5	mascular once a weak	medium	medium	hard	-0.900801637
170	7	1	other	5.5	subcutances other days	medium	low	hard	-0.312098058
170	8	1	iran	2.5	mascular once a weak	high	low	hard	-0.0957683
170	8	0	iran	5.5	subcutances three times a weak	high	low	easy	-0.090441429
170	9	1	iran	2.5	subcutances three times a weak	medium	low	hard	-0.617903819
170	9	0	other	0.5	subcutances other days	high	medium	hard	-0.643869086
170	10	0	other	2.5	mascular once a weak	high	low	hard	0.00383736
170	10	1	iran	5.5	mascular once a weak	medium	medium	easy	-0.697626621
170	11	0	other	5.5	subcutances other days	high	medium	easy	-0.34108841
170	11	1	iran	0.5	subcutances three times a weak	medium	low	hard	-0.699689278

170	12	0	iran	5.5	subcutances three times a weak	high	medium	hard	-0.787756632
170	12	1	other	2.5	subcutances other days	medium	low	hard	-0.352164436
171	1	1	iran	5.5	mascular once a weak	high	medium	hard	-0.572088286
171	1	0	iran	0.5	subcutances three times a weak	high	medium	easy	-0.72867963
171	2	1	other	0.5	subcutances other days	high	medium	hard	-0.643869086
171	2	0	iran	5.5	subcutances other days	medium	medium	hard	-0.928090082
171	3	1	iran	5.5	mascular once a weak	high	medium	hard	-0.572088286
171	3	0	iran	2.5	subcutances three times a weak	high	medium	easy	-0.646894171
171	4	0	iran	0.5	subcutances three times a weak	medium	low	hard	-0.699689278
171	4	1	other	2.5	mascular once a weak	high	low	hard	0.00383736
171	5	0	iran	0.5	mascular once a weak	medium	low	easy	-0.303092094
171	5	1	other	2.5	subcutances three times a weak	high	low	easy	-0.030902146
171	6	0	other	2.5	subcutances three times a weak	high	low	easy	-0.030902146
171	6	1	other	0.5	subcutances other days	high	medium	easy	-0.462940247
171	7	1	other	0.5	mascular once a weak	medium	medium	hard	-0.900801637
171	7	0	other	5.5	subcutances other days	medium	low	hard	-0.312098058
171	8	1	iran	2.5	mascular once a weak	high	low	hard	-0.0957683
171	8	0	iran	5.5	subcutances three times a weak	high	low	easy	-0.090441429
171	9	0	iran	2.5	subcutances three times a weak	medium	low	hard	-0.617903819
171	9	1	other	0.5	subcutances other days	high	medium	hard	-0.643869086
171	10	1	other	2.5	mascular once a weak	high	low	hard	0.00383736
171	10	0	iran	5.5	mascular once a weak	medium	medium	easy	-0.697626621
171	11	1	other	5.5	subcutances other days	high	medium	easy	-0.34108841
171	11	0	iran	0.5	subcutances three times a weak	medium	low	hard	-0.699689278
171	12	1	iran	5.5	subcutances three times a weak	high	medium	hard	-0.787756632
171	12	0	other	2.5	subcutances other days	medium	low	hard	-0.352164436
172	1	0	iran	5.5	mascular once a weak	high	medium	hard	-0.572088286
172	1	0	iran	0.5	subcutances three times a weak	high	medium	easy	-0.72867963
172	2	1	other	0.5	subcutances other days	high	medium	hard	-0.643869086
172	2	0	iran	5.5	subcutances other days	medium	medium	hard	-0.928090082
172	3	0	iran	5.5	mascular once a weak	high	medium	hard	-0.572088286
172	3	0	iran	2.5	subcutances three times a weak	high	medium	easy	-0.646894171
172	4	0	iran	0.5	subcutances three times a weak	medium	low	hard	-0.699689278
172	4	1	other	2.5	mascular once a weak	high	low	hard	0.00383736

172	5	0	iran	0.5	mascular once a weak	medium	low	easy	-0.303092094
172	5	1	other	2.5	subcutances three times a weak	high	low	easy	-0.030902146
172	6	1	other	2.5	subcutances three times a weak	high	low	easy	-0.030902146
172	6	0	other	0.5	subcutances other days	high	medium	easy	-0.462940247
172	7	1	other	0.5	mascular once a weak	medium	medium	hard	-0.900801637
172	7	0	other	5.5	subcutances other days	medium	low	hard	-0.312098058
172	8	0	iran	2.5	mascular once a weak	high	low	hard	-0.0957683
172	8	0	iran	5.5	subcutances three times a weak	high	low	easy	-0.090441429
172	9	0	iran	2.5	subcutances three times a weak	medium	low	hard	-0.617903819
172	9	1	other	0.5	subcutances other days	high	medium	hard	-0.643869086
172	10	1	other	2.5	mascular once a weak	high	low	hard	0.00383736
172	10	0	iran	5.5	mascular once a weak	medium	medium	easy	-0.697626621
172	11	1	other	5.5	subcutances other days	high	medium	easy	-0.34108841
172	11	0	iran	0.5	subcutances three times a weak	medium	low	hard	-0.699689278
172	12	0	iran	5.5	subcutances three times a weak	high	medium	hard	-0.787756632
172	12	1	other	2.5	subcutances other days	medium	low	hard	-0.352164436
173	1	1	iran	5.5	mascular once a weak	high	medium	hard	-0.572088286
173	1	0	iran	0.5	subcutances three times a weak	high	medium	easy	-0.72867963
173	2	1	other	0.5	subcutances other days	high	medium	hard	-0.643869086
173	2	0	iran	5.5	subcutances other days	medium	medium	hard	-0.928090082
173	3	0	iran	5.5	mascular once a weak	high	medium	hard	-0.572088286
173	3	1	iran	2.5	subcutances three times a weak	high	medium	easy	-0.646894171
173	4	0	iran	0.5	subcutances three times a weak	medium	low	hard	-0.699689278
173	4	1	other	2.5	mascular once a weak	high	low	hard	0.00383736
173	5	0	iran	0.5	mascular once a weak	medium	low	easy	-0.303092094
173	5	1	other	2.5	subcutances three times a weak	high	low	easy	-0.030902146
173	6	0	other	2.5	subcutances three times a weak	high	low	easy	-0.030902146
173	6	1	other	0.5	subcutances other days	high	medium	easy	-0.462940247
173	7	0	other	0.5	mascular once a weak	medium	medium	hard	-0.900801637
173	7	1	other	5.5	subcutances other days	medium	low	hard	-0.312098058
173	8	1	iran	2.5	mascular once a weak	high	low	hard	-0.0957683
173	8	0	iran	5.5	subcutances three times a weak	high	low	easy	-0.090441429
173	9	0	iran	2.5	subcutances three times a weak	medium	low	hard	-0.617903819
173	9	1	other	0.5	subcutances other days	high	medium	hard	-0.643869086

173	10	1	other	2.5	mascular once a weak	high	low	hard	0.00383736
173	10	0	iran	5.5	mascular once a weak	medium	medium	easy	-0.697626621
173	11	1	other	5.5	subcutances other days	high	medium	easy	-0.34108841
173	11	0	iran	0.5	subcutances three times a weak	medium	low	hard	-0.699689278
173	12	1	iran	5.5	subcutances three times a weak	high	medium	hard	-0.787756632
173	12	0	other	2.5	subcutances other days	medium	low	hard	-0.352164436
174	1	1	iran	5.5	mascular once a weak	high	medium	hard	-0.572088286
174	1	0	iran	0.5	subcutances three times a weak	high	medium	easy	-0.72867963
174	2	1	other	0.5	subcutances other days	high	medium	hard	-0.643869086
174	2	0	iran	5.5	subcutances other days	medium	medium	hard	-0.928090082
174	3	1	iran	5.5	mascular once a weak	high	medium	hard	-0.572088286
174	3	0	iran	2.5	subcutances three times a weak	high	medium	easy	-0.646894171
174	4	0	iran	0.5	subcutances three times a weak	medium	low	hard	-0.699689278
174	4	1	other	2.5	mascular once a weak	high	low	hard	0.00383736
174	5	0	iran	0.5	mascular once a weak	medium	low	easy	-0.303092094
174	5	1	other	2.5	subcutances three times a weak	high	low	easy	-0.030902146
174	6	1	other	2.5	subcutances three times a weak	high	low	easy	-0.030902146
174	6	0	other	0.5	subcutances other days	high	medium	easy	-0.462940247
174	7	0	other	0.5	mascular once a weak	medium	medium	hard	-0.900801637
174	7	1	other	5.5	subcutances other days	medium	low	hard	-0.312098058
174	8	1	iran	2.5	mascular once a weak	high	low	hard	-0.0957683
174	8	0	iran	5.5	subcutances three times a weak	high	low	easy	-0.090441429
174	9	0	iran	2.5	subcutances three times a weak	medium	low	hard	-0.617903819
174	9	1	other	0.5	subcutances other days	high	medium	hard	-0.643869086
174	10	1	other	2.5	mascular once a weak	high	low	hard	0.00383736
174	10	0	iran	5.5	mascular once a weak	medium	medium	easy	-0.697626621
174	11	1	other	5.5	subcutances other days	high	medium	easy	-0.34108841
174	11	0	iran	0.5	subcutances three times a weak	medium	low	hard	-0.699689278
174	12	1	iran	5.5	subcutances three times a weak	high	medium	hard	-0.787756632
174	12	0	other	2.5	subcutances other days	medium	low	hard	-0.352164436
175	1	0	iran	5.5	mascular once a weak	high	medium	hard	-0.572088286
175	1	0	iran	0.5	subcutances three times a weak	high	medium	easy	-0.72867963
175	2	1	other	0.5	subcutances other days	high	medium	hard	-0.643869086
175	2	0	iran	5.5	subcutances other days	medium	medium	hard	-0.928090082

175	3	0	iran	5.5	mascular once a weak	high	medium	hard	-0.572088286
175	3	0	iran	2.5	subcutances three times a weak	high	medium	easy	-0.646894171
175	4	0	iran	0.5	subcutances three times a weak	medium	low	hard	-0.699689278
175	4	1	other	2.5	mascular once a weak	high	low	hard	0.00383736
175	5	0	iran	0.5	mascular once a weak	medium	low	easy	-0.303092094
175	5	1	other	2.5	subcutances three times a weak	high	low	easy	-0.030902146
175	6	1	other	2.5	subcutances three times a weak	high	low	easy	-0.030902146
175	6	0	other	0.5	subcutances other days	high	medium	easy	-0.462940247
175	7	0	other	0.5	mascular once a weak	medium	medium	hard	-0.900801637
175	7	1	other	5.5	subcutances other days	medium	low	hard	-0.312098058
175	8	0	iran	2.5	mascular once a weak	high	low	hard	-0.0957683
175	8	0	iran	5.5	subcutances three times a weak	high	low	easy	-0.090441429
175	9	0	iran	2.5	subcutances three times a weak	medium	low	hard	-0.617903819
175	9	1	other	0.5	subcutances other days	high	medium	hard	-0.643869086
175	10	1	other	2.5	mascular once a weak	high	low	hard	0.00383736
175	10	0	iran	5.5	mascular once a weak	medium	medium	easy	-0.697626621
175	11	1	other	5.5	subcutances other days	high	medium	easy	-0.34108841
175	11	0	iran	0.5	subcutances three times a weak	medium	low	hard	-0.699689278
175	12	0	iran	5.5	subcutances three times a weak	high	medium	hard	-0.787756632
175	12	1	other	2.5	subcutances other days	medium	low	hard	-0.352164436
176	1	1	iran	5.5	mascular once a weak	high	medium	hard	-0.572088286
176	1	0	iran	0.5	subcutances three times a weak	high	medium	easy	-0.72867963
176	2	1	other	0.5	subcutances other days	high	medium	hard	-0.643869086
176	2	0	iran	5.5	subcutances other days	medium	medium	hard	-0.928090082
176	3	1	iran	5.5	mascular once a weak	high	medium	hard	-0.572088286
176	3	0	iran	2.5	subcutances three times a weak	high	medium	easy	-0.646894171
176	4	0	iran	0.5	subcutances three times a weak	medium	low	hard	-0.699689278
176	4	1	other	2.5	mascular once a weak	high	low	hard	0.00383736
176	5	0	iran	0.5	mascular once a weak	medium	low	easy	-0.303092094
176	5	1	other	2.5	subcutances three times a weak	high	low	easy	-0.030902146
176	6	1	other	2.5	subcutances three times a weak	high	low	easy	-0.030902146
176	6	0	other	0.5	subcutances other days	high	medium	easy	-0.462940247
176	7	0	other	0.5	mascular once a weak	medium	medium	hard	-0.900801637
176	7	1	other	5.5	subcutances other days	medium	low	hard	-0.312098058

176	8	1	iran	2.5	mascular once a weak	high	low	hard	-0.0957683
176	8	0	iran	5.5	subcutances three times a weak	high	low	easy	-0.090441429
176	9	0	iran	2.5	subcutances three times a weak	medium	low	hard	-0.617903819
176	9	1	other	0.5	subcutances other days	high	medium	hard	-0.643869086
176	10	1	other	2.5	mascular once a weak	high	low	hard	0.00383736
176	10	0	iran	5.5	mascular once a weak	medium	medium	easy	-0.697626621
176	11	1	other	5.5	subcutances other days	high	medium	easy	-0.34108841
176	11	0	iran	0.5	subcutances three times a weak	medium	low	hard	-0.699689278
176	12	1	iran	5.5	subcutances three times a weak	high	medium	hard	-0.787756632
176	12	0	other	2.5	subcutances other days	medium	low	hard	-0.352164436
177	1	0	iran	5.5	mascular once a weak	high	medium	hard	-0.572088286
177	1	1	iran	0.5	subcutances three times a weak	high	medium	easy	-0.72867963
177	2	1	other	0.5	subcutances other days	high	medium	hard	-0.643869086
177	2	0	iran	5.5	subcutances other days	medium	medium	hard	-0.928090082
177	3	0	iran	5.5	mascular once a weak	high	medium	hard	-0.572088286
177	3	1	iran	2.5	subcutances three times a weak	high	medium	easy	-0.646894171
177	4	0	iran	0.5	subcutances three times a weak	medium	low	hard	-0.699689278
177	4	1	other	2.5	mascular once a weak	high	low	hard	0.00383736
177	5	0	iran	0.5	mascular once a weak	medium	low	easy	-0.303092094
177	5	1	other	2.5	subcutances three times a weak	high	low	easy	-0.030902146
177	6	1	other	2.5	subcutances three times a weak	high	low	easy	-0.030902146
177	6	0	other	0.5	subcutances other days	high	medium	easy	-0.462940247
177	7	0	other	0.5	mascular once a weak	medium	medium	hard	-0.900801637
177	7	1	other	5.5	subcutances other days	medium	low	hard	-0.312098058
177	8	0	iran	2.5	mascular once a weak	high	low	hard	-0.0957683
177	8	1	iran	5.5	subcutances three times a weak	high	low	easy	-0.090441429
177	9	0	iran	2.5	subcutances three times a weak	medium	low	hard	-0.617903819
177	9	1	other	0.5	subcutances other days	high	medium	hard	-0.643869086
177	10	1	other	2.5	mascular once a weak	high	low	hard	0.00383736
177	10	0	iran	5.5	mascular once a weak	medium	medium	easy	-0.697626621
177	11	1	other	5.5	subcutances other days	high	medium	easy	-0.34108841
177	11	0	iran	0.5	subcutances three times a weak	medium	low	hard	-0.699689278
177	12	0	iran	5.5	subcutances three times a weak	high	medium	hard	-0.787756632
177	12	1	other	2.5	subcutances other days	medium	low	hard	-0.352164436

178	1	0	iran	5.5	mascular once a weak	high	medium	hard	-0.572088286
178	1	1	iran	0.5	subcutances three times a weak	high	medium	easy	-0.72867963
178	2	1	other	0.5	subcutances other days	high	medium	hard	-0.643869086
178	2	0	iran	5.5	subcutances other days	medium	medium	hard	-0.928090082
178	3	0	iran	5.5	mascular once a weak	high	medium	hard	-0.572088286
178	3	1	iran	2.5	subcutances three times a weak	high	medium	easy	-0.646894171
178	4	0	iran	0.5	subcutances three times a weak	medium	low	hard	-0.699689278
178	4	1	other	2.5	mascular once a weak	high	low	hard	0.00383736
178	5	1	iran	0.5	mascular once a weak	medium	low	easy	-0.303092094
178	5	0	other	2.5	subcutances three times a weak	high	low	easy	-0.030902146
178	6	0	other	2.5	subcutances three times a weak	high	low	easy	-0.030902146
178	6	1	other	0.5	subcutances other days	high	medium	easy	-0.462940247
178	7	0	other	0.5	mascular once a weak	medium	medium	hard	-0.900801637
178	7	1	other	5.5	subcutances other days	medium	low	hard	-0.312098058
178	8	0	iran	2.5	mascular once a weak	high	low	hard	-0.0957683
178	8	1	iran	5.5	subcutances three times a weak	high	low	easy	-0.090441429
178	9	1	iran	2.5	subcutances three times a weak	medium	low	hard	-0.617903819
178	9	0	other	0.5	subcutances other days	high	medium	hard	-0.643869086
178	10	1	other	2.5	mascular once a weak	high	low	hard	0.00383736
178	10	0	iran	5.5	mascular once a weak	medium	medium	easy	-0.697626621
178	11	1	other	5.5	subcutances other days	high	medium	easy	-0.34108841
178	11	0	iran	0.5	subcutances three times a weak	medium	low	hard	-0.699689278
178	12	0	iran	5.5	subcutances three times a weak	high	medium	hard	-0.787756632
178	12	1	other	2.5	subcutances other days	medium	low	hard	-0.352164436
179	1	0	iran	5.5	mascular once a weak	high	medium	hard	-0.572088286
179	1	1	iran	0.5	subcutances three times a weak	high	medium	easy	-0.72867963
179	2	1	other	0.5	subcutances other days	high	medium	hard	-0.643869086
179	2	0	iran	5.5	subcutances other days	medium	medium	hard	-0.928090082
179	3	0	iran	5.5	mascular once a weak	high	medium	hard	-0.572088286
179	3	1	iran	2.5	subcutances three times a weak	high	medium	easy	-0.646894171
179	4	0	iran	0.5	subcutances three times a weak	medium	low	hard	-0.699689278
179	4	1	other	2.5	mascular once a weak	high	low	hard	0.00383736
179	5	0	iran	0.5	mascular once a weak	medium	low	easy	-0.303092094
179	5	1	other	2.5	subcutances three times a weak	high	low	easy	-0.030902146

179	6	0	other	2.5	subcutances three times a weak	high	low	easy	-0.030902146
179	6	1	other	0.5	subcutances other days	high	medium	easy	-0.462940247
179	7	0	other	0.5	mascular once a weak	medium	medium	hard	-0.900801637
179	7	1	other	5.5	subcutances other days	medium	low	hard	-0.312098058
179	8	0	iran	2.5	mascular once a weak	high	low	hard	-0.0957683
179	8	1	iran	5.5	subcutances three times a weak	high	low	easy	-0.090441429
179	9	0	iran	2.5	subcutances three times a weak	medium	low	hard	-0.617903819
179	9	1	other	0.5	subcutances other days	high	medium	hard	-0.643869086
179	10	1	other	2.5	mascular once a weak	high	low	hard	0.00383736
179	10	0	iran	5.5	mascular once a weak	medium	medium	easy	-0.697626621
179	11	1	other	5.5	subcutances other days	high	medium	easy	-0.34108841
179	11	0	iran	0.5	subcutances three times a weak	medium	low	hard	-0.699689278
179	12	1	iran	5.5	subcutances three times a weak	high	medium	hard	-0.787756632
179	12	0	other	2.5	subcutances other days	medium	low	hard	-0.352164436
180	1	0	iran	5.5	mascular once a weak	high	medium	hard	-0.572088286
180	1	1	iran	0.5	subcutances three times a weak	high	medium	easy	-0.72867963
180	2	1	other	0.5	subcutances other days	high	medium	hard	-0.643869086
180	2	0	iran	5.5	subcutances other days	medium	medium	hard	-0.928090082
180	3	0	iran	5.5	mascular once a weak	high	medium	hard	-0.572088286
180	3	1	iran	2.5	subcutances three times a weak	high	medium	easy	-0.646894171
180	4	0	iran	0.5	subcutances three times a weak	medium	low	hard	-0.699689278
180	4	1	other	2.5	mascular once a weak	high	low	hard	0.00383736
180	5	0	iran	0.5	mascular once a weak	medium	low	easy	-0.303092094
180	5	1	other	2.5	subcutances three times a weak	high	low	easy	-0.030902146
180	6	1	other	2.5	subcutances three times a weak	high	low	easy	-0.030902146
180	6	0	other	0.5	subcutances other days	high	medium	easy	-0.462940247
180	7	0	other	0.5	mascular once a weak	medium	medium	hard	-0.900801637
180	7	1	other	5.5	subcutances other days	medium	low	hard	-0.312098058
180	8	0	iran	2.5	mascular once a weak	high	low	hard	-0.0957683
180	8	1	iran	5.5	subcutances three times a weak	high	low	easy	-0.090441429
180	9	0	iran	2.5	subcutances three times a weak	medium	low	hard	-0.617903819
180	9	1	other	0.5	subcutances other days	high	medium	hard	-0.643869086
180	10	1	other	2.5	mascular once a weak	high	low	hard	0.00383736
180	10	0	iran	5.5	mascular once a weak	medium	medium	easy	-0.697626621

180	11	1	other	5.5	subcutances other days	high	medium	easy	-0.34108841
180	11	0	iran	0.5	subcutances three times a weak	medium	low	hard	-0.699689278
180	12	1	iran	5.5	subcutances three times a weak	high	medium	hard	-0.787756632
180	12	0	other	2.5	subcutances other days	medium	low	hard	-0.352164436
181	1	1	iran	5.5	mascular once a weak	high	medium	hard	-0.572088286
181	1	0	iran	0.5	subcutances three times a weak	high	medium	easy	-0.72867963
181	2	0	other	0.5	subcutances other days	high	medium	hard	-0.643869086
181	2	1	iran	5.5	subcutances other days	medium	medium	hard	-0.928090082
181	3	0	iran	5.5	mascular once a weak	high	medium	hard	-0.572088286
181	3	1	iran	2.5	subcutances three times a weak	high	medium	easy	-0.646894171
181	4	0	iran	0.5	subcutances three times a weak	medium	low	hard	-0.699689278
181	4	1	other	2.5	mascular once a weak	high	low	hard	0.00383736
181	5	0	iran	0.5	mascular once a weak	medium	low	easy	-0.303092094
181	5	1	other	2.5	subcutances three times a weak	high	low	easy	-0.030902146
181	6	0	other	2.5	subcutances three times a weak	high	low	easy	-0.030902146
181	6	1	other	0.5	subcutances other days	high	medium	easy	-0.462940247
181	7	0	other	0.5	mascular once a weak	medium	medium	hard	-0.900801637
181	7	1	other	5.5	subcutances other days	medium	low	hard	-0.312098058
181	8	1	iran	2.5	mascular once a weak	high	low	hard	-0.0957683
181	8	0	iran	5.5	subcutances three times a weak	high	low	easy	-0.090441429
181	9	0	iran	2.5	subcutances three times a weak	medium	low	hard	-0.617903819
181	9	1	other	0.5	subcutances other days	high	medium	hard	-0.643869086
181	10	0	other	2.5	mascular once a weak	high	low	hard	0.00383736
181	10	1	iran	5.5	mascular once a weak	medium	medium	easy	-0.697626621
181	11	1	other	5.5	subcutances other days	high	medium	easy	-0.34108841
181	11	0	iran	0.5	subcutances three times a weak	medium	low	hard	-0.699689278
181	12	1	iran	5.5	subcutances three times a weak	high	medium	hard	-0.787756632
181	12	0	other	2.5	subcutances other days	medium	low	hard	-0.352164436
182	1	1	iran	5.5	mascular once a weak	high	medium	hard	-0.572088286
182	1	0	iran	0.5	subcutances three times a weak	high	medium	easy	-0.72867963
182	2	1	other	0.5	subcutances other days	high	medium	hard	-0.643869086
182	2	0	iran	5.5	subcutances other days	medium	medium	hard	-0.928090082
182	3	0	iran	5.5	mascular once a weak	high	medium	hard	-0.572088286
182	3	1	iran	2.5	subcutances three times a weak	high	medium	easy	-0.646894171

182	4	0	iran	0.5	subcutances three times a weak	medium	low	hard	-0.699689278
182	4	1	other	2.5	mascular once a weak	high	low	hard	0.00383736
182	5	0	iran	0.5	mascular once a weak	medium	low	easy	-0.303092094
182	5	1	other	2.5	subcutances three times a weak	high	low	easy	-0.030902146
182	6	0	other	2.5	subcutances three times a weak	high	low	easy	-0.030902146
182	6	1	other	0.5	subcutances other days	high	medium	easy	-0.462940247
182	7	0	other	0.5	mascular once a weak	medium	medium	hard	-0.900801637
182	7	1	other	5.5	subcutances other days	medium	low	hard	-0.312098058
182	8	1	iran	2.5	mascular once a weak	high	low	hard	-0.0957683
182	8	0	iran	5.5	subcutances three times a weak	high	low	easy	-0.090441429
182	9	1	iran	2.5	subcutances three times a weak	medium	low	hard	-0.617903819
182	9	0	other	0.5	subcutances other days	high	medium	hard	-0.643869086
182	10	0	other	2.5	mascular once a weak	high	low	hard	0.00383736
182	10	1	iran	5.5	mascular once a weak	medium	medium	easy	-0.697626621
182	11	0	other	5.5	subcutances other days	high	medium	easy	-0.34108841
182	11	1	iran	0.5	subcutances three times a weak	medium	low	hard	-0.699689278
182	12	0	iran	5.5	subcutances three times a weak	high	medium	hard	-0.787756632
182	12	1	other	2.5	subcutances other days	medium	low	hard	-0.352164436
183	1	1	iran	5.5	mascular once a weak	high	medium	hard	-0.572088286
183	1	0	iran	0.5	subcutances three times a weak	high	medium	easy	-0.72867963
183	2	0	other	0.5	subcutances other days	high	medium	hard	-0.643869086
183	2	1	iran	5.5	subcutances other days	medium	medium	hard	-0.928090082
183	3	1	iran	5.5	mascular once a weak	high	medium	hard	-0.572088286
183	3	0	iran	2.5	subcutances three times a weak	high	medium	easy	-0.646894171
183	4	0	iran	0.5	subcutances three times a weak	medium	low	hard	-0.699689278
183	4	1	other	2.5	mascular once a weak	high	low	hard	0.00383736
183	5	0	iran	0.5	mascular once a weak	medium	low	easy	-0.303092094
183	5	1	other	2.5	subcutances three times a weak	high	low	easy	-0.030902146
183	6	1	other	2.5	subcutances three times a weak	high	low	easy	-0.030902146
183	6	0	other	0.5	subcutances other days	high	medium	easy	-0.462940247
183	7	1	other	0.5	mascular once a weak	medium	medium	hard	-0.900801637
183	7	0	other	5.5	subcutances other days	medium	low	hard	-0.312098058
183	8	1	iran	2.5	mascular once a weak	high	low	hard	-0.0957683
183	8	0	iran	5.5	subcutances three times a weak	high	low	easy	-0.090441429

183	9	1	iran	2.5	subcutances three times a weak	medium	low	hard	-0.617903819
183	9	0	other	0.5	subcutances other days	high	medium	hard	-0.643869086
183	10	1	other	2.5	mascular once a weak	high	low	hard	0.00383736
183	10	0	iran	5.5	mascular once a weak	medium	medium	easy	-0.697626621
183	11	0	other	5.5	subcutances other days	high	medium	easy	-0.34108841
183	11	1	iran	0.5	subcutances three times a weak	medium	low	hard	-0.699689278
183	12	1	iran	5.5	subcutances three times a weak	high	medium	hard	-0.787756632
183	12	0	other	2.5	subcutances other days	medium	low	hard	-0.352164436
184	1	1	iran	5.5	mascular once a weak	high	medium	hard	-0.572088286
184	1	0	iran	0.5	subcutances three times a weak	high	medium	easy	-0.72867963
184	2	1	other	0.5	subcutances other days	high	medium	hard	-0.643869086
184	2	0	iran	5.5	subcutances other days	medium	medium	hard	-0.928090082
184	3	0	iran	5.5	mascular once a weak	high	medium	hard	-0.572088286
184	3	1	iran	2.5	subcutances three times a weak	high	medium	easy	-0.646894171
184	4	0	iran	0.5	subcutances three times a weak	medium	low	hard	-0.699689278
184	4	1	other	2.5	mascular once a weak	high	low	hard	0.00383736
184	5	0	iran	0.5	mascular once a weak	medium	low	easy	-0.303092094
184	5	1	other	2.5	subcutances three times a weak	high	low	easy	-0.030902146
184	6	1	other	2.5	subcutances three times a weak	high	low	easy	-0.030902146
184	6	0	other	0.5	subcutances other days	high	medium	easy	-0.462940247
184	7	0	other	0.5	mascular once a weak	medium	medium	hard	-0.900801637
184	7	1	other	5.5	subcutances other days	medium	low	hard	-0.312098058
184	8	1	iran	2.5	mascular once a weak	high	low	hard	-0.0957683
184	8	0	iran	5.5	subcutances three times a weak	high	low	easy	-0.090441429
184	9	1	iran	2.5	subcutances three times a weak	medium	low	hard	-0.617903819
184	9	0	other	0.5	subcutances other days	high	medium	hard	-0.643869086
184	10	1	other	2.5	mascular once a weak	high	low	hard	0.00383736
184	10	0	iran	5.5	mascular once a weak	medium	medium	easy	-0.697626621
184	11	0	other	5.5	subcutances other days	high	medium	easy	-0.34108841
184	11	1	iran	0.5	subcutances three times a weak	medium	low	hard	-0.699689278
184	12	0	iran	5.5	subcutances three times a weak	high	medium	hard	-0.787756632
184	12	1	other	2.5	subcutances other days	medium	low	hard	-0.352164436
185	1	1	iran	5.5	mascular once a weak	high	medium	hard	-0.572088286
185	1	0	iran	0.5	subcutances three times a weak	high	medium	easy	-0.72867963

185	2	0	other	0.5	subcutances other days	high	medium	hard	-0.643869086
185	2	1	iran	5.5	subcutances other days	medium	medium	hard	-0.928090082
185	3	0	iran	5.5	mascular once a weak	high	medium	hard	-0.572088286
185	3	1	iran	2.5	subcutances three times a weak	high	medium	easy	-0.646894171
185	4	0	iran	0.5	subcutances three times a weak	medium	low	hard	-0.699689278
185	4	1	other	2.5	mascular once a weak	high	low	hard	0.00383736
185	5	0	iran	0.5	mascular once a weak	medium	low	easy	-0.303092094
185	5	1	other	2.5	subcutances three times a weak	high	low	easy	-0.030902146
185	6	0	other	2.5	subcutances three times a weak	high	low	easy	-0.030902146
185	6	1	other	0.5	subcutances other days	high	medium	easy	-0.462940247
185	7	0	other	0.5	mascular once a weak	medium	medium	hard	-0.900801637
185	7	0	other	5.5	subcutances other days	medium	low	hard	-0.312098058
185	8	1	iran	2.5	mascular once a weak	high	low	hard	-0.0957683
185	8	0	iran	5.5	subcutances three times a weak	high	low	easy	-0.090441429
185	9	0	iran	2.5	subcutances three times a weak	medium	low	hard	-0.617903819
185	9	1	other	0.5	subcutances other days	high	medium	hard	-0.643869086
185	10	0	other	2.5	mascular once a weak	high	low	hard	0.00383736
185	10	1	iran	5.5	mascular once a weak	medium	medium	easy	-0.697626621
185	11	0	other	5.5	subcutances other days	high	medium	easy	-0.34108841
185	11	1	iran	0.5	subcutances three times a weak	medium	low	hard	-0.699689278
185	12	0	iran	5.5	subcutances three times a weak	high	medium	hard	-0.787756632
185	12	1	other	2.5	subcutances other days	medium	low	hard	-0.352164436
186	1	0	iran	5.5	mascular once a weak	high	medium	hard	-0.572088286
186	1	1	iran	0.5	subcutances three times a weak	high	medium	easy	-0.72867963
186	2	1	other	0.5	subcutances other days	high	medium	hard	-0.643869086
186	2	0	iran	5.5	subcutances other days	medium	medium	hard	-0.928090082
186	3	0	iran	5.5	mascular once a weak	high	medium	hard	-0.572088286
186	3	1	iran	2.5	subcutances three times a weak	high	medium	easy	-0.646894171
186	4	0	iran	0.5	subcutances three times a weak	medium	low	hard	-0.699689278
186	4	1	other	2.5	mascular once a weak	high	low	hard	0.00383736
186	5	0	iran	0.5	mascular once a weak	medium	low	easy	-0.303092094
186	5	1	other	2.5	subcutances three times a weak	high	low	easy	-0.030902146
186	6	0	other	2.5	subcutances three times a weak	high	low	easy	-0.030902146
186	6	1	other	0.5	subcutances other days	high	medium	easy	-0.462940247

186	7	0	other	0.5	mascular once a weak	medium	medium	hard	-0.900801637
186	7	1	other	5.5	subcutances other days	medium	low	hard	-0.312098058
186	8	1	iran	2.5	mascular once a weak	high	low	hard	-0.0957683
186	8	0	iran	5.5	subcutances three times a weak	high	low	easy	-0.090441429
186	9	0	iran	2.5	subcutances three times a weak	medium	low	hard	-0.617903819
186	9	1	other	0.5	subcutances other days	high	medium	hard	-0.643869086
186	10	1	other	2.5	mascular once a weak	high	low	hard	0.00383736
186	10	0	iran	5.5	mascular once a weak	medium	medium	easy	-0.697626621
186	11	1	other	5.5	subcutances other days	high	medium	easy	-0.34108841
186	11	0	iran	0.5	subcutances three times a weak	medium	low	hard	-0.699689278
186	12	1	iran	5.5	subcutances three times a weak	high	medium	hard	-0.787756632
186	12	0	other	2.5	subcutances other days	medium	low	hard	-0.352164436
187	1	0	iran	5.5	mascular once a weak	high	medium	hard	-0.572088286
187	1	1	iran	0.5	subcutances three times a weak	high	medium	easy	-0.72867963
187	2	1	other	0.5	subcutances other days	high	medium	hard	-0.643869086
187	2	0	iran	5.5	subcutances other days	medium	medium	hard	-0.928090082
187	3	0	iran	5.5	mascular once a weak	high	medium	hard	-0.572088286
187	3	1	iran	2.5	subcutances three times a weak	high	medium	easy	-0.646894171
187	4	0	iran	0.5	subcutances three times a weak	medium	low	hard	-0.699689278
187	4	1	other	2.5	mascular once a weak	high	low	hard	0.00383736
187	5	0	iran	0.5	mascular once a weak	medium	low	easy	-0.303092094
187	5	1	other	2.5	subcutances three times a weak	high	low	easy	-0.030902146
187	6	1	other	2.5	subcutances three times a weak	high	low	easy	-0.030902146
187	6	0	other	0.5	subcutances other days	high	medium	easy	-0.462940247
187	7	0	other	0.5	mascular once a weak	medium	medium	hard	-0.900801637
187	7	1	other	5.5	subcutances other days	medium	low	hard	-0.312098058
187	8	0	iran	2.5	mascular once a weak	high	low	hard	-0.0957683
187	8	1	iran	5.5	subcutances three times a weak	high	low	easy	-0.090441429
187	9	0	iran	2.5	subcutances three times a weak	medium	low	hard	-0.617903819
187	9	1	other	0.5	subcutances other days	high	medium	hard	-0.643869086
187	10	1	other	2.5	mascular once a weak	high	low	hard	0.00383736
187	10	0	iran	5.5	mascular once a weak	medium	medium	easy	-0.697626621
187	11	1	other	5.5	subcutances other days	high	medium	easy	-0.34108841
187	11	0	iran	0.5	subcutances three times a weak	medium	low	hard	-0.699689278

187	12	1	iran	5.5	subcutances three times a weak	high	medium	hard	-0.787756632
187	12	0	other	2.5	subcutances other days	medium	low	hard	-0.352164436
188	1	0	iran	5.5	mascular once a weak	high	medium	hard	-0.572088286
188	1	1	iran	0.5	subcutances three times a weak	high	medium	easy	-0.72867963
188	2	1	other	0.5	subcutances other days	high	medium	hard	-0.643869086
188	2	0	iran	5.5	subcutances other days	medium	medium	hard	-0.928090082
188	3	0	iran	5.5	mascular once a weak	high	medium	hard	-0.572088286
188	3	1	iran	2.5	subcutances three times a weak	high	medium	easy	-0.646894171
188	4	0	iran	0.5	subcutances three times a weak	medium	low	hard	-0.699689278
188	4	1	other	2.5	mascular once a weak	high	low	hard	0.00383736
188	5	0	iran	0.5	mascular once a weak	medium	low	easy	-0.303092094
188	5	1	other	2.5	subcutances three times a weak	high	low	easy	-0.030902146
188	6	0	other	2.5	subcutances three times a weak	high	low	easy	-0.030902146
188	6	1	other	0.5	subcutances other days	high	medium	easy	-0.462940247
188	7	0	other	0.5	mascular once a weak	medium	medium	hard	-0.900801637
188	7	1	other	5.5	subcutances other days	medium	low	hard	-0.312098058
188	8	0	iran	2.5	mascular once a weak	high	low	hard	-0.0957683
188	8	1	iran	5.5	subcutances three times a weak	high	low	easy	-0.090441429
188	9	0	iran	2.5	subcutances three times a weak	medium	low	hard	-0.617903819
188	9	1	other	0.5	subcutances other days	high	medium	hard	-0.643869086
188	10	1	other	2.5	mascular once a weak	high	low	hard	0.00383736
188	10	0	iran	5.5	mascular once a weak	medium	medium	easy	-0.697626621
188	11	1	other	5.5	subcutances other days	high	medium	easy	-0.34108841
188	11	0	iran	0.5	subcutances three times a weak	medium	low	hard	-0.699689278
188	12	1	iran	5.5	subcutances three times a weak	high	medium	hard	-0.787756632
188	12	0	other	2.5	subcutances other days	medium	low	hard	-0.352164436
189	1	0	iran	5.5	mascular once a weak	high	medium	hard	-0.572088286
189	1	0	iran	0.5	subcutances three times a weak	high	medium	easy	-0.72867963
189	2	1	other	0.5	subcutances other days	high	medium	hard	-0.643869086
189	2	0	iran	5.5	subcutances other days	medium	medium	hard	-0.928090082
189	3	0	iran	5.5	mascular once a weak	high	medium	hard	-0.572088286
189	3	0	iran	2.5	subcutances three times a weak	high	medium	easy	-0.646894171
189	4	0	iran	0.5	subcutances three times a weak	medium	low	hard	-0.699689278
189	4	1	other	2.5	mascular once a weak	high	low	hard	0.00383736

189	5	0	iran	0.5	mascular once a weak	medium	low	easy	-0.303092094
189	5	1	other	2.5	subcutances three times a weak	high	low	easy	-0.030902146
189	6	1	other	2.5	subcutances three times a weak	high	low	easy	-0.030902146
189	6	0	other	0.5	subcutances other days	high	medium	easy	-0.462940247
189	7	0	other	0.5	mascular once a weak	medium	medium	hard	-0.900801637
189	7	1	other	5.5	subcutances other days	medium	low	hard	-0.312098058
189	8	0	iran	2.5	mascular once a weak	high	low	hard	-0.0957683
189	8	0	iran	5.5	subcutances three times a weak	high	low	easy	-0.090441429
189	9	0	iran	2.5	subcutances three times a weak	medium	low	hard	-0.617903819
189	9	1	other	0.5	subcutances other days	high	medium	hard	-0.643869086
189	10	1	other	2.5	mascular once a weak	high	low	hard	0.00383736
189	10	0	iran	5.5	mascular once a weak	medium	medium	easy	-0.697626621
189	11	1	other	5.5	subcutances other days	high	medium	easy	-0.34108841
189	11	0	iran	0.5	subcutances three times a weak	medium	low	hard	-0.699689278
189	12	0	iran	5.5	subcutances three times a weak	high	medium	hard	-0.787756632
189	12	1	other	2.5	subcutances other days	medium	low	hard	-0.352164436
190	1	0	iran	5.5	mascular once a weak	high	medium	hard	-0.572088286
190	1	0	iran	0.5	subcutances three times a weak	high	medium	easy	-0.72867963
190	2	1	other	0.5	subcutances other days	high	medium	hard	-0.643869086
190	2	0	iran	5.5	subcutances other days	medium	medium	hard	-0.928090082
190	3	0	iran	5.5	mascular once a weak	high	medium	hard	-0.572088286
190	3	1	iran	2.5	subcutances three times a weak	high	medium	easy	-0.646894171
190	4	0	iran	0.5	subcutances three times a weak	medium	low	hard	-0.699689278
190	4	1	other	2.5	mascular once a weak	high	low	hard	0.00383736
190	5	0	iran	0.5	mascular once a weak	medium	low	easy	-0.303092094
190	5	1	other	2.5	subcutances three times a weak	high	low	easy	-0.030902146
190	6	0	other	2.5	subcutances three times a weak	high	low	easy	-0.030902146
190	6	1	other	0.5	subcutances other days	high	medium	easy	-0.462940247
190	7	0	other	0.5	mascular once a weak	medium	medium	hard	-0.900801637
190	7	1	other	5.5	subcutances other days	medium	low	hard	-0.312098058
190	8	1	iran	2.5	mascular once a weak	high	low	hard	-0.0957683
190	8	0	iran	5.5	subcutances three times a weak	high	low	easy	-0.090441429
190	9	0	iran	2.5	subcutances three times a weak	medium	low	hard	-0.617903819
190	9	1	other	0.5	subcutances other days	high	medium	hard	-0.643869086

190	10	1	other	2.5	mascular once a weak	high	low	hard	0.00383736
190	10	0	iran	5.5	mascular once a weak	medium	medium	easy	-0.697626621
190	11	1	other	5.5	subcutances other days	high	medium	easy	-0.34108841
190	11	0	iran	0.5	subcutances three times a weak	medium	low	hard	-0.699689278
190	12	0	iran	5.5	subcutances three times a weak	high	medium	hard	-0.787756632
190	12	1	other	2.5	subcutances other days	medium	low	hard	-0.352164436
191	1	0	iran	5.5	mascular once a weak	high	medium	hard	-0.572088286
191	1	1	iran	0.5	subcutances three times a weak	high	medium	easy	-0.72867963
191	2	1	other	0.5	subcutances other days	high	medium	hard	-0.643869086
191	2	0	iran	5.5	subcutances other days	medium	medium	hard	-0.928090082
191	3	0	iran	5.5	mascular once a weak	high	medium	hard	-0.572088286
191	3	1	iran	2.5	subcutances three times a weak	high	medium	easy	-0.646894171
191	4	0	iran	0.5	subcutances three times a weak	medium	low	hard	-0.699689278
191	4	1	other	2.5	mascular once a weak	high	low	hard	0.00383736
191	5	0	iran	0.5	mascular once a weak	medium	low	easy	-0.303092094
191	5	1	other	2.5	subcutances three times a weak	high	low	easy	-0.030902146
191	6	1	other	2.5	subcutances three times a weak	high	low	easy	-0.030902146
191	6	0	other	0.5	subcutances other days	high	medium	easy	-0.462940247
191	7	0	other	0.5	mascular once a weak	medium	medium	hard	-0.900801637
191	7	1	other	5.5	subcutances other days	medium	low	hard	-0.312098058
191	8	0	iran	2.5	mascular once a weak	high	low	hard	-0.0957683
191	8	1	iran	5.5	subcutances three times a weak	high	low	easy	-0.090441429
191	9	0	iran	2.5	subcutances three times a weak	medium	low	hard	-0.617903819
191	9	1	other	0.5	subcutances other days	high	medium	hard	-0.643869086
191	10	1	other	2.5	mascular once a weak	high	low	hard	0.00383736
191	10	0	iran	5.5	mascular once a weak	medium	medium	easy	-0.697626621
191	11	1	other	5.5	subcutances other days	high	medium	easy	-0.34108841
191	11	0	iran	0.5	subcutances three times a weak	medium	low	hard	-0.699689278
191	12	0	iran	5.5	subcutances three times a weak	high	medium	hard	-0.787756632
191	12	1	other	2.5	subcutances other days	medium	low	hard	-0.352164436
192	1	1	iran	5.5	mascular once a weak	high	medium	hard	-0.572088286
192	1	0	iran	0.5	subcutances three times a weak	high	medium	easy	-0.72867963
192	2	1	other	0.5	subcutances other days	high	medium	hard	-0.643869086
192	2	0	iran	5.5	subcutances other days	medium	medium	hard	-0.928090082

192	3	0	iran	5.5	mascular once a weak	high	medium	hard	-0.572088286
192	3	1	iran	2.5	subcutances three times a weak	high	medium	easy	-0.646894171
192	4	0	iran	0.5	subcutances three times a weak	medium	low	hard	-0.699689278
192	4	1	other	2.5	mascular once a weak	high	low	hard	0.00383736
192	5	0	iran	0.5	mascular once a weak	medium	low	easy	-0.303092094
192	5	1	other	2.5	subcutances three times a weak	high	low	easy	-0.030902146
192	6	1	other	2.5	subcutances three times a weak	high	low	easy	-0.030902146
192	6	0	other	0.5	subcutances other days	high	medium	easy	-0.462940247
192	7	0	other	0.5	mascular once a weak	medium	medium	hard	-0.900801637
192	7	1	other	5.5	subcutances other days	medium	low	hard	-0.312098058
192	8	1	iran	2.5	mascular once a weak	high	low	hard	-0.0957683
192	8	0	iran	5.5	subcutances three times a weak	high	low	easy	-0.090441429
192	9	0	iran	2.5	subcutances three times a weak	medium	low	hard	-0.617903819
192	9	1	other	0.5	subcutances other days	high	medium	hard	-0.643869086
192	10	1	other	2.5	mascular once a weak	high	low	hard	0.00383736
192	10	0	iran	5.5	mascular once a weak	medium	medium	easy	-0.697626621
192	11	1	other	5.5	subcutances other days	high	medium	easy	-0.34108841
192	11	0	iran	0.5	subcutances three times a weak	medium	low	hard	-0.699689278
192	12	1	iran	5.5	subcutances three times a weak	high	medium	hard	-0.787756632
192	12	0	other	2.5	subcutances other days	medium	low	hard	-0.352164436
193	1	0	iran	5.5	mascular once a weak	high	medium	hard	-0.572088286
193	1	0	iran	0.5	subcutances three times a weak	high	medium	easy	-0.72867963
193	2	1	other	0.5	subcutances other days	high	medium	hard	-0.643869086
193	2	0	iran	5.5	subcutances other days	medium	medium	hard	-0.928090082
193	3	0	iran	5.5	mascular once a weak	high	medium	hard	-0.572088286
193	3	0	iran	2.5	subcutances three times a weak	high	medium	easy	-0.646894171
193	4	0	iran	0.5	subcutances three times a weak	medium	low	hard	-0.699689278
193	4	1	other	2.5	mascular once a weak	high	low	hard	0.00383736
193	5	0	iran	0.5	mascular once a weak	medium	low	easy	-0.303092094
193	5	1	other	2.5	subcutances three times a weak	high	low	easy	-0.030902146
193	6	1	other	2.5	subcutances three times a weak	high	low	easy	-0.030902146
193	6	0	other	0.5	subcutances other days	high	medium	easy	-0.462940247
193	7	0	other	0.5	mascular once a weak	medium	medium	hard	-0.900801637
193	7	1	other	5.5	subcutances other days	medium	low	hard	-0.312098058

193	8	0	iran	2.5	mascular once a weak	high	low	hard	-0.0957683
193	8	1	iran	5.5	subcutances three times a weak	high	low	easy	-0.090441429
193	9	0	iran	2.5	subcutances three times a weak	medium	low	hard	-0.617903819
193	9	1	other	0.5	subcutances other days	high	medium	hard	-0.643869086
193	10	1	other	2.5	mascular once a weak	high	low	hard	0.00383736
193	10	0	iran	5.5	mascular once a weak	medium	medium	easy	-0.697626621
193	11	1	other	5.5	subcutances other days	high	medium	easy	-0.34108841
193	11	0	iran	0.5	subcutances three times a weak	medium	low	hard	-0.699689278
193	12	0	iran	5.5	subcutances three times a weak	high	medium	hard	-0.787756632
193	12	1	other	2.5	subcutances other days	medium	low	hard	-0.352164436
194	1	1	iran	5.5	mascular once a weak	high	medium	hard	-0.572088286
194	1	0	iran	0.5	subcutances three times a weak	high	medium	easy	-0.72867963
194	2	0	other	0.5	subcutances other days	high	medium	hard	-0.643869086
194	2	1	iran	5.5	subcutances other days	medium	medium	hard	-0.928090082
194	3	0	iran	5.5	mascular once a weak	high	medium	hard	-0.572088286
194	3	1	iran	2.5	subcutances three times a weak	high	medium	easy	-0.646894171
194	4	0	iran	0.5	subcutances three times a weak	medium	low	hard	-0.699689278
194	4	1	other	2.5	mascular once a weak	high	low	hard	0.00383736
194	5	1	iran	0.5	mascular once a weak	medium	low	easy	-0.303092094
194	5	0	other	2.5	subcutances three times a weak	high	low	easy	-0.030902146
194	6	0	other	2.5	subcutances three times a weak	high	low	easy	-0.030902146
194	6	1	other	0.5	subcutances other days	high	medium	easy	-0.462940247
194	7	0	other	0.5	mascular once a weak	medium	medium	hard	-0.900801637
194	7	1	other	5.5	subcutances other days	medium	low	hard	-0.312098058
194	8	1	iran	2.5	mascular once a weak	high	low	hard	-0.0957683
194	8	0	iran	5.5	subcutances three times a weak	high	low	easy	-0.090441429
194	9	1	iran	2.5	subcutances three times a weak	medium	low	hard	-0.617903819
194	9	0	other	0.5	subcutances other days	high	medium	hard	-0.643869086
194	10	0	other	2.5	mascular once a weak	high	low	hard	0.00383736
194	10	1	iran	5.5	mascular once a weak	medium	medium	easy	-0.697626621
194	11	0	other	5.5	subcutances other days	high	medium	easy	-0.34108841
194	11	1	iran	0.5	subcutances three times a weak	medium	low	hard	-0.699689278
194	12	0	iran	5.5	subcutances three times a weak	high	medium	hard	-0.787756632
194	12	1	other	2.5	subcutances other days	medium	low	hard	-0.352164436

195	1	1	iran	5.5	mascular once a weak	high	medium	hard	-0.572088286
195	1	0	iran	0.5	subcutances three times a weak	high	medium	easy	-0.72867963
195	2	0	other	0.5	subcutances other days	high	medium	hard	-0.643869086
195	2	1	iran	5.5	subcutances other days	medium	medium	hard	-0.928090082
195	3	0	iran	5.5	mascular once a weak	high	medium	hard	-0.572088286
195	3	1	iran	2.5	subcutances three times a weak	high	medium	easy	-0.646894171
195	4	1	iran	0.5	subcutances three times a weak	medium	low	hard	-0.699689278
195	4	0	other	2.5	mascular once a weak	high	low	hard	0.00383736
195	5	1	iran	0.5	mascular once a weak	medium	low	easy	-0.303092094
195	5	0	other	2.5	subcutances three times a weak	high	low	easy	-0.030902146
195	6	0	other	2.5	subcutances three times a weak	high	low	easy	-0.030902146
195	6	1	other	0.5	subcutances other days	high	medium	easy	-0.462940247
195	7	0	other	0.5	mascular once a weak	medium	medium	hard	-0.900801637
195	7	0	other	5.5	subcutances other days	medium	low	hard	-0.312098058
195	8	1	iran	2.5	mascular once a weak	high	low	hard	-0.0957683
195	8	0	iran	5.5	subcutances three times a weak	high	low	easy	-0.090441429
195	9	1	iran	2.5	subcutances three times a weak	medium	low	hard	-0.617903819
195	9	0	other	0.5	subcutances other days	high	medium	hard	-0.643869086
195	10	0	other	2.5	mascular once a weak	high	low	hard	0.00383736
195	10	1	iran	5.5	mascular once a weak	medium	medium	easy	-0.697626621
195	11	0	other	5.5	subcutances other days	high	medium	easy	-0.34108841
195	11	1	iran	0.5	subcutances three times a weak	medium	low	hard	-0.699689278
195	12	0	iran	5.5	subcutances three times a weak	high	medium	hard	-0.787756632
195	12	1	other	2.5	subcutances other days	medium	low	hard	-0.352164436
196	1	0	iran	5.5	mascular once a weak	high	medium	hard	-0.572088286
196	1	1	iran	0.5	subcutances three times a weak	high	medium	easy	-0.72867963
196	2	1	other	0.5	subcutances other days	high	medium	hard	-0.643869086
196	2	0	iran	5.5	subcutances other days	medium	medium	hard	-0.928090082
196	3	0	iran	5.5	mascular once a weak	high	medium	hard	-0.572088286
196	3	1	iran	2.5	subcutances three times a weak	high	medium	easy	-0.646894171
196	4	0	iran	0.5	subcutances three times a weak	medium	low	hard	-0.699689278
196	4	1	other	2.5	mascular once a weak	high	low	hard	0.00383736
196	5	0	iran	0.5	mascular once a weak	medium	low	easy	-0.303092094
196	5	1	other	2.5	subcutances three times a weak	high	low	easy	-0.030902146

196	6	0	other	2.5	subcutances three times a weak	high	low	easy	-0.030902146
196	6	1	other	0.5	subcutances other days	high	medium	easy	-0.462940247
196	7	0	other	0.5	mascular once a weak	medium	medium	hard	-0.900801637
196	7	1	other	5.5	subcutances other days	medium	low	hard	-0.312098058
196	8	1	iran	2.5	mascular once a weak	high	low	hard	-0.0957683
196	8	0	iran	5.5	subcutances three times a weak	high	low	easy	-0.090441429
196	9	0	iran	2.5	subcutances three times a weak	medium	low	hard	-0.617903819
196	9	1	other	0.5	subcutances other days	high	medium	hard	-0.643869086
196	10	0	other	2.5	mascular once a weak	high	low	hard	0.00383736
196	10	1	iran	5.5	mascular once a weak	medium	medium	easy	-0.697626621
196	11	0	other	5.5	subcutances other days	high	medium	easy	-0.34108841
196	11	1	iran	0.5	subcutances three times a weak	medium	low	hard	-0.699689278
196	12	0	iran	5.5	subcutances three times a weak	high	medium	hard	-0.787756632
196	12	1	other	2.5	subcutances other days	medium	low	hard	-0.352164436
197	1	0	iran	5.5	mascular once a weak	high	medium	hard	-0.572088286
197	1	1	iran	0.5	subcutances three times a weak	high	medium	easy	-0.72867963
197	2	1	other	0.5	subcutances other days	high	medium	hard	-0.643869086
197	2	0	iran	5.5	subcutances other days	medium	medium	hard	-0.928090082
197	3	1	iran	5.5	mascular once a weak	high	medium	hard	-0.572088286
197	3	0	iran	2.5	subcutances three times a weak	high	medium	easy	-0.646894171
197	4	0	iran	0.5	subcutances three times a weak	medium	low	hard	-0.699689278
197	4	1	other	2.5	mascular once a weak	high	low	hard	0.00383736
197	5	0	iran	0.5	mascular once a weak	medium	low	easy	-0.303092094
197	5	1	other	2.5	subcutances three times a weak	high	low	easy	-0.030902146
197	6	1	other	2.5	subcutances three times a weak	high	low	easy	-0.030902146
197	6	0	other	0.5	subcutances other days	high	medium	easy	-0.462940247
197	7	0	other	0.5	mascular once a weak	medium	medium	hard	-0.900801637
197	7	1	other	5.5	subcutances other days	medium	low	hard	-0.312098058
197	8	0	iran	2.5	mascular once a weak	high	low	hard	-0.0957683
197	8	1	iran	5.5	subcutances three times a weak	high	low	easy	-0.090441429
197	9	0	iran	2.5	subcutances three times a weak	medium	low	hard	-0.617903819
197	9	1	other	0.5	subcutances other days	high	medium	hard	-0.643869086
197	10	1	other	2.5	mascular once a weak	high	low	hard	0.00383736
197	10	0	iran	5.5	mascular once a weak	medium	medium	easy	-0.697626621

197	11	1	other	5.5	subcutances other days	high	medium	easy	-0.34108841
197	11	0	iran	0.5	subcutances three times a weak	medium	low	hard	-0.699689278
197	12	0	iran	5.5	subcutances three times a weak	high	medium	hard	-0.787756632
197	12	1	other	2.5	subcutances other days	medium	low	hard	-0.352164436
198	1	0	iran	5.5	mascular once a weak	high	medium	hard	-0.572088286
198	1	1	iran	0.5	subcutances three times a weak	high	medium	easy	-0.72867963
198	2	0	other	0.5	subcutances other days	high	medium	hard	-0.643869086
198	2	1	iran	5.5	subcutances other days	medium	medium	hard	-0.928090082
198	3	0	iran	5.5	mascular once a weak	high	medium	hard	-0.572088286
198	3	1	iran	2.5	subcutances three times a weak	high	medium	easy	-0.646894171
198	4	1	iran	0.5	subcutances three times a weak	medium	low	hard	-0.699689278
198	4	0	other	2.5	mascular once a weak	high	low	hard	0.00383736
198	5	0	iran	0.5	mascular once a weak	medium	low	easy	-0.303092094
198	5	1	other	2.5	subcutances three times a weak	high	low	easy	-0.030902146
198	6	0	other	2.5	subcutances three times a weak	high	low	easy	-0.030902146
198	6	1	other	0.5	subcutances other days	high	medium	easy	-0.462940247
198	7	0	other	0.5	mascular once a weak	medium	medium	hard	-0.900801637
198	7	1	other	5.5	subcutances other days	medium	low	hard	-0.312098058
198	8	0	iran	2.5	mascular once a weak	high	low	hard	-0.0957683
198	8	1	iran	5.5	subcutances three times a weak	high	low	easy	-0.090441429
198	9	0	iran	2.5	subcutances three times a weak	medium	low	hard	-0.617903819
198	9	1	other	0.5	subcutances other days	high	medium	hard	-0.643869086
198	10	0	other	2.5	mascular once a weak	high	low	hard	0.00383736
198	10	1	iran	5.5	mascular once a weak	medium	medium	easy	-0.697626621
198	11	0	other	5.5	subcutances other days	high	medium	easy	-0.34108841
198	11	1	iran	0.5	subcutances three times a weak	medium	low	hard	-0.699689278
198	12	0	iran	5.5	subcutances three times a weak	high	medium	hard	-0.787756632
198	12	1	other	2.5	subcutances other days	medium	low	hard	-0.352164436
199	1	1	iran	5.5	mascular once a weak	high	medium	hard	-0.572088286
199	1	0	iran	0.5	subcutances three times a weak	high	medium	easy	-0.72867963
199	2	0	other	0.5	subcutances other days	high	medium	hard	-0.643869086
199	2	1	iran	5.5	subcutances other days	medium	medium	hard	-0.928090082
199	3	0	iran	5.5	mascular once a weak	high	medium	hard	-0.572088286
199	3	1	iran	2.5	subcutances three times a weak	high	medium	easy	-0.646894171

199	4	1	iran	0.5	subcutances	three times a weak	medium	low	hard	-0.699689278
199	4	0	other	2.5	mascular	once a weak	high	low	hard	0.00383736
199	5	1	iran	0.5	mascular	once a weak	medium	low	easy	-0.303092094
199	5	0	other	2.5	subcutances	three times a weak	high	low	easy	-0.030902146
199	6	0	other	2.5	subcutances	three times a weak	high	low	easy	-0.030902146
199	6	1	other	0.5	subcutances	other days	high	medium	easy	-0.462940247
199	7	1	other	0.5	mascular	once a weak	medium	medium	hard	-0.900801637
199	7	0	other	5.5	subcutances	other days	medium	low	hard	-0.312098058
199	8	1	iran	2.5	mascular	once a weak	high	low	hard	-0.0957683
199	8	0	iran	5.5	subcutances	three times a weak	high	low	easy	-0.090441429
199	9	1	iran	2.5	subcutances	three times a weak	medium	low	hard	-0.617903819
199	9	0	other	0.5	subcutances	other days	high	medium	hard	-0.643869086
199	10	0	other	2.5	mascular	once a weak	high	low	hard	0.00383736
199	10	1	iran	5.5	mascular	once a weak	medium	medium	easy	-0.697626621
199	11	0	other	5.5	subcutances	other days	high	medium	easy	-0.34108841
199	11	1	iran	0.5	subcutances	three times a weak	medium	low	hard	-0.699689278
199	12	0	iran	5.5	subcutances	three times a weak	high	medium	hard	-0.787756632
199	12	1	other	2.5	subcutances	other days	medium	low	hard	-0.352164436
200	1	1	iran	5.5	mascular	once a weak	high	medium	hard	-0.572088286
200	1	0	iran	0.5	subcutances	three times a weak	high	medium	easy	-0.72867963
200	2	1	other	0.5	subcutances	other days	high	medium	hard	-0.643869086
200	2	0	iran	5.5	subcutances	other days	medium	medium	hard	-0.928090082
200	3	0	iran	5.5	mascular	once a weak	high	medium	hard	-0.572088286
200	3	1	iran	2.5	subcutances	three times a weak	high	medium	easy	-0.646894171
200	4	0	iran	0.5	subcutances	three times a weak	medium	low	hard	-0.699689278
200	4	1	other	2.5	mascular	once a weak	high	low	hard	0.00383736
200	5	0	iran	0.5	mascular	once a weak	medium	low	easy	-0.303092094
200	5	1	other	2.5	subcutances	three times a weak	high	low	easy	-0.030902146
200	6	0	other	2.5	subcutances	three times a weak	high	low	easy	-0.030902146
200	6	1	other	0.5	subcutances	other days	high	medium	easy	-0.462940247
200	7	1	other	0.5	mascular	once a weak	medium	medium	hard	-0.900801637
200	7	0	other	5.5	subcutances	other days	medium	low	hard	-0.312098058
200	8	1	iran	2.5	mascular	once a weak	high	low	hard	-0.0957683
200	8	0	iran	5.5	subcutances	three times a weak	high	low	easy	-0.090441429

200	9	0	iran	2.5	subcutances three times a weak	medium	low	hard	-0.617903819
200	9	1	other	0.5	subcutances other days	high	medium	hard	-0.643869086
200	10	0	other	2.5	mascular once a weak	high	low	hard	0.00383736
200	10	1	iran	5.5	mascular once a weak	medium	medium	easy	-0.697626621
200	11	0	other	5.5	subcutances other days	high	medium	easy	-0.34108841
200	11	1	iran	0.5	subcutances three times a weak	medium	low	hard	-0.699689278
200	12	0	iran	5.5	subcutances three times a weak	high	medium	hard	-0.787756632
200	12	1	other	2.5	subcutances other days	medium	low	hard	-0.352164436
201	1	0	iran	5.5	mascular once a weak	high	medium	hard	-0.572088286
201	1	1	iran	0.5	subcutances three times a weak	high	medium	easy	-0.72867963
201	2	0	other	0.5	subcutances other days	high	medium	hard	-0.643869086
201	2	0	iran	5.5	subcutances other days	medium	medium	hard	-0.928090082
201	3	0	iran	5.5	mascular once a weak	high	medium	hard	-0.572088286
201	3	1	iran	2.5	subcutances three times a weak	high	medium	easy	-0.646894171
201	4	0	iran	0.5	subcutances three times a weak	medium	low	hard	-0.699689278
201	4	1	other	2.5	mascular once a weak	high	low	hard	0.00383736
201	5	0	iran	0.5	mascular once a weak	medium	low	easy	-0.303092094
201	5	1	other	2.5	subcutances three times a weak	high	low	easy	-0.030902146
201	6	0	other	2.5	subcutances three times a weak	high	low	easy	-0.030902146
201	6	1	other	0.5	subcutances other days	high	medium	easy	-0.462940247
201	7	0	other	0.5	mascular once a weak	medium	medium	hard	-0.900801637
201	7	1	other	5.5	subcutances other days	medium	low	hard	-0.312098058
201	8	1	iran	2.5	mascular once a weak	high	low	hard	-0.0957683
201	8	0	iran	5.5	subcutances three times a weak	high	low	easy	-0.090441429
201	9	1	iran	2.5	subcutances three times a weak	medium	low	hard	-0.617903819
201	9	0	other	0.5	subcutances other days	high	medium	hard	-0.643869086
201	10	0	other	2.5	mascular once a weak	high	low	hard	0.00383736
201	10	1	iran	5.5	mascular once a weak	medium	medium	easy	-0.697626621
201	11	1	other	5.5	subcutances other days	high	medium	easy	-0.34108841
201	11	0	iran	0.5	subcutances three times a weak	medium	low	hard	-0.699689278
201	12	0	iran	5.5	subcutances three times a weak	high	medium	hard	-0.787756632
201	12	1	other	2.5	subcutances other days	medium	low	hard	-0.352164436
202	1	1	iran	5.5	mascular once a weak	high	medium	hard	-0.572088286
202	1	0	iran	0.5	subcutances three times a weak	high	medium	easy	-0.72867963

202	2	0	other	0.5	subcutances other days	high	medium	hard	-0.643869086
202	2	1	iran	5.5	subcutances other days	medium	medium	hard	-0.928090082
202	3	0	iran	5.5	mascular once a weak	high	medium	hard	-0.572088286
202	3	1	iran	2.5	subcutances three times a weak	high	medium	easy	-0.646894171
202	4	1	iran	0.5	subcutances three times a weak	medium	low	hard	-0.699689278
202	4	0	other	2.5	mascular once a weak	high	low	hard	0.00383736
202	5	1	iran	0.5	mascular once a weak	medium	low	easy	-0.303092094
202	5	0	other	2.5	subcutances three times a weak	high	low	easy	-0.030902146
202	6	0	other	2.5	subcutances three times a weak	high	low	easy	-0.030902146
202	6	1	other	0.5	subcutances other days	high	medium	easy	-0.462940247
202	7	0	other	0.5	mascular once a weak	medium	medium	hard	-0.900801637
202	7	1	other	5.5	subcutances other days	medium	low	hard	-0.312098058
202	8	1	iran	2.5	mascular once a weak	high	low	hard	-0.0957683
202	8	0	iran	5.5	subcutances three times a weak	high	low	easy	-0.090441429
202	9	1	iran	2.5	subcutances three times a weak	medium	low	hard	-0.617903819
202	9	0	other	0.5	subcutances other days	high	medium	hard	-0.643869086
202	10	0	other	2.5	mascular once a weak	high	low	hard	0.00383736
202	10	1	iran	5.5	mascular once a weak	medium	medium	easy	-0.697626621
202	11	0	other	5.5	subcutances other days	high	medium	easy	-0.34108841
202	11	1	iran	0.5	subcutances three times a weak	medium	low	hard	-0.699689278
202	12	0	iran	5.5	subcutances three times a weak	high	medium	hard	-0.787756632
202	12	1	other	2.5	subcutances other days	medium	low	hard	-0.352164436
203	1	1	iran	5.5	mascular once a weak	high	medium	hard	-0.572088286
203	1	0	iran	0.5	subcutances three times a weak	high	medium	easy	-0.72867963
203	2	1	other	0.5	subcutances other days	high	medium	hard	-0.643869086
203	2	0	iran	5.5	subcutances other days	medium	medium	hard	-0.928090082
203	3	1	iran	5.5	mascular once a weak	high	medium	hard	-0.572088286
203	3	0	iran	2.5	subcutances three times a weak	high	medium	easy	-0.646894171
203	4	0	iran	0.5	subcutances three times a weak	medium	low	hard	-0.699689278
203	4	1	other	2.5	mascular once a weak	high	low	hard	0.00383736
203	5	0	iran	0.5	mascular once a weak	medium	low	easy	-0.303092094
203	5	1	other	2.5	subcutances three times a weak	high	low	easy	-0.030902146
203	6	1	other	2.5	subcutances three times a weak	high	low	easy	-0.030902146
203	6	0	other	0.5	subcutances other days	high	medium	easy	-0.462940247

203	7	0	other	0.5	mascular once a weak	medium	medium	hard	-0.900801637
203	7	1	other	5.5	subcutances other days	medium	low	hard	-0.312098058
203	8	1	iran	2.5	mascular once a weak	high	low	hard	-0.0957683
203	8	0	iran	5.5	subcutances three times a weak	high	low	easy	-0.090441429
203	9	0	iran	2.5	subcutances three times a weak	medium	low	hard	-0.617903819
203	9	1	other	0.5	subcutances other days	high	medium	hard	-0.643869086
203	10	0	other	2.5	mascular once a weak	high	low	hard	0.00383736
203	10	1	iran	5.5	mascular once a weak	medium	medium	easy	-0.697626621
203	11	1	other	5.5	subcutances other days	high	medium	easy	-0.34108841
203	11	0	iran	0.5	subcutances three times a weak	medium	low	hard	-0.699689278
203	12	1	iran	5.5	subcutances three times a weak	high	medium	hard	-0.787756632
203	12	0	other	2.5	subcutances other days	medium	low	hard	-0.352164436
204	1	1	iran	5.5	mascular once a weak	high	medium	hard	-0.572088286
204	1	0	iran	0.5	subcutances three times a weak	high	medium	easy	-0.72867963
204	2	0	other	0.5	subcutances other days	high	medium	hard	-0.643869086
204	2	1	iran	5.5	subcutances other days	medium	medium	hard	-0.928090082
204	3	1	iran	5.5	mascular once a weak	high	medium	hard	-0.572088286
204	3	0	iran	2.5	subcutances three times a weak	high	medium	easy	-0.646894171
204	4	1	iran	0.5	subcutances three times a weak	medium	low	hard	-0.699689278
204	4	0	other	2.5	mascular once a weak	high	low	hard	0.00383736
204	5	1	iran	0.5	mascular once a weak	medium	low	easy	-0.303092094
204	5	0	other	2.5	subcutances three times a weak	high	low	easy	-0.030902146
204	6	0	other	2.5	subcutances three times a weak	high	low	easy	-0.030902146
204	6	1	other	0.5	subcutances other days	high	medium	easy	-0.462940247
204	7	1	other	0.5	mascular once a weak	medium	medium	hard	-0.900801637
204	7	0	other	5.5	subcutances other days	medium	low	hard	-0.312098058
204	8	0	iran	2.5	mascular once a weak	high	low	hard	-0.0957683
204	8	1	iran	5.5	subcutances three times a weak	high	low	easy	-0.090441429
204	9	1	iran	2.5	subcutances three times a weak	medium	low	hard	-0.617903819
204	9	0	other	0.5	subcutances other days	high	medium	hard	-0.643869086
204	10	0	other	2.5	mascular once a weak	high	low	hard	0.00383736
204	10	1	iran	5.5	mascular once a weak	medium	medium	easy	-0.697626621
204	11	0	other	5.5	subcutances other days	high	medium	easy	-0.34108841
204	11	1	iran	0.5	subcutances three times a weak	medium	low	hard	-0.699689278

204	12	0	iran	5.5	subcutances three times a weak	high	medium	hard	-0.787756632
204	12	1	other	2.5	subcutances other days	medium	low	hard	-0.352164436
205	1	0	iran	5.5	mascular once a weak	high	medium	hard	-0.572088286
205	1	1	iran	0.5	subcutances three times a weak	high	medium	easy	-0.72867963
205	2	1	other	0.5	subcutances other days	high	medium	hard	-0.643869086
205	2	0	iran	5.5	subcutances other days	medium	medium	hard	-0.928090082
205	3	1	iran	5.5	mascular once a weak	high	medium	hard	-0.572088286
205	3	0	iran	2.5	subcutances three times a weak	high	medium	easy	-0.646894171
205	4	0	iran	0.5	subcutances three times a weak	medium	low	hard	-0.699689278
205	4	1	other	2.5	mascular once a weak	high	low	hard	0.00383736
205	5	0	iran	0.5	mascular once a weak	medium	low	easy	-0.303092094
205	5	1	other	2.5	subcutances three times a weak	high	low	easy	-0.030902146
205	6	1	other	2.5	subcutances three times a weak	high	low	easy	-0.030902146
205	6	0	other	0.5	subcutances other days	high	medium	easy	-0.462940247
205	7	0	other	0.5	mascular once a weak	medium	medium	hard	-0.900801637
205	7	1	other	5.5	subcutances other days	medium	low	hard	-0.312098058
205	8	1	iran	2.5	mascular once a weak	high	low	hard	-0.0957683
205	8	0	iran	5.5	subcutances three times a weak	high	low	easy	-0.090441429
205	9	1	iran	2.5	subcutances three times a weak	medium	low	hard	-0.617903819
205	9	0	other	0.5	subcutances other days	high	medium	hard	-0.643869086
205	10	1	other	2.5	mascular once a weak	high	low	hard	0.00383736
205	10	0	iran	5.5	mascular once a weak	medium	medium	easy	-0.697626621
205	11	1	other	5.5	subcutances other days	high	medium	easy	-0.34108841
205	11	0	iran	0.5	subcutances three times a weak	medium	low	hard	-0.699689278
205	12	1	iran	5.5	subcutances three times a weak	high	medium	hard	-0.787756632
205	12	0	other	2.5	subcutances other days	medium	low	hard	-0.352164436
206	1	0	iran	5.5	mascular once a weak	high	medium	hard	-0.572088286
206	1	0	iran	0.5	subcutances three times a weak	high	medium	easy	-0.72867963
206	2	1	other	0.5	subcutances other days	high	medium	hard	-0.643869086
206	2	0	iran	5.5	subcutances other days	medium	medium	hard	-0.928090082
206	3	0	iran	5.5	mascular once a weak	high	medium	hard	-0.572088286
206	3	0	iran	2.5	subcutances three times a weak	high	medium	easy	-0.646894171
206	4	0	iran	0.5	subcutances three times a weak	medium	low	hard	-0.699689278
206	4	1	other	2.5	mascular once a weak	high	low	hard	0.00383736

206	5	0	iran	0.5	mascular once a weak	medium	low	easy	-0.303092094
206	5	1	other	2.5	subcutances three times a weak	high	low	easy	-0.030902146
206	6	0	other	2.5	subcutances three times a weak	high	low	easy	-0.030902146
206	6	1	other	0.5	subcutances other days	high	medium	easy	-0.462940247
206	7	1	other	0.5	mascular once a weak	medium	medium	hard	-0.900801637
206	7	0	other	5.5	subcutances other days	medium	low	hard	-0.312098058
206	8	0	iran	2.5	mascular once a weak	high	low	hard	-0.0957683
206	8	0	iran	5.5	subcutances three times a weak	high	low	easy	-0.090441429
206	9	0	iran	2.5	subcutances three times a weak	medium	low	hard	-0.617903819
206	9	1	other	0.5	subcutances other days	high	medium	hard	-0.643869086
206	10	1	other	2.5	mascular once a weak	high	low	hard	0.00383736
206	10	0	iran	5.5	mascular once a weak	medium	medium	easy	-0.697626621
206	11	1	other	5.5	subcutances other days	high	medium	easy	-0.34108841
206	11	0	iran	0.5	subcutances three times a weak	medium	low	hard	-0.699689278
206	12	0	iran	5.5	subcutances three times a weak	high	medium	hard	-0.787756632
206	12	1	other	2.5	subcutances other days	medium	low	hard	-0.352164436
207	1	0	iran	5.5	mascular once a weak	high	medium	hard	-0.572088286
207	1	0	iran	0.5	subcutances three times a weak	high	medium	easy	-0.72867963
207	2	1	other	0.5	subcutances other days	high	medium	hard	-0.643869086
207	2	0	iran	5.5	subcutances other days	medium	medium	hard	-0.928090082
207	3	0	iran	5.5	mascular once a weak	high	medium	hard	-0.572088286
207	3	0	iran	2.5	subcutances three times a weak	high	medium	easy	-0.646894171
207	4	0	iran	0.5	subcutances three times a weak	medium	low	hard	-0.699689278
207	4	1	other	2.5	mascular once a weak	high	low	hard	0.00383736
207	5	0	iran	0.5	mascular once a weak	medium	low	easy	-0.303092094
207	5	1	other	2.5	subcutances three times a weak	high	low	easy	-0.030902146
207	6	1	other	2.5	subcutances three times a weak	high	low	easy	-0.030902146
207	6	0	other	0.5	subcutances other days	high	medium	easy	-0.462940247
207	7	0	other	0.5	mascular once a weak	medium	medium	hard	-0.900801637
207	7	1	other	5.5	subcutances other days	medium	low	hard	-0.312098058
207	8	0	iran	2.5	mascular once a weak	high	low	hard	-0.0957683
207	8	0	iran	5.5	subcutances three times a weak	high	low	easy	-0.090441429
207	9	0	iran	2.5	subcutances three times a weak	medium	low	hard	-0.617903819
207	9	1	other	0.5	subcutances other days	high	medium	hard	-0.643869086

207	10	1	other	2.5	mascular once a weak	high	low	hard	0.00383736
207	10	0	iran	5.5	mascular once a weak	medium	medium	easy	-0.697626621
207	11	1	other	5.5	subcutances other days	high	medium	easy	-0.34108841
207	11	0	iran	0.5	subcutances three times a weak	medium	low	hard	-0.699689278
207	12	1	iran	5.5	subcutances three times a weak	high	medium	hard	-0.787756632
207	12	0	other	2.5	subcutances other days	medium	low	hard	-0.352164436
208	1	1	iran	5.5	mascular once a weak	high	medium	hard	-0.572088286
208	1	0	iran	0.5	subcutances three times a weak	high	medium	easy	-0.72867963
208	2	1	other	0.5	subcutances other days	high	medium	hard	-0.643869086
208	2	0	iran	5.5	subcutances other days	medium	medium	hard	-0.928090082
208	3	0	iran	5.5	mascular once a weak	high	medium	hard	-0.572088286
208	3	1	iran	2.5	subcutances three times a weak	high	medium	easy	-0.646894171
208	4	0	iran	0.5	subcutances three times a weak	medium	low	hard	-0.699689278
208	4	1	other	2.5	mascular once a weak	high	low	hard	0.00383736
208	5	0	iran	0.5	mascular once a weak	medium	low	easy	-0.303092094
208	5	1	other	2.5	subcutances three times a weak	high	low	easy	-0.030902146
208	6	1	other	2.5	subcutances three times a weak	high	low	easy	-0.030902146
208	6	0	other	0.5	subcutances other days	high	medium	easy	-0.462940247
208	7	0	other	0.5	mascular once a weak	medium	medium	hard	-0.900801637
208	7	1	other	5.5	subcutances other days	medium	low	hard	-0.312098058
208	8	1	iran	2.5	mascular once a weak	high	low	hard	-0.0957683
208	8	0	iran	5.5	subcutances three times a weak	high	low	easy	-0.090441429
208	9	1	iran	2.5	subcutances three times a weak	medium	low	hard	-0.617903819
208	9	0	other	0.5	subcutances other days	high	medium	hard	-0.643869086
208	10	1	other	2.5	mascular once a weak	high	low	hard	0.00383736
208	10	0	iran	5.5	mascular once a weak	medium	medium	easy	-0.697626621
208	11	1	other	5.5	subcutances other days	high	medium	easy	-0.34108841
208	11	0	iran	0.5	subcutances three times a weak	medium	low	hard	-0.699689278
208	12	1	iran	5.5	subcutances three times a weak	high	medium	hard	-0.787756632
208	12	0	other	2.5	subcutances other days	medium	low	hard	-0.352164436
209	1	1	iran	5.5	mascular once a weak	high	medium	hard	-0.572088286
209	1	0	iran	0.5	subcutances three times a weak	high	medium	easy	-0.72867963
209	2	1	other	0.5	subcutances other days	high	medium	hard	-0.643869086
209	2	0	iran	5.5	subcutances other days	medium	medium	hard	-0.928090082

209	3	1	iran	5.5	mascular once a weak	high	medium	hard	-0.572088286
209	3	0	iran	2.5	subcutances three times a weak	high	medium	easy	-0.646894171
209	4	0	iran	0.5	subcutances three times a weak	medium	low	hard	-0.699689278
209	4	1	other	2.5	mascular once a weak	high	low	hard	0.00383736
209	5	1	iran	0.5	mascular once a weak	medium	low	easy	-0.303092094
209	5	0	other	2.5	subcutances three times a weak	high	low	easy	-0.030902146
209	6	1	other	2.5	subcutances three times a weak	high	low	easy	-0.030902146
209	6	0	other	0.5	subcutances other days	high	medium	easy	-0.462940247
209	7	1	other	0.5	mascular once a weak	medium	medium	hard	-0.900801637
209	7	0	other	5.5	subcutances other days	medium	low	hard	-0.312098058
209	8	1	iran	2.5	mascular once a weak	high	low	hard	-0.0957683
209	8	0	iran	5.5	subcutances three times a weak	high	low	easy	-0.090441429
209	9	1	iran	2.5	subcutances three times a weak	medium	low	hard	-0.617903819
209	9	0	other	0.5	subcutances other days	high	medium	hard	-0.643869086
209	10	0	other	2.5	mascular once a weak	high	low	hard	0.00383736
209	10	1	iran	5.5	mascular once a weak	medium	medium	easy	-0.697626621
209	11	0	other	5.5	subcutances other days	high	medium	easy	-0.34108841
209	11	1	iran	0.5	subcutances three times a weak	medium	low	hard	-0.699689278
209	12	1	iran	5.5	subcutances three times a weak	high	medium	hard	-0.787756632
209	12	0	other	2.5	subcutances other days	medium	low	hard	-0.352164436
210	1	0	iran	5.5	mascular once a weak	high	medium	hard	-0.572088286
210	1	1	iran	0.5	subcutances three times a weak	high	medium	easy	-0.72867963
210	2	1	other	0.5	subcutances other days	high	medium	hard	-0.643869086
210	2	0	iran	5.5	subcutances other days	medium	medium	hard	-0.928090082
210	3	0	iran	5.5	mascular once a weak	high	medium	hard	-0.572088286
210	3	1	iran	2.5	subcutances three times a weak	high	medium	easy	-0.646894171
210	4	0	iran	0.5	subcutances three times a weak	medium	low	hard	-0.699689278
210	4	1	other	2.5	mascular once a weak	high	low	hard	0.00383736
210	5	0	iran	0.5	mascular once a weak	medium	low	easy	-0.303092094
210	5	1	other	2.5	subcutances three times a weak	high	low	easy	-0.030902146
210	6	1	other	2.5	subcutances three times a weak	high	low	easy	-0.030902146
210	6	0	other	0.5	subcutances other days	high	medium	easy	-0.462940247
210	7	0	other	0.5	mascular once a weak	medium	medium	hard	-0.900801637
210	7	1	other	5.5	subcutances other days	medium	low	hard	-0.312098058

210	8	0	iran	2.5	mascular once a weak	high	low	hard	-0.0957683
210	8	1	iran	5.5	subcutances three times a weak	high	low	easy	-0.090441429
210	9	0	iran	2.5	subcutances three times a weak	medium	low	hard	-0.617903819
210	9	1	other	0.5	subcutances other days	high	medium	hard	-0.643869086
210	10	1	other	2.5	mascular once a weak	high	low	hard	0.00383736
210	10	0	iran	5.5	mascular once a weak	medium	medium	easy	-0.697626621
210	11	1	other	5.5	subcutances other days	high	medium	easy	-0.34108841
210	11	0	iran	0.5	subcutances three times a weak	medium	low	hard	-0.699689278
210	12	1	iran	5.5	subcutances three times a weak	high	medium	hard	-0.787756632
210	12	0	other	2.5	subcutances other days	medium	low	hard	-0.352164436
211	1	1	iran	5.5	mascular once a weak	high	medium	hard	-0.572088286
211	1	0	iran	0.5	subcutances three times a weak	high	medium	easy	-0.72867963
211	2	0	other	0.5	subcutances other days	high	medium	hard	-0.643869086
211	2	1	iran	5.5	subcutances other days	medium	medium	hard	-0.928090082
211	3	0	iran	5.5	mascular once a weak	high	medium	hard	-0.572088286
211	3	1	iran	2.5	subcutances three times a weak	high	medium	easy	-0.646894171
211	4	1	iran	0.5	subcutances three times a weak	medium	low	hard	-0.699689278
211	4	0	other	2.5	mascular once a weak	high	low	hard	0.00383736
211	5	1	iran	0.5	mascular once a weak	medium	low	easy	-0.303092094
211	5	0	other	2.5	subcutances three times a weak	high	low	easy	-0.030902146
211	6	0	other	2.5	subcutances three times a weak	high	low	easy	-0.030902146
211	6	1	other	0.5	subcutances other days	high	medium	easy	-0.462940247
211	7	0	other	0.5	mascular once a weak	medium	medium	hard	-0.900801637
211	7	1	other	5.5	subcutances other days	medium	low	hard	-0.312098058
211	8	1	iran	2.5	mascular once a weak	high	low	hard	-0.0957683
211	8	0	iran	5.5	subcutances three times a weak	high	low	easy	-0.090441429
211	9	1	iran	2.5	subcutances three times a weak	medium	low	hard	-0.617903819
211	9	0	other	0.5	subcutances other days	high	medium	hard	-0.643869086
211	10	0	other	2.5	mascular once a weak	high	low	hard	0.00383736
211	10	1	iran	5.5	mascular once a weak	medium	medium	easy	-0.697626621
211	11	0	other	5.5	subcutances other days	high	medium	easy	-0.34108841
211	11	1	iran	0.5	subcutances three times a weak	medium	low	hard	-0.699689278
211	12	0	iran	5.5	subcutances three times a weak	high	medium	hard	-0.787756632
211	12	1	other	2.5	subcutances other days	medium	low	hard	-0.352164436

212	1	1	iran	5.5	mascular once a weak	high	medium	hard	-0.572088286
212	1	0	iran	0.5	subcutances three times a weak	high	medium	easy	-0.72867963
212	2	1	other	0.5	subcutances other days	high	medium	hard	-0.643869086
212	2	0	iran	5.5	subcutances other days	medium	medium	hard	-0.928090082
212	3	1	iran	5.5	mascular once a weak	high	medium	hard	-0.572088286
212	3	0	iran	2.5	subcutances three times a weak	high	medium	easy	-0.646894171
212	4	0	iran	0.5	subcutances three times a weak	medium	low	hard	-0.699689278
212	4	1	other	2.5	mascular once a weak	high	low	hard	0.00383736
212	5	1	iran	0.5	mascular once a weak	medium	low	easy	-0.303092094
212	5	0	other	2.5	subcutances three times a weak	high	low	easy	-0.030902146
212	6	0	other	2.5	subcutances three times a weak	high	low	easy	-0.030902146
212	6	1	other	0.5	subcutances other days	high	medium	easy	-0.462940247
212	7	0	other	0.5	mascular once a weak	medium	medium	hard	-0.900801637
212	7	1	other	5.5	subcutances other days	medium	low	hard	-0.312098058
212	8	0	iran	2.5	mascular once a weak	high	low	hard	-0.0957683
212	8	1	iran	5.5	subcutances three times a weak	high	low	easy	-0.090441429
212	9	1	iran	2.5	subcutances three times a weak	medium	low	hard	-0.617903819
212	9	0	other	0.5	subcutances other days	high	medium	hard	-0.643869086
212	10	0	other	2.5	mascular once a weak	high	low	hard	0.00383736
212	10	1	iran	5.5	mascular once a weak	medium	medium	easy	-0.697626621
212	11	1	other	5.5	subcutances other days	high	medium	easy	-0.34108841
212	11	0	iran	0.5	subcutances three times a weak	medium	low	hard	-0.699689278
212	12	1	iran	5.5	subcutances three times a weak	high	medium	hard	-0.787756632
212	12	0	other	2.5	subcutances other days	medium	low	hard	-0.352164436
213	1	0	iran	5.5	mascular once a weak	high	medium	hard	-0.572088286
213	1	1	iran	0.5	subcutances three times a weak	high	medium	easy	-0.72867963
213	2	1	other	0.5	subcutances other days	high	medium	hard	-0.643869086
213	2	0	iran	5.5	subcutances other days	medium	medium	hard	-0.928090082
213	3	0	iran	5.5	mascular once a weak	high	medium	hard	-0.572088286
213	3	0	iran	2.5	subcutances three times a weak	high	medium	easy	-0.646894171
213	4	0	iran	0.5	subcutances three times a weak	medium	low	hard	-0.699689278
213	4	1	other	2.5	mascular once a weak	high	low	hard	0.00383736
213	5	0	iran	0.5	mascular once a weak	medium	low	easy	-0.303092094
213	5	1	other	2.5	subcutances three times a weak	high	low	easy	-0.030902146

213	6	0	other	2.5	subcutances three times a weak	high	low	easy	-0.030902146
213	6	1	other	0.5	subcutances other days	high	medium	easy	-0.462940247
213	7	1	other	0.5	mascular once a weak	medium	medium	hard	-0.900801637
213	7	0	other	5.5	subcutances other days	medium	low	hard	-0.312098058
213	8	0	iran	2.5	mascular once a weak	high	low	hard	-0.0957683
213	8	0	iran	5.5	subcutances three times a weak	high	low	easy	-0.090441429
213	9	0	iran	2.5	subcutances three times a weak	medium	low	hard	-0.617903819
213	9	1	other	0.5	subcutances other days	high	medium	hard	-0.643869086
213	10	1	other	2.5	mascular once a weak	high	low	hard	0.00383736
213	10	0	iran	5.5	mascular once a weak	medium	medium	easy	-0.697626621
213	11	1	other	5.5	subcutances other days	high	medium	easy	-0.34108841
213	11	0	iran	0.5	subcutances three times a weak	medium	low	hard	-0.699689278
213	12	0	iran	5.5	subcutances three times a weak	high	medium	hard	-0.787756632
213	12	1	other	2.5	subcutances other days	medium	low	hard	-0.352164436
214	1	0	iran	5.5	mascular once a weak	high	medium	hard	-0.572088286
214	1	1	iran	0.5	subcutances three times a weak	high	medium	easy	-0.72867963
214	2	1	other	0.5	subcutances other days	high	medium	hard	-0.643869086
214	2	0	iran	5.5	subcutances other days	medium	medium	hard	-0.928090082
214	3	0	iran	5.5	mascular once a weak	high	medium	hard	-0.572088286
214	3	1	iran	2.5	subcutances three times a weak	high	medium	easy	-0.646894171
214	4	0	iran	0.5	subcutances three times a weak	medium	low	hard	-0.699689278
214	4	1	other	2.5	mascular once a weak	high	low	hard	0.00383736
214	5	0	iran	0.5	mascular once a weak	medium	low	easy	-0.303092094
214	5	1	other	2.5	subcutances three times a weak	high	low	easy	-0.030902146
214	6	1	other	2.5	subcutances three times a weak	high	low	easy	-0.030902146
214	6	0	other	0.5	subcutances other days	high	medium	easy	-0.462940247
214	7	0	other	0.5	mascular once a weak	medium	medium	hard	-0.900801637
214	7	1	other	5.5	subcutances other days	medium	low	hard	-0.312098058
214	8	0	iran	2.5	mascular once a weak	high	low	hard	-0.0957683
214	8	1	iran	5.5	subcutances three times a weak	high	low	easy	-0.090441429
214	9	0	iran	2.5	subcutances three times a weak	medium	low	hard	-0.617903819
214	9	1	other	0.5	subcutances other days	high	medium	hard	-0.643869086
214	10	1	other	2.5	mascular once a weak	high	low	hard	0.00383736
214	10	0	iran	5.5	mascular once a weak	medium	medium	easy	-0.697626621

214	11	1	other	5.5	subcutances other days	high	medium	easy	-0.34108841
214	11	0	iran	0.5	subcutances three times a weak	medium	low	hard	-0.699689278
214	12	1	iran	5.5	subcutances three times a weak	high	medium	hard	-0.787756632
214	12	0	other	2.5	subcutances other days	medium	low	hard	-0.352164436
215	1	1	iran	5.5	mascular once a weak	high	medium	hard	-0.572088286
215	1	0	iran	0.5	subcutances three times a weak	high	medium	easy	-0.72867963
215	2	0	other	0.5	subcutances other days	high	medium	hard	-0.643869086
215	2	1	iran	5.5	subcutances other days	medium	medium	hard	-0.928090082
215	3	0	iran	5.5	mascular once a weak	high	medium	hard	-0.572088286
215	3	1	iran	2.5	subcutances three times a weak	high	medium	easy	-0.646894171
215	4	1	iran	0.5	subcutances three times a weak	medium	low	hard	-0.699689278
215	4	0	other	2.5	mascular once a weak	high	low	hard	0.00383736
215	5	1	iran	0.5	mascular once a weak	medium	low	easy	-0.303092094
215	5	0	other	2.5	subcutances three times a weak	high	low	easy	-0.030902146
215	6	1	other	2.5	subcutances three times a weak	high	low	easy	-0.030902146
215	6	0	other	0.5	subcutances other days	high	medium	easy	-0.462940247
215	7	0	other	0.5	mascular once a weak	medium	medium	hard	-0.900801637
215	7	1	other	5.5	subcutances other days	medium	low	hard	-0.312098058
215	8	0	iran	2.5	mascular once a weak	high	low	hard	-0.0957683
215	8	1	iran	5.5	subcutances three times a weak	high	low	easy	-0.090441429
215	9	0	iran	2.5	subcutances three times a weak	medium	low	hard	-0.617903819
215	9	1	other	0.5	subcutances other days	high	medium	hard	-0.643869086
215	10	1	other	2.5	mascular once a weak	high	low	hard	0.00383736
215	10	0	iran	5.5	mascular once a weak	medium	medium	easy	-0.697626621
215	11	0	other	5.5	subcutances other days	high	medium	easy	-0.34108841
215	11	1	iran	0.5	subcutances three times a weak	medium	low	hard	-0.699689278
215	12	1	iran	5.5	subcutances three times a weak	high	medium	hard	-0.787756632
215	12	0	other	2.5	subcutances other days	medium	low	hard	-0.352164436
216	1	1	iran	5.5	mascular once a weak	high	medium	hard	-0.572088286
216	1	0	iran	0.5	subcutances three times a weak	high	medium	easy	-0.72867963
216	2	0	other	0.5	subcutances other days	high	medium	hard	-0.643869086
216	2	1	iran	5.5	subcutances other days	medium	medium	hard	-0.928090082
216	3	0	iran	5.5	mascular once a weak	high	medium	hard	-0.572088286
216	3	1	iran	2.5	subcutances three times a weak	high	medium	easy	-0.646894171

216	4	1	iran	0.5	subcutances three times a weak	medium	low	hard	-0.699689278
216	4	0	other	2.5	mascular once a weak	high	low	hard	0.00383736
216	5	1	iran	0.5	mascular once a weak	medium	low	easy	-0.303092094
216	5	0	other	2.5	subcutances three times a weak	high	low	easy	-0.030902146
216	6	0	other	2.5	subcutances three times a weak	high	low	easy	-0.030902146
216	6	1	other	0.5	subcutances other days	high	medium	easy	-0.462940247
216	7	0	other	0.5	mascular once a weak	medium	medium	hard	-0.900801637
216	7	0	other	5.5	subcutances other days	medium	low	hard	-0.312098058
216	8	1	iran	2.5	mascular once a weak	high	low	hard	-0.0957683
216	8	0	iran	5.5	subcutances three times a weak	high	low	easy	-0.090441429
216	9	1	iran	2.5	subcutances three times a weak	medium	low	hard	-0.617903819
216	9	0	other	0.5	subcutances other days	high	medium	hard	-0.643869086
216	10	0	other	2.5	mascular once a weak	high	low	hard	0.00383736
216	10	1	iran	5.5	mascular once a weak	medium	medium	easy	-0.697626621
216	11	0	other	5.5	subcutances other days	high	medium	easy	-0.34108841
216	11	1	iran	0.5	subcutances three times a weak	medium	low	hard	-0.699689278
216	12	0	iran	5.5	subcutances three times a weak	high	medium	hard	-0.787756632
216	12	1	other	2.5	subcutances other days	medium	low	hard	-0.352164436
217	1	0	iran	5.5	mascular once a weak	high	medium	hard	-0.572088286
217	1	1	iran	0.5	subcutances three times a weak	high	medium	easy	-0.72867963
217	2	1	other	0.5	subcutances other days	high	medium	hard	-0.643869086
217	2	0	iran	5.5	subcutances other days	medium	medium	hard	-0.928090082
217	3	0	iran	5.5	mascular once a weak	high	medium	hard	-0.572088286
217	3	1	iran	2.5	subcutances three times a weak	high	medium	easy	-0.646894171
217	4	1	iran	0.5	subcutances three times a weak	medium	low	hard	-0.699689278
217	4	0	other	2.5	mascular once a weak	high	low	hard	0.00383736
217	5	1	iran	0.5	mascular once a weak	medium	low	easy	-0.303092094
217	5	0	other	2.5	subcutances three times a weak	high	low	easy	-0.030902146
217	6	1	other	2.5	subcutances three times a weak	high	low	easy	-0.030902146
217	6	0	other	0.5	subcutances other days	high	medium	easy	-0.462940247
217	7	1	other	0.5	mascular once a weak	medium	medium	hard	-0.900801637
217	7	0	other	5.5	subcutances other days	medium	low	hard	-0.312098058
217	8	0	iran	2.5	mascular once a weak	high	low	hard	-0.0957683
217	8	1	iran	5.5	subcutances three times a weak	high	low	easy	-0.090441429

217	9	0	iran	2.5	subcutances three times a weak	medium	low	hard	-0.617903819
217	9	1	other	0.5	subcutances other days	high	medium	hard	-0.643869086
217	10	1	other	2.5	mascular once a weak	high	low	hard	0.00383736
217	10	0	iran	5.5	mascular once a weak	medium	medium	easy	-0.697626621
217	11	1	other	5.5	subcutances other days	high	medium	easy	-0.34108841
217	11	0	iran	0.5	subcutances three times a weak	medium	low	hard	-0.699689278
217	12	0	iran	5.5	subcutances three times a weak	high	medium	hard	-0.787756632
217	12	1	other	2.5	subcutances other days	medium	low	hard	-0.352164436
218	1	0	iran	5.5	mascular once a weak	high	medium	hard	-0.572088286
218	1	1	iran	0.5	subcutances three times a weak	high	medium	easy	-0.72867963
218	2	1	other	0.5	subcutances other days	high	medium	hard	-0.643869086
218	2	0	iran	5.5	subcutances other days	medium	medium	hard	-0.928090082
218	3	0	iran	5.5	mascular once a weak	high	medium	hard	-0.572088286
218	3	1	iran	2.5	subcutances three times a weak	high	medium	easy	-0.646894171
218	4	0	iran	0.5	subcutances three times a weak	medium	low	hard	-0.699689278
218	4	1	other	2.5	mascular once a weak	high	low	hard	0.00383736
218	5	0	iran	0.5	mascular once a weak	medium	low	easy	-0.303092094
218	5	1	other	2.5	subcutances three times a weak	high	low	easy	-0.030902146
218	6	1	other	2.5	subcutances three times a weak	high	low	easy	-0.030902146
218	6	0	other	0.5	subcutances other days	high	medium	easy	-0.462940247
218	7	0	other	0.5	mascular once a weak	medium	medium	hard	-0.900801637
218	7	1	other	5.5	subcutances other days	medium	low	hard	-0.312098058
218	8	0	iran	2.5	mascular once a weak	high	low	hard	-0.0957683
218	8	1	iran	5.5	subcutances three times a weak	high	low	easy	-0.090441429
218	9	1	iran	2.5	subcutances three times a weak	medium	low	hard	-0.617903819
218	9	0	other	0.5	subcutances other days	high	medium	hard	-0.643869086
218	10	1	other	2.5	mascular once a weak	high	low	hard	0.00383736
218	10	0	iran	5.5	mascular once a weak	medium	medium	easy	-0.697626621
218	11	1	other	5.5	subcutances other days	high	medium	easy	-0.34108841
218	11	0	iran	0.5	subcutances three times a weak	medium	low	hard	-0.699689278
218	12	1	iran	5.5	subcutances three times a weak	high	medium	hard	-0.787756632
218	12	0	other	2.5	subcutances other days	medium	low	hard	-0.352164436
219	1	0	iran	5.5	mascular once a weak	high	medium	hard	-0.572088286
219	1	1	iran	0.5	subcutances three times a weak	high	medium	easy	-0.72867963

219	2	1	other	0.5	subcutances other days	high	medium	hard	-0.643869086
219	2	0	iran	5.5	subcutances other days	medium	medium	hard	-0.928090082
219	3	0	iran	5.5	mascular once a weak	high	medium	hard	-0.572088286
219	3	1	iran	2.5	subcutances three times a weak	high	medium	easy	-0.646894171
219	4	0	iran	0.5	subcutances three times a weak	medium	low	hard	-0.699689278
219	4	1	other	2.5	mascular once a weak	high	low	hard	0.00383736
219	5	1	iran	0.5	mascular once a weak	medium	low	easy	-0.303092094
219	5	0	other	2.5	subcutances three times a weak	high	low	easy	-0.030902146
219	6	1	other	2.5	subcutances three times a weak	high	low	easy	-0.030902146
219	6	0	other	0.5	subcutances other days	high	medium	easy	-0.462940247
219	7	1	other	0.5	mascular once a weak	medium	medium	hard	-0.900801637
219	7	0	other	5.5	subcutances other days	medium	low	hard	-0.312098058
219	8	0	iran	2.5	mascular once a weak	high	low	hard	-0.0957683
219	8	1	iran	5.5	subcutances three times a weak	high	low	easy	-0.090441429
219	9	0	iran	2.5	subcutances three times a weak	medium	low	hard	-0.617903819
219	9	1	other	0.5	subcutances other days	high	medium	hard	-0.643869086
219	10	1	other	2.5	mascular once a weak	high	low	hard	0.00383736
219	10	0	iran	5.5	mascular once a weak	medium	medium	easy	-0.697626621
219	11	1	other	5.5	subcutances other days	high	medium	easy	-0.34108841
219	11	0	iran	0.5	subcutances three times a weak	medium	low	hard	-0.699689278
219	12	1	iran	5.5	subcutances three times a weak	high	medium	hard	-0.787756632
219	12	0	other	2.5	subcutances other days	medium	low	hard	-0.352164436
220	1	1	iran	5.5	mascular once a weak	high	medium	hard	-0.572088286
220	1	0	iran	0.5	subcutances three times a weak	high	medium	easy	-0.72867963
220	2	1	other	0.5	subcutances other days	high	medium	hard	-0.643869086
220	2	0	iran	5.5	subcutances other days	medium	medium	hard	-0.928090082
220	3	1	iran	5.5	mascular once a weak	high	medium	hard	-0.572088286
220	3	0	iran	2.5	subcutances three times a weak	high	medium	easy	-0.646894171
220	4	1	iran	0.5	subcutances three times a weak	medium	low	hard	-0.699689278
220	4	0	other	2.5	mascular once a weak	high	low	hard	0.00383736
220	5	1	iran	0.5	mascular once a weak	medium	low	easy	-0.303092094
220	5	0	other	2.5	subcutances three times a weak	high	low	easy	-0.030902146
220	6	1	other	2.5	subcutances three times a weak	high	low	easy	-0.030902146
220	6	0	other	0.5	subcutances other days	high	medium	easy	-0.462940247

220	7	1	other	0.5	mascular once a weak	medium	medium	hard	-0.900801637
220	7	0	other	5.5	subcutances other days	medium	low	hard	-0.312098058
220	8	0	iran	2.5	mascular once a weak	high	low	hard	-0.0957683
220	8	1	iran	5.5	subcutances three times a weak	high	low	easy	-0.090441429
220	9	1	iran	2.5	subcutances three times a weak	medium	low	hard	-0.617903819
220	9	0	other	0.5	subcutances other days	high	medium	hard	-0.643869086
220	10	1	other	2.5	mascular once a weak	high	low	hard	0.00383736
220	10	0	iran	5.5	mascular once a weak	medium	medium	easy	-0.697626621
220	11	1	other	5.5	subcutances other days	high	medium	easy	-0.34108841
220	11	0	iran	0.5	subcutances three times a weak	medium	low	hard	-0.699689278
220	12	1	iran	5.5	subcutances three times a weak	high	medium	hard	-0.787756632
220	12	0	other	2.5	subcutances other days	medium	low	hard	-0.352164436
221	1	0	iran	5.5	mascular once a weak	high	medium	hard	-0.572088286
221	1	1	iran	0.5	subcutances three times a weak	high	medium	easy	-0.72867963
221	2	0	other	0.5	subcutances other days	high	medium	hard	-0.643869086
221	2	1	iran	5.5	subcutances other days	medium	medium	hard	-0.928090082
221	3	0	iran	5.5	mascular once a weak	high	medium	hard	-0.572088286
221	3	1	iran	2.5	subcutances three times a weak	high	medium	easy	-0.646894171
221	4	1	iran	0.5	subcutances three times a weak	medium	low	hard	-0.699689278
221	4	0	other	2.5	mascular once a weak	high	low	hard	0.00383736
221	5	0	iran	0.5	mascular once a weak	medium	low	easy	-0.303092094
221	5	1	other	2.5	subcutances three times a weak	high	low	easy	-0.030902146
221	6	0	other	2.5	subcutances three times a weak	high	low	easy	-0.030902146
221	6	1	other	0.5	subcutances other days	high	medium	easy	-0.462940247
221	7	0	other	0.5	mascular once a weak	medium	medium	hard	-0.900801637
221	7	0	other	5.5	subcutances other days	medium	low	hard	-0.312098058
221	8	1	iran	2.5	mascular once a weak	high	low	hard	-0.0957683
221	8	0	iran	5.5	subcutances three times a weak	high	low	easy	-0.090441429
221	9	0	iran	2.5	subcutances three times a weak	medium	low	hard	-0.617903819
221	9	1	other	0.5	subcutances other days	high	medium	hard	-0.643869086
221	10	0	other	2.5	mascular once a weak	high	low	hard	0.00383736
221	10	1	iran	5.5	mascular once a weak	medium	medium	easy	-0.697626621
221	11	0	other	5.5	subcutances other days	high	medium	easy	-0.34108841
221	11	1	iran	0.5	subcutances three times a weak	medium	low	hard	-0.699689278

221	12	0	iran	5.5	subcutances three times a weak	high	medium	hard	-0.787756632
221	12	1	other	2.5	subcutances other days	medium	low	hard	-0.352164436
222	1	1	iran	5.5	mascular once a weak	high	medium	hard	-0.572088286
222	1	0	iran	0.5	subcutances three times a weak	high	medium	easy	-0.72867963
222	2	0	other	0.5	subcutances other days	high	medium	hard	-0.643869086
222	2	1	iran	5.5	subcutances other days	medium	medium	hard	-0.928090082
222	3	0	iran	5.5	mascular once a weak	high	medium	hard	-0.572088286
222	3	1	iran	2.5	subcutances three times a weak	high	medium	easy	-0.646894171
222	4	0	iran	0.5	subcutances three times a weak	medium	low	hard	-0.699689278
222	4	1	other	2.5	mascular once a weak	high	low	hard	0.00383736
222	5	0	iran	0.5	mascular once a weak	medium	low	easy	-0.303092094
222	5	1	other	2.5	subcutances three times a weak	high	low	easy	-0.030902146
222	6	0	other	2.5	subcutances three times a weak	high	low	easy	-0.030902146
222	6	1	other	0.5	subcutances other days	high	medium	easy	-0.462940247
222	7	0	other	0.5	mascular once a weak	medium	medium	hard	-0.900801637
222	7	1	other	5.5	subcutances other days	medium	low	hard	-0.312098058
222	8	1	iran	2.5	mascular once a weak	high	low	hard	-0.0957683
222	8	0	iran	5.5	subcutances three times a weak	high	low	easy	-0.090441429
222	9	1	iran	2.5	subcutances three times a weak	medium	low	hard	-0.617903819
222	9	0	other	0.5	subcutances other days	high	medium	hard	-0.643869086
222	10	0	other	2.5	mascular once a weak	high	low	hard	0.00383736
222	10	1	iran	5.5	mascular once a weak	medium	medium	easy	-0.697626621
222	11	0	other	5.5	subcutances other days	high	medium	easy	-0.34108841
222	11	1	iran	0.5	subcutances three times a weak	medium	low	hard	-0.699689278
222	12	0	iran	5.5	subcutances three times a weak	high	medium	hard	-0.787756632
222	12	1	other	2.5	subcutances other days	medium	low	hard	-0.352164436
223	1	1	iran	5.5	mascular once a weak	high	medium	hard	-0.572088286
223	1	0	iran	0.5	subcutances three times a weak	high	medium	easy	-0.72867963
223	2	1	other	0.5	subcutances other days	high	medium	hard	-0.643869086
223	2	0	iran	5.5	subcutances other days	medium	medium	hard	-0.928090082
223	3	1	iran	5.5	mascular once a weak	high	medium	hard	-0.572088286
223	3	0	iran	2.5	subcutances three times a weak	high	medium	easy	-0.646894171
223	4	0	iran	0.5	subcutances three times a weak	medium	low	hard	-0.699689278
223	4	1	other	2.5	mascular once a weak	high	low	hard	0.00383736

223	5	0	iran	0.5	mascular once a weak	medium	low	easy	-0.303092094
223	5	1	other	2.5	subcutances three times a weak	high	low	easy	-0.030902146
223	6	1	other	2.5	subcutances three times a weak	high	low	easy	-0.030902146
223	6	0	other	0.5	subcutances other days	high	medium	easy	-0.462940247
223	7	1	other	0.5	mascular once a weak	medium	medium	hard	-0.900801637
223	7	0	other	5.5	subcutances other days	medium	low	hard	-0.312098058
223	8	1	iran	2.5	mascular once a weak	high	low	hard	-0.0957683
223	8	0	iran	5.5	subcutances three times a weak	high	low	easy	-0.090441429
223	9	0	iran	2.5	subcutances three times a weak	medium	low	hard	-0.617903819
223	9	1	other	0.5	subcutances other days	high	medium	hard	-0.643869086
223	10	1	other	2.5	mascular once a weak	high	low	hard	0.00383736
223	10	0	iran	5.5	mascular once a weak	medium	medium	easy	-0.697626621
223	11	1	other	5.5	subcutances other days	high	medium	easy	-0.34108841
223	11	0	iran	0.5	subcutances three times a weak	medium	low	hard	-0.699689278
223	12	1	iran	5.5	subcutances three times a weak	high	medium	hard	-0.787756632
223	12	0	other	2.5	subcutances other days	medium	low	hard	-0.352164436
224	1	1	iran	5.5	mascular once a weak	high	medium	hard	-0.572088286
224	1	0	iran	0.5	subcutances three times a weak	high	medium	easy	-0.72867963
224	2	0	other	0.5	subcutances other days	high	medium	hard	-0.643869086
224	2	1	iran	5.5	subcutances other days	medium	medium	hard	-0.928090082
224	3	0	iran	5.5	mascular once a weak	high	medium	hard	-0.572088286
224	3	1	iran	2.5	subcutances three times a weak	high	medium	easy	-0.646894171
224	4	1	iran	0.5	subcutances three times a weak	medium	low	hard	-0.699689278
224	4	0	other	2.5	mascular once a weak	high	low	hard	0.00383736
224	5	1	iran	0.5	mascular once a weak	medium	low	easy	-0.303092094
224	5	0	other	2.5	subcutances three times a weak	high	low	easy	-0.030902146
224	6	1	other	2.5	subcutances three times a weak	high	low	easy	-0.030902146
224	6	0	other	0.5	subcutances other days	high	medium	easy	-0.462940247
224	7	0	other	0.5	mascular once a weak	medium	medium	hard	-0.900801637
224	7	1	other	5.5	subcutances other days	medium	low	hard	-0.312098058
224	8	1	iran	2.5	mascular once a weak	high	low	hard	-0.0957683
224	8	0	iran	5.5	subcutances three times a weak	high	low	easy	-0.090441429
224	9	1	iran	2.5	subcutances three times a weak	medium	low	hard	-0.617903819
224	9	0	other	0.5	subcutances other days	high	medium	hard	-0.643869086

224	10	0	other	2.5	mascular once a weak	high	low	hard	0.00383736
224	10	1	iran	5.5	mascular once a weak	medium	medium	easy	-0.697626621
224	11	1	other	5.5	subcutances other days	high	medium	easy	-0.34108841
224	11	0	iran	0.5	subcutances three times a weak	medium	low	hard	-0.699689278
224	12	0	iran	5.5	subcutances three times a weak	high	medium	hard	-0.787756632
224	12	1	other	2.5	subcutances other days	medium	low	hard	-0.352164436
225	1	0	iran	5.5	mascular once a weak	high	medium	hard	-0.572088286
225	1	1	iran	0.5	subcutances three times a weak	high	medium	easy	-0.72867963
225	2	1	other	0.5	subcutances other days	high	medium	hard	-0.643869086
225	2	0	iran	5.5	subcutances other days	medium	medium	hard	-0.928090082
225	3	0	iran	5.5	mascular once a weak	high	medium	hard	-0.572088286
225	3	1	iran	2.5	subcutances three times a weak	high	medium	easy	-0.646894171
225	4	0	iran	0.5	subcutances three times a weak	medium	low	hard	-0.699689278
225	4	1	other	2.5	mascular once a weak	high	low	hard	0.00383736
225	5	0	iran	0.5	mascular once a weak	medium	low	easy	-0.303092094
225	5	1	other	2.5	subcutances three times a weak	high	low	easy	-0.030902146
225	6	1	other	2.5	subcutances three times a weak	high	low	easy	-0.030902146
225	6	0	other	0.5	subcutances other days	high	medium	easy	-0.462940247
225	7	0	other	0.5	mascular once a weak	medium	medium	hard	-0.900801637
225	7	1	other	5.5	subcutances other days	medium	low	hard	-0.312098058
225	8	0	iran	2.5	mascular once a weak	high	low	hard	-0.0957683
225	8	1	iran	5.5	subcutances three times a weak	high	low	easy	-0.090441429
225	9	0	iran	2.5	subcutances three times a weak	medium	low	hard	-0.617903819
225	9	1	other	0.5	subcutances other days	high	medium	hard	-0.643869086
225	10	1	other	2.5	mascular once a weak	high	low	hard	0.00383736
225	10	0	iran	5.5	mascular once a weak	medium	medium	easy	-0.697626621
225	11	1	other	5.5	subcutances other days	high	medium	easy	-0.34108841
225	11	0	iran	0.5	subcutances three times a weak	medium	low	hard	-0.699689278
225	12	1	iran	5.5	subcutances three times a weak	high	medium	hard	-0.787756632
225	12	0	other	2.5	subcutances other days	medium	low	hard	-0.352164436
226	1	1	iran	5.5	mascular once a weak	high	medium	hard	-0.572088286
226	1	0	iran	0.5	subcutances three times a weak	high	medium	easy	-0.72867963
226	2	1	other	0.5	subcutances other days	high	medium	hard	-0.643869086
226	2	0	iran	5.5	subcutances other days	medium	medium	hard	-0.928090082

226	3	0	iran	5.5	mascular once a weak	high	medium	hard	-0.572088286
226	3	1	iran	2.5	subcutances three times a weak	high	medium	easy	-0.646894171
226	4	1	iran	0.5	subcutances three times a weak	medium	low	hard	-0.699689278
226	4	0	other	2.5	mascular once a weak	high	low	hard	0.00383736
226	5	1	iran	0.5	mascular once a weak	medium	low	easy	-0.303092094
226	5	0	other	2.5	subcutances three times a weak	high	low	easy	-0.030902146
226	6	0	other	2.5	subcutances three times a weak	high	low	easy	-0.030902146
226	6	1	other	0.5	subcutances other days	high	medium	easy	-0.462940247
226	7	0	other	0.5	mascular once a weak	medium	medium	hard	-0.900801637
226	7	1	other	5.5	subcutances other days	medium	low	hard	-0.312098058
226	8	1	iran	2.5	mascular once a weak	high	low	hard	-0.0957683
226	8	0	iran	5.5	subcutances three times a weak	high	low	easy	-0.090441429
226	9	1	iran	2.5	subcutances three times a weak	medium	low	hard	-0.617903819
226	9	0	other	0.5	subcutances other days	high	medium	hard	-0.643869086
226	10	1	other	2.5	mascular once a weak	high	low	hard	0.00383736
226	10	0	iran	5.5	mascular once a weak	medium	medium	easy	-0.697626621
226	11	0	other	5.5	subcutances other days	high	medium	easy	-0.34108841
226	11	1	iran	0.5	subcutances three times a weak	medium	low	hard	-0.699689278
226	12	0	iran	5.5	subcutances three times a weak	high	medium	hard	-0.787756632
226	12	1	other	2.5	subcutances other days	medium	low	hard	-0.352164436
227	1	0	iran	5.5	mascular once a weak	high	medium	hard	-0.572088286
227	1	1	iran	0.5	subcutances three times a weak	high	medium	easy	-0.72867963
227	2	1	other	0.5	subcutances other days	high	medium	hard	-0.643869086
227	2	0	iran	5.5	subcutances other days	medium	medium	hard	-0.928090082
227	3	0	iran	5.5	mascular once a weak	high	medium	hard	-0.572088286
227	3	1	iran	2.5	subcutances three times a weak	high	medium	easy	-0.646894171
227	4	0	iran	0.5	subcutances three times a weak	medium	low	hard	-0.699689278
227	4	1	other	2.5	mascular once a weak	high	low	hard	0.00383736
227	5	0	iran	0.5	mascular once a weak	medium	low	easy	-0.303092094
227	5	1	other	2.5	subcutances three times a weak	high	low	easy	-0.030902146
227	6	1	other	2.5	subcutances three times a weak	high	low	easy	-0.030902146
227	6	0	other	0.5	subcutances other days	high	medium	easy	-0.462940247
227	7	0	other	0.5	mascular once a weak	medium	medium	hard	-0.900801637
227	7	1	other	5.5	subcutances other days	medium	low	hard	-0.312098058

227	8	0	iran	2.5	mascular once a weak	high	low	hard	-0.0957683
227	8	1	iran	5.5	subcutances three times a weak	high	low	easy	-0.090441429
227	9	0	iran	2.5	subcutances three times a weak	medium	low	hard	-0.617903819
227	9	1	other	0.5	subcutances other days	high	medium	hard	-0.643869086
227	10	0	other	2.5	mascular once a weak	high	low	hard	0.00383736
227	10	1	iran	5.5	mascular once a weak	medium	medium	easy	-0.697626621
227	11	1	other	5.5	subcutances other days	high	medium	easy	-0.34108841
227	11	0	iran	0.5	subcutances three times a weak	medium	low	hard	-0.699689278
227	12	0	iran	5.5	subcutances three times a weak	high	medium	hard	-0.787756632
227	12	1	other	2.5	subcutances other days	medium	low	hard	-0.352164436
228	1	1	iran	5.5	mascular once a weak	high	medium	hard	-0.572088286
228	1	0	iran	0.5	subcutances three times a weak	high	medium	easy	-0.72867963
228	2	0	other	0.5	subcutances other days	high	medium	hard	-0.643869086
228	2	1	iran	5.5	subcutances other days	medium	medium	hard	-0.928090082
228	3	0	iran	5.5	mascular once a weak	high	medium	hard	-0.572088286
228	3	1	iran	2.5	subcutances three times a weak	high	medium	easy	-0.646894171
228	4	1	iran	0.5	subcutances three times a weak	medium	low	hard	-0.699689278
228	4	0	other	2.5	mascular once a weak	high	low	hard	0.00383736
228	5	1	iran	0.5	mascular once a weak	medium	low	easy	-0.303092094
228	5	0	other	2.5	subcutances three times a weak	high	low	easy	-0.030902146
228	6	0	other	2.5	subcutances three times a weak	high	low	easy	-0.030902146
228	6	1	other	0.5	subcutances other days	high	medium	easy	-0.462940247
228	7	0	other	0.5	mascular once a weak	medium	medium	hard	-0.900801637
228	7	0	other	5.5	subcutances other days	medium	low	hard	-0.312098058
228	8	1	iran	2.5	mascular once a weak	high	low	hard	-0.0957683
228	8	0	iran	5.5	subcutances three times a weak	high	low	easy	-0.090441429
228	9	1	iran	2.5	subcutances three times a weak	medium	low	hard	-0.617903819
228	9	0	other	0.5	subcutances other days	high	medium	hard	-0.643869086
228	10	0	other	2.5	mascular once a weak	high	low	hard	0.00383736
228	10	1	iran	5.5	mascular once a weak	medium	medium	easy	-0.697626621
228	11	0	other	5.5	subcutances other days	high	medium	easy	-0.34108841
228	11	1	iran	0.5	subcutances three times a weak	medium	low	hard	-0.699689278
228	12	0	iran	5.5	subcutances three times a weak	high	medium	hard	-0.787756632
228	12	1	other	2.5	subcutances other days	medium	low	hard	-0.352164436

229	1	0	iran	5.5	mascular once a weak	high	medium	hard	-0.572088286
229	1	1	iran	0.5	subcutances three times a weak	high	medium	easy	-0.72867963
229	2	1	other	0.5	subcutances other days	high	medium	hard	-0.643869086
229	2	0	iran	5.5	subcutances other days	medium	medium	hard	-0.928090082
229	3	0	iran	5.5	mascular once a weak	high	medium	hard	-0.572088286
229	3	1	iran	2.5	subcutances three times a weak	high	medium	easy	-0.646894171
229	4	0	iran	0.5	subcutances three times a weak	medium	low	hard	-0.699689278
229	4	1	other	2.5	mascular once a weak	high	low	hard	0.00383736
229	5	0	iran	0.5	mascular once a weak	medium	low	easy	-0.303092094
229	5	1	other	2.5	subcutances three times a weak	high	low	easy	-0.030902146
229	6	1	other	2.5	subcutances three times a weak	high	low	easy	-0.030902146
229	6	0	other	0.5	subcutances other days	high	medium	easy	-0.462940247
229	7	1	other	0.5	mascular once a weak	medium	medium	hard	-0.900801637
229	7	0	other	5.5	subcutances other days	medium	low	hard	-0.312098058
229	8	1	iran	2.5	mascular once a weak	high	low	hard	-0.0957683
229	8	0	iran	5.5	subcutances three times a weak	high	low	easy	-0.090441429
229	9	0	iran	2.5	subcutances three times a weak	medium	low	hard	-0.617903819
229	9	1	other	0.5	subcutances other days	high	medium	hard	-0.643869086
229	10	1	other	2.5	mascular once a weak	high	low	hard	0.00383736
229	10	0	iran	5.5	mascular once a weak	medium	medium	easy	-0.697626621
229	11	1	other	5.5	subcutances other days	high	medium	easy	-0.34108841
229	11	0	iran	0.5	subcutances three times a weak	medium	low	hard	-0.699689278
229	12	1	iran	5.5	subcutances three times a weak	high	medium	hard	-0.787756632
229	12	0	other	2.5	subcutances other days	medium	low	hard	-0.352164436
230	1	1	iran	5.5	mascular once a weak	high	medium	hard	-0.572088286
230	1	0	iran	0.5	subcutances three times a weak	high	medium	easy	-0.72867963
230	2	1	other	0.5	subcutances other days	high	medium	hard	-0.643869086
230	2	0	iran	5.5	subcutances other days	medium	medium	hard	-0.928090082
230	3	1	iran	5.5	mascular once a weak	high	medium	hard	-0.572088286
230	3	0	iran	2.5	subcutances three times a weak	high	medium	easy	-0.646894171
230	4	0	iran	0.5	subcutances three times a weak	medium	low	hard	-0.699689278
230	4	1	other	2.5	mascular once a weak	high	low	hard	0.00383736
230	5	1	iran	0.5	mascular once a weak	medium	low	easy	-0.303092094
230	5	0	other	2.5	subcutances three times a weak	high	low	easy	-0.030902146

230	6	1	other	2.5	subcutances three times a weak	high	low	easy	-0.030902146
230	6	0	other	0.5	subcutances other days	high	medium	easy	-0.462940247
230	7	0	other	0.5	mascular once a weak	medium	medium	hard	-0.900801637
230	7	1	other	5.5	subcutances other days	medium	low	hard	-0.312098058
230	8	0	iran	2.5	mascular once a weak	high	low	hard	-0.0957683
230	8	1	iran	5.5	subcutances three times a weak	high	low	easy	-0.090441429
230	9	1	iran	2.5	subcutances three times a weak	medium	low	hard	-0.617903819
230	9	0	other	0.5	subcutances other days	high	medium	hard	-0.643869086
230	10	0	other	2.5	mascular once a weak	high	low	hard	0.00383736
230	10	1	iran	5.5	mascular once a weak	medium	medium	easy	-0.697626621
230	11	1	other	5.5	subcutances other days	high	medium	easy	-0.34108841
230	11	0	iran	0.5	subcutances three times a weak	medium	low	hard	-0.699689278
230	12	1	iran	5.5	subcutances three times a weak	high	medium	hard	-0.787756632
230	12	0	other	2.5	subcutances other days	medium	low	hard	-0.352164436
231	1	0	iran	5.5	mascular once a weak	high	medium	hard	-0.572088286
231	1	0	iran	0.5	subcutances three times a weak	high	medium	easy	-0.72867963
231	2	1	other	0.5	subcutances other days	high	medium	hard	-0.643869086
231	2	0	iran	5.5	subcutances other days	medium	medium	hard	-0.928090082
231	3	0	iran	5.5	mascular once a weak	high	medium	hard	-0.572088286
231	3	0	iran	2.5	subcutances three times a weak	high	medium	easy	-0.646894171
231	4	0	iran	0.5	subcutances three times a weak	medium	low	hard	-0.699689278
231	4	1	other	2.5	mascular once a weak	high	low	hard	0.00383736
231	5	0	iran	0.5	mascular once a weak	medium	low	easy	-0.303092094
231	5	1	other	2.5	subcutances three times a weak	high	low	easy	-0.030902146
231	6	1	other	2.5	subcutances three times a weak	high	low	easy	-0.030902146
231	6	0	other	0.5	subcutances other days	high	medium	easy	-0.462940247
231	7	0	other	0.5	mascular once a weak	medium	medium	hard	-0.900801637
231	7	1	other	5.5	subcutances other days	medium	low	hard	-0.312098058
231	8	0	iran	2.5	mascular once a weak	high	low	hard	-0.0957683
231	8	0	iran	5.5	subcutances three times a weak	high	low	easy	-0.090441429
231	9	0	iran	2.5	subcutances three times a weak	medium	low	hard	-0.617903819
231	9	1	other	0.5	subcutances other days	high	medium	hard	-0.643869086
231	10	1	other	2.5	mascular once a weak	high	low	hard	0.00383736
231	10	0	iran	5.5	mascular once a weak	medium	medium	easy	-0.697626621

231	11	1	other	5.5	subcutances other days	high	medium	easy	-0.34108841
231	11	0	iran	0.5	subcutances three times a weak	medium	low	hard	-0.699689278
231	12	0	iran	5.5	subcutances three times a weak	high	medium	hard	-0.787756632
231	12	1	other	2.5	subcutances other days	medium	low	hard	-0.352164436
232	1	0	iran	5.5	mascular once a weak	high	medium	hard	-0.572088286
232	1	1	iran	0.5	subcutances three times a weak	high	medium	easy	-0.72867963
232	2	1	other	0.5	subcutances other days	high	medium	hard	-0.643869086
232	2	0	iran	5.5	subcutances other days	medium	medium	hard	-0.928090082
232	3	0	iran	5.5	mascular once a weak	high	medium	hard	-0.572088286
232	3	1	iran	2.5	subcutances three times a weak	high	medium	easy	-0.646894171
232	4	0	iran	0.5	subcutances three times a weak	medium	low	hard	-0.699689278
232	4	1	other	2.5	mascular once a weak	high	low	hard	0.00383736
232	5	0	iran	0.5	mascular once a weak	medium	low	easy	-0.303092094
232	5	1	other	2.5	subcutances three times a weak	high	low	easy	-0.030902146
232	6	1	other	2.5	subcutances three times a weak	high	low	easy	-0.030902146
232	6	0	other	0.5	subcutances other days	high	medium	easy	-0.462940247
232	7	0	other	0.5	mascular once a weak	medium	medium	hard	-0.900801637
232	7	1	other	5.5	subcutances other days	medium	low	hard	-0.312098058
232	8	0	iran	2.5	mascular once a weak	high	low	hard	-0.0957683
232	8	1	iran	5.5	subcutances three times a weak	high	low	easy	-0.090441429
232	9	0	iran	2.5	subcutances three times a weak	medium	low	hard	-0.617903819
232	9	1	other	0.5	subcutances other days	high	medium	hard	-0.643869086
232	10	0	other	2.5	mascular once a weak	high	low	hard	0.00383736
232	10	1	iran	5.5	mascular once a weak	medium	medium	easy	-0.697626621
232	11	1	other	5.5	subcutances other days	high	medium	easy	-0.34108841
232	11	0	iran	0.5	subcutances three times a weak	medium	low	hard	-0.699689278
232	12	1	iran	5.5	subcutances three times a weak	high	medium	hard	-0.787756632
232	12	0	other	2.5	subcutances other days	medium	low	hard	-0.352164436
233	1	1	iran	5.5	mascular once a weak	high	medium	hard	-0.572088286
233	1	0	iran	0.5	subcutances three times a weak	high	medium	easy	-0.72867963
233	2	0	other	0.5	subcutances other days	high	medium	hard	-0.643869086
233	2	1	iran	5.5	subcutances other days	medium	medium	hard	-0.928090082
233	3	0	iran	5.5	mascular once a weak	high	medium	hard	-0.572088286
233	3	1	iran	2.5	subcutances three times a weak	high	medium	easy	-0.646894171

233	4	1	iran	0.5	subcutances	three times a weak	medium	low	hard	-0.699689278
233	4	0	other	2.5	mascular	once a weak	high	low	hard	0.00383736
233	5	1	iran	0.5	mascular	once a weak	medium	low	easy	-0.303092094
233	5	0	other	2.5	subcutances	three times a weak	high	low	easy	-0.030902146
233	6	1	other	2.5	subcutances	three times a weak	high	low	easy	-0.030902146
233	6	0	other	0.5	subcutances	other days	high	medium	easy	-0.462940247
233	7	1	other	0.5	mascular	once a weak	medium	medium	hard	-0.900801637
233	7	0	other	5.5	subcutances	other days	medium	low	hard	-0.312098058
233	8	1	iran	2.5	mascular	once a weak	high	low	hard	-0.0957683
233	8	0	iran	5.5	subcutances	three times a weak	high	low	easy	-0.090441429
233	9	1	iran	2.5	subcutances	three times a weak	medium	low	hard	-0.617903819
233	9	0	other	0.5	subcutances	other days	high	medium	hard	-0.643869086
233	10	0	other	2.5	mascular	once a weak	high	low	hard	0.00383736
233	10	1	iran	5.5	mascular	once a weak	medium	medium	easy	-0.697626621
233	11	0	other	5.5	subcutances	other days	high	medium	easy	-0.34108841
233	11	1	iran	0.5	subcutances	three times a weak	medium	low	hard	-0.699689278
233	12	1	iran	5.5	subcutances	three times a weak	high	medium	hard	-0.787756632
233	12	0	other	2.5	subcutances	other days	medium	low	hard	-0.352164436
234	1	1	iran	5.5	mascular	once a weak	high	medium	hard	-0.572088286
234	1	0	iran	0.5	subcutances	three times a weak	high	medium	easy	-0.72867963
234	2	1	other	0.5	subcutances	other days	high	medium	hard	-0.643869086
234	2	0	iran	5.5	subcutances	other days	medium	medium	hard	-0.928090082
234	3	0	iran	5.5	mascular	once a weak	high	medium	hard	-0.572088286
234	3	1	iran	2.5	subcutances	three times a weak	high	medium	easy	-0.646894171
234	4	0	iran	0.5	subcutances	three times a weak	medium	low	hard	-0.699689278
234	4	1	other	2.5	mascular	once a weak	high	low	hard	0.00383736
234	5	0	iran	0.5	mascular	once a weak	medium	low	easy	-0.303092094
234	5	1	other	2.5	subcutances	three times a weak	high	low	easy	-0.030902146
234	6	1	other	2.5	subcutances	three times a weak	high	low	easy	-0.030902146
234	6	0	other	0.5	subcutances	other days	high	medium	easy	-0.462940247
234	7	0	other	0.5	mascular	once a weak	medium	medium	hard	-0.900801637
234	7	1	other	5.5	subcutances	other days	medium	low	hard	-0.312098058
234	8	0	iran	2.5	mascular	once a weak	high	low	hard	-0.0957683
234	8	1	iran	5.5	subcutances	three times a weak	high	low	easy	-0.090441429

234	9	1	iran	2.5	subcutances three times a weak	medium	low	hard	-0.617903819
234	9	0	other	0.5	subcutances other days	high	medium	hard	-0.643869086
234	10	1	other	2.5	mascular once a weak	high	low	hard	0.00383736
234	10	0	iran	5.5	mascular once a weak	medium	medium	easy	-0.697626621
234	11	0	other	5.5	subcutances other days	high	medium	easy	-0.34108841
234	11	1	iran	0.5	subcutances three times a weak	medium	low	hard	-0.699689278
234	12	0	iran	5.5	subcutances three times a weak	high	medium	hard	-0.787756632
234	12	1	other	2.5	subcutances other days	medium	low	hard	-0.352164436
235	1	1	iran	5.5	mascular once a weak	high	medium	hard	-0.572088286
235	1	0	iran	0.5	subcutances three times a weak	high	medium	easy	-0.72867963
235	2	1	other	0.5	subcutances other days	high	medium	hard	-0.643869086
235	2	0	iran	5.5	subcutances other days	medium	medium	hard	-0.928090082
235	3	0	iran	5.5	mascular once a weak	high	medium	hard	-0.572088286
235	3	1	iran	2.5	subcutances three times a weak	high	medium	easy	-0.646894171
235	4	0	iran	0.5	subcutances three times a weak	medium	low	hard	-0.699689278
235	4	1	other	2.5	mascular once a weak	high	low	hard	0.00383736
235	5	0	iran	0.5	mascular once a weak	medium	low	easy	-0.303092094
235	5	1	other	2.5	subcutances three times a weak	high	low	easy	-0.030902146
235	6	1	other	2.5	subcutances three times a weak	high	low	easy	-0.030902146
235	6	0	other	0.5	subcutances other days	high	medium	easy	-0.462940247
235	7	0	other	0.5	mascular once a weak	medium	medium	hard	-0.900801637
235	7	1	other	5.5	subcutances other days	medium	low	hard	-0.312098058
235	8	1	iran	2.5	mascular once a weak	high	low	hard	-0.0957683
235	8	0	iran	5.5	subcutances three times a weak	high	low	easy	-0.090441429
235	9	1	iran	2.5	subcutances three times a weak	medium	low	hard	-0.617903819
235	9	0	other	0.5	subcutances other days	high	medium	hard	-0.643869086
235	10	1	other	2.5	mascular once a weak	high	low	hard	0.00383736
235	10	0	iran	5.5	mascular once a weak	medium	medium	easy	-0.697626621
235	11	0	other	5.5	subcutances other days	high	medium	easy	-0.34108841
235	11	1	iran	0.5	subcutances three times a weak	medium	low	hard	-0.699689278
235	12	0	iran	5.5	subcutances three times a weak	high	medium	hard	-0.787756632
235	12	1	other	2.5	subcutances other days	medium	low	hard	-0.352164436
236	1	1	iran	5.5	mascular once a weak	high	medium	hard	-0.572088286
236	1	0	iran	0.5	subcutances three times a weak	high	medium	easy	-0.72867963

236	2	1	other	0.5	subcutances	other days	high	medium	hard	-0.643869086
236	2	0	iran	5.5	subcutances	other days	medium	medium	hard	-0.928090082
236	3	1	iran	5.5	mascular	once a weak	high	medium	hard	-0.572088286
236	3	0	iran	2.5	subcutances	three times a weak	high	medium	easy	-0.646894171
236	4	0	iran	0.5	subcutances	three times a weak	medium	low	hard	-0.699689278
236	4	1	other	2.5	mascular	once a weak	high	low	hard	0.00383736
236	5	0	iran	0.5	mascular	once a weak	medium	low	easy	-0.303092094
236	5	1	other	2.5	subcutances	three times a weak	high	low	easy	-0.030902146
236	6	1	other	2.5	subcutances	three times a weak	high	low	easy	-0.030902146
236	6	0	other	0.5	subcutances	other days	high	medium	easy	-0.462940247
236	7	1	other	0.5	mascular	once a weak	medium	medium	hard	-0.900801637
236	7	0	other	5.5	subcutances	other days	medium	low	hard	-0.312098058
236	8	1	iran	2.5	mascular	once a weak	high	low	hard	-0.0957683
236	8	0	iran	5.5	subcutances	three times a weak	high	low	easy	-0.090441429
236	9	0	iran	2.5	subcutances	three times a weak	medium	low	hard	-0.617903819
236	9	1	other	0.5	subcutances	other days	high	medium	hard	-0.643869086
236	10	1	other	2.5	mascular	once a weak	high	low	hard	0.00383736
236	10	0	iran	5.5	mascular	once a weak	medium	medium	easy	-0.697626621
236	11	1	other	5.5	subcutances	other days	high	medium	easy	-0.34108841
236	11	0	iran	0.5	subcutances	three times a weak	medium	low	hard	-0.699689278
236	12	0	iran	5.5	subcutances	three times a weak	high	medium	hard	-0.787756632
236	12	1	other	2.5	subcutances	other days	medium	low	hard	-0.352164436
237	1	1	iran	5.5	mascular	once a weak	high	medium	hard	-0.572088286
237	1	0	iran	0.5	subcutances	three times a weak	high	medium	easy	-0.72867963
237	2	1	other	0.5	subcutances	other days	high	medium	hard	-0.643869086
237	2	0	iran	5.5	subcutances	other days	medium	medium	hard	-0.928090082
237	3	0	iran	5.5	mascular	once a weak	high	medium	hard	-0.572088286
237	3	1	iran	2.5	subcutances	three times a weak	high	medium	easy	-0.646894171
237	4	0	iran	0.5	subcutances	three times a weak	medium	low	hard	-0.699689278
237	4	1	other	2.5	mascular	once a weak	high	low	hard	0.00383736
237	5	0	iran	0.5	mascular	once a weak	medium	low	easy	-0.303092094
237	5	1	other	2.5	subcutances	three times a weak	high	low	easy	-0.030902146
237	6	1	other	2.5	subcutances	three times a weak	high	low	easy	-0.030902146
237	6	0	other	0.5	subcutances	other days	high	medium	easy	-0.462940247

237	7	0	other	0.5	mascular once a weak	medium	medium	hard	-0.900801637
237	7	1	other	5.5	subcutances other days	medium	low	hard	-0.312098058
237	8	1	iran	2.5	mascular once a weak	high	low	hard	-0.0957683
237	8	0	iran	5.5	subcutances three times a weak	high	low	easy	-0.090441429
237	9	0	iran	2.5	subcutances three times a weak	medium	low	hard	-0.617903819
237	9	1	other	0.5	subcutances other days	high	medium	hard	-0.643869086
237	10	1	other	2.5	mascular once a weak	high	low	hard	0.00383736
237	10	0	iran	5.5	mascular once a weak	medium	medium	easy	-0.697626621
237	11	1	other	5.5	subcutances other days	high	medium	easy	-0.34108841
237	11	0	iran	0.5	subcutances three times a weak	medium	low	hard	-0.699689278
237	12	0	iran	5.5	subcutances three times a weak	high	medium	hard	-0.787756632
237	12	1	other	2.5	subcutances other days	medium	low	hard	-0.352164436
238	1	1	iran	5.5	mascular once a weak	high	medium	hard	-0.572088286
238	1	0	iran	0.5	subcutances three times a weak	high	medium	easy	-0.72867963
238	2	1	other	0.5	subcutances other days	high	medium	hard	-0.643869086
238	2	0	iran	5.5	subcutances other days	medium	medium	hard	-0.928090082
238	3	0	iran	5.5	mascular once a weak	high	medium	hard	-0.572088286
238	3	1	iran	2.5	subcutances three times a weak	high	medium	easy	-0.646894171
238	4	0	iran	0.5	subcutances three times a weak	medium	low	hard	-0.699689278
238	4	1	other	2.5	mascular once a weak	high	low	hard	0.00383736
238	5	0	iran	0.5	mascular once a weak	medium	low	easy	-0.303092094
238	5	1	other	2.5	subcutances three times a weak	high	low	easy	-0.030902146
238	6	1	other	2.5	subcutances three times a weak	high	low	easy	-0.030902146
238	6	0	other	0.5	subcutances other days	high	medium	easy	-0.462940247
238	7	0	other	0.5	mascular once a weak	medium	medium	hard	-0.900801637
238	7	1	other	5.5	subcutances other days	medium	low	hard	-0.312098058
238	8	0	iran	2.5	mascular once a weak	high	low	hard	-0.0957683
238	8	1	iran	5.5	subcutances three times a weak	high	low	easy	-0.090441429
238	9	0	iran	2.5	subcutances three times a weak	medium	low	hard	-0.617903819
238	9	1	other	0.5	subcutances other days	high	medium	hard	-0.643869086
238	10	1	other	2.5	mascular once a weak	high	low	hard	0.00383736
238	10	0	iran	5.5	mascular once a weak	medium	medium	easy	-0.697626621
238	11	1	other	5.5	subcutances other days	high	medium	easy	-0.34108841
238	11	0	iran	0.5	subcutances three times a weak	medium	low	hard	-0.699689278

238	12	0	iran	5.5	subcutances three times a weak	high	medium	hard	-0.787756632
238	12	1	other	2.5	subcutances other days	medium	low	hard	-0.352164436
239	1	0	iran	5.5	mascular once a weak	high	medium	hard	-0.572088286
239	1	1	iran	0.5	subcutances three times a weak	high	medium	easy	-0.72867963
239	2	0	other	0.5	subcutances other days	high	medium	hard	-0.643869086
239	2	1	iran	5.5	subcutances other days	medium	medium	hard	-0.928090082
239	3	0	iran	5.5	mascular once a weak	high	medium	hard	-0.572088286
239	3	1	iran	2.5	subcutances three times a weak	high	medium	easy	-0.646894171
239	4	1	iran	0.5	subcutances three times a weak	medium	low	hard	-0.699689278
239	4	0	other	2.5	mascular once a weak	high	low	hard	0.00383736
239	5	0	iran	0.5	mascular once a weak	medium	low	easy	-0.303092094
239	5	1	other	2.5	subcutances three times a weak	high	low	easy	-0.030902146
239	6	0	other	2.5	subcutances three times a weak	high	low	easy	-0.030902146
239	6	1	other	0.5	subcutances other days	high	medium	easy	-0.462940247
239	7	0	other	0.5	mascular once a weak	medium	medium	hard	-0.900801637
239	7	1	other	5.5	subcutances other days	medium	low	hard	-0.312098058
239	8	0	iran	2.5	mascular once a weak	high	low	hard	-0.0957683
239	8	1	iran	5.5	subcutances three times a weak	high	low	easy	-0.090441429
239	9	1	iran	2.5	subcutances three times a weak	medium	low	hard	-0.617903819
239	9	0	other	0.5	subcutances other days	high	medium	hard	-0.643869086
239	10	0	other	2.5	mascular once a weak	high	low	hard	0.00383736
239	10	1	iran	5.5	mascular once a weak	medium	medium	easy	-0.697626621
239	11	0	other	5.5	subcutances other days	high	medium	easy	-0.34108841
239	11	1	iran	0.5	subcutances three times a weak	medium	low	hard	-0.699689278
239	12	0	iran	5.5	subcutances three times a weak	high	medium	hard	-0.787756632
239	12	1	other	2.5	subcutances other days	medium	low	hard	-0.352164436
240	1	1	iran	5.5	mascular once a weak	high	medium	hard	-0.572088286
240	1	0	iran	0.5	subcutances three times a weak	high	medium	easy	-0.72867963
240	2	0	other	0.5	subcutances other days	high	medium	hard	-0.643869086
240	2	1	iran	5.5	subcutances other days	medium	medium	hard	-0.928090082
240	3	0	iran	5.5	mascular once a weak	high	medium	hard	-0.572088286
240	3	1	iran	2.5	subcutances three times a weak	high	medium	easy	-0.646894171
240	4	1	iran	0.5	subcutances three times a weak	medium	low	hard	-0.699689278
240	4	0	other	2.5	mascular once a weak	high	low	hard	0.00383736

240	5	0	iran	0.5	mascular once a weak	medium	low	easy	-0.303092094
240	5	1	other	2.5	subcutances three times a weak	high	low	easy	-0.030902146
240	6	0	other	2.5	subcutances three times a weak	high	low	easy	-0.030902146
240	6	1	other	0.5	subcutances other days	high	medium	easy	-0.462940247
240	7	0	other	0.5	mascular once a weak	medium	medium	hard	-0.900801637
240	7	1	other	5.5	subcutances other days	medium	low	hard	-0.312098058
240	8	1	iran	2.5	mascular once a weak	high	low	hard	-0.0957683
240	8	0	iran	5.5	subcutances three times a weak	high	low	easy	-0.090441429
240	9	0	iran	2.5	subcutances three times a weak	medium	low	hard	-0.617903819
240	9	1	other	0.5	subcutances other days	high	medium	hard	-0.643869086
240	10	0	other	2.5	mascular once a weak	high	low	hard	0.00383736
240	10	1	iran	5.5	mascular once a weak	medium	medium	easy	-0.697626621
240	11	0	other	5.5	subcutances other days	high	medium	easy	-0.34108841
240	11	1	iran	0.5	subcutances three times a weak	medium	low	hard	-0.699689278
240	12	0	iran	5.5	subcutances three times a weak	high	medium	hard	-0.787756632
240	12	1	other	2.5	subcutances other days	medium	low	hard	-0.352164436
241	1	1	iran	5.5	mascular once a weak	high	medium	hard	-0.572088286
241	1	0	iran	0.5	subcutances three times a weak	high	medium	easy	-0.72867963
241	2	1	other	0.5	subcutances other days	high	medium	hard	-0.643869086
241	2	0	iran	5.5	subcutances other days	medium	medium	hard	-0.928090082
241	3	0	iran	5.5	mascular once a weak	high	medium	hard	-0.572088286
241	3	1	iran	2.5	subcutances three times a weak	high	medium	easy	-0.646894171
241	4	0	iran	0.5	subcutances three times a weak	medium	low	hard	-0.699689278
241	4	1	other	2.5	mascular once a weak	high	low	hard	0.00383736
241	5	0	iran	0.5	mascular once a weak	medium	low	easy	-0.303092094
241	5	1	other	2.5	subcutances three times a weak	high	low	easy	-0.030902146
241	6	1	other	2.5	subcutances three times a weak	high	low	easy	-0.030902146
241	6	0	other	0.5	subcutances other days	high	medium	easy	-0.462940247
241	7	0	other	0.5	mascular once a weak	medium	medium	hard	-0.900801637
241	7	1	other	5.5	subcutances other days	medium	low	hard	-0.312098058
241	8	0	iran	2.5	mascular once a weak	high	low	hard	-0.0957683
241	8	1	iran	5.5	subcutances three times a weak	high	low	easy	-0.090441429
241	9	0	iran	2.5	subcutances three times a weak	medium	low	hard	-0.617903819
241	9	1	other	0.5	subcutances other days	high	medium	hard	-0.643869086

241	10	1	other	2.5	mascular once a weak	high	low	hard	0.00383736
241	10	0	iran	5.5	mascular once a weak	medium	medium	easy	-0.697626621
241	11	1	other	5.5	subcutances other days	high	medium	easy	-0.34108841
241	11	0	iran	0.5	subcutances three times a weak	medium	low	hard	-0.699689278
241	12	0	iran	5.5	subcutances three times a weak	high	medium	hard	-0.787756632
241	12	1	other	2.5	subcutances other days	medium	low	hard	-0.352164436
242	1	0	iran	5.5	mascular once a weak	high	medium	hard	-0.572088286
242	1	1	iran	0.5	subcutances three times a weak	high	medium	easy	-0.72867963
242	2	0	other	0.5	subcutances other days	high	medium	hard	-0.643869086
242	2	1	iran	5.5	subcutances other days	medium	medium	hard	-0.928090082
242	3	0	iran	5.5	mascular once a weak	high	medium	hard	-0.572088286
242	3	1	iran	2.5	subcutances three times a weak	high	medium	easy	-0.646894171
242	4	0	iran	0.5	subcutances three times a weak	medium	low	hard	-0.699689278
242	4	1	other	2.5	mascular once a weak	high	low	hard	0.00383736
242	5	0	iran	0.5	mascular once a weak	medium	low	easy	-0.303092094
242	5	1	other	2.5	subcutances three times a weak	high	low	easy	-0.030902146
242	6	1	other	2.5	subcutances three times a weak	high	low	easy	-0.030902146
242	6	0	other	0.5	subcutances other days	high	medium	easy	-0.462940247
242	7	0	other	0.5	mascular once a weak	medium	medium	hard	-0.900801637
242	7	1	other	5.5	subcutances other days	medium	low	hard	-0.312098058
242	8	0	iran	2.5	mascular once a weak	high	low	hard	-0.0957683
242	8	1	iran	5.5	subcutances three times a weak	high	low	easy	-0.090441429
242	9	1	iran	2.5	subcutances three times a weak	medium	low	hard	-0.617903819
242	9	0	other	0.5	subcutances other days	high	medium	hard	-0.643869086
242	10	0	other	2.5	mascular once a weak	high	low	hard	0.00383736
242	10	1	iran	5.5	mascular once a weak	medium	medium	easy	-0.697626621
242	11	0	other	5.5	subcutances other days	high	medium	easy	-0.34108841
242	11	1	iran	0.5	subcutances three times a weak	medium	low	hard	-0.699689278
242	12	1	iran	5.5	subcutances three times a weak	high	medium	hard	-0.787756632
242	12	0	other	2.5	subcutances other days	medium	low	hard	-0.352164436
243	1	1	iran	5.5	mascular once a weak	high	medium	hard	-0.572088286
243	1	0	iran	0.5	subcutances three times a weak	high	medium	easy	-0.72867963
243	2	1	other	0.5	subcutances other days	high	medium	hard	-0.643869086
243	2	0	iran	5.5	subcutances other days	medium	medium	hard	-0.928090082

243	3	1	iran	5.5	mascular once a weak	high	medium	hard	-0.572088286
243	3	0	iran	2.5	subcutances three times a weak	high	medium	easy	-0.646894171
243	4	0	iran	0.5	subcutances three times a weak	medium	low	hard	-0.699689278
243	4	1	other	2.5	mascular once a weak	high	low	hard	0.00383736
243	5	0	iran	0.5	mascular once a weak	medium	low	easy	-0.303092094
243	5	1	other	2.5	subcutances three times a weak	high	low	easy	-0.030902146
243	6	1	other	2.5	subcutances three times a weak	high	low	easy	-0.030902146
243	6	0	other	0.5	subcutances other days	high	medium	easy	-0.462940247
243	7	0	other	0.5	mascular once a weak	medium	medium	hard	-0.900801637
243	7	1	other	5.5	subcutances other days	medium	low	hard	-0.312098058
243	8	1	iran	2.5	mascular once a weak	high	low	hard	-0.0957683
243	8	0	iran	5.5	subcutances three times a weak	high	low	easy	-0.090441429
243	9	1	iran	2.5	subcutances three times a weak	medium	low	hard	-0.617903819
243	9	0	other	0.5	subcutances other days	high	medium	hard	-0.643869086
243	10	1	other	2.5	mascular once a weak	high	low	hard	0.00383736
243	10	0	iran	5.5	mascular once a weak	medium	medium	easy	-0.697626621
243	11	1	other	5.5	subcutances other days	high	medium	easy	-0.34108841
243	11	0	iran	0.5	subcutances three times a weak	medium	low	hard	-0.699689278
243	12	1	iran	5.5	subcutances three times a weak	high	medium	hard	-0.787756632
243	12	0	other	2.5	subcutances other days	medium	low	hard	-0.352164436
244	1	1	iran	5.5	mascular once a weak	high	medium	hard	-0.572088286
244	1	0	iran	0.5	subcutances three times a weak	high	medium	easy	-0.72867963
244	2	1	other	0.5	subcutances other days	high	medium	hard	-0.643869086
244	2	0	iran	5.5	subcutances other days	medium	medium	hard	-0.928090082
244	3	1	iran	5.5	mascular once a weak	high	medium	hard	-0.572088286
244	3	0	iran	2.5	subcutances three times a weak	high	medium	easy	-0.646894171
244	4	0	iran	0.5	subcutances three times a weak	medium	low	hard	-0.699689278
244	4	1	other	2.5	mascular once a weak	high	low	hard	0.00383736
244	5	0	iran	0.5	mascular once a weak	medium	low	easy	-0.303092094
244	5	1	other	2.5	subcutances three times a weak	high	low	easy	-0.030902146
244	6	1	other	2.5	subcutances three times a weak	high	low	easy	-0.030902146
244	6	0	other	0.5	subcutances other days	high	medium	easy	-0.462940247
244	7	0	other	0.5	mascular once a weak	medium	medium	hard	-0.900801637
244	7	1	other	5.5	subcutances other days	medium	low	hard	-0.312098058

244	8	0	iran	2.5	mascular once a weak	high	low	hard	-0.0957683
244	8	1	iran	5.5	subcutances three times a weak	high	low	easy	-0.090441429
244	9	0	iran	2.5	subcutances three times a weak	medium	low	hard	-0.617903819
244	9	1	other	0.5	subcutances other days	high	medium	hard	-0.643869086
244	10	1	other	2.5	mascular once a weak	high	low	hard	0.00383736
244	10	0	iran	5.5	mascular once a weak	medium	medium	easy	-0.697626621
244	11	1	other	5.5	subcutances other days	high	medium	easy	-0.34108841
244	11	0	iran	0.5	subcutances three times a weak	medium	low	hard	-0.699689278
244	12	1	iran	5.5	subcutances three times a weak	high	medium	hard	-0.787756632
244	12	0	other	2.5	subcutances other days	medium	low	hard	-0.352164436
245	1	1	iran	5.5	mascular once a weak	high	medium	hard	-0.572088286
245	1	0	iran	0.5	subcutances three times a weak	high	medium	easy	-0.72867963
245	2	0	other	0.5	subcutances other days	high	medium	hard	-0.643869086
245	2	1	iran	5.5	subcutances other days	medium	medium	hard	-0.928090082
245	3	0	iran	5.5	mascular once a weak	high	medium	hard	-0.572088286
245	3	1	iran	2.5	subcutances three times a weak	high	medium	easy	-0.646894171
245	4	1	iran	0.5	subcutances three times a weak	medium	low	hard	-0.699689278
245	4	0	other	2.5	mascular once a weak	high	low	hard	0.00383736
245	5	0	iran	0.5	mascular once a weak	medium	low	easy	-0.303092094
245	5	1	other	2.5	subcutances three times a weak	high	low	easy	-0.030902146
245	6	0	other	2.5	subcutances three times a weak	high	low	easy	-0.030902146
245	6	1	other	0.5	subcutances other days	high	medium	easy	-0.462940247
245	7	0	other	0.5	mascular once a weak	medium	medium	hard	-0.900801637
245	7	1	other	5.5	subcutances other days	medium	low	hard	-0.312098058
245	8	1	iran	2.5	mascular once a weak	high	low	hard	-0.0957683
245	8	0	iran	5.5	subcutances three times a weak	high	low	easy	-0.090441429
245	9	1	iran	2.5	subcutances three times a weak	medium	low	hard	-0.617903819
245	9	0	other	0.5	subcutances other days	high	medium	hard	-0.643869086
245	10	1	other	2.5	mascular once a weak	high	low	hard	0.00383736
245	10	0	iran	5.5	mascular once a weak	medium	medium	easy	-0.697626621
245	11	0	other	5.5	subcutances other days	high	medium	easy	-0.34108841
245	11	1	iran	0.5	subcutances three times a weak	medium	low	hard	-0.699689278
245	12	0	iran	5.5	subcutances three times a weak	high	medium	hard	-0.787756632
245	12	1	other	2.5	subcutances other days	medium	low	hard	-0.352164436

246	1	1	iran	5.5	mascular once a weak	high	medium	hard	-0.572088286
246	1	0	iran	0.5	subcutances three times a weak	high	medium	easy	-0.72867963
246	2	0	other	0.5	subcutances other days	high	medium	hard	-0.643869086
246	2	1	iran	5.5	subcutances other days	medium	medium	hard	-0.928090082
246	3	0	iran	5.5	mascular once a weak	high	medium	hard	-0.572088286
246	3	1	iran	2.5	subcutances three times a weak	high	medium	easy	-0.646894171
246	4	1	iran	0.5	subcutances three times a weak	medium	low	hard	-0.699689278
246	4	0	other	2.5	mascular once a weak	high	low	hard	0.00383736
246	5	1	iran	0.5	mascular once a weak	medium	low	easy	-0.303092094
246	5	0	other	2.5	subcutances three times a weak	high	low	easy	-0.030902146
246	6	0	other	2.5	subcutances three times a weak	high	low	easy	-0.030902146
246	6	1	other	0.5	subcutances other days	high	medium	easy	-0.462940247
246	7	0	other	0.5	mascular once a weak	medium	medium	hard	-0.900801637
246	7	0	other	5.5	subcutances other days	medium	low	hard	-0.312098058
246	8	1	iran	2.5	mascular once a weak	high	low	hard	-0.0957683
246	8	0	iran	5.5	subcutances three times a weak	high	low	easy	-0.090441429
246	9	1	iran	2.5	subcutances three times a weak	medium	low	hard	-0.617903819
246	9	0	other	0.5	subcutances other days	high	medium	hard	-0.643869086
246	10	0	other	2.5	mascular once a weak	high	low	hard	0.00383736
246	10	1	iran	5.5	mascular once a weak	medium	medium	easy	-0.697626621
246	11	0	other	5.5	subcutances other days	high	medium	easy	-0.34108841
246	11	1	iran	0.5	subcutances three times a weak	medium	low	hard	-0.699689278
246	12	0	iran	5.5	subcutances three times a weak	high	medium	hard	-0.787756632
246	12	1	other	2.5	subcutances other days	medium	low	hard	-0.352164436
247	1	1	iran	5.5	mascular once a weak	high	medium	hard	-0.572088286
247	1	0	iran	0.5	subcutances three times a weak	high	medium	easy	-0.72867963
247	2	1	other	0.5	subcutances other days	high	medium	hard	-0.643869086
247	2	0	iran	5.5	subcutances other days	medium	medium	hard	-0.928090082
247	3	0	iran	5.5	mascular once a weak	high	medium	hard	-0.572088286
247	3	1	iran	2.5	subcutances three times a weak	high	medium	easy	-0.646894171
247	4	0	iran	0.5	subcutances three times a weak	medium	low	hard	-0.699689278
247	4	1	other	2.5	mascular once a weak	high	low	hard	0.00383736
247	5	0	iran	0.5	mascular once a weak	medium	low	easy	-0.303092094
247	5	1	other	2.5	subcutances three times a weak	high	low	easy	-0.030902146

247	6	1	other	2.5	subcutances three times a weak	high	low	easy	-0.030902146
247	6	0	other	0.5	subcutances other days	high	medium	easy	-0.462940247
247	7	0	other	0.5	mascular once a weak	medium	medium	hard	-0.900801637
247	7	1	other	5.5	subcutances other days	medium	low	hard	-0.312098058
247	8	0	iran	2.5	mascular once a weak	high	low	hard	-0.0957683
247	8	1	iran	5.5	subcutances three times a weak	high	low	easy	-0.090441429
247	9	0	iran	2.5	subcutances three times a weak	medium	low	hard	-0.617903819
247	9	1	other	0.5	subcutances other days	high	medium	hard	-0.643869086
247	10	1	other	2.5	mascular once a weak	high	low	hard	0.00383736
247	10	0	iran	5.5	mascular once a weak	medium	medium	easy	-0.697626621
247	11	1	other	5.5	subcutances other days	high	medium	easy	-0.34108841
247	11	0	iran	0.5	subcutances three times a weak	medium	low	hard	-0.699689278
247	12	1	iran	5.5	subcutances three times a weak	high	medium	hard	-0.787756632
247	12	0	other	2.5	subcutances other days	medium	low	hard	-0.352164436
248	1	1	iran	5.5	mascular once a weak	high	medium	hard	-0.572088286
248	1	0	iran	0.5	subcutances three times a weak	high	medium	easy	-0.72867963
248	2	1	other	0.5	subcutances other days	high	medium	hard	-0.643869086
248	2	0	iran	5.5	subcutances other days	medium	medium	hard	-0.928090082
248	3	1	iran	5.5	mascular once a weak	high	medium	hard	-0.572088286
248	3	0	iran	2.5	subcutances three times a weak	high	medium	easy	-0.646894171
248	4	0	iran	0.5	subcutances three times a weak	medium	low	hard	-0.699689278
248	4	1	other	2.5	mascular once a weak	high	low	hard	0.00383736
248	5	1	iran	0.5	mascular once a weak	medium	low	easy	-0.303092094
248	5	0	other	2.5	subcutances three times a weak	high	low	easy	-0.030902146
248	6	1	other	2.5	subcutances three times a weak	high	low	easy	-0.030902146
248	6	0	other	0.5	subcutances other days	high	medium	easy	-0.462940247
248	7	1	other	0.5	mascular once a weak	medium	medium	hard	-0.900801637
248	7	0	other	5.5	subcutances other days	medium	low	hard	-0.312098058
248	8	1	iran	2.5	mascular once a weak	high	low	hard	-0.0957683
248	8	0	iran	5.5	subcutances three times a weak	high	low	easy	-0.090441429
248	9	1	iran	2.5	subcutances three times a weak	medium	low	hard	-0.617903819
248	9	0	other	0.5	subcutances other days	high	medium	hard	-0.643869086
248	10	1	other	2.5	mascular once a weak	high	low	hard	0.00383736
248	10	0	iran	5.5	mascular once a weak	medium	medium	easy	-0.697626621

248	11	0	other	5.5	subcutances other days	high	medium	easy	-0.34108841
248	11	1	iran	0.5	subcutances three times a weak	medium	low	hard	-0.699689278
248	12	0	iran	5.5	subcutances three times a weak	high	medium	hard	-0.787756632
248	12	1	other	2.5	subcutances other days	medium	low	hard	-0.352164436
249	1	1	iran	5.5	mascular once a weak	high	medium	hard	-0.572088286
249	1	0	iran	0.5	subcutances three times a weak	high	medium	easy	-0.72867963
249	2	0	other	0.5	subcutances other days	high	medium	hard	-0.643869086
249	2	1	iran	5.5	subcutances other days	medium	medium	hard	-0.928090082
249	3	0	iran	5.5	mascular once a weak	high	medium	hard	-0.572088286
249	3	1	iran	2.5	subcutances three times a weak	high	medium	easy	-0.646894171
249	4	1	iran	0.5	subcutances three times a weak	medium	low	hard	-0.699689278
249	4	0	other	2.5	mascular once a weak	high	low	hard	0.00383736
249	5	1	iran	0.5	mascular once a weak	medium	low	easy	-0.303092094
249	5	0	other	2.5	subcutances three times a weak	high	low	easy	-0.030902146
249	6	0	other	2.5	subcutances three times a weak	high	low	easy	-0.030902146
249	6	1	other	0.5	subcutances other days	high	medium	easy	-0.462940247
249	7	0	other	0.5	mascular once a weak	medium	medium	hard	-0.900801637
249	7	0	other	5.5	subcutances other days	medium	low	hard	-0.312098058
249	8	1	iran	2.5	mascular once a weak	high	low	hard	-0.0957683
249	8	0	iran	5.5	subcutances three times a weak	high	low	easy	-0.090441429
249	9	1	iran	2.5	subcutances three times a weak	medium	low	hard	-0.617903819
249	9	0	other	0.5	subcutances other days	high	medium	hard	-0.643869086
249	10	0	other	2.5	mascular once a weak	high	low	hard	0.00383736
249	10	1	iran	5.5	mascular once a weak	medium	medium	easy	-0.697626621
249	11	0	other	5.5	subcutances other days	high	medium	easy	-0.34108841
249	11	1	iran	0.5	subcutances three times a weak	medium	low	hard	-0.699689278
249	12	0	iran	5.5	subcutances three times a weak	high	medium	hard	-0.787756632
249	12	1	other	2.5	subcutances other days	medium	low	hard	-0.352164436
250	1	1	iran	5.5	mascular once a weak	high	medium	hard	-0.572088286
250	1	0	iran	0.5	subcutances three times a weak	high	medium	easy	-0.72867963
250	2	0	other	0.5	subcutances other days	high	medium	hard	-0.643869086
250	2	1	iran	5.5	subcutances other days	medium	medium	hard	-0.928090082
250	3	0	iran	5.5	mascular once a weak	high	medium	hard	-0.572088286
250	3	1	iran	2.5	subcutances three times a weak	high	medium	easy	-0.646894171

250	4	1	iran	0.5	subcutances three times a weak	medium	low	hard	-0.699689278
250	4	0	other	2.5	mascular once a weak	high	low	hard	0.00383736
250	5	1	iran	0.5	mascular once a weak	medium	low	easy	-0.303092094
250	5	0	other	2.5	subcutances three times a weak	high	low	easy	-0.030902146
250	6	0	other	2.5	subcutances three times a weak	high	low	easy	-0.030902146
250	6	1	other	0.5	subcutances other days	high	medium	easy	-0.462940247
250	7	1	other	0.5	mascular once a weak	medium	medium	hard	-0.900801637
250	7	0	other	5.5	subcutances other days	medium	low	hard	-0.312098058
250	8	1	iran	2.5	mascular once a weak	high	low	hard	-0.0957683
250	8	0	iran	5.5	subcutances three times a weak	high	low	easy	-0.090441429
250	9	1	iran	2.5	subcutances three times a weak	medium	low	hard	-0.617903819
250	9	0	other	0.5	subcutances other days	high	medium	hard	-0.643869086
250	10	0	other	2.5	mascular once a weak	high	low	hard	0.00383736
250	10	1	iran	5.5	mascular once a weak	medium	medium	easy	-0.697626621
250	11	0	other	5.5	subcutances other days	high	medium	easy	-0.34108841
250	11	1	iran	0.5	subcutances three times a weak	medium	low	hard	-0.699689278
250	12	0	iran	5.5	subcutances three times a weak	high	medium	hard	-0.787756632
250	12	1	other	2.5	subcutances other days	medium	low	hard	-0.352164436
251	1	1	iran	5.5	mascular once a weak	high	medium	hard	-0.572088286
251	1	0	iran	0.5	subcutances three times a weak	high	medium	easy	-0.72867963
251	2	0	other	0.5	subcutances other days	high	medium	hard	-0.643869086
251	2	0	iran	5.5	subcutances other days	medium	medium	hard	-0.928090082
251	3	1	iran	5.5	mascular once a weak	high	medium	hard	-0.572088286
251	3	0	iran	2.5	subcutances three times a weak	high	medium	easy	-0.646894171
251	4	0	iran	0.5	subcutances three times a weak	medium	low	hard	-0.699689278
251	4	1	other	2.5	mascular once a weak	high	low	hard	0.00383736
251	5	1	iran	0.5	mascular once a weak	medium	low	easy	-0.303092094
251	5	0	other	2.5	subcutances three times a weak	high	low	easy	-0.030902146
251	6	0	other	2.5	subcutances three times a weak	high	low	easy	-0.030902146
251	6	0	other	0.5	subcutances other days	high	medium	easy	-0.462940247
251	7	1	other	0.5	mascular once a weak	medium	medium	hard	-0.900801637
251	7	0	other	5.5	subcutances other days	medium	low	hard	-0.312098058
251	8	1	iran	2.5	mascular once a weak	high	low	hard	-0.0957683
251	8	0	iran	5.5	subcutances three times a weak	high	low	easy	-0.090441429

251	9	0	iran	2.5	subcutances three times a weak	medium	low	hard	-0.617903819
251	9	0	other	0.5	subcutances other days	high	medium	hard	-0.643869086
251	10	1	other	2.5	mascular once a weak	high	low	hard	0.00383736
251	10	0	iran	5.5	mascular once a weak	medium	medium	easy	-0.697626621
251	11	0	other	5.5	subcutances other days	high	medium	easy	-0.34108841
251	11	0	iran	0.5	subcutances three times a weak	medium	low	hard	-0.699689278
251	12	0	iran	5.5	subcutances three times a weak	high	medium	hard	-0.787756632
251	12	0	other	2.5	subcutances other days	medium	low	hard	-0.352164436
252	1	0	iran	5.5	mascular once a weak	high	medium	hard	-0.572088286
252	1	1	iran	0.5	subcutances three times a weak	high	medium	easy	-0.72867963
252	2	1	other	0.5	subcutances other days	high	medium	hard	-0.643869086
252	2	0	iran	5.5	subcutances other days	medium	medium	hard	-0.928090082
252	3	0	iran	5.5	mascular once a weak	high	medium	hard	-0.572088286
252	3	1	iran	2.5	subcutances three times a weak	high	medium	easy	-0.646894171
252	4	0	iran	0.5	subcutances three times a weak	medium	low	hard	-0.699689278
252	4	1	other	2.5	mascular once a weak	high	low	hard	0.00383736
252	5	0	iran	0.5	mascular once a weak	medium	low	easy	-0.303092094
252	5	1	other	2.5	subcutances three times a weak	high	low	easy	-0.030902146
252	6	1	other	2.5	subcutances three times a weak	high	low	easy	-0.030902146
252	6	0	other	0.5	subcutances other days	high	medium	easy	-0.462940247
252	7	0	other	0.5	mascular once a weak	medium	medium	hard	-0.900801637
252	7	1	other	5.5	subcutances other days	medium	low	hard	-0.312098058
252	8	0	iran	2.5	mascular once a weak	high	low	hard	-0.0957683
252	8	1	iran	5.5	subcutances three times a weak	high	low	easy	-0.090441429
252	9	1	iran	2.5	subcutances three times a weak	medium	low	hard	-0.617903819
252	9	0	other	0.5	subcutances other days	high	medium	hard	-0.643869086
252	10	1	other	2.5	mascular once a weak	high	low	hard	0.00383736
252	10	0	iran	5.5	mascular once a weak	medium	medium	easy	-0.697626621
252	11	0	other	5.5	subcutances other days	high	medium	easy	-0.34108841
252	11	1	iran	0.5	subcutances three times a weak	medium	low	hard	-0.699689278
252	12	0	iran	5.5	subcutances three times a weak	high	medium	hard	-0.787756632
252	12	1	other	2.5	subcutances other days	medium	low	hard	-0.352164436
253	1	0	iran	5.5	mascular once a weak	high	medium	hard	-0.572088286
253	1	1	iran	0.5	subcutances three times a weak	high	medium	easy	-0.72867963

253	2	1	other	0.5	subcutances	other days	high	medium	hard	-0.643869086
253	2	0	iran	5.5	subcutances	other days	medium	medium	hard	-0.928090082
253	3	1	iran	5.5	mascular	once a weak	high	medium	hard	-0.572088286
253	3	0	iran	2.5	subcutances	three times a weak	high	medium	easy	-0.646894171
253	4	0	iran	0.5	subcutances	three times a weak	medium	low	hard	-0.699689278
253	4	1	other	2.5	mascular	once a weak	high	low	hard	0.00383736
253	5	1	iran	0.5	mascular	once a weak	medium	low	easy	-0.303092094
253	5	0	other	2.5	subcutances	three times a weak	high	low	easy	-0.030902146
253	6	0	other	2.5	subcutances	three times a weak	high	low	easy	-0.030902146
253	6	1	other	0.5	subcutances	other days	high	medium	easy	-0.462940247
253	7	0	other	0.5	mascular	once a weak	medium	medium	hard	-0.900801637
253	7	1	other	5.5	subcutances	other days	medium	low	hard	-0.312098058
253	8	1	iran	2.5	mascular	once a weak	high	low	hard	-0.0957683
253	8	0	iran	5.5	subcutances	three times a weak	high	low	easy	-0.090441429
253	9	1	iran	2.5	subcutances	three times a weak	medium	low	hard	-0.617903819
253	9	0	other	0.5	subcutances	other days	high	medium	hard	-0.643869086
253	10	1	other	2.5	mascular	once a weak	high	low	hard	0.00383736
253	10	0	iran	5.5	mascular	once a weak	medium	medium	easy	-0.697626621
253	11	1	other	5.5	subcutances	other days	high	medium	easy	-0.34108841
253	11	0	iran	0.5	subcutances	three times a weak	medium	low	hard	-0.699689278
253	12	0	iran	5.5	subcutances	three times a weak	high	medium	hard	-0.787756632
253	12	1	other	2.5	subcutances	other days	medium	low	hard	-0.352164436
254	1	1	iran	5.5	mascular	once a weak	high	medium	hard	-0.572088286
254	1	0	iran	0.5	subcutances	three times a weak	high	medium	easy	-0.72867963
254	2	0	other	0.5	subcutances	other days	high	medium	hard	-0.643869086
254	2	1	iran	5.5	subcutances	other days	medium	medium	hard	-0.928090082
254	3	0	iran	5.5	mascular	once a weak	high	medium	hard	-0.572088286
254	3	1	iran	2.5	subcutances	three times a weak	high	medium	easy	-0.646894171
254	4	1	iran	0.5	subcutances	three times a weak	medium	low	hard	-0.699689278
254	4	0	other	2.5	mascular	once a weak	high	low	hard	0.00383736
254	5	1	iran	0.5	mascular	once a weak	medium	low	easy	-0.303092094
254	5	0	other	2.5	subcutances	three times a weak	high	low	easy	-0.030902146
254	6	0	other	2.5	subcutances	three times a weak	high	low	easy	-0.030902146
254	6	1	other	0.5	subcutances	other days	high	medium	easy	-0.462940247

254	7	1	other	0.5	mascular once a weak	medium	medium	hard	-0.900801637
254	7	0	other	5.5	subcutances other days	medium	low	hard	-0.312098058
254	8	1	iran	2.5	mascular once a weak	high	low	hard	-0.0957683
254	8	0	iran	5.5	subcutances three times a weak	high	low	easy	-0.090441429
254	9	1	iran	2.5	subcutances three times a weak	medium	low	hard	-0.617903819
254	9	0	other	0.5	subcutances other days	high	medium	hard	-0.643869086
254	10	0	other	2.5	mascular once a weak	high	low	hard	0.00383736
254	10	1	iran	5.5	mascular once a weak	medium	medium	easy	-0.697626621
254	11	0	other	5.5	subcutances other days	high	medium	easy	-0.34108841
254	11	1	iran	0.5	subcutances three times a weak	medium	low	hard	-0.699689278
254	12	0	iran	5.5	subcutances three times a weak	high	medium	hard	-0.787756632
254	12	1	other	2.5	subcutances other days	medium	low	hard	-0.352164436
255	1	1	iran	5.5	mascular once a weak	high	medium	hard	-0.572088286
255	1	0	iran	0.5	subcutances three times a weak	high	medium	easy	-0.72867963
255	2	1	other	0.5	subcutances other days	high	medium	hard	-0.643869086
255	2	0	iran	5.5	subcutances other days	medium	medium	hard	-0.928090082
255	3	0	iran	5.5	mascular once a weak	high	medium	hard	-0.572088286
255	3	1	iran	2.5	subcutances three times a weak	high	medium	easy	-0.646894171
255	4	0	iran	0.5	subcutances three times a weak	medium	low	hard	-0.699689278
255	4	1	other	2.5	mascular once a weak	high	low	hard	0.00383736
255	5	0	iran	0.5	mascular once a weak	medium	low	easy	-0.303092094
255	5	1	other	2.5	subcutances three times a weak	high	low	easy	-0.030902146
255	6	1	other	2.5	subcutances three times a weak	high	low	easy	-0.030902146
255	6	0	other	0.5	subcutances other days	high	medium	easy	-0.462940247
255	7	0	other	0.5	mascular once a weak	medium	medium	hard	-0.900801637
255	7	1	other	5.5	subcutances other days	medium	low	hard	-0.312098058
255	8	1	iran	2.5	mascular once a weak	high	low	hard	-0.0957683
255	8	0	iran	5.5	subcutances three times a weak	high	low	easy	-0.090441429
255	9	0	iran	2.5	subcutances three times a weak	medium	low	hard	-0.617903819
255	9	1	other	0.5	subcutances other days	high	medium	hard	-0.643869086
255	10	1	other	2.5	mascular once a weak	high	low	hard	0.00383736
255	10	0	iran	5.5	mascular once a weak	medium	medium	easy	-0.697626621
255	11	1	other	5.5	subcutances other days	high	medium	easy	-0.34108841
255	11	0	iran	0.5	subcutances three times a weak	medium	low	hard	-0.699689278

255	12	1	iran	5.5	subcutances three times a weak	high	medium	hard	-0.787756632
255	12	0	other	2.5	subcutances other days	medium	low	hard	-0.352164436
256	1	1	iran	5.5	mascular once a weak	high	medium	hard	-0.572088286
256	1	0	iran	0.5	subcutances three times a weak	high	medium	easy	-0.72867963
256	2	1	other	0.5	subcutances other days	high	medium	hard	-0.643869086
256	2	0	iran	5.5	subcutances other days	medium	medium	hard	-0.928090082
256	3	0	iran	5.5	mascular once a weak	high	medium	hard	-0.572088286
256	3	1	iran	2.5	subcutances three times a weak	high	medium	easy	-0.646894171
256	4	0	iran	0.5	subcutances three times a weak	medium	low	hard	-0.699689278
256	4	1	other	2.5	mascular once a weak	high	low	hard	0.00383736
256	5	0	iran	0.5	mascular once a weak	medium	low	easy	-0.303092094
256	5	1	other	2.5	subcutances three times a weak	high	low	easy	-0.030902146
256	6	1	other	2.5	subcutances three times a weak	high	low	easy	-0.030902146
256	6	0	other	0.5	subcutances other days	high	medium	easy	-0.462940247
256	7	0	other	0.5	mascular once a weak	medium	medium	hard	-0.900801637
256	7	1	other	5.5	subcutances other days	medium	low	hard	-0.312098058
256	8	1	iran	2.5	mascular once a weak	high	low	hard	-0.0957683
256	8	0	iran	5.5	subcutances three times a weak	high	low	easy	-0.090441429
256	9	1	iran	2.5	subcutances three times a weak	medium	low	hard	-0.617903819
256	9	0	other	0.5	subcutances other days	high	medium	hard	-0.643869086
256	10	1	other	2.5	mascular once a weak	high	low	hard	0.00383736
256	10	0	iran	5.5	mascular once a weak	medium	medium	easy	-0.697626621
256	11	1	other	5.5	subcutances other days	high	medium	easy	-0.34108841
256	11	0	iran	0.5	subcutances three times a weak	medium	low	hard	-0.699689278
256	12	1	iran	5.5	subcutances three times a weak	high	medium	hard	-0.787756632
256	12	0	other	2.5	subcutances other days	medium	low	hard	-0.352164436
257	1	0	iran	5.5	mascular once a weak	high	medium	hard	-0.572088286
257	1	1	iran	0.5	subcutances three times a weak	high	medium	easy	-0.72867963
257	2	1	other	0.5	subcutances other days	high	medium	hard	-0.643869086
257	2	0	iran	5.5	subcutances other days	medium	medium	hard	-0.928090082
257	3	0	iran	5.5	mascular once a weak	high	medium	hard	-0.572088286
257	3	1	iran	2.5	subcutances three times a weak	high	medium	easy	-0.646894171
257	4	1	iran	0.5	subcutances three times a weak	medium	low	hard	-0.699689278
257	4	0	other	2.5	mascular once a weak	high	low	hard	0.00383736

257	5	0	iran	0.5	mascular once a weak	medium	low	easy	-0.303092094
257	5	1	other	2.5	subcutances three times a weak	high	low	easy	-0.030902146
257	6	1	other	2.5	subcutances three times a weak	high	low	easy	-0.030902146
257	6	0	other	0.5	subcutances other days	high	medium	easy	-0.462940247
257	7	1	other	0.5	mascular once a weak	medium	medium	hard	-0.900801637
257	7	0	other	5.5	subcutances other days	medium	low	hard	-0.312098058
257	8	0	iran	2.5	mascular once a weak	high	low	hard	-0.0957683
257	8	1	iran	5.5	subcutances three times a weak	high	low	easy	-0.090441429
257	9	0	iran	2.5	subcutances three times a weak	medium	low	hard	-0.617903819
257	9	1	other	0.5	subcutances other days	high	medium	hard	-0.643869086
257	10	0	other	2.5	mascular once a weak	high	low	hard	0.00383736
257	10	1	iran	5.5	mascular once a weak	medium	medium	easy	-0.697626621
257	11	0	other	5.5	subcutances other days	high	medium	easy	-0.34108841
257	11	1	iran	0.5	subcutances three times a weak	medium	low	hard	-0.699689278
257	12	1	iran	5.5	subcutances three times a weak	high	medium	hard	-0.787756632
257	12	0	other	2.5	subcutances other days	medium	low	hard	-0.352164436
258	1	1	iran	5.5	mascular once a weak	high	medium	hard	-0.572088286
258	1	0	iran	0.5	subcutances three times a weak	high	medium	easy	-0.72867963
258	2	1	other	0.5	subcutances other days	high	medium	hard	-0.643869086
258	2	0	iran	5.5	subcutances other days	medium	medium	hard	-0.928090082
258	3	0	iran	5.5	mascular once a weak	high	medium	hard	-0.572088286
258	3	1	iran	2.5	subcutances three times a weak	high	medium	easy	-0.646894171
258	4	0	iran	0.5	subcutances three times a weak	medium	low	hard	-0.699689278
258	4	1	other	2.5	mascular once a weak	high	low	hard	0.00383736
258	5	0	iran	0.5	mascular once a weak	medium	low	easy	-0.303092094
258	5	1	other	2.5	subcutances three times a weak	high	low	easy	-0.030902146
258	6	0	other	2.5	subcutances three times a weak	high	low	easy	-0.030902146
258	6	1	other	0.5	subcutances other days	high	medium	easy	-0.462940247
258	7	1	other	0.5	mascular once a weak	medium	medium	hard	-0.900801637
258	7	0	other	5.5	subcutances other days	medium	low	hard	-0.312098058
258	8	1	iran	2.5	mascular once a weak	high	low	hard	-0.0957683
258	8	0	iran	5.5	subcutances three times a weak	high	low	easy	-0.090441429
258	9	1	iran	2.5	subcutances three times a weak	medium	low	hard	-0.617903819
258	9	0	other	0.5	subcutances other days	high	medium	hard	-0.643869086

258	10	0	other	2.5	mascular once a weak	high	low	hard	0.00383736
258	10	1	iran	5.5	mascular once a weak	medium	medium	easy	-0.697626621
258	11	1	other	5.5	subcutances other days	high	medium	easy	-0.34108841
258	11	0	iran	0.5	subcutances three times a weak	medium	low	hard	-0.699689278
258	12	1	iran	5.5	subcutances three times a weak	high	medium	hard	-0.787756632
258	12	0	other	2.5	subcutances other days	medium	low	hard	-0.352164436
259	1	1	iran	5.5	mascular once a weak	high	medium	hard	-0.572088286
259	1	0	iran	0.5	subcutances three times a weak	high	medium	easy	-0.72867963
259	2	1	other	0.5	subcutances other days	high	medium	hard	-0.643869086
259	2	0	iran	5.5	subcutances other days	medium	medium	hard	-0.928090082
259	3	0	iran	5.5	mascular once a weak	high	medium	hard	-0.572088286
259	3	1	iran	2.5	subcutances three times a weak	high	medium	easy	-0.646894171
259	4	0	iran	0.5	subcutances three times a weak	medium	low	hard	-0.699689278
259	4	1	other	2.5	mascular once a weak	high	low	hard	0.00383736
259	5	0	iran	0.5	mascular once a weak	medium	low	easy	-0.303092094
259	5	1	other	2.5	subcutances three times a weak	high	low	easy	-0.030902146
259	6	0	other	2.5	subcutances three times a weak	high	low	easy	-0.030902146
259	6	1	other	0.5	subcutances other days	high	medium	easy	-0.462940247
259	7	0	other	0.5	mascular once a weak	medium	medium	hard	-0.900801637
259	7	1	other	5.5	subcutances other days	medium	low	hard	-0.312098058
259	8	1	iran	2.5	mascular once a weak	high	low	hard	-0.0957683
259	8	0	iran	5.5	subcutances three times a weak	high	low	easy	-0.090441429
259	9	0	iran	2.5	subcutances three times a weak	medium	low	hard	-0.617903819
259	9	1	other	0.5	subcutances other days	high	medium	hard	-0.643869086
259	10	1	other	2.5	mascular once a weak	high	low	hard	0.00383736
259	10	0	iran	5.5	mascular once a weak	medium	medium	easy	-0.697626621
259	11	1	other	5.5	subcutances other days	high	medium	easy	-0.34108841
259	11	0	iran	0.5	subcutances three times a weak	medium	low	hard	-0.699689278
259	12	1	iran	5.5	subcutances three times a weak	high	medium	hard	-0.787756632
259	12	0	other	2.5	subcutances other days	medium	low	hard	-0.352164436
260	1	1	iran	5.5	mascular once a weak	high	medium	hard	-0.572088286
260	1	0	iran	0.5	subcutances three times a weak	high	medium	easy	-0.72867963
260	2	0	other	0.5	subcutances other days	high	medium	hard	-0.643869086
260	2	1	iran	5.5	subcutances other days	medium	medium	hard	-0.928090082

260	3	0	iran	5.5	mascular once a weak	high	medium	hard	-0.572088286
260	3	1	iran	2.5	subcutances three times a weak	high	medium	easy	-0.646894171
260	4	1	iran	0.5	subcutances three times a weak	medium	low	hard	-0.699689278
260	4	0	other	2.5	mascular once a weak	high	low	hard	0.00383736
260	5	1	iran	0.5	mascular once a weak	medium	low	easy	-0.303092094
260	5	0	other	2.5	subcutances three times a weak	high	low	easy	-0.030902146
260	6	1	other	2.5	subcutances three times a weak	high	low	easy	-0.030902146
260	6	0	other	0.5	subcutances other days	high	medium	easy	-0.462940247
260	7	0	other	0.5	mascular once a weak	medium	medium	hard	-0.900801637
260	7	1	other	5.5	subcutances other days	medium	low	hard	-0.312098058
260	8	1	iran	2.5	mascular once a weak	high	low	hard	-0.0957683
260	8	0	iran	5.5	subcutances three times a weak	high	low	easy	-0.090441429
260	9	1	iran	2.5	subcutances three times a weak	medium	low	hard	-0.617903819
260	9	0	other	0.5	subcutances other days	high	medium	hard	-0.643869086
260	10	1	other	2.5	mascular once a weak	high	low	hard	0.00383736
260	10	0	iran	5.5	mascular once a weak	medium	medium	easy	-0.697626621
260	11	0	other	5.5	subcutances other days	high	medium	easy	-0.34108841
260	11	1	iran	0.5	subcutances three times a weak	medium	low	hard	-0.699689278
260	12	0	iran	5.5	subcutances three times a weak	high	medium	hard	-0.787756632
260	12	1	other	2.5	subcutances other days	medium	low	hard	-0.352164436
261	1	1	iran	5.5	mascular once a weak	high	medium	hard	-0.572088286
261	1	0	iran	0.5	subcutances three times a weak	high	medium	easy	-0.72867963
261	2	1	other	0.5	subcutances other days	high	medium	hard	-0.643869086
261	2	0	iran	5.5	subcutances other days	medium	medium	hard	-0.928090082
261	3	1	iran	5.5	mascular once a weak	high	medium	hard	-0.572088286
261	3	0	iran	2.5	subcutances three times a weak	high	medium	easy	-0.646894171
261	4	0	iran	0.5	subcutances three times a weak	medium	low	hard	-0.699689278
261	4	1	other	2.5	mascular once a weak	high	low	hard	0.00383736
261	5	0	iran	0.5	mascular once a weak	medium	low	easy	-0.303092094
261	5	1	other	2.5	subcutances three times a weak	high	low	easy	-0.030902146
261	6	1	other	2.5	subcutances three times a weak	high	low	easy	-0.030902146
261	6	0	other	0.5	subcutances other days	high	medium	easy	-0.462940247
261	7	0	other	0.5	mascular once a weak	medium	medium	hard	-0.900801637
261	7	1	other	5.5	subcutances other days	medium	low	hard	-0.312098058

261	8	1	iran	2.5	mascular once a weak	high	low	hard	-0.0957683
261	8	0	iran	5.5	subcutances three times a weak	high	low	easy	-0.090441429
261	9	0	iran	2.5	subcutances three times a weak	medium	low	hard	-0.617903819
261	9	1	other	0.5	subcutances other days	high	medium	hard	-0.643869086
261	10	0	other	2.5	mascular once a weak	high	low	hard	0.00383736
261	10	1	iran	5.5	mascular once a weak	medium	medium	easy	-0.697626621
261	11	0	other	5.5	subcutances other days	high	medium	easy	-0.34108841
261	11	1	iran	0.5	subcutances three times a weak	medium	low	hard	-0.699689278
261	12	1	iran	5.5	subcutances three times a weak	high	medium	hard	-0.787756632
261	12	0	other	2.5	subcutances other days	medium	low	hard	-0.352164436
262	1	1	iran	5.5	mascular once a weak	high	medium	hard	-0.572088286
262	1	0	iran	0.5	subcutances three times a weak	high	medium	easy	-0.72867963
262	2	1	other	0.5	subcutances other days	high	medium	hard	-0.643869086
262	2	0	iran	5.5	subcutances other days	medium	medium	hard	-0.928090082
262	3	1	iran	5.5	mascular once a weak	high	medium	hard	-0.572088286
262	3	0	iran	2.5	subcutances three times a weak	high	medium	easy	-0.646894171
262	4	0	iran	0.5	subcutances three times a weak	medium	low	hard	-0.699689278
262	4	1	other	2.5	mascular once a weak	high	low	hard	0.00383736
262	5	1	iran	0.5	mascular once a weak	medium	low	easy	-0.303092094
262	5	0	other	2.5	subcutances three times a weak	high	low	easy	-0.030902146
262	6	1	other	2.5	subcutances three times a weak	high	low	easy	-0.030902146
262	6	0	other	0.5	subcutances other days	high	medium	easy	-0.462940247
262	7	1	other	0.5	mascular once a weak	medium	medium	hard	-0.900801637
262	7	0	other	5.5	subcutances other days	medium	low	hard	-0.312098058
262	8	1	iran	2.5	mascular once a weak	high	low	hard	-0.0957683
262	8	0	iran	5.5	subcutances three times a weak	high	low	easy	-0.090441429
262	9	1	iran	2.5	subcutances three times a weak	medium	low	hard	-0.617903819
262	9	0	other	0.5	subcutances other days	high	medium	hard	-0.643869086
262	10	1	other	2.5	mascular once a weak	high	low	hard	0.00383736
262	10	0	iran	5.5	mascular once a weak	medium	medium	easy	-0.697626621
262	11	0	other	5.5	subcutances other days	high	medium	easy	-0.34108841
262	11	1	iran	0.5	subcutances three times a weak	medium	low	hard	-0.699689278
262	12	0	iran	5.5	subcutances three times a weak	high	medium	hard	-0.787756632
262	12	1	other	2.5	subcutances other days	medium	low	hard	-0.352164436

263	1	1	iran	5.5	mascular once a weak	high	medium	hard	-0.572088286
263	1	0	iran	0.5	subcutances three times a weak	high	medium	easy	-0.72867963
263	2	1	other	0.5	subcutances other days	high	medium	hard	-0.643869086
263	2	0	iran	5.5	subcutances other days	medium	medium	hard	-0.928090082
263	3	0	iran	5.5	mascular once a weak	high	medium	hard	-0.572088286
263	3	1	iran	2.5	subcutances three times a weak	high	medium	easy	-0.646894171
263	4	0	iran	0.5	subcutances three times a weak	medium	low	hard	-0.699689278
263	4	1	other	2.5	mascular once a weak	high	low	hard	0.00383736
263	5	0	iran	0.5	mascular once a weak	medium	low	easy	-0.303092094
263	5	1	other	2.5	subcutances three times a weak	high	low	easy	-0.030902146
263	6	1	other	2.5	subcutances three times a weak	high	low	easy	-0.030902146
263	6	0	other	0.5	subcutances other days	high	medium	easy	-0.462940247
263	7	0	other	0.5	mascular once a weak	medium	medium	hard	-0.900801637
263	7	1	other	5.5	subcutances other days	medium	low	hard	-0.312098058
263	8	1	iran	2.5	mascular once a weak	high	low	hard	-0.0957683
263	8	0	iran	5.5	subcutances three times a weak	high	low	easy	-0.090441429
263	9	0	iran	2.5	subcutances three times a weak	medium	low	hard	-0.617903819
263	9	1	other	0.5	subcutances other days	high	medium	hard	-0.643869086
263	10	1	other	2.5	mascular once a weak	high	low	hard	0.00383736
263	10	0	iran	5.5	mascular once a weak	medium	medium	easy	-0.697626621
263	11	1	other	5.5	subcutances other days	high	medium	easy	-0.34108841
263	11	0	iran	0.5	subcutances three times a weak	medium	low	hard	-0.699689278
263	12	1	iran	5.5	subcutances three times a weak	high	medium	hard	-0.787756632
263	12	0	other	2.5	subcutances other days	medium	low	hard	-0.352164436
264	1	1	iran	5.5	mascular once a weak	high	medium	hard	-0.572088286
264	1	0	iran	0.5	subcutances three times a weak	high	medium	easy	-0.72867963
264	2	1	other	0.5	subcutances other days	high	medium	hard	-0.643869086
264	2	0	iran	5.5	subcutances other days	medium	medium	hard	-0.928090082
264	3	1	iran	5.5	mascular once a weak	high	medium	hard	-0.572088286
264	3	0	iran	2.5	subcutances three times a weak	high	medium	easy	-0.646894171
264	4	0	iran	0.5	subcutances three times a weak	medium	low	hard	-0.699689278
264	4	1	other	2.5	mascular once a weak	high	low	hard	0.00383736
264	5	1	iran	0.5	mascular once a weak	medium	low	easy	-0.303092094
264	5	0	other	2.5	subcutances three times a weak	high	low	easy	-0.030902146

264	6	1	other	2.5	subcutances three times a weak	high	low	easy	-0.030902146
264	6	0	other	0.5	subcutances other days	high	medium	easy	-0.462940247
264	7	0	other	0.5	mascular once a weak	medium	medium	hard	-0.900801637
264	7	1	other	5.5	subcutances other days	medium	low	hard	-0.312098058
264	8	1	iran	2.5	mascular once a weak	high	low	hard	-0.0957683
264	8	0	iran	5.5	subcutances three times a weak	high	low	easy	-0.090441429
264	9	0	iran	2.5	subcutances three times a weak	medium	low	hard	-0.617903819
264	9	1	other	0.5	subcutances other days	high	medium	hard	-0.643869086
264	10	1	other	2.5	mascular once a weak	high	low	hard	0.00383736
264	10	0	iran	5.5	mascular once a weak	medium	medium	easy	-0.697626621
264	11	1	other	5.5	subcutances other days	high	medium	easy	-0.34108841
264	11	0	iran	0.5	subcutances three times a weak	medium	low	hard	-0.699689278
264	12	1	iran	5.5	subcutances three times a weak	high	medium	hard	-0.787756632
264	12	0	other	2.5	subcutances other days	medium	low	hard	-0.352164436
265	1	1	iran	5.5	mascular once a weak	high	medium	hard	-0.572088286
265	1	0	iran	0.5	subcutances three times a weak	high	medium	easy	-0.72867963
265	2	1	other	0.5	subcutances other days	high	medium	hard	-0.643869086
265	2	0	iran	5.5	subcutances other days	medium	medium	hard	-0.928090082
265	3	1	iran	5.5	mascular once a weak	high	medium	hard	-0.572088286
265	3	0	iran	2.5	subcutances three times a weak	high	medium	easy	-0.646894171
265	4	0	iran	0.5	subcutances three times a weak	medium	low	hard	-0.699689278
265	4	1	other	2.5	mascular once a weak	high	low	hard	0.00383736
265	5	0	iran	0.5	mascular once a weak	medium	low	easy	-0.303092094
265	5	1	other	2.5	subcutances three times a weak	high	low	easy	-0.030902146
265	6	1	other	2.5	subcutances three times a weak	high	low	easy	-0.030902146
265	6	0	other	0.5	subcutances other days	high	medium	easy	-0.462940247
265	7	0	other	0.5	mascular once a weak	medium	medium	hard	-0.900801637
265	7	1	other	5.5	subcutances other days	medium	low	hard	-0.312098058
265	8	1	iran	2.5	mascular once a weak	high	low	hard	-0.0957683
265	8	0	iran	5.5	subcutances three times a weak	high	low	easy	-0.090441429
265	9	0	iran	2.5	subcutances three times a weak	medium	low	hard	-0.617903819
265	9	1	other	0.5	subcutances other days	high	medium	hard	-0.643869086
265	10	1	other	2.5	mascular once a weak	high	low	hard	0.00383736
265	10	0	iran	5.5	mascular once a weak	medium	medium	easy	-0.697626621

265	11	1	other	5.5	subcutances other days	high	medium	easy	-0.34108841
265	11	0	iran	0.5	subcutances three times a weak	medium	low	hard	-0.699689278
265	12	1	iran	5.5	subcutances three times a weak	high	medium	hard	-0.787756632
265	12	0	other	2.5	subcutances other days	medium	low	hard	-0.352164436
266	1	0	iran	5.5	mascular once a weak	high	medium	hard	-0.572088286
266	1	1	iran	0.5	subcutances three times a weak	high	medium	easy	-0.72867963
266	2	1	other	0.5	subcutances other days	high	medium	hard	-0.643869086
266	2	0	iran	5.5	subcutances other days	medium	medium	hard	-0.928090082
266	3	0	iran	5.5	mascular once a weak	high	medium	hard	-0.572088286
266	3	1	iran	2.5	subcutances three times a weak	high	medium	easy	-0.646894171
266	4	0	iran	0.5	subcutances three times a weak	medium	low	hard	-0.699689278
266	4	1	other	2.5	mascular once a weak	high	low	hard	0.00383736
266	5	0	iran	0.5	mascular once a weak	medium	low	easy	-0.303092094
266	5	1	other	2.5	subcutances three times a weak	high	low	easy	-0.030902146
266	6	1	other	2.5	subcutances three times a weak	high	low	easy	-0.030902146
266	6	0	other	0.5	subcutances other days	high	medium	easy	-0.462940247
266	7	0	other	0.5	mascular once a weak	medium	medium	hard	-0.900801637
266	7	1	other	5.5	subcutances other days	medium	low	hard	-0.312098058
266	8	0	iran	2.5	mascular once a weak	high	low	hard	-0.0957683
266	8	1	iran	5.5	subcutances three times a weak	high	low	easy	-0.090441429
266	9	1	iran	2.5	subcutances three times a weak	medium	low	hard	-0.617903819
266	9	0	other	0.5	subcutances other days	high	medium	hard	-0.643869086
266	10	1	other	2.5	mascular once a weak	high	low	hard	0.00383736
266	10	0	iran	5.5	mascular once a weak	medium	medium	easy	-0.697626621
266	11	1	other	5.5	subcutances other days	high	medium	easy	-0.34108841
266	11	0	iran	0.5	subcutances three times a weak	medium	low	hard	-0.699689278
266	12	0	iran	5.5	subcutances three times a weak	high	medium	hard	-0.787756632
266	12	1	other	2.5	subcutances other days	medium	low	hard	-0.352164436
267	1	0	iran	5.5	mascular once a weak	high	medium	hard	-0.572088286
267	1	1	iran	0.5	subcutances three times a weak	high	medium	easy	-0.72867963
267	2	1	other	0.5	subcutances other days	high	medium	hard	-0.643869086
267	2	0	iran	5.5	subcutances other days	medium	medium	hard	-0.928090082
267	3	0	iran	5.5	mascular once a weak	high	medium	hard	-0.572088286
267	3	1	iran	2.5	subcutances three times a weak	high	medium	easy	-0.646894171

267	4	0	iran	0.5	subcutances three times a weak	medium	low	hard	-0.699689278
267	4	1	other	2.5	mascular once a weak	high	low	hard	0.00383736
267	5	0	iran	0.5	mascular once a weak	medium	low	easy	-0.303092094
267	5	1	other	2.5	subcutances three times a weak	high	low	easy	-0.030902146
267	6	1	other	2.5	subcutances three times a weak	high	low	easy	-0.030902146
267	6	0	other	0.5	subcutances other days	high	medium	easy	-0.462940247
267	7	0	other	0.5	mascular once a weak	medium	medium	hard	-0.900801637
267	7	1	other	5.5	subcutances other days	medium	low	hard	-0.312098058
267	8	0	iran	2.5	mascular once a weak	high	low	hard	-0.0957683
267	8	1	iran	5.5	subcutances three times a weak	high	low	easy	-0.090441429
267	9	1	iran	2.5	subcutances three times a weak	medium	low	hard	-0.617903819
267	9	0	other	0.5	subcutances other days	high	medium	hard	-0.643869086
267	10	0	other	2.5	mascular once a weak	high	low	hard	0.00383736
267	10	1	iran	5.5	mascular once a weak	medium	medium	easy	-0.697626621
267	11	1	other	5.5	subcutances other days	high	medium	easy	-0.34108841
267	11	0	iran	0.5	subcutances three times a weak	medium	low	hard	-0.699689278
267	12	1	iran	5.5	subcutances three times a weak	high	medium	hard	-0.787756632
267	12	0	other	2.5	subcutances other days	medium	low	hard	-0.352164436
268	1	0	iran	5.5	mascular once a weak	high	medium	hard	-0.572088286
268	1	1	iran	0.5	subcutances three times a weak	high	medium	easy	-0.72867963
268	2	1	other	0.5	subcutances other days	high	medium	hard	-0.643869086
268	2	0	iran	5.5	subcutances other days	medium	medium	hard	-0.928090082
268	3	0	iran	5.5	mascular once a weak	high	medium	hard	-0.572088286
268	3	1	iran	2.5	subcutances three times a weak	high	medium	easy	-0.646894171
268	4	0	iran	0.5	subcutances three times a weak	medium	low	hard	-0.699689278
268	4	1	other	2.5	mascular once a weak	high	low	hard	0.00383736
268	5	0	iran	0.5	mascular once a weak	medium	low	easy	-0.303092094
268	5	1	other	2.5	subcutances three times a weak	high	low	easy	-0.030902146
268	6	0	other	2.5	subcutances three times a weak	high	low	easy	-0.030902146
268	6	1	other	0.5	subcutances other days	high	medium	easy	-0.462940247
268	7	0	other	0.5	mascular once a weak	medium	medium	hard	-0.900801637
268	7	1	other	5.5	subcutances other days	medium	low	hard	-0.312098058
268	8	1	iran	2.5	mascular once a weak	high	low	hard	-0.0957683
268	8	0	iran	5.5	subcutances three times a weak	high	low	easy	-0.090441429

268	9	0	iran	2.5	subcutances three times a weak	medium	low	hard	-0.617903819
268	9	1	other	0.5	subcutances other days	high	medium	hard	-0.643869086
268	10	1	other	2.5	mascular once a weak	high	low	hard	0.00383736
268	10	0	iran	5.5	mascular once a weak	medium	medium	easy	-0.697626621
268	11	1	other	5.5	subcutances other days	high	medium	easy	-0.34108841
268	11	0	iran	0.5	subcutances three times a weak	medium	low	hard	-0.699689278
268	12	1	iran	5.5	subcutances three times a weak	high	medium	hard	-0.787756632
268	12	0	other	2.5	subcutances other days	medium	low	hard	-0.352164436
269	1	0	iran	5.5	mascular once a weak	high	medium	hard	-0.572088286
269	1	1	iran	0.5	subcutances three times a weak	high	medium	easy	-0.72867963
269	2	1	other	0.5	subcutances other days	high	medium	hard	-0.643869086
269	2	0	iran	5.5	subcutances other days	medium	medium	hard	-0.928090082
269	3	0	iran	5.5	mascular once a weak	high	medium	hard	-0.572088286
269	3	1	iran	2.5	subcutances three times a weak	high	medium	easy	-0.646894171
269	4	0	iran	0.5	subcutances three times a weak	medium	low	hard	-0.699689278
269	4	1	other	2.5	mascular once a weak	high	low	hard	0.00383736
269	5	0	iran	0.5	mascular once a weak	medium	low	easy	-0.303092094
269	5	1	other	2.5	subcutances three times a weak	high	low	easy	-0.030902146
269	6	1	other	2.5	subcutances three times a weak	high	low	easy	-0.030902146
269	6	0	other	0.5	subcutances other days	high	medium	easy	-0.462940247
269	7	0	other	0.5	mascular once a weak	medium	medium	hard	-0.900801637
269	7	1	other	5.5	subcutances other days	medium	low	hard	-0.312098058
269	8	0	iran	2.5	mascular once a weak	high	low	hard	-0.0957683
269	8	1	iran	5.5	subcutances three times a weak	high	low	easy	-0.090441429
269	9	1	iran	2.5	subcutances three times a weak	medium	low	hard	-0.617903819
269	9	0	other	0.5	subcutances other days	high	medium	hard	-0.643869086
269	10	1	other	2.5	mascular once a weak	high	low	hard	0.00383736
269	10	0	iran	5.5	mascular once a weak	medium	medium	easy	-0.697626621
269	11	0	other	5.5	subcutances other days	high	medium	easy	-0.34108841
269	11	1	iran	0.5	subcutances three times a weak	medium	low	hard	-0.699689278
269	12	0	iran	5.5	subcutances three times a weak	high	medium	hard	-0.787756632
269	12	1	other	2.5	subcutances other days	medium	low	hard	-0.352164436
270	1	0	iran	5.5	mascular once a weak	high	medium	hard	-0.572088286
270	1	1	iran	0.5	subcutances three times a weak	high	medium	easy	-0.72867963

270	2	0	other	0.5	subcutances other days	high	medium	hard	-0.643869086
270	2	1	iran	5.5	subcutances other days	medium	medium	hard	-0.928090082
270	3	0	iran	5.5	mascular once a weak	high	medium	hard	-0.572088286
270	3	1	iran	2.5	subcutances three times a weak	high	medium	easy	-0.646894171
270	4	0	iran	0.5	subcutances three times a weak	medium	low	hard	-0.699689278
270	4	1	other	2.5	mascular once a weak	high	low	hard	0.00383736
270	5	1	iran	0.5	mascular once a weak	medium	low	easy	-0.303092094
270	5	0	other	2.5	subcutances three times a weak	high	low	easy	-0.030902146
270	6	1	other	2.5	subcutances three times a weak	high	low	easy	-0.030902146
270	6	0	other	0.5	subcutances other days	high	medium	easy	-0.462940247
270	7	0	other	0.5	mascular once a weak	medium	medium	hard	-0.900801637
270	7	1	other	5.5	subcutances other days	medium	low	hard	-0.312098058
270	8	1	iran	2.5	mascular once a weak	high	low	hard	-0.0957683
270	8	0	iran	5.5	subcutances three times a weak	high	low	easy	-0.090441429
270	9	1	iran	2.5	subcutances three times a weak	medium	low	hard	-0.617903819
270	9	0	other	0.5	subcutances other days	high	medium	hard	-0.643869086
270	10	0	other	2.5	mascular once a weak	high	low	hard	0.00383736
270	10	1	iran	5.5	mascular once a weak	medium	medium	easy	-0.697626621
270	11	0	other	5.5	subcutances other days	high	medium	easy	-0.34108841
270	11	1	iran	0.5	subcutances three times a weak	medium	low	hard	-0.699689278
270	12	0	iran	5.5	subcutances three times a weak	high	medium	hard	-0.787756632
270	12	1	other	2.5	subcutances other days	medium	low	hard	-0.352164436
271	1	0	iran	5.5	mascular once a weak	high	medium	hard	-0.572088286
271	1	1	iran	0.5	subcutances three times a weak	high	medium	easy	-0.72867963
271	2	1	other	0.5	subcutances other days	high	medium	hard	-0.643869086
271	2	0	iran	5.5	subcutances other days	medium	medium	hard	-0.928090082
271	3	0	iran	5.5	mascular once a weak	high	medium	hard	-0.572088286
271	3	1	iran	2.5	subcutances three times a weak	high	medium	easy	-0.646894171
271	4	0	iran	0.5	subcutances three times a weak	medium	low	hard	-0.699689278
271	4	1	other	2.5	mascular once a weak	high	low	hard	0.00383736
271	5	0	iran	0.5	mascular once a weak	medium	low	easy	-0.303092094
271	5	1	other	2.5	subcutances three times a weak	high	low	easy	-0.030902146
271	6	1	other	2.5	subcutances three times a weak	high	low	easy	-0.030902146
271	6	0	other	0.5	subcutances other days	high	medium	easy	-0.462940247

271	7	0	other	0.5	mascular once a weak	medium	medium	hard	-0.900801637
271	7	1	other	5.5	subcutances other days	medium	low	hard	-0.312098058
271	8	0	iran	2.5	mascular once a weak	high	low	hard	-0.0957683
271	8	1	iran	5.5	subcutances three times a weak	high	low	easy	-0.090441429
271	9	0	iran	2.5	subcutances three times a weak	medium	low	hard	-0.617903819
271	9	1	other	0.5	subcutances other days	high	medium	hard	-0.643869086
271	10	1	other	2.5	mascular once a weak	high	low	hard	0.00383736
271	10	0	iran	5.5	mascular once a weak	medium	medium	easy	-0.697626621
271	11	1	other	5.5	subcutances other days	high	medium	easy	-0.34108841
271	11	0	iran	0.5	subcutances three times a weak	medium	low	hard	-0.699689278
271	12	1	iran	5.5	subcutances three times a weak	high	medium	hard	-0.787756632
271	12	0	other	2.5	subcutances other days	medium	low	hard	-0.352164436
272	1	1	iran	5.5	mascular once a weak	high	medium	hard	-0.572088286
272	1	0	iran	0.5	subcutances three times a weak	high	medium	easy	-0.72867963
272	2	0	other	0.5	subcutances other days	high	medium	hard	-0.643869086
272	2	1	iran	5.5	subcutances other days	medium	medium	hard	-0.928090082
272	3	0	iran	5.5	mascular once a weak	high	medium	hard	-0.572088286
272	3	1	iran	2.5	subcutances three times a weak	high	medium	easy	-0.646894171
272	4	1	iran	0.5	subcutances three times a weak	medium	low	hard	-0.699689278
272	4	0	other	2.5	mascular once a weak	high	low	hard	0.00383736
272	5	1	iran	0.5	mascular once a weak	medium	low	easy	-0.303092094
272	5	0	other	2.5	subcutances three times a weak	high	low	easy	-0.030902146
272	6	0	other	2.5	subcutances three times a weak	high	low	easy	-0.030902146
272	6	1	other	0.5	subcutances other days	high	medium	easy	-0.462940247
272	7	0	other	0.5	mascular once a weak	medium	medium	hard	-0.900801637
272	7	1	other	5.5	subcutances other days	medium	low	hard	-0.312098058
272	8	1	iran	2.5	mascular once a weak	high	low	hard	-0.0957683
272	8	0	iran	5.5	subcutances three times a weak	high	low	easy	-0.090441429
272	9	1	iran	2.5	subcutances three times a weak	medium	low	hard	-0.617903819
272	9	0	other	0.5	subcutances other days	high	medium	hard	-0.643869086
272	10	0	other	2.5	mascular once a weak	high	low	hard	0.00383736
272	10	1	iran	5.5	mascular once a weak	medium	medium	easy	-0.697626621
272	11	0	other	5.5	subcutances other days	high	medium	easy	-0.34108841
272	11	1	iran	0.5	subcutances three times a weak	medium	low	hard	-0.699689278

272	12	0	iran	5.5	subcutances three times a weak	high	medium	hard	-0.787756632
272	12	1	other	2.5	subcutances other days	medium	low	hard	-0.352164436
273	1	0	iran	5.5	mascular once a weak	high	medium	hard	-0.572088286
273	1	1	iran	0.5	subcutances three times a weak	high	medium	easy	-0.72867963
273	2	1	other	0.5	subcutances other days	high	medium	hard	-0.643869086
273	2	0	iran	5.5	subcutances other days	medium	medium	hard	-0.928090082
273	3	0	iran	5.5	mascular once a weak	high	medium	hard	-0.572088286
273	3	1	iran	2.5	subcutances three times a weak	high	medium	easy	-0.646894171
273	4	0	iran	0.5	subcutances three times a weak	medium	low	hard	-0.699689278
273	4	1	other	2.5	mascular once a weak	high	low	hard	0.00383736
273	5	0	iran	0.5	mascular once a weak	medium	low	easy	-0.303092094
273	5	1	other	2.5	subcutances three times a weak	high	low	easy	-0.030902146
273	6	1	other	2.5	subcutances three times a weak	high	low	easy	-0.030902146
273	6	0	other	0.5	subcutances other days	high	medium	easy	-0.462940247
273	7	0	other	0.5	mascular once a weak	medium	medium	hard	-0.900801637
273	7	1	other	5.5	subcutances other days	medium	low	hard	-0.312098058
273	8	0	iran	2.5	mascular once a weak	high	low	hard	-0.0957683
273	8	1	iran	5.5	subcutances three times a weak	high	low	easy	-0.090441429
273	9	1	iran	2.5	subcutances three times a weak	medium	low	hard	-0.617903819
273	9	0	other	0.5	subcutances other days	high	medium	hard	-0.643869086
273	10	1	other	2.5	mascular once a weak	high	low	hard	0.00383736
273	10	0	iran	5.5	mascular once a weak	medium	medium	easy	-0.697626621
273	11	1	other	5.5	subcutances other days	high	medium	easy	-0.34108841
273	11	0	iran	0.5	subcutances three times a weak	medium	low	hard	-0.699689278
273	12	0	iran	5.5	subcutances three times a weak	high	medium	hard	-0.787756632
273	12	1	other	2.5	subcutances other days	medium	low	hard	-0.352164436
274	1	1	iran	5.5	mascular once a weak	high	medium	hard	-0.572088286
274	1	0	iran	0.5	subcutances three times a weak	high	medium	easy	-0.72867963
274	2	0	other	0.5	subcutances other days	high	medium	hard	-0.643869086
274	2	1	iran	5.5	subcutances other days	medium	medium	hard	-0.928090082
274	3	0	iran	5.5	mascular once a weak	high	medium	hard	-0.572088286
274	3	1	iran	2.5	subcutances three times a weak	high	medium	easy	-0.646894171
274	4	1	iran	0.5	subcutances three times a weak	medium	low	hard	-0.699689278
274	4	0	other	2.5	mascular once a weak	high	low	hard	0.00383736

274	5	1	iran	0.5	mascular once a weak	medium	low	easy	-0.303092094
274	5	0	other	2.5	subcutances three times a weak	high	low	easy	-0.030902146
274	6	0	other	2.5	subcutances three times a weak	high	low	easy	-0.030902146
274	6	1	other	0.5	subcutances other days	high	medium	easy	-0.462940247
274	7	0	other	0.5	mascular once a weak	medium	medium	hard	-0.900801637
274	7	1	other	5.5	subcutances other days	medium	low	hard	-0.312098058
274	8	1	iran	2.5	mascular once a weak	high	low	hard	-0.0957683
274	8	0	iran	5.5	subcutances three times a weak	high	low	easy	-0.090441429
274	9	1	iran	2.5	subcutances three times a weak	medium	low	hard	-0.617903819
274	9	0	other	0.5	subcutances other days	high	medium	hard	-0.643869086
274	10	0	other	2.5	mascular once a weak	high	low	hard	0.00383736
274	10	1	iran	5.5	mascular once a weak	medium	medium	easy	-0.697626621
274	11	0	other	5.5	subcutances other days	high	medium	easy	-0.34108841
274	11	1	iran	0.5	subcutances three times a weak	medium	low	hard	-0.699689278
274	12	0	iran	5.5	subcutances three times a weak	high	medium	hard	-0.787756632
274	12	1	other	2.5	subcutances other days	medium	low	hard	-0.352164436
275	1	0	iran	5.5	mascular once a weak	high	medium	hard	-0.572088286
275	1	1	iran	0.5	subcutances three times a weak	high	medium	easy	-0.72867963
275	2	0	other	0.5	subcutances other days	high	medium	hard	-0.643869086
275	2	1	iran	5.5	subcutances other days	medium	medium	hard	-0.928090082
275	3	0	iran	5.5	mascular once a weak	high	medium	hard	-0.572088286
275	3	1	iran	2.5	subcutances three times a weak	high	medium	easy	-0.646894171
275	4	1	iran	0.5	subcutances three times a weak	medium	low	hard	-0.699689278
275	4	0	other	2.5	mascular once a weak	high	low	hard	0.00383736
275	5	0	iran	0.5	mascular once a weak	medium	low	easy	-0.303092094
275	5	1	other	2.5	subcutances three times a weak	high	low	easy	-0.030902146
275	6	0	other	2.5	subcutances three times a weak	high	low	easy	-0.030902146
275	6	1	other	0.5	subcutances other days	high	medium	easy	-0.462940247
275	7	0	other	0.5	mascular once a weak	medium	medium	hard	-0.900801637
275	7	1	other	5.5	subcutances other days	medium	low	hard	-0.312098058
275	8	1	iran	2.5	mascular once a weak	high	low	hard	-0.0957683
275	8	0	iran	5.5	subcutances three times a weak	high	low	easy	-0.090441429
275	9	1	iran	2.5	subcutances three times a weak	medium	low	hard	-0.617903819
275	9	0	other	0.5	subcutances other days	high	medium	hard	-0.643869086

275	10	0	other	2.5	mascular once a weak	high	low	hard	0.00383736
275	10	1	iran	5.5	mascular once a weak	medium	medium	easy	-0.697626621
275	11	1	other	5.5	subcutances other days	high	medium	easy	-0.34108841
275	11	0	iran	0.5	subcutances three times a weak	medium	low	hard	-0.699689278
275	12	1	iran	5.5	subcutances three times a weak	high	medium	hard	-0.787756632
275	12	0	other	2.5	subcutances other days	medium	low	hard	-0.352164436
276	1	1	iran	5.5	mascular once a weak	high	medium	hard	-0.572088286
276	1	0	iran	0.5	subcutances three times a weak	high	medium	easy	-0.72867963
276	2	0	other	0.5	subcutances other days	high	medium	hard	-0.643869086
276	2	1	iran	5.5	subcutances other days	medium	medium	hard	-0.928090082
276	3	0	iran	5.5	mascular once a weak	high	medium	hard	-0.572088286
276	3	1	iran	2.5	subcutances three times a weak	high	medium	easy	-0.646894171
276	4	0	iran	0.5	subcutances three times a weak	medium	low	hard	-0.699689278
276	4	1	other	2.5	mascular once a weak	high	low	hard	0.00383736
276	5	0	iran	0.5	mascular once a weak	medium	low	easy	-0.303092094
276	5	1	other	2.5	subcutances three times a weak	high	low	easy	-0.030902146
276	6	0	other	2.5	subcutances three times a weak	high	low	easy	-0.030902146
276	6	1	other	0.5	subcutances other days	high	medium	easy	-0.462940247
276	7	0	other	0.5	mascular once a weak	medium	medium	hard	-0.900801637
276	7	1	other	5.5	subcutances other days	medium	low	hard	-0.312098058
276	8	0	iran	2.5	mascular once a weak	high	low	hard	-0.0957683
276	8	1	iran	5.5	subcutances three times a weak	high	low	easy	-0.090441429
276	9	1	iran	2.5	subcutances three times a weak	medium	low	hard	-0.617903819
276	9	0	other	0.5	subcutances other days	high	medium	hard	-0.643869086
276	10	0	other	2.5	mascular once a weak	high	low	hard	0.00383736
276	10	1	iran	5.5	mascular once a weak	medium	medium	easy	-0.697626621
276	11	0	other	5.5	subcutances other days	high	medium	easy	-0.34108841
276	11	1	iran	0.5	subcutances three times a weak	medium	low	hard	-0.699689278
276	12	0	iran	5.5	subcutances three times a weak	high	medium	hard	-0.787756632
276	12	1	other	2.5	subcutances other days	medium	low	hard	-0.352164436
277	1	1	iran	5.5	mascular once a weak	high	medium	hard	-0.572088286
277	1	0	iran	0.5	subcutances three times a weak	high	medium	easy	-0.72867963
277	2	0	other	0.5	subcutances other days	high	medium	hard	-0.643869086
277	2	1	iran	5.5	subcutances other days	medium	medium	hard	-0.928090082

277	3	0	iran	5.5	mascular once a weak	high	medium	hard	-0.572088286
277	3	1	iran	2.5	subcutances three times a weak	high	medium	easy	-0.646894171
277	4	1	iran	0.5	subcutances three times a weak	medium	low	hard	-0.699689278
277	4	0	other	2.5	mascular once a weak	high	low	hard	0.00383736
277	5	1	iran	0.5	mascular once a weak	medium	low	easy	-0.303092094
277	5	0	other	2.5	subcutances three times a weak	high	low	easy	-0.030902146
277	6	0	other	2.5	subcutances three times a weak	high	low	easy	-0.030902146
277	6	1	other	0.5	subcutances other days	high	medium	easy	-0.462940247
277	7	0	other	0.5	mascular once a weak	medium	medium	hard	-0.900801637
277	7	1	other	5.5	subcutances other days	medium	low	hard	-0.312098058
277	8	1	iran	2.5	mascular once a weak	high	low	hard	-0.0957683
277	8	0	iran	5.5	subcutances three times a weak	high	low	easy	-0.090441429
277	9	1	iran	2.5	subcutances three times a weak	medium	low	hard	-0.617903819
277	9	0	other	0.5	subcutances other days	high	medium	hard	-0.643869086
277	10	0	other	2.5	mascular once a weak	high	low	hard	0.00383736
277	10	1	iran	5.5	mascular once a weak	medium	medium	easy	-0.697626621
277	11	0	other	5.5	subcutances other days	high	medium	easy	-0.34108841
277	11	1	iran	0.5	subcutances three times a weak	medium	low	hard	-0.699689278
277	12	0	iran	5.5	subcutances three times a weak	high	medium	hard	-0.787756632
277	12	1	other	2.5	subcutances other days	medium	low	hard	-0.352164436
278	1	1	iran	5.5	mascular once a weak	high	medium	hard	-0.572088286
278	1	0	iran	0.5	subcutances three times a weak	high	medium	easy	-0.72867963
278	2	1	other	0.5	subcutances other days	high	medium	hard	-0.643869086
278	2	0	iran	5.5	subcutances other days	medium	medium	hard	-0.928090082
278	3	0	iran	5.5	mascular once a weak	high	medium	hard	-0.572088286
278	3	1	iran	2.5	subcutances three times a weak	high	medium	easy	-0.646894171
278	4	0	iran	0.5	subcutances three times a weak	medium	low	hard	-0.699689278
278	4	1	other	2.5	mascular once a weak	high	low	hard	0.00383736
278	5	0	iran	0.5	mascular once a weak	medium	low	easy	-0.303092094
278	5	1	other	2.5	subcutances three times a weak	high	low	easy	-0.030902146
278	6	1	other	2.5	subcutances three times a weak	high	low	easy	-0.030902146
278	6	0	other	0.5	subcutances other days	high	medium	easy	-0.462940247
278	7	0	other	0.5	mascular once a weak	medium	medium	hard	-0.900801637
278	7	1	other	5.5	subcutances other days	medium	low	hard	-0.312098058

278	8	0	iran	2.5	mascular once a weak	high	low	hard	-0.0957683
278	8	1	iran	5.5	subcutances three times a weak	high	low	easy	-0.090441429
278	9	1	iran	2.5	subcutances three times a weak	medium	low	hard	-0.617903819
278	9	0	other	0.5	subcutances other days	high	medium	hard	-0.643869086
278	10	1	other	2.5	mascular once a weak	high	low	hard	0.00383736
278	10	0	iran	5.5	mascular once a weak	medium	medium	easy	-0.697626621
278	11	0	other	5.5	subcutances other days	high	medium	easy	-0.34108841
278	11	1	iran	0.5	subcutances three times a weak	medium	low	hard	-0.699689278
278	12	1	iran	5.5	subcutances three times a weak	high	medium	hard	-0.787756632
278	12	0	other	2.5	subcutances other days	medium	low	hard	-0.352164436
279	1	0	iran	5.5	mascular once a weak	high	medium	hard	-0.572088286
279	1	1	iran	0.5	subcutances three times a weak	high	medium	easy	-0.72867963
279	2	1	other	0.5	subcutances other days	high	medium	hard	-0.643869086
279	2	0	iran	5.5	subcutances other days	medium	medium	hard	-0.928090082
279	3	0	iran	5.5	mascular once a weak	high	medium	hard	-0.572088286
279	3	1	iran	2.5	subcutances three times a weak	high	medium	easy	-0.646894171
279	4	0	iran	0.5	subcutances three times a weak	medium	low	hard	-0.699689278
279	4	1	other	2.5	mascular once a weak	high	low	hard	0.00383736
279	5	0	iran	0.5	mascular once a weak	medium	low	easy	-0.303092094
279	5	1	other	2.5	subcutances three times a weak	high	low	easy	-0.030902146
279	6	1	other	2.5	subcutances three times a weak	high	low	easy	-0.030902146
279	6	0	other	0.5	subcutances other days	high	medium	easy	-0.462940247
279	7	0	other	0.5	mascular once a weak	medium	medium	hard	-0.900801637
279	7	1	other	5.5	subcutances other days	medium	low	hard	-0.312098058
279	8	0	iran	2.5	mascular once a weak	high	low	hard	-0.0957683
279	8	1	iran	5.5	subcutances three times a weak	high	low	easy	-0.090441429
279	9	0	iran	2.5	subcutances three times a weak	medium	low	hard	-0.617903819
279	9	1	other	0.5	subcutances other days	high	medium	hard	-0.643869086
279	10	0	other	2.5	mascular once a weak	high	low	hard	0.00383736
279	10	1	iran	5.5	mascular once a weak	medium	medium	easy	-0.697626621
279	11	1	other	5.5	subcutances other days	high	medium	easy	-0.34108841
279	11	0	iran	0.5	subcutances three times a weak	medium	low	hard	-0.699689278
279	12	0	iran	5.5	subcutances three times a weak	high	medium	hard	-0.787756632
279	12	1	other	2.5	subcutances other days	medium	low	hard	-0.352164436

280	1	1	iran	5.5	mascular once a weak	high	medium	hard	-0.572088286
280	1	0	iran	0.5	subcutances three times a weak	high	medium	easy	-0.72867963
280	2	0	other	0.5	subcutances other days	high	medium	hard	-0.643869086
280	2	1	iran	5.5	subcutances other days	medium	medium	hard	-0.928090082
280	3	0	iran	5.5	mascular once a weak	high	medium	hard	-0.572088286
280	3	1	iran	2.5	subcutances three times a weak	high	medium	easy	-0.646894171
280	4	1	iran	0.5	subcutances three times a weak	medium	low	hard	-0.699689278
280	4	0	other	2.5	mascular once a weak	high	low	hard	0.00383736
280	5	1	iran	0.5	mascular once a weak	medium	low	easy	-0.303092094
280	5	0	other	2.5	subcutances three times a weak	high	low	easy	-0.030902146
280	6	0	other	2.5	subcutances three times a weak	high	low	easy	-0.030902146
280	6	1	other	0.5	subcutances other days	high	medium	easy	-0.462940247
280	7	0	other	0.5	mascular once a weak	medium	medium	hard	-0.900801637
280	7	0	other	5.5	subcutances other days	medium	low	hard	-0.312098058
280	8	1	iran	2.5	mascular once a weak	high	low	hard	-0.0957683
280	8	0	iran	5.5	subcutances three times a weak	high	low	easy	-0.090441429
280	9	1	iran	2.5	subcutances three times a weak	medium	low	hard	-0.617903819
280	9	0	other	0.5	subcutances other days	high	medium	hard	-0.643869086
280	10	0	other	2.5	mascular once a weak	high	low	hard	0.00383736
280	10	1	iran	5.5	mascular once a weak	medium	medium	easy	-0.697626621
280	11	0	other	5.5	subcutances other days	high	medium	easy	-0.34108841
280	11	1	iran	0.5	subcutances three times a weak	medium	low	hard	-0.699689278
280	12	0	iran	5.5	subcutances three times a weak	high	medium	hard	-0.787756632
280	12	1	other	2.5	subcutances other days	medium	low	hard	-0.352164436
281	1	0	iran	5.5	mascular once a weak	high	medium	hard	-0.572088286
281	1	1	iran	0.5	subcutances three times a weak	high	medium	easy	-0.72867963
281	2	1	other	0.5	subcutances other days	high	medium	hard	-0.643869086
281	2	0	iran	5.5	subcutances other days	medium	medium	hard	-0.928090082
281	3	0	iran	5.5	mascular once a weak	high	medium	hard	-0.572088286
281	3	1	iran	2.5	subcutances three times a weak	high	medium	easy	-0.646894171
281	4	0	iran	0.5	subcutances three times a weak	medium	low	hard	-0.699689278
281	4	1	other	2.5	mascular once a weak	high	low	hard	0.00383736
281	5	0	iran	0.5	mascular once a weak	medium	low	easy	-0.303092094
281	5	1	other	2.5	subcutances three times a weak	high	low	easy	-0.030902146

281	6	1	other	2.5	subcutances three times a weak	high	low	easy	-0.030902146
281	6	0	other	0.5	subcutances other days	high	medium	easy	-0.462940247
281	7	0	other	0.5	mascular once a weak	medium	medium	hard	-0.900801637
281	7	1	other	5.5	subcutances other days	medium	low	hard	-0.312098058
281	8	0	iran	2.5	mascular once a weak	high	low	hard	-0.0957683
281	8	1	iran	5.5	subcutances three times a weak	high	low	easy	-0.090441429
281	9	0	iran	2.5	subcutances three times a weak	medium	low	hard	-0.617903819
281	9	1	other	0.5	subcutances other days	high	medium	hard	-0.643869086
281	10	1	other	2.5	mascular once a weak	high	low	hard	0.00383736
281	10	0	iran	5.5	mascular once a weak	medium	medium	easy	-0.697626621
281	11	1	other	5.5	subcutances other days	high	medium	easy	-0.34108841
281	11	0	iran	0.5	subcutances three times a weak	medium	low	hard	-0.699689278
281	12	1	iran	5.5	subcutances three times a weak	high	medium	hard	-0.787756632
281	12	0	other	2.5	subcutances other days	medium	low	hard	-0.352164436
282	1	0	iran	5.5	mascular once a weak	high	medium	hard	-0.572088286
282	1	1	iran	0.5	subcutances three times a weak	high	medium	easy	-0.72867963
282	2	1	other	0.5	subcutances other days	high	medium	hard	-0.643869086
282	2	0	iran	5.5	subcutances other days	medium	medium	hard	-0.928090082
282	3	0	iran	5.5	mascular once a weak	high	medium	hard	-0.572088286
282	3	1	iran	2.5	subcutances three times a weak	high	medium	easy	-0.646894171
282	4	0	iran	0.5	subcutances three times a weak	medium	low	hard	-0.699689278
282	4	1	other	2.5	mascular once a weak	high	low	hard	0.00383736
282	5	0	iran	0.5	mascular once a weak	medium	low	easy	-0.303092094
282	5	1	other	2.5	subcutances three times a weak	high	low	easy	-0.030902146
282	6	1	other	2.5	subcutances three times a weak	high	low	easy	-0.030902146
282	6	0	other	0.5	subcutances other days	high	medium	easy	-0.462940247
282	7	0	other	0.5	mascular once a weak	medium	medium	hard	-0.900801637
282	7	1	other	5.5	subcutances other days	medium	low	hard	-0.312098058
282	8	0	iran	2.5	mascular once a weak	high	low	hard	-0.0957683
282	8	1	iran	5.5	subcutances three times a weak	high	low	easy	-0.090441429
282	9	0	iran	2.5	subcutances three times a weak	medium	low	hard	-0.617903819
282	9	1	other	0.5	subcutances other days	high	medium	hard	-0.643869086
282	10	1	other	2.5	mascular once a weak	high	low	hard	0.00383736
282	10	0	iran	5.5	mascular once a weak	medium	medium	easy	-0.697626621

282	11	1	other	5.5	subcutances other days	high	medium	easy	-0.34108841
282	11	0	iran	0.5	subcutances three times a weak	medium	low	hard	-0.699689278
282	12	1	iran	5.5	subcutances three times a weak	high	medium	hard	-0.787756632
282	12	0	other	2.5	subcutances other days	medium	low	hard	-0.352164436
283	1	1	iran	5.5	mascular once a weak	high	medium	hard	-0.572088286
283	1	0	iran	0.5	subcutances three times a weak	high	medium	easy	-0.72867963
283	2	0	other	0.5	subcutances other days	high	medium	hard	-0.643869086
283	2	1	iran	5.5	subcutances other days	medium	medium	hard	-0.928090082
283	3	1	iran	5.5	mascular once a weak	high	medium	hard	-0.572088286
283	3	0	iran	2.5	subcutances three times a weak	high	medium	easy	-0.646894171
283	4	0	iran	0.5	subcutances three times a weak	medium	low	hard	-0.699689278
283	4	1	other	2.5	mascular once a weak	high	low	hard	0.00383736
283	5	1	iran	0.5	mascular once a weak	medium	low	easy	-0.303092094
283	5	0	other	2.5	subcutances three times a weak	high	low	easy	-0.030902146
283	6	1	other	2.5	subcutances three times a weak	high	low	easy	-0.030902146
283	6	0	other	0.5	subcutances other days	high	medium	easy	-0.462940247
283	7	0	other	0.5	mascular once a weak	medium	medium	hard	-0.900801637
283	7	1	other	5.5	subcutances other days	medium	low	hard	-0.312098058
283	8	1	iran	2.5	mascular once a weak	high	low	hard	-0.0957683
283	8	0	iran	5.5	subcutances three times a weak	high	low	easy	-0.090441429
283	9	0	iran	2.5	subcutances three times a weak	medium	low	hard	-0.617903819
283	9	1	other	0.5	subcutances other days	high	medium	hard	-0.643869086
283	10	1	other	2.5	mascular once a weak	high	low	hard	0.00383736
283	10	0	iran	5.5	mascular once a weak	medium	medium	easy	-0.697626621
283	11	0	other	5.5	subcutances other days	high	medium	easy	-0.34108841
283	11	1	iran	0.5	subcutances three times a weak	medium	low	hard	-0.699689278
283	12	1	iran	5.5	subcutances three times a weak	high	medium	hard	-0.787756632
283	12	0	other	2.5	subcutances other days	medium	low	hard	-0.352164436
284	1	0	iran	5.5	mascular once a weak	high	medium	hard	-0.572088286
284	1	1	iran	0.5	subcutances three times a weak	high	medium	easy	-0.72867963
284	2	1	other	0.5	subcutances other days	high	medium	hard	-0.643869086
284	2	0	iran	5.5	subcutances other days	medium	medium	hard	-0.928090082
284	3	0	iran	5.5	mascular once a weak	high	medium	hard	-0.572088286
284	3	1	iran	2.5	subcutances three times a weak	high	medium	easy	-0.646894171

284	4	0	iran	0.5	subcutances three times a weak	medium	low	hard	-0.699689278
284	4	1	other	2.5	mascular once a weak	high	low	hard	0.00383736
284	5	0	iran	0.5	mascular once a weak	medium	low	easy	-0.303092094
284	5	1	other	2.5	subcutances three times a weak	high	low	easy	-0.030902146
284	6	1	other	2.5	subcutances three times a weak	high	low	easy	-0.030902146
284	6	0	other	0.5	subcutances other days	high	medium	easy	-0.462940247
284	7	0	other	0.5	mascular once a weak	medium	medium	hard	-0.900801637
284	7	1	other	5.5	subcutances other days	medium	low	hard	-0.312098058
284	8	0	iran	2.5	mascular once a weak	high	low	hard	-0.0957683
284	8	1	iran	5.5	subcutances three times a weak	high	low	easy	-0.090441429
284	9	0	iran	2.5	subcutances three times a weak	medium	low	hard	-0.617903819
284	9	1	other	0.5	subcutances other days	high	medium	hard	-0.643869086
284	10	1	other	2.5	mascular once a weak	high	low	hard	0.00383736
284	10	0	iran	5.5	mascular once a weak	medium	medium	easy	-0.697626621
284	11	1	other	5.5	subcutances other days	high	medium	easy	-0.34108841
284	11	0	iran	0.5	subcutances three times a weak	medium	low	hard	-0.699689278
284	12	1	iran	5.5	subcutances three times a weak	high	medium	hard	-0.787756632
284	12	0	other	2.5	subcutances other days	medium	low	hard	-0.352164436
285	1	0	iran	5.5	mascular once a weak	high	medium	hard	-0.572088286
285	1	1	iran	0.5	subcutances three times a weak	high	medium	easy	-0.72867963
285	2	1	other	0.5	subcutances other days	high	medium	hard	-0.643869086
285	2	0	iran	5.5	subcutances other days	medium	medium	hard	-0.928090082
285	3	0	iran	5.5	mascular once a weak	high	medium	hard	-0.572088286
285	3	1	iran	2.5	subcutances three times a weak	high	medium	easy	-0.646894171
285	4	0	iran	0.5	subcutances three times a weak	medium	low	hard	-0.699689278
285	4	1	other	2.5	mascular once a weak	high	low	hard	0.00383736
285	5	0	iran	0.5	mascular once a weak	medium	low	easy	-0.303092094
285	5	1	other	2.5	subcutances three times a weak	high	low	easy	-0.030902146
285	6	1	other	2.5	subcutances three times a weak	high	low	easy	-0.030902146
285	6	0	other	0.5	subcutances other days	high	medium	easy	-0.462940247
285	7	0	other	0.5	mascular once a weak	medium	medium	hard	-0.900801637
285	7	1	other	5.5	subcutances other days	medium	low	hard	-0.312098058
285	8	0	iran	2.5	mascular once a weak	high	low	hard	-0.0957683
285	8	1	iran	5.5	subcutances three times a weak	high	low	easy	-0.090441429

285	9	0	iran	2.5	subcutances three times a weak	medium	low	hard	-0.617903819
285	9	1	other	0.5	subcutances other days	high	medium	hard	-0.643869086
285	10	1	other	2.5	mascular once a weak	high	low	hard	0.00383736
285	10	0	iran	5.5	mascular once a weak	medium	medium	easy	-0.697626621
285	11	1	other	5.5	subcutances other days	high	medium	easy	-0.34108841
285	11	0	iran	0.5	subcutances three times a weak	medium	low	hard	-0.699689278
285	12	1	iran	5.5	subcutances three times a weak	high	medium	hard	-0.787756632
285	12	0	other	2.5	subcutances other days	medium	low	hard	-0.352164436
286	1	1	iran	5.5	mascular once a weak	high	medium	hard	-0.572088286
286	1	0	iran	0.5	subcutances three times a weak	high	medium	easy	-0.72867963
286	2	1	other	0.5	subcutances other days	high	medium	hard	-0.643869086
286	2	0	iran	5.5	subcutances other days	medium	medium	hard	-0.928090082
286	3	0	iran	5.5	mascular once a weak	high	medium	hard	-0.572088286
286	3	1	iran	2.5	subcutances three times a weak	high	medium	easy	-0.646894171
286	4	0	iran	0.5	subcutances three times a weak	medium	low	hard	-0.699689278
286	4	1	other	2.5	mascular once a weak	high	low	hard	0.00383736
286	5	0	iran	0.5	mascular once a weak	medium	low	easy	-0.303092094
286	5	1	other	2.5	subcutances three times a weak	high	low	easy	-0.030902146
286	6	0	other	2.5	subcutances three times a weak	high	low	easy	-0.030902146
286	6	1	other	0.5	subcutances other days	high	medium	easy	-0.462940247
286	7	1	other	0.5	mascular once a weak	medium	medium	hard	-0.900801637
286	7	0	other	5.5	subcutances other days	medium	low	hard	-0.312098058
286	8	1	iran	2.5	mascular once a weak	high	low	hard	-0.0957683
286	8	0	iran	5.5	subcutances three times a weak	high	low	easy	-0.090441429
286	9	0	iran	2.5	subcutances three times a weak	medium	low	hard	-0.617903819
286	9	1	other	0.5	subcutances other days	high	medium	hard	-0.643869086
286	10	1	other	2.5	mascular once a weak	high	low	hard	0.00383736
286	10	0	iran	5.5	mascular once a weak	medium	medium	easy	-0.697626621
286	11	0	other	5.5	subcutances other days	high	medium	easy	-0.34108841
286	11	1	iran	0.5	subcutances three times a weak	medium	low	hard	-0.699689278
286	12	0	iran	5.5	subcutances three times a weak	high	medium	hard	-0.787756632
286	12	1	other	2.5	subcutances other days	medium	low	hard	-0.352164436
287	1	1	iran	5.5	mascular once a weak	high	medium	hard	-0.572088286
287	1	0	iran	0.5	subcutances three times a weak	high	medium	easy	-0.72867963

287	2	0	other	0.5	subcutances other days	high	medium	hard	-0.643869086
287	2	1	iran	5.5	subcutances other days	medium	medium	hard	-0.928090082
287	3	1	iran	5.5	mascular once a weak	high	medium	hard	-0.572088286
287	3	0	iran	2.5	subcutances three times a weak	high	medium	easy	-0.646894171
287	4	0	iran	0.5	subcutances three times a weak	medium	low	hard	-0.699689278
287	4	1	other	2.5	mascular once a weak	high	low	hard	0.00383736
287	5	1	iran	0.5	mascular once a weak	medium	low	easy	-0.303092094
287	5	0	other	2.5	subcutances three times a weak	high	low	easy	-0.030902146
287	6	0	other	2.5	subcutances three times a weak	high	low	easy	-0.030902146
287	6	1	other	0.5	subcutances other days	high	medium	easy	-0.462940247
287	7	1	other	0.5	mascular once a weak	medium	medium	hard	-0.900801637
287	7	0	other	5.5	subcutances other days	medium	low	hard	-0.312098058
287	8	1	iran	2.5	mascular once a weak	high	low	hard	-0.0957683
287	8	0	iran	5.5	subcutances three times a weak	high	low	easy	-0.090441429
287	9	1	iran	2.5	subcutances three times a weak	medium	low	hard	-0.617903819
287	9	0	other	0.5	subcutances other days	high	medium	hard	-0.643869086
287	10	0	other	2.5	mascular once a weak	high	low	hard	0.00383736
287	10	1	iran	5.5	mascular once a weak	medium	medium	easy	-0.697626621
287	11	1	other	5.5	subcutances other days	high	medium	easy	-0.34108841
287	11	0	iran	0.5	subcutances three times a weak	medium	low	hard	-0.699689278
287	12	0	iran	5.5	subcutances three times a weak	high	medium	hard	-0.787756632
287	12	1	other	2.5	subcutances other days	medium	low	hard	-0.352164436
288	1	1	iran	5.5	mascular once a weak	high	medium	hard	-0.572088286
288	1	0	iran	0.5	subcutances three times a weak	high	medium	easy	-0.72867963
288	2	0	other	0.5	subcutances other days	high	medium	hard	-0.643869086
288	2	1	iran	5.5	subcutances other days	medium	medium	hard	-0.928090082
288	3	1	iran	5.5	mascular once a weak	high	medium	hard	-0.572088286
288	3	0	iran	2.5	subcutances three times a weak	high	medium	easy	-0.646894171
288	4	0	iran	0.5	subcutances three times a weak	medium	low	hard	-0.699689278
288	4	1	other	2.5	mascular once a weak	high	low	hard	0.00383736
288	5	1	iran	0.5	mascular once a weak	medium	low	easy	-0.303092094
288	5	0	other	2.5	subcutances three times a weak	high	low	easy	-0.030902146
288	6	0	other	2.5	subcutances three times a weak	high	low	easy	-0.030902146
288	6	1	other	0.5	subcutances other days	high	medium	easy	-0.462940247

288	7	1	other	0.5	mascular once a weak	medium	medium	hard	-0.900801637
288	7	0	other	5.5	subcutances other days	medium	low	hard	-0.312098058
288	8	1	iran	2.5	mascular once a weak	high	low	hard	-0.0957683
288	8	0	iran	5.5	subcutances three times a weak	high	low	easy	-0.090441429
288	9	1	iran	2.5	subcutances three times a weak	medium	low	hard	-0.617903819
288	9	0	other	0.5	subcutances other days	high	medium	hard	-0.643869086
288	10	0	other	2.5	mascular once a weak	high	low	hard	0.00383736
288	10	1	iran	5.5	mascular once a weak	medium	medium	easy	-0.697626621
288	11	0	other	5.5	subcutances other days	high	medium	easy	-0.34108841
288	11	1	iran	0.5	subcutances three times a weak	medium	low	hard	-0.699689278
288	12	0	iran	5.5	subcutances three times a weak	high	medium	hard	-0.787756632
288	12	1	other	2.5	subcutances other days	medium	low	hard	-0.352164436
289	1	0	iran	5.5	mascular once a weak	high	medium	hard	-0.572088286
289	1	1	iran	0.5	subcutances three times a weak	high	medium	easy	-0.72867963
289	2	0	other	0.5	subcutances other days	high	medium	hard	-0.643869086
289	2	1	iran	5.5	subcutances other days	medium	medium	hard	-0.928090082
289	3	0	iran	5.5	mascular once a weak	high	medium	hard	-0.572088286
289	3	1	iran	2.5	subcutances three times a weak	high	medium	easy	-0.646894171
289	4	0	iran	0.5	subcutances three times a weak	medium	low	hard	-0.699689278
289	4	1	other	2.5	mascular once a weak	high	low	hard	0.00383736
289	5	1	iran	0.5	mascular once a weak	medium	low	easy	-0.303092094
289	5	0	other	2.5	subcutances three times a weak	high	low	easy	-0.030902146
289	6	1	other	2.5	subcutances three times a weak	high	low	easy	-0.030902146
289	6	0	other	0.5	subcutances other days	high	medium	easy	-0.462940247
289	7	0	other	0.5	mascular once a weak	medium	medium	hard	-0.900801637
289	7	1	other	5.5	subcutances other days	medium	low	hard	-0.312098058
289	8	1	iran	2.5	mascular once a weak	high	low	hard	-0.0957683
289	8	0	iran	5.5	subcutances three times a weak	high	low	easy	-0.090441429
289	9	1	iran	2.5	subcutances three times a weak	medium	low	hard	-0.617903819
289	9	0	other	0.5	subcutances other days	high	medium	hard	-0.643869086
289	10	1	other	2.5	mascular once a weak	high	low	hard	0.00383736
289	10	0	iran	5.5	mascular once a weak	medium	medium	easy	-0.697626621
289	11	1	other	5.5	subcutances other days	high	medium	easy	-0.34108841
289	11	0	iran	0.5	subcutances three times a weak	medium	low	hard	-0.699689278

289	12	0	iran	5.5	subcutances three times a weak	high	medium	hard	-0.787756632
289	12	1	other	2.5	subcutances other days	medium	low	hard	-0.352164436
290	1	1	iran	5.5	mascular once a weak	high	medium	hard	-0.572088286
290	1	0	iran	0.5	subcutances three times a weak	high	medium	easy	-0.72867963
290	2	0	other	0.5	subcutances other days	high	medium	hard	-0.643869086
290	2	1	iran	5.5	subcutances other days	medium	medium	hard	-0.928090082
290	3	0	iran	5.5	mascular once a weak	high	medium	hard	-0.572088286
290	3	1	iran	2.5	subcutances three times a weak	high	medium	easy	-0.646894171
290	4	1	iran	0.5	subcutances three times a weak	medium	low	hard	-0.699689278
290	4	0	other	2.5	mascular once a weak	high	low	hard	0.00383736
290	5	1	iran	0.5	mascular once a weak	medium	low	easy	-0.303092094
290	5	0	other	2.5	subcutances three times a weak	high	low	easy	-0.030902146
290	6	0	other	2.5	subcutances three times a weak	high	low	easy	-0.030902146
290	6	1	other	0.5	subcutances other days	high	medium	easy	-0.462940247
290	7	1	other	0.5	mascular once a weak	medium	medium	hard	-0.900801637
290	7	0	other	5.5	subcutances other days	medium	low	hard	-0.312098058
290	8	1	iran	2.5	mascular once a weak	high	low	hard	-0.0957683
290	8	0	iran	5.5	subcutances three times a weak	high	low	easy	-0.090441429
290	9	1	iran	2.5	subcutances three times a weak	medium	low	hard	-0.617903819
290	9	0	other	0.5	subcutances other days	high	medium	hard	-0.643869086
290	10	0	other	2.5	mascular once a weak	high	low	hard	0.00383736
290	10	1	iran	5.5	mascular once a weak	medium	medium	easy	-0.697626621
290	11	0	other	5.5	subcutances other days	high	medium	easy	-0.34108841
290	11	1	iran	0.5	subcutances three times a weak	medium	low	hard	-0.699689278
290	12	0	iran	5.5	subcutances three times a weak	high	medium	hard	-0.787756632
290	12	1	other	2.5	subcutances other days	medium	low	hard	-0.352164436
291	1	1	iran	5.5	mascular once a weak	high	medium	hard	-0.572088286
291	1	0	iran	0.5	subcutances three times a weak	high	medium	easy	-0.72867963
291	2	0	other	0.5	subcutances other days	high	medium	hard	-0.643869086
291	2	1	iran	5.5	subcutances other days	medium	medium	hard	-0.928090082
291	3	0	iran	5.5	mascular once a weak	high	medium	hard	-0.572088286
291	3	1	iran	2.5	subcutances three times a weak	high	medium	easy	-0.646894171
291	4	1	iran	0.5	subcutances three times a weak	medium	low	hard	-0.699689278
291	4	0	other	2.5	mascular once a weak	high	low	hard	0.00383736

291	5	1	iran	0.5	mascular once a weak	medium	low	easy	-0.303092094
291	5	0	other	2.5	subcutances three times a weak	high	low	easy	-0.030902146
291	6	0	other	2.5	subcutances three times a weak	high	low	easy	-0.030902146
291	6	1	other	0.5	subcutances other days	high	medium	easy	-0.462940247
291	7	1	other	0.5	mascular once a weak	medium	medium	hard	-0.900801637
291	7	0	other	5.5	subcutances other days	medium	low	hard	-0.312098058
291	8	1	iran	2.5	mascular once a weak	high	low	hard	-0.0957683
291	8	0	iran	5.5	subcutances three times a weak	high	low	easy	-0.090441429
291	9	1	iran	2.5	subcutances three times a weak	medium	low	hard	-0.617903819
291	9	0	other	0.5	subcutances other days	high	medium	hard	-0.643869086
291	10	0	other	2.5	mascular once a weak	high	low	hard	0.00383736
291	10	1	iran	5.5	mascular once a weak	medium	medium	easy	-0.697626621
291	11	0	other	5.5	subcutances other days	high	medium	easy	-0.34108841
291	11	1	iran	0.5	subcutances three times a weak	medium	low	hard	-0.699689278
291	12	0	iran	5.5	subcutances three times a weak	high	medium	hard	-0.787756632
291	12	1	other	2.5	subcutances other days	medium	low	hard	-0.352164436
292	1	1	iran	5.5	mascular once a weak	high	medium	hard	-0.572088286
292	1	0	iran	0.5	subcutances three times a weak	high	medium	easy	-0.72867963
292	2	1	other	0.5	subcutances other days	high	medium	hard	-0.643869086
292	2	0	iran	5.5	subcutances other days	medium	medium	hard	-0.928090082
292	3	0	iran	5.5	mascular once a weak	high	medium	hard	-0.572088286
292	3	1	iran	2.5	subcutances three times a weak	high	medium	easy	-0.646894171
292	4	0	iran	0.5	subcutances three times a weak	medium	low	hard	-0.699689278
292	4	1	other	2.5	mascular once a weak	high	low	hard	0.00383736
292	5	0	iran	0.5	mascular once a weak	medium	low	easy	-0.303092094
292	5	1	other	2.5	subcutances three times a weak	high	low	easy	-0.030902146
292	6	1	other	2.5	subcutances three times a weak	high	low	easy	-0.030902146
292	6	0	other	0.5	subcutances other days	high	medium	easy	-0.462940247
292	7	0	other	0.5	mascular once a weak	medium	medium	hard	-0.900801637
292	7	1	other	5.5	subcutances other days	medium	low	hard	-0.312098058
292	8	0	iran	2.5	mascular once a weak	high	low	hard	-0.0957683
292	8	1	iran	5.5	subcutances three times a weak	high	low	easy	-0.090441429
292	9	1	iran	2.5	subcutances three times a weak	medium	low	hard	-0.617903819
292	9	0	other	0.5	subcutances other days	high	medium	hard	-0.643869086

292	10	1	other	2.5	mascular once a weak	high	low	hard	0.00383736
292	10	0	iran	5.5	mascular once a weak	medium	medium	easy	-0.697626621
292	11	0	other	5.5	subcutances other days	high	medium	easy	-0.34108841
292	11	1	iran	0.5	subcutances three times a weak	medium	low	hard	-0.699689278
292	12	0	iran	5.5	subcutances three times a weak	high	medium	hard	-0.787756632
292	12	1	other	2.5	subcutances other days	medium	low	hard	-0.352164436
293	1	1	iran	5.5	mascular once a weak	high	medium	hard	-0.572088286
293	1	0	iran	0.5	subcutances three times a weak	high	medium	easy	-0.72867963
293	2	1	other	0.5	subcutances other days	high	medium	hard	-0.643869086
293	2	0	iran	5.5	subcutances other days	medium	medium	hard	-0.928090082
293	3	0	iran	5.5	mascular once a weak	high	medium	hard	-0.572088286
293	3	1	iran	2.5	subcutances three times a weak	high	medium	easy	-0.646894171
293	4	1	iran	0.5	subcutances three times a weak	medium	low	hard	-0.699689278
293	4	0	other	2.5	mascular once a weak	high	low	hard	0.00383736
293	5	0	iran	0.5	mascular once a weak	medium	low	easy	-0.303092094
293	5	1	other	2.5	subcutances three times a weak	high	low	easy	-0.030902146
293	6	1	other	2.5	subcutances three times a weak	high	low	easy	-0.030902146
293	6	0	other	0.5	subcutances other days	high	medium	easy	-0.462940247
293	7	1	other	0.5	mascular once a weak	medium	medium	hard	-0.900801637
293	7	0	other	5.5	subcutances other days	medium	low	hard	-0.312098058
293	8	0	iran	2.5	mascular once a weak	high	low	hard	-0.0957683
293	8	1	iran	5.5	subcutances three times a weak	high	low	easy	-0.090441429
293	9	1	iran	2.5	subcutances three times a weak	medium	low	hard	-0.617903819
293	9	0	other	0.5	subcutances other days	high	medium	hard	-0.643869086
293	10	1	other	2.5	mascular once a weak	high	low	hard	0.00383736
293	10	0	iran	5.5	mascular once a weak	medium	medium	easy	-0.697626621
293	11	0	other	5.5	subcutances other days	high	medium	easy	-0.34108841
293	11	1	iran	0.5	subcutances three times a weak	medium	low	hard	-0.699689278
293	12	0	iran	5.5	subcutances three times a weak	high	medium	hard	-0.787756632
293	12	1	other	2.5	subcutances other days	medium	low	hard	-0.352164436
294	1	0	iran	5.5	mascular once a weak	high	medium	hard	-0.572088286
294	1	1	iran	0.5	subcutances three times a weak	high	medium	easy	-0.72867963
294	2	1	other	0.5	subcutances other days	high	medium	hard	-0.643869086
294	2	0	iran	5.5	subcutances other days	medium	medium	hard	-0.928090082

294	3	0	iran	5.5	mascular once a weak	high	medium	hard	-0.572088286
294	3	1	iran	2.5	subcutances three times a weak	high	medium	easy	-0.646894171
294	4	0	iran	0.5	subcutances three times a weak	medium	low	hard	-0.699689278
294	4	1	other	2.5	mascular once a weak	high	low	hard	0.00383736
294	5	0	iran	0.5	mascular once a weak	medium	low	easy	-0.303092094
294	5	1	other	2.5	subcutances three times a weak	high	low	easy	-0.030902146
294	6	1	other	2.5	subcutances three times a weak	high	low	easy	-0.030902146
294	6	0	other	0.5	subcutances other days	high	medium	easy	-0.462940247
294	7	0	other	0.5	mascular once a weak	medium	medium	hard	-0.900801637
294	7	1	other	5.5	subcutances other days	medium	low	hard	-0.312098058
294	8	0	iran	2.5	mascular once a weak	high	low	hard	-0.0957683
294	8	1	iran	5.5	subcutances three times a weak	high	low	easy	-0.090441429
294	9	1	iran	2.5	subcutances three times a weak	medium	low	hard	-0.617903819
294	9	0	other	0.5	subcutances other days	high	medium	hard	-0.643869086
294	10	1	other	2.5	mascular once a weak	high	low	hard	0.00383736
294	10	0	iran	5.5	mascular once a weak	medium	medium	easy	-0.697626621
294	11	0	other	5.5	subcutances other days	high	medium	easy	-0.34108841
294	11	1	iran	0.5	subcutances three times a weak	medium	low	hard	-0.699689278
294	12	0	iran	5.5	subcutances three times a weak	high	medium	hard	-0.787756632
294	12	1	other	2.5	subcutances other days	medium	low	hard	-0.352164436
295	1	1	iran	5.5	mascular once a weak	high	medium	hard	-0.572088286
295	1	0	iran	0.5	subcutances three times a weak	high	medium	easy	-0.72867963
295	2	0	other	0.5	subcutances other days	high	medium	hard	-0.643869086
295	2	1	iran	5.5	subcutances other days	medium	medium	hard	-0.928090082
295	3	0	iran	5.5	mascular once a weak	high	medium	hard	-0.572088286
295	3	1	iran	2.5	subcutances three times a weak	high	medium	easy	-0.646894171
295	4	1	iran	0.5	subcutances three times a weak	medium	low	hard	-0.699689278
295	4	0	other	2.5	mascular once a weak	high	low	hard	0.00383736
295	5	1	iran	0.5	mascular once a weak	medium	low	easy	-0.303092094
295	5	0	other	2.5	subcutances three times a weak	high	low	easy	-0.030902146
295	6	0	other	2.5	subcutances three times a weak	high	low	easy	-0.030902146
295	6	1	other	0.5	subcutances other days	high	medium	easy	-0.462940247
295	7	0	other	0.5	mascular once a weak	medium	medium	hard	-0.900801637
295	7	0	other	5.5	subcutances other days	medium	low	hard	-0.312098058

295	8	1	iran	2.5	mascular once a weak	high	low	hard	-0.0957683
295	8	0	iran	5.5	subcutances three times a weak	high	low	easy	-0.090441429
295	9	1	iran	2.5	subcutances three times a weak	medium	low	hard	-0.617903819
295	9	0	other	0.5	subcutances other days	high	medium	hard	-0.643869086
295	10	0	other	2.5	mascular once a weak	high	low	hard	0.00383736
295	10	1	iran	5.5	mascular once a weak	medium	medium	easy	-0.697626621
295	11	0	other	5.5	subcutances other days	high	medium	easy	-0.34108841
295	11	1	iran	0.5	subcutances three times a weak	medium	low	hard	-0.699689278
295	12	0	iran	5.5	subcutances three times a weak	high	medium	hard	-0.787756632
295	12	1	other	2.5	subcutances other days	medium	low	hard	-0.352164436
296	1	1	iran	5.5	mascular once a weak	high	medium	hard	-0.572088286
296	1	0	iran	0.5	subcutances three times a weak	high	medium	easy	-0.72867963
296	2	0	other	0.5	subcutances other days	high	medium	hard	-0.643869086
296	2	1	iran	5.5	subcutances other days	medium	medium	hard	-0.928090082
296	3	0	iran	5.5	mascular once a weak	high	medium	hard	-0.572088286
296	3	1	iran	2.5	subcutances three times a weak	high	medium	easy	-0.646894171
296	4	0	iran	0.5	subcutances three times a weak	medium	low	hard	-0.699689278
296	4	1	other	2.5	mascular once a weak	high	low	hard	0.00383736
296	5	0	iran	0.5	mascular once a weak	medium	low	easy	-0.303092094
296	5	1	other	2.5	subcutances three times a weak	high	low	easy	-0.030902146
296	6	1	other	2.5	subcutances three times a weak	high	low	easy	-0.030902146
296	6	0	other	0.5	subcutances other days	high	medium	easy	-0.462940247
296	7	0	other	0.5	mascular once a weak	medium	medium	hard	-0.900801637
296	7	1	other	5.5	subcutances other days	medium	low	hard	-0.312098058
296	8	0	iran	2.5	mascular once a weak	high	low	hard	-0.0957683
296	8	1	iran	5.5	subcutances three times a weak	high	low	easy	-0.090441429
296	9	0	iran	2.5	subcutances three times a weak	medium	low	hard	-0.617903819
296	9	1	other	0.5	subcutances other days	high	medium	hard	-0.643869086
296	10	0	other	2.5	mascular once a weak	high	low	hard	0.00383736
296	10	1	iran	5.5	mascular once a weak	medium	medium	easy	-0.697626621
296	11	0	other	5.5	subcutances other days	high	medium	easy	-0.34108841
296	11	1	iran	0.5	subcutances three times a weak	medium	low	hard	-0.699689278
296	12	0	iran	5.5	subcutances three times a weak	high	medium	hard	-0.787756632
296	12	1	other	2.5	subcutances other days	medium	low	hard	-0.352164436

297	1	0	iran	5.5	mascular once a weak	high	medium	hard	-0.572088286
297	1	1	iran	0.5	subcutances three times a weak	high	medium	easy	-0.72867963
297	2	1	other	0.5	subcutances other days	high	medium	hard	-0.643869086
297	2	0	iran	5.5	subcutances other days	medium	medium	hard	-0.928090082
297	3	1	iran	5.5	mascular once a weak	high	medium	hard	-0.572088286
297	3	0	iran	2.5	subcutances three times a weak	high	medium	easy	-0.646894171
297	4	0	iran	0.5	subcutances three times a weak	medium	low	hard	-0.699689278
297	4	1	other	2.5	mascular once a weak	high	low	hard	0.00383736
297	5	0	iran	0.5	mascular once a weak	medium	low	easy	-0.303092094
297	5	1	other	2.5	subcutances three times a weak	high	low	easy	-0.030902146
297	6	1	other	2.5	subcutances three times a weak	high	low	easy	-0.030902146
297	6	0	other	0.5	subcutances other days	high	medium	easy	-0.462940247
297	7	0	other	0.5	mascular once a weak	medium	medium	hard	-0.900801637
297	7	1	other	5.5	subcutances other days	medium	low	hard	-0.312098058
297	8	0	iran	2.5	mascular once a weak	high	low	hard	-0.0957683
297	8	1	iran	5.5	subcutances three times a weak	high	low	easy	-0.090441429
297	9	1	iran	2.5	subcutances three times a weak	medium	low	hard	-0.617903819
297	9	0	other	0.5	subcutances other days	high	medium	hard	-0.643869086
297	10	1	other	2.5	mascular once a weak	high	low	hard	0.00383736
297	10	0	iran	5.5	mascular once a weak	medium	medium	easy	-0.697626621
297	11	1	other	5.5	subcutances other days	high	medium	easy	-0.34108841
297	11	0	iran	0.5	subcutances three times a weak	medium	low	hard	-0.699689278
297	12	0	iran	5.5	subcutances three times a weak	high	medium	hard	-0.787756632
297	12	1	other	2.5	subcutances other days	medium	low	hard	-0.352164436
298	1	1	iran	5.5	mascular once a weak	high	medium	hard	-0.572088286
298	1	0	iran	0.5	subcutances three times a weak	high	medium	easy	-0.72867963
298	2	0	other	0.5	subcutances other days	high	medium	hard	-0.643869086
298	2	1	iran	5.5	subcutances other days	medium	medium	hard	-0.928090082
298	3	0	iran	5.5	mascular once a weak	high	medium	hard	-0.572088286
298	3	1	iran	2.5	subcutances three times a weak	high	medium	easy	-0.646894171
298	4	1	iran	0.5	subcutances three times a weak	medium	low	hard	-0.699689278
298	4	0	other	2.5	mascular once a weak	high	low	hard	0.00383736
298	5	1	iran	0.5	mascular once a weak	medium	low	easy	-0.303092094
298	5	0	other	2.5	subcutances three times a weak	high	low	easy	-0.030902146

298	6	0	other	2.5	subcutances three times a weak	high	low	easy	-0.030902146
298	6	1	other	0.5	subcutances other days	high	medium	easy	-0.462940247
298	7	1	other	0.5	mascular once a weak	medium	medium	hard	-0.900801637
298	7	0	other	5.5	subcutances other days	medium	low	hard	-0.312098058
298	8	1	iran	2.5	mascular once a weak	high	low	hard	-0.0957683
298	8	0	iran	5.5	subcutances three times a weak	high	low	easy	-0.090441429
298	9	1	iran	2.5	subcutances three times a weak	medium	low	hard	-0.617903819
298	9	0	other	0.5	subcutances other days	high	medium	hard	-0.643869086
298	10	0	other	2.5	mascular once a weak	high	low	hard	0.00383736
298	10	1	iran	5.5	mascular once a weak	medium	medium	easy	-0.697626621
298	11	0	other	5.5	subcutances other days	high	medium	easy	-0.34108841
298	11	1	iran	0.5	subcutances three times a weak	medium	low	hard	-0.699689278
298	12	0	iran	5.5	subcutances three times a weak	high	medium	hard	-0.787756632
298	12	1	other	2.5	subcutances other days	medium	low	hard	-0.352164436
299	1	1	iran	5.5	mascular once a weak	high	medium	hard	-0.572088286
299	1	0	iran	0.5	subcutances three times a weak	high	medium	easy	-0.72867963
299	2	1	other	0.5	subcutances other days	high	medium	hard	-0.643869086
299	2	0	iran	5.5	subcutances other days	medium	medium	hard	-0.928090082
299	3	0	iran	5.5	mascular once a weak	high	medium	hard	-0.572088286
299	3	1	iran	2.5	subcutances three times a weak	high	medium	easy	-0.646894171
299	4	0	iran	0.5	subcutances three times a weak	medium	low	hard	-0.699689278
299	4	1	other	2.5	mascular once a weak	high	low	hard	0.00383736
299	5	0	iran	0.5	mascular once a weak	medium	low	easy	-0.303092094
299	5	1	other	2.5	subcutances three times a weak	high	low	easy	-0.030902146
299	6	0	other	2.5	subcutances three times a weak	high	low	easy	-0.030902146
299	6	1	other	0.5	subcutances other days	high	medium	easy	-0.462940247
299	7	0	other	0.5	mascular once a weak	medium	medium	hard	-0.900801637
299	7	1	other	5.5	subcutances other days	medium	low	hard	-0.312098058
299	8	1	iran	2.5	mascular once a weak	high	low	hard	-0.0957683
299	8	0	iran	5.5	subcutances three times a weak	high	low	easy	-0.090441429
299	9	1	iran	2.5	subcutances three times a weak	medium	low	hard	-0.617903819
299	9	0	other	0.5	subcutances other days	high	medium	hard	-0.643869086
299	10	0	other	2.5	mascular once a weak	high	low	hard	0.00383736
299	10	1	iran	5.5	mascular once a weak	medium	medium	easy	-0.697626621

299	11	1	other	5.5	subcutances other days	high	medium	easy	-0.34108841
299	11	0	iran	0.5	subcutances three times a weak	medium	low	hard	-0.699689278
299	12	1	iran	5.5	subcutances three times a weak	high	medium	hard	-0.787756632
299	12	0	other	2.5	subcutances other days	medium	low	hard	-0.352164436
300	1	0	iran	5.5	mascular once a weak	high	medium	hard	-0.572088286
300	1	1	iran	0.5	subcutances three times a weak	high	medium	easy	-0.72867963
300	2	0	other	0.5	subcutances other days	high	medium	hard	-0.643869086
300	2	1	iran	5.5	subcutances other days	medium	medium	hard	-0.928090082
300	3	0	iran	5.5	mascular once a weak	high	medium	hard	-0.572088286
300	3	1	iran	2.5	subcutances three times a weak	high	medium	easy	-0.646894171
300	4	0	iran	0.5	subcutances three times a weak	medium	low	hard	-0.699689278
300	4	1	other	2.5	mascular once a weak	high	low	hard	0.00383736
300	5	0	iran	0.5	mascular once a weak	medium	low	easy	-0.303092094
300	5	1	other	2.5	subcutances three times a weak	high	low	easy	-0.030902146
300	6	1	other	2.5	subcutances three times a weak	high	low	easy	-0.030902146
300	6	0	other	0.5	subcutances other days	high	medium	easy	-0.462940247
300	7	0	other	0.5	mascular once a weak	medium	medium	hard	-0.900801637
300	7	1	other	5.5	subcutances other days	medium	low	hard	-0.312098058
300	8	0	iran	2.5	mascular once a weak	high	low	hard	-0.0957683
300	8	1	iran	5.5	subcutances three times a weak	high	low	easy	-0.090441429
300	9	0	iran	2.5	subcutances three times a weak	medium	low	hard	-0.617903819
300	9	1	other	0.5	subcutances other days	high	medium	hard	-0.643869086
300	10	1	other	2.5	mascular once a weak	high	low	hard	0.00383736
300	10	0	iran	5.5	mascular once a weak	medium	medium	easy	-0.697626621
300	11	1	other	5.5	subcutances other days	high	medium	easy	-0.34108841
300	11	0	iran	0.5	subcutances three times a weak	medium	low	hard	-0.699689278
300	12	1	iran	5.5	subcutances three times a weak	high	medium	hard	-0.787756632
300	12	0	other	2.5	subcutances other days	medium	low	hard	-0.352164436
301	1	1	iran	5.5	mascular once a weak	high	medium	hard	-0.572088286
301	1	0	iran	0.5	subcutances three times a weak	high	medium	easy	-0.72867963
301	2	1	other	0.5	subcutances other days	high	medium	hard	-0.643869086
301	2	0	iran	5.5	subcutances other days	medium	medium	hard	-0.928090082
301	3	0	iran	5.5	mascular once a weak	high	medium	hard	-0.572088286
301	3	1	iran	2.5	subcutances three times a weak	high	medium	easy	-0.646894171

301	4	1	iran	0.5	subcutances three times a weak	medium	low	hard	-0.699689278
301	4	0	other	2.5	mascular once a weak	high	low	hard	0.00383736
301	5	0	iran	0.5	mascular once a weak	medium	low	easy	-0.303092094
301	5	1	other	2.5	subcutances three times a weak	high	low	easy	-0.030902146
301	6	0	other	2.5	subcutances three times a weak	high	low	easy	-0.030902146
301	6	1	other	0.5	subcutances other days	high	medium	easy	-0.462940247
301	7	0	other	0.5	mascular once a weak	medium	medium	hard	-0.900801637
301	7	1	other	5.5	subcutances other days	medium	low	hard	-0.312098058
301	8	1	iran	2.5	mascular once a weak	high	low	hard	-0.0957683
301	8	0	iran	5.5	subcutances three times a weak	high	low	easy	-0.090441429
301	9	1	iran	2.5	subcutances three times a weak	medium	low	hard	-0.617903819
301	9	0	other	0.5	subcutances other days	high	medium	hard	-0.643869086
301	10	0	other	2.5	mascular once a weak	high	low	hard	0.00383736
301	10	1	iran	5.5	mascular once a weak	medium	medium	easy	-0.697626621
301	11	0	other	5.5	subcutances other days	high	medium	easy	-0.34108841
301	11	1	iran	0.5	subcutances three times a weak	medium	low	hard	-0.699689278
301	12	0	iran	5.5	subcutances three times a weak	high	medium	hard	-0.787756632
301	12	1	other	2.5	subcutances other days	medium	low	hard	-0.352164436
302	1	1	iran	5.5	mascular once a weak	high	medium	hard	-0.572088286
302	1	0	iran	0.5	subcutances three times a weak	high	medium	easy	-0.72867963
302	2	0	other	0.5	subcutances other days	high	medium	hard	-0.643869086
302	2	1	iran	5.5	subcutances other days	medium	medium	hard	-0.928090082
302	3	1	iran	5.5	mascular once a weak	high	medium	hard	-0.572088286
302	3	0	iran	2.5	subcutances three times a weak	high	medium	easy	-0.646894171
302	4	1	iran	0.5	subcutances three times a weak	medium	low	hard	-0.699689278
302	4	0	other	2.5	mascular once a weak	high	low	hard	0.00383736
302	5	1	iran	0.5	mascular once a weak	medium	low	easy	-0.303092094
302	5	0	other	2.5	subcutances three times a weak	high	low	easy	-0.030902146
302	6	0	other	2.5	subcutances three times a weak	high	low	easy	-0.030902146
302	6	1	other	0.5	subcutances other days	high	medium	easy	-0.462940247
302	7	1	other	0.5	mascular once a weak	medium	medium	hard	-0.900801637
302	7	0	other	5.5	subcutances other days	medium	low	hard	-0.312098058
302	8	1	iran	2.5	mascular once a weak	high	low	hard	-0.0957683
302	8	0	iran	5.5	subcutances three times a weak	high	low	easy	-0.090441429

302	9	0	iran	2.5	subcutances three times a weak	medium	low	hard	-0.617903819
302	9	1	other	0.5	subcutances other days	high	medium	hard	-0.643869086
302	10	1	other	2.5	mascular once a weak	high	low	hard	0.00383736
302	10	0	iran	5.5	mascular once a weak	medium	medium	easy	-0.697626621
302	11	0	other	5.5	subcutances other days	high	medium	easy	-0.34108841
302	11	1	iran	0.5	subcutances three times a weak	medium	low	hard	-0.699689278
302	12	1	iran	5.5	subcutances three times a weak	high	medium	hard	-0.787756632
302	12	0	other	2.5	subcutances other days	medium	low	hard	-0.352164436
303	1	1	iran	5.5	mascular once a weak	high	medium	hard	-0.572088286
303	1	0	iran	0.5	subcutances three times a weak	high	medium	easy	-0.72867963
303	2	1	other	0.5	subcutances other days	high	medium	hard	-0.643869086
303	2	0	iran	5.5	subcutances other days	medium	medium	hard	-0.928090082
303	3	0	iran	5.5	mascular once a weak	high	medium	hard	-0.572088286
303	3	1	iran	2.5	subcutances three times a weak	high	medium	easy	-0.646894171
303	4	0	iran	0.5	subcutances three times a weak	medium	low	hard	-0.699689278
303	4	1	other	2.5	mascular once a weak	high	low	hard	0.00383736
303	5	0	iran	0.5	mascular once a weak	medium	low	easy	-0.303092094
303	5	1	other	2.5	subcutances three times a weak	high	low	easy	-0.030902146
303	6	0	other	2.5	subcutances three times a weak	high	low	easy	-0.030902146
303	6	1	other	0.5	subcutances other days	high	medium	easy	-0.462940247
303	7	0	other	0.5	mascular once a weak	medium	medium	hard	-0.900801637
303	7	1	other	5.5	subcutances other days	medium	low	hard	-0.312098058
303	8	1	iran	2.5	mascular once a weak	high	low	hard	-0.0957683
303	8	0	iran	5.5	subcutances three times a weak	high	low	easy	-0.090441429
303	9	0	iran	2.5	subcutances three times a weak	medium	low	hard	-0.617903819
303	9	1	other	0.5	subcutances other days	high	medium	hard	-0.643869086
303	10	1	other	2.5	mascular once a weak	high	low	hard	0.00383736
303	10	0	iran	5.5	mascular once a weak	medium	medium	easy	-0.697626621
303	11	1	other	5.5	subcutances other days	high	medium	easy	-0.34108841
303	11	0	iran	0.5	subcutances three times a weak	medium	low	hard	-0.699689278
303	12	1	iran	5.5	subcutances three times a weak	high	medium	hard	-0.787756632
303	12	0	other	2.5	subcutances other days	medium	low	hard	-0.352164436
304	1	1	iran	5.5	mascular once a weak	high	medium	hard	-0.572088286
304	1	0	iran	0.5	subcutances three times a weak	high	medium	easy	-0.72867963

304	2	1	other	0.5	subcutances other days	high	medium	hard	-0.643869086
304	2	0	iran	5.5	subcutances other days	medium	medium	hard	-0.928090082
304	3	1	iran	5.5	mascular once a weak	high	medium	hard	-0.572088286
304	3	0	iran	2.5	subcutances three times a weak	high	medium	easy	-0.646894171
304	4	0	iran	0.5	subcutances three times a weak	medium	low	hard	-0.699689278
304	4	1	other	2.5	mascular once a weak	high	low	hard	0.00383736
304	5	1	iran	0.5	mascular once a weak	medium	low	easy	-0.303092094
304	5	0	other	2.5	subcutances three times a weak	high	low	easy	-0.030902146
304	6	1	other	2.5	subcutances three times a weak	high	low	easy	-0.030902146
304	6	0	other	0.5	subcutances other days	high	medium	easy	-0.462940247
304	7	1	other	0.5	mascular once a weak	medium	medium	hard	-0.900801637
304	7	0	other	5.5	subcutances other days	medium	low	hard	-0.312098058
304	8	1	iran	2.5	mascular once a weak	high	low	hard	-0.0957683
304	8	0	iran	5.5	subcutances three times a weak	high	low	easy	-0.090441429
304	9	1	iran	2.5	subcutances three times a weak	medium	low	hard	-0.617903819
304	9	0	other	0.5	subcutances other days	high	medium	hard	-0.643869086
304	10	1	other	2.5	mascular once a weak	high	low	hard	0.00383736
304	10	0	iran	5.5	mascular once a weak	medium	medium	easy	-0.697626621
304	11	0	other	5.5	subcutances other days	high	medium	easy	-0.34108841
304	11	1	iran	0.5	subcutances three times a weak	medium	low	hard	-0.699689278
304	12	0	iran	5.5	subcutances three times a weak	high	medium	hard	-0.787756632
304	12	1	other	2.5	subcutances other days	medium	low	hard	-0.352164436
305	1	1	iran	5.5	mascular once a weak	high	medium	hard	-0.572088286
305	1	0	iran	0.5	subcutances three times a weak	high	medium	easy	-0.72867963
305	2	1	other	0.5	subcutances other days	high	medium	hard	-0.643869086
305	2	0	iran	5.5	subcutances other days	medium	medium	hard	-0.928090082
305	3	1	iran	5.5	mascular once a weak	high	medium	hard	-0.572088286
305	3	0	iran	2.5	subcutances three times a weak	high	medium	easy	-0.646894171
305	4	0	iran	0.5	subcutances three times a weak	medium	low	hard	-0.699689278
305	4	1	other	2.5	mascular once a weak	high	low	hard	0.00383736
305	5	0	iran	0.5	mascular once a weak	medium	low	easy	-0.303092094
305	5	1	other	2.5	subcutances three times a weak	high	low	easy	-0.030902146
305	6	1	other	2.5	subcutances three times a weak	high	low	easy	-0.030902146
305	6	0	other	0.5	subcutances other days	high	medium	easy	-0.462940247

305	7	0	other	0.5	mascular once a weak	medium	medium	hard	-0.900801637
305	7	1	other	5.5	subcutances other days	medium	low	hard	-0.312098058
305	8	1	iran	2.5	mascular once a weak	high	low	hard	-0.0957683
305	8	0	iran	5.5	subcutances three times a weak	high	low	easy	-0.090441429
305	9	1	iran	2.5	subcutances three times a weak	medium	low	hard	-0.617903819
305	9	0	other	0.5	subcutances other days	high	medium	hard	-0.643869086
305	10	1	other	2.5	mascular once a weak	high	low	hard	0.00383736
305	10	0	iran	5.5	mascular once a weak	medium	medium	easy	-0.697626621
305	11	1	other	5.5	subcutances other days	high	medium	easy	-0.34108841
305	11	0	iran	0.5	subcutances three times a weak	medium	low	hard	-0.699689278
305	12	0	iran	5.5	subcutances three times a weak	high	medium	hard	-0.787756632
305	12	1	other	2.5	subcutances other days	medium	low	hard	-0.352164436
306	1	1	iran	5.5	mascular once a weak	high	medium	hard	-0.572088286
306	1	0	iran	0.5	subcutances three times a weak	high	medium	easy	-0.72867963
306	2	1	other	0.5	subcutances other days	high	medium	hard	-0.643869086
306	2	0	iran	5.5	subcutances other days	medium	medium	hard	-0.928090082
306	3	1	iran	5.5	mascular once a weak	high	medium	hard	-0.572088286
306	3	0	iran	2.5	subcutances three times a weak	high	medium	easy	-0.646894171
306	4	0	iran	0.5	subcutances three times a weak	medium	low	hard	-0.699689278
306	4	1	other	2.5	mascular once a weak	high	low	hard	0.00383736
306	5	0	iran	0.5	mascular once a weak	medium	low	easy	-0.303092094
306	5	1	other	2.5	subcutances three times a weak	high	low	easy	-0.030902146
306	6	1	other	2.5	subcutances three times a weak	high	low	easy	-0.030902146
306	6	0	other	0.5	subcutances other days	high	medium	easy	-0.462940247
306	7	0	other	0.5	mascular once a weak	medium	medium	hard	-0.900801637
306	7	1	other	5.5	subcutances other days	medium	low	hard	-0.312098058
306	8	1	iran	2.5	mascular once a weak	high	low	hard	-0.0957683
306	8	0	iran	5.5	subcutances three times a weak	high	low	easy	-0.090441429
306	9	1	iran	2.5	subcutances three times a weak	medium	low	hard	-0.617903819
306	9	0	other	0.5	subcutances other days	high	medium	hard	-0.643869086
306	10	1	other	2.5	mascular once a weak	high	low	hard	0.00383736
306	10	0	iran	5.5	mascular once a weak	medium	medium	easy	-0.697626621
306	11	1	other	5.5	subcutances other days	high	medium	easy	-0.34108841
306	11	0	iran	0.5	subcutances three times a weak	medium	low	hard	-0.699689278

306	12	1	iran	5.5	subcutances three times a weak	high	medium	hard	-0.787756632
306	12	0	other	2.5	subcutances other days	medium	low	hard	-0.352164436
307	1	1	iran	5.5	mascular once a weak	high	medium	hard	-0.572088286
307	1	0	iran	0.5	subcutances three times a weak	high	medium	easy	-0.72867963
307	2	1	other	0.5	subcutances other days	high	medium	hard	-0.643869086
307	2	0	iran	5.5	subcutances other days	medium	medium	hard	-0.928090082
307	3	1	iran	5.5	mascular once a weak	high	medium	hard	-0.572088286
307	3	0	iran	2.5	subcutances three times a weak	high	medium	easy	-0.646894171
307	4	0	iran	0.5	subcutances three times a weak	medium	low	hard	-0.699689278
307	4	1	other	2.5	mascular once a weak	high	low	hard	0.00383736
307	5	1	iran	0.5	mascular once a weak	medium	low	easy	-0.303092094
307	5	0	other	2.5	subcutances three times a weak	high	low	easy	-0.030902146
307	6	1	other	2.5	subcutances three times a weak	high	low	easy	-0.030902146
307	6	0	other	0.5	subcutances other days	high	medium	easy	-0.462940247
307	7	1	other	0.5	mascular once a weak	medium	medium	hard	-0.900801637
307	7	0	other	5.5	subcutances other days	medium	low	hard	-0.312098058
307	8	1	iran	2.5	mascular once a weak	high	low	hard	-0.0957683
307	8	0	iran	5.5	subcutances three times a weak	high	low	easy	-0.090441429
307	9	1	iran	2.5	subcutances three times a weak	medium	low	hard	-0.617903819
307	9	0	other	0.5	subcutances other days	high	medium	hard	-0.643869086
307	10	1	other	2.5	mascular once a weak	high	low	hard	0.00383736
307	10	0	iran	5.5	mascular once a weak	medium	medium	easy	-0.697626621
307	11	1	other	5.5	subcutances other days	high	medium	easy	-0.34108841
307	11	0	iran	0.5	subcutances three times a weak	medium	low	hard	-0.699689278
307	12	0	iran	5.5	subcutances three times a weak	high	medium	hard	-0.787756632
307	12	1	other	2.5	subcutances other days	medium	low	hard	-0.352164436
308	1	1	iran	5.5	mascular once a weak	high	medium	hard	-0.572088286
308	1	0	iran	0.5	subcutances three times a weak	high	medium	easy	-0.72867963
308	2	1	other	0.5	subcutances other days	high	medium	hard	-0.643869086
308	2	0	iran	5.5	subcutances other days	medium	medium	hard	-0.928090082
308	3	0	iran	5.5	mascular once a weak	high	medium	hard	-0.572088286
308	3	1	iran	2.5	subcutances three times a weak	high	medium	easy	-0.646894171
308	4	0	iran	0.5	subcutances three times a weak	medium	low	hard	-0.699689278
308	4	1	other	2.5	mascular once a weak	high	low	hard	0.00383736

308	5	1	iran	0.5	mascular once a weak	medium	low	easy	-0.303092094
308	5	0	other	2.5	subcutances three times a weak	high	low	easy	-0.030902146
308	6	1	other	2.5	subcutances three times a weak	high	low	easy	-0.030902146
308	6	0	other	0.5	subcutances other days	high	medium	easy	-0.462940247
308	7	0	other	0.5	mascular once a weak	medium	medium	hard	-0.900801637
308	7	1	other	5.5	subcutances other days	medium	low	hard	-0.312098058
308	8	1	iran	2.5	mascular once a weak	high	low	hard	-0.0957683
308	8	0	iran	5.5	subcutances three times a weak	high	low	easy	-0.090441429
308	9	1	iran	2.5	subcutances three times a weak	medium	low	hard	-0.617903819
308	9	0	other	0.5	subcutances other days	high	medium	hard	-0.643869086
308	10	1	other	2.5	mascular once a weak	high	low	hard	0.00383736
308	10	0	iran	5.5	mascular once a weak	medium	medium	easy	-0.697626621
308	11	1	other	5.5	subcutances other days	high	medium	easy	-0.34108841
308	11	0	iran	0.5	subcutances three times a weak	medium	low	hard	-0.699689278
308	12	1	iran	5.5	subcutances three times a weak	high	medium	hard	-0.787756632
308	12	0	other	2.5	subcutances other days	medium	low	hard	-0.352164436
309	1	1	iran	5.5	mascular once a weak	high	medium	hard	-0.572088286
309	1	0	iran	0.5	subcutances three times a weak	high	medium	easy	-0.72867963
309	2	1	other	0.5	subcutances other days	high	medium	hard	-0.643869086
309	2	0	iran	5.5	subcutances other days	medium	medium	hard	-0.928090082
309	3	1	iran	5.5	mascular once a weak	high	medium	hard	-0.572088286
309	3	0	iran	2.5	subcutances three times a weak	high	medium	easy	-0.646894171
309	4	0	iran	0.5	subcutances three times a weak	medium	low	hard	-0.699689278
309	4	1	other	2.5	mascular once a weak	high	low	hard	0.00383736
309	5	1	iran	0.5	mascular once a weak	medium	low	easy	-0.303092094
309	5	0	other	2.5	subcutances three times a weak	high	low	easy	-0.030902146
309	6	1	other	2.5	subcutances three times a weak	high	low	easy	-0.030902146
309	6	0	other	0.5	subcutances other days	high	medium	easy	-0.462940247
309	7	0	other	0.5	mascular once a weak	medium	medium	hard	-0.900801637
309	7	1	other	5.5	subcutances other days	medium	low	hard	-0.312098058
309	8	1	iran	2.5	mascular once a weak	high	low	hard	-0.0957683
309	8	0	iran	5.5	subcutances three times a weak	high	low	easy	-0.090441429
309	9	1	iran	2.5	subcutances three times a weak	medium	low	hard	-0.617903819
309	9	0	other	0.5	subcutances other days	high	medium	hard	-0.643869086

309	10	1	other	2.5	mascular once a weak	high	low	hard	0.00383736
309	10	0	iran	5.5	mascular once a weak	medium	medium	easy	-0.697626621
309	11	0	other	5.5	subcutances other days	high	medium	easy	-0.34108841
309	11	1	iran	0.5	subcutances three times a weak	medium	low	hard	-0.699689278
309	12	0	iran	5.5	subcutances three times a weak	high	medium	hard	-0.787756632
309	12	1	other	2.5	subcutances other days	medium	low	hard	-0.352164436
310	1	1	iran	5.5	mascular once a weak	high	medium	hard	-0.572088286
310	1	0	iran	0.5	subcutances three times a weak	high	medium	easy	-0.72867963
310	2	1	other	0.5	subcutances other days	high	medium	hard	-0.643869086
310	2	0	iran	5.5	subcutances other days	medium	medium	hard	-0.928090082
310	3	0	iran	5.5	mascular once a weak	high	medium	hard	-0.572088286
310	3	1	iran	2.5	subcutances three times a weak	high	medium	easy	-0.646894171
310	4	0	iran	0.5	subcutances three times a weak	medium	low	hard	-0.699689278
310	4	1	other	2.5	mascular once a weak	high	low	hard	0.00383736
310	5	0	iran	0.5	mascular once a weak	medium	low	easy	-0.303092094
310	5	1	other	2.5	subcutances three times a weak	high	low	easy	-0.030902146
310	6	0	other	2.5	subcutances three times a weak	high	low	easy	-0.030902146
310	6	1	other	0.5	subcutances other days	high	medium	easy	-0.462940247
310	7	0	other	0.5	mascular once a weak	medium	medium	hard	-0.900801637
310	7	1	other	5.5	subcutances other days	medium	low	hard	-0.312098058
310	8	0	iran	2.5	mascular once a weak	high	low	hard	-0.0957683
310	8	1	iran	5.5	subcutances three times a weak	high	low	easy	-0.090441429
310	9	0	iran	2.5	subcutances three times a weak	medium	low	hard	-0.617903819
310	9	1	other	0.5	subcutances other days	high	medium	hard	-0.643869086
310	10	1	other	2.5	mascular once a weak	high	low	hard	0.00383736
310	10	0	iran	5.5	mascular once a weak	medium	medium	easy	-0.697626621
310	11	1	other	5.5	subcutances other days	high	medium	easy	-0.34108841
310	11	0	iran	0.5	subcutances three times a weak	medium	low	hard	-0.699689278
310	12	1	iran	5.5	subcutances three times a weak	high	medium	hard	-0.787756632
310	12	0	other	2.5	subcutances other days	medium	low	hard	-0.352164436
311	1	1	iran	5.5	mascular once a weak	high	medium	hard	-0.572088286
311	1	0	iran	0.5	subcutances three times a weak	high	medium	easy	-0.72867963
311	2	1	other	0.5	subcutances other days	high	medium	hard	-0.643869086
311	2	0	iran	5.5	subcutances other days	medium	medium	hard	-0.928090082

311	3	1	iran	5.5	mascular once a weak	high	medium	hard	-0.572088286
311	3	0	iran	2.5	subcutances three times a weak	high	medium	easy	-0.646894171
311	4	0	iran	0.5	subcutances three times a weak	medium	low	hard	-0.699689278
311	4	1	other	2.5	mascular once a weak	high	low	hard	0.00383736
311	5	0	iran	0.5	mascular once a weak	medium	low	easy	-0.303092094
311	5	1	other	2.5	subcutances three times a weak	high	low	easy	-0.030902146
311	6	1	other	2.5	subcutances three times a weak	high	low	easy	-0.030902146
311	6	0	other	0.5	subcutances other days	high	medium	easy	-0.462940247
311	7	0	other	0.5	mascular once a weak	medium	medium	hard	-0.900801637
311	7	1	other	5.5	subcutances other days	medium	low	hard	-0.312098058
311	8	1	iran	2.5	mascular once a weak	high	low	hard	-0.0957683
311	8	0	iran	5.5	subcutances three times a weak	high	low	easy	-0.090441429
311	9	0	iran	2.5	subcutances three times a weak	medium	low	hard	-0.617903819
311	9	1	other	0.5	subcutances other days	high	medium	hard	-0.643869086
311	10	1	other	2.5	mascular once a weak	high	low	hard	0.00383736
311	10	0	iran	5.5	mascular once a weak	medium	medium	easy	-0.697626621
311	11	1	other	5.5	subcutances other days	high	medium	easy	-0.34108841
311	11	0	iran	0.5	subcutances three times a weak	medium	low	hard	-0.699689278
311	12	0	iran	5.5	subcutances three times a weak	high	medium	hard	-0.787756632
311	12	1	other	2.5	subcutances other days	medium	low	hard	-0.352164436
312	1	1	iran	5.5	mascular once a weak	high	medium	hard	-0.572088286
312	1	0	iran	0.5	subcutances three times a weak	high	medium	easy	-0.72867963
312	2	1	other	0.5	subcutances other days	high	medium	hard	-0.643869086
312	2	0	iran	5.5	subcutances other days	medium	medium	hard	-0.928090082
312	3	1	iran	5.5	mascular once a weak	high	medium	hard	-0.572088286
312	3	0	iran	2.5	subcutances three times a weak	high	medium	easy	-0.646894171
312	4	0	iran	0.5	subcutances three times a weak	medium	low	hard	-0.699689278
312	4	1	other	2.5	mascular once a weak	high	low	hard	0.00383736
312	5	0	iran	0.5	mascular once a weak	medium	low	easy	-0.303092094
312	5	1	other	2.5	subcutances three times a weak	high	low	easy	-0.030902146
312	6	0	other	2.5	subcutances three times a weak	high	low	easy	-0.030902146
312	6	1	other	0.5	subcutances other days	high	medium	easy	-0.462940247
312	7	1	other	0.5	mascular once a weak	medium	medium	hard	-0.900801637
312	7	0	other	5.5	subcutances other days	medium	low	hard	-0.312098058

312	8	1	iran	2.5	mascular once a weak	high	low	hard	-0.0957683
312	8	0	iran	5.5	subcutances three times a weak	high	low	easy	-0.090441429
312	9	1	iran	2.5	subcutances three times a weak	medium	low	hard	-0.617903819
312	9	0	other	0.5	subcutances other days	high	medium	hard	-0.643869086
312	10	1	other	2.5	mascular once a weak	high	low	hard	0.00383736
312	10	0	iran	5.5	mascular once a weak	medium	medium	easy	-0.697626621
312	11	0	other	5.5	subcutances other days	high	medium	easy	-0.34108841
312	11	1	iran	0.5	subcutances three times a weak	medium	low	hard	-0.699689278
312	12	0	iran	5.5	subcutances three times a weak	high	medium	hard	-0.787756632
312	12	1	other	2.5	subcutances other days	medium	low	hard	-0.352164436
313	1	1	iran	5.5	mascular once a weak	high	medium	hard	-0.572088286
313	1	0	iran	0.5	subcutances three times a weak	high	medium	easy	-0.72867963
313	2	0	other	0.5	subcutances other days	high	medium	hard	-0.643869086
313	2	1	iran	5.5	subcutances other days	medium	medium	hard	-0.928090082
313	3	1	iran	5.5	mascular once a weak	high	medium	hard	-0.572088286
313	3	0	iran	2.5	subcutances three times a weak	high	medium	easy	-0.646894171
313	4	0	iran	0.5	subcutances three times a weak	medium	low	hard	-0.699689278
313	4	1	other	2.5	mascular once a weak	high	low	hard	0.00383736
313	5	1	iran	0.5	mascular once a weak	medium	low	easy	-0.303092094
313	5	0	other	2.5	subcutances three times a weak	high	low	easy	-0.030902146
313	6	0	other	2.5	subcutances three times a weak	high	low	easy	-0.030902146
313	6	1	other	0.5	subcutances other days	high	medium	easy	-0.462940247
313	7	1	other	0.5	mascular once a weak	medium	medium	hard	-0.900801637
313	7	0	other	5.5	subcutances other days	medium	low	hard	-0.312098058
313	8	1	iran	2.5	mascular once a weak	high	low	hard	-0.0957683
313	8	0	iran	5.5	subcutances three times a weak	high	low	easy	-0.090441429
313	9	0	iran	2.5	subcutances three times a weak	medium	low	hard	-0.617903819
313	9	1	other	0.5	subcutances other days	high	medium	hard	-0.643869086
313	10	1	other	2.5	mascular once a weak	high	low	hard	0.00383736
313	10	0	iran	5.5	mascular once a weak	medium	medium	easy	-0.697626621
313	11	0	other	5.5	subcutances other days	high	medium	easy	-0.34108841
313	11	1	iran	0.5	subcutances three times a weak	medium	low	hard	-0.699689278
313	12	1	iran	5.5	subcutances three times a weak	high	medium	hard	-0.787756632
313	12	0	other	2.5	subcutances other days	medium	low	hard	-0.352164436

314	1	0	iran	5.5	mascular once a weak	high	medium	hard	-0.572088286
314	1	1	iran	0.5	subcutances three times a weak	high	medium	easy	-0.72867963
314	2	0	other	0.5	subcutances other days	high	medium	hard	-0.643869086
314	2	1	iran	5.5	subcutances other days	medium	medium	hard	-0.928090082
314	3	0	iran	5.5	mascular once a weak	high	medium	hard	-0.572088286
314	3	1	iran	2.5	subcutances three times a weak	high	medium	easy	-0.646894171
314	4	1	iran	0.5	subcutances three times a weak	medium	low	hard	-0.699689278
314	4	0	other	2.5	mascular once a weak	high	low	hard	0.00383736
314	5	1	iran	0.5	mascular once a weak	medium	low	easy	-0.303092094
314	5	0	other	2.5	subcutances three times a weak	high	low	easy	-0.030902146
314	6	0	other	2.5	subcutances three times a weak	high	low	easy	-0.030902146
314	6	1	other	0.5	subcutances other days	high	medium	easy	-0.462940247
314	7	0	other	0.5	mascular once a weak	medium	medium	hard	-0.900801637
314	7	1	other	5.5	subcutances other days	medium	low	hard	-0.312098058
314	8	1	iran	2.5	mascular once a weak	high	low	hard	-0.0957683
314	8	0	iran	5.5	subcutances three times a weak	high	low	easy	-0.090441429
314	9	1	iran	2.5	subcutances three times a weak	medium	low	hard	-0.617903819
314	9	0	other	0.5	subcutances other days	high	medium	hard	-0.643869086
314	10	0	other	2.5	mascular once a weak	high	low	hard	0.00383736
314	10	1	iran	5.5	mascular once a weak	medium	medium	easy	-0.697626621
314	11	0	other	5.5	subcutances other days	high	medium	easy	-0.34108841
314	11	1	iran	0.5	subcutances three times a weak	medium	low	hard	-0.699689278
314	12	1	iran	5.5	subcutances three times a weak	high	medium	hard	-0.787756632
314	12	0	other	2.5	subcutances other days	medium	low	hard	-0.352164436
315	1	0	iran	5.5	mascular once a weak	high	medium	hard	-0.572088286
315	1	1	iran	0.5	subcutances three times a weak	high	medium	easy	-0.72867963
315	2	1	other	0.5	subcutances other days	high	medium	hard	-0.643869086
315	2	0	iran	5.5	subcutances other days	medium	medium	hard	-0.928090082
315	3	0	iran	5.5	mascular once a weak	high	medium	hard	-0.572088286
315	3	1	iran	2.5	subcutances three times a weak	high	medium	easy	-0.646894171
315	4	0	iran	0.5	subcutances three times a weak	medium	low	hard	-0.699689278
315	4	1	other	2.5	mascular once a weak	high	low	hard	0.00383736
315	5	0	iran	0.5	mascular once a weak	medium	low	easy	-0.303092094
315	5	1	other	2.5	subcutances three times a weak	high	low	easy	-0.030902146

315	6	0	other	2.5	subcutances three times a weak	high	low	easy	-0.030902146
315	6	1	other	0.5	subcutances other days	high	medium	easy	-0.462940247
315	7	1	other	0.5	mascular once a weak	medium	medium	hard	-0.900801637
315	7	0	other	5.5	subcutances other days	medium	low	hard	-0.312098058
315	8	0	iran	2.5	mascular once a weak	high	low	hard	-0.0957683
315	8	1	iran	5.5	subcutances three times a weak	high	low	easy	-0.090441429
315	9	0	iran	2.5	subcutances three times a weak	medium	low	hard	-0.617903819
315	9	1	other	0.5	subcutances other days	high	medium	hard	-0.643869086
315	10	0	other	2.5	mascular once a weak	high	low	hard	0.00383736
315	10	1	iran	5.5	mascular once a weak	medium	medium	easy	-0.697626621
315	11	1	other	5.5	subcutances other days	high	medium	easy	-0.34108841
315	11	0	iran	0.5	subcutances three times a weak	medium	low	hard	-0.699689278
315	12	1	iran	5.5	subcutances three times a weak	high	medium	hard	-0.787756632
315	12	0	other	2.5	subcutances other days	medium	low	hard	-0.352164436
316	1	0	iran	5.5	mascular once a weak	high	medium	hard	-0.572088286
316	1	1	iran	0.5	subcutances three times a weak	high	medium	easy	-0.72867963
316	2	1	other	0.5	subcutances other days	high	medium	hard	-0.643869086
316	2	0	iran	5.5	subcutances other days	medium	medium	hard	-0.928090082
316	3	0	iran	5.5	mascular once a weak	high	medium	hard	-0.572088286
316	3	1	iran	2.5	subcutances three times a weak	high	medium	easy	-0.646894171
316	4	0	iran	0.5	subcutances three times a weak	medium	low	hard	-0.699689278
316	4	1	other	2.5	mascular once a weak	high	low	hard	0.00383736
316	5	0	iran	0.5	mascular once a weak	medium	low	easy	-0.303092094
316	5	1	other	2.5	subcutances three times a weak	high	low	easy	-0.030902146
316	6	1	other	2.5	subcutances three times a weak	high	low	easy	-0.030902146
316	6	0	other	0.5	subcutances other days	high	medium	easy	-0.462940247
316	7	0	other	0.5	mascular once a weak	medium	medium	hard	-0.900801637
316	7	1	other	5.5	subcutances other days	medium	low	hard	-0.312098058
316	8	0	iran	2.5	mascular once a weak	high	low	hard	-0.0957683
316	8	1	iran	5.5	subcutances three times a weak	high	low	easy	-0.090441429
316	9	1	iran	2.5	subcutances three times a weak	medium	low	hard	-0.617903819
316	9	0	other	0.5	subcutances other days	high	medium	hard	-0.643869086
316	10	0	other	2.5	mascular once a weak	high	low	hard	0.00383736
316	10	1	iran	5.5	mascular once a weak	medium	medium	easy	-0.697626621

316	11	1	other	5.5	subcutances other days	high	medium	easy	-0.34108841
316	11	0	iran	0.5	subcutances three times a weak	medium	low	hard	-0.699689278
316	12	0	iran	5.5	subcutances three times a weak	high	medium	hard	-0.787756632
316	12	1	other	2.5	subcutances other days	medium	low	hard	-0.352164436
317	1	1	iran	5.5	mascular once a weak	high	medium	hard	-0.572088286
317	1	0	iran	0.5	subcutances three times a weak	high	medium	easy	-0.72867963
317	2	0	other	0.5	subcutances other days	high	medium	hard	-0.643869086
317	2	1	iran	5.5	subcutances other days	medium	medium	hard	-0.928090082
317	3	1	iran	5.5	mascular once a weak	high	medium	hard	-0.572088286
317	3	0	iran	2.5	subcutances three times a weak	high	medium	easy	-0.646894171
317	4	0	iran	0.5	subcutances three times a weak	medium	low	hard	-0.699689278
317	4	1	other	2.5	mascular once a weak	high	low	hard	0.00383736
317	5	0	iran	0.5	mascular once a weak	medium	low	easy	-0.303092094
317	5	1	other	2.5	subcutances three times a weak	high	low	easy	-0.030902146
317	6	1	other	2.5	subcutances three times a weak	high	low	easy	-0.030902146
317	6	0	other	0.5	subcutances other days	high	medium	easy	-0.462940247
317	7	1	other	0.5	mascular once a weak	medium	medium	hard	-0.900801637
317	7	0	other	5.5	subcutances other days	medium	low	hard	-0.312098058
317	8	1	iran	2.5	mascular once a weak	high	low	hard	-0.0957683
317	8	0	iran	5.5	subcutances three times a weak	high	low	easy	-0.090441429
317	9	0	iran	2.5	subcutances three times a weak	medium	low	hard	-0.617903819
317	9	1	other	0.5	subcutances other days	high	medium	hard	-0.643869086
317	10	1	other	2.5	mascular once a weak	high	low	hard	0.00383736
317	10	0	iran	5.5	mascular once a weak	medium	medium	easy	-0.697626621
317	11	1	other	5.5	subcutances other days	high	medium	easy	-0.34108841
317	11	0	iran	0.5	subcutances three times a weak	medium	low	hard	-0.699689278
317	12	1	iran	5.5	subcutances three times a weak	high	medium	hard	-0.787756632
317	12	0	other	2.5	subcutances other days	medium	low	hard	-0.352164436
318	1	1	iran	5.5	mascular once a weak	high	medium	hard	-0.572088286
318	1	0	iran	0.5	subcutances three times a weak	high	medium	easy	-0.72867963
318	2	0	other	0.5	subcutances other days	high	medium	hard	-0.643869086
318	2	1	iran	5.5	subcutances other days	medium	medium	hard	-0.928090082
318	3	0	iran	5.5	mascular once a weak	high	medium	hard	-0.572088286
318	3	1	iran	2.5	subcutances three times a weak	high	medium	easy	-0.646894171

318	4	1	iran	0.5	subcutances three times a weak	medium	low	hard	-0.699689278
318	4	0	other	2.5	mascular once a weak	high	low	hard	0.00383736
318	5	1	iran	0.5	mascular once a weak	medium	low	easy	-0.303092094
318	5	0	other	2.5	subcutances three times a weak	high	low	easy	-0.030902146
318	6	0	other	2.5	subcutances three times a weak	high	low	easy	-0.030902146
318	6	1	other	0.5	subcutances other days	high	medium	easy	-0.462940247
318	7	0	other	0.5	mascular once a weak	medium	medium	hard	-0.900801637
318	7	1	other	5.5	subcutances other days	medium	low	hard	-0.312098058
318	8	1	iran	2.5	mascular once a weak	high	low	hard	-0.0957683
318	8	0	iran	5.5	subcutances three times a weak	high	low	easy	-0.090441429
318	9	1	iran	2.5	subcutances three times a weak	medium	low	hard	-0.617903819
318	9	0	other	0.5	subcutances other days	high	medium	hard	-0.643869086
318	10	0	other	2.5	mascular once a weak	high	low	hard	0.00383736
318	10	1	iran	5.5	mascular once a weak	medium	medium	easy	-0.697626621
318	11	0	other	5.5	subcutances other days	high	medium	easy	-0.34108841
318	11	1	iran	0.5	subcutances three times a weak	medium	low	hard	-0.699689278
318	12	0	iran	5.5	subcutances three times a weak	high	medium	hard	-0.787756632
318	12	1	other	2.5	subcutances other days	medium	low	hard	-0.352164436
319	1	0	iran	5.5	mascular once a weak	high	medium	hard	-0.572088286
319	1	0	iran	0.5	subcutances three times a weak	high	medium	easy	-0.72867963
319	2	1	other	0.5	subcutances other days	high	medium	hard	-0.643869086
319	2	0	iran	5.5	subcutances other days	medium	medium	hard	-0.928090082
319	3	0	iran	5.5	mascular once a weak	high	medium	hard	-0.572088286
319	3	1	iran	2.5	subcutances three times a weak	high	medium	easy	-0.646894171
319	4	0	iran	0.5	subcutances three times a weak	medium	low	hard	-0.699689278
319	4	1	other	2.5	mascular once a weak	high	low	hard	0.00383736
319	5	0	iran	0.5	mascular once a weak	medium	low	easy	-0.303092094
319	5	1	other	2.5	subcutances three times a weak	high	low	easy	-0.030902146
319	6	1	other	2.5	subcutances three times a weak	high	low	easy	-0.030902146
319	6	0	other	0.5	subcutances other days	high	medium	easy	-0.462940247
319	7	0	other	0.5	mascular once a weak	medium	medium	hard	-0.900801637
319	7	1	other	5.5	subcutances other days	medium	low	hard	-0.312098058
319	8	0	iran	2.5	mascular once a weak	high	low	hard	-0.0957683
319	8	1	iran	5.5	subcutances three times a weak	high	low	easy	-0.090441429

319	9	1	iran	2.5	subcutances three times a weak	medium	low	hard	-0.617903819
319	9	0	other	0.5	subcutances other days	high	medium	hard	-0.643869086
319	10	0	other	2.5	mascular once a weak	high	low	hard	0.00383736
319	10	1	iran	5.5	mascular once a weak	medium	medium	easy	-0.697626621
319	11	1	other	5.5	subcutances other days	high	medium	easy	-0.34108841
319	11	0	iran	0.5	subcutances three times a weak	medium	low	hard	-0.699689278
319	12	0	iran	5.5	subcutances three times a weak	high	medium	hard	-0.787756632
319	12	1	other	2.5	subcutances other days	medium	low	hard	-0.352164436
320	1	0	iran	5.5	mascular once a weak	high	medium	hard	-0.572088286
320	1	1	iran	0.5	subcutances three times a weak	high	medium	easy	-0.72867963
320	2	0	other	0.5	subcutances other days	high	medium	hard	-0.643869086
320	2	1	iran	5.5	subcutances other days	medium	medium	hard	-0.928090082
320	3	0	iran	5.5	mascular once a weak	high	medium	hard	-0.572088286
320	3	1	iran	2.5	subcutances three times a weak	high	medium	easy	-0.646894171
320	4	1	iran	0.5	subcutances three times a weak	medium	low	hard	-0.699689278
320	4	0	other	2.5	mascular once a weak	high	low	hard	0.00383736
320	5	0	iran	0.5	mascular once a weak	medium	low	easy	-0.303092094
320	5	1	other	2.5	subcutances three times a weak	high	low	easy	-0.030902146
320	6	0	other	2.5	subcutances three times a weak	high	low	easy	-0.030902146
320	6	1	other	0.5	subcutances other days	high	medium	easy	-0.462940247
320	7	0	other	0.5	mascular once a weak	medium	medium	hard	-0.900801637
320	7	1	other	5.5	subcutances other days	medium	low	hard	-0.312098058
320	8	1	iran	2.5	mascular once a weak	high	low	hard	-0.0957683
320	8	0	iran	5.5	subcutances three times a weak	high	low	easy	-0.090441429
320	9	1	iran	2.5	subcutances three times a weak	medium	low	hard	-0.617903819
320	9	0	other	0.5	subcutances other days	high	medium	hard	-0.643869086
320	10	0	other	2.5	mascular once a weak	high	low	hard	0.00383736
320	10	1	iran	5.5	mascular once a weak	medium	medium	easy	-0.697626621
320	11	0	other	5.5	subcutances other days	high	medium	easy	-0.34108841
320	11	1	iran	0.5	subcutances three times a weak	medium	low	hard	-0.699689278
320	12	0	iran	5.5	subcutances three times a weak	high	medium	hard	-0.787756632
320	12	1	other	2.5	subcutances other days	medium	low	hard	-0.352164436
321	1	0	iran	5.5	mascular once a weak	high	medium	hard	-0.572088286
321	1	1	iran	0.5	subcutances three times a weak	high	medium	easy	-0.72867963

321	2	1	other	0.5	subcutances	other days	high	medium	hard	-0.643869086
321	2	0	iran	5.5	subcutances	other days	medium	medium	hard	-0.928090082
321	3	0	iran	5.5	mascular	once a weak	high	medium	hard	-0.572088286
321	3	1	iran	2.5	subcutances	three times a weak	high	medium	easy	-0.646894171
321	4	0	iran	0.5	subcutances	three times a weak	medium	low	hard	-0.699689278
321	4	1	other	2.5	mascular	once a weak	high	low	hard	0.00383736
321	5	0	iran	0.5	mascular	once a weak	medium	low	easy	-0.303092094
321	5	1	other	2.5	subcutances	three times a weak	high	low	easy	-0.030902146
321	6	1	other	2.5	subcutances	three times a weak	high	low	easy	-0.030902146
321	6	0	other	0.5	subcutances	other days	high	medium	easy	-0.462940247
321	7	0	other	0.5	mascular	once a weak	medium	medium	hard	-0.900801637
321	7	1	other	5.5	subcutances	other days	medium	low	hard	-0.312098058
321	8	1	iran	2.5	mascular	once a weak	high	low	hard	-0.0957683
321	8	0	iran	5.5	subcutances	three times a weak	high	low	easy	-0.090441429
321	9	0	iran	2.5	subcutances	three times a weak	medium	low	hard	-0.617903819
321	9	1	other	0.5	subcutances	other days	high	medium	hard	-0.643869086
321	10	1	other	2.5	mascular	once a weak	high	low	hard	0.00383736
321	10	0	iran	5.5	mascular	once a weak	medium	medium	easy	-0.697626621
321	11	1	other	5.5	subcutances	other days	high	medium	easy	-0.34108841
321	11	0	iran	0.5	subcutances	three times a weak	medium	low	hard	-0.699689278
321	12	1	iran	5.5	subcutances	three times a weak	high	medium	hard	-0.787756632
321	12	0	other	2.5	subcutances	other days	medium	low	hard	-0.352164436
322	1	1	iran	5.5	mascular	once a weak	high	medium	hard	-0.572088286
322	1	0	iran	0.5	subcutances	three times a weak	high	medium	easy	-0.72867963
322	2	1	other	0.5	subcutances	other days	high	medium	hard	-0.643869086
322	2	0	iran	5.5	subcutances	other days	medium	medium	hard	-0.928090082
322	3	0	iran	5.5	mascular	once a weak	high	medium	hard	-0.572088286
322	3	1	iran	2.5	subcutances	three times a weak	high	medium	easy	-0.646894171
322	4	0	iran	0.5	subcutances	three times a weak	medium	low	hard	-0.699689278
322	4	1	other	2.5	mascular	once a weak	high	low	hard	0.00383736
322	5	0	iran	0.5	mascular	once a weak	medium	low	easy	-0.303092094
322	5	1	other	2.5	subcutances	three times a weak	high	low	easy	-0.030902146
322	6	1	other	2.5	subcutances	three times a weak	high	low	easy	-0.030902146
322	6	0	other	0.5	subcutances	other days	high	medium	easy	-0.462940247

322	7	0	other	0.5	mascular once a weak	medium	medium	hard	-0.900801637
322	7	1	other	5.5	subcutances other days	medium	low	hard	-0.312098058
322	8	0	iran	2.5	mascular once a weak	high	low	hard	-0.0957683
322	8	1	iran	5.5	subcutances three times a weak	high	low	easy	-0.090441429
322	9	1	iran	2.5	subcutances three times a weak	medium	low	hard	-0.617903819
322	9	0	other	0.5	subcutances other days	high	medium	hard	-0.643869086
322	10	0	other	2.5	mascular once a weak	high	low	hard	0.00383736
322	10	1	iran	5.5	mascular once a weak	medium	medium	easy	-0.697626621
322	11	1	other	5.5	subcutances other days	high	medium	easy	-0.34108841
322	11	0	iran	0.5	subcutances three times a weak	medium	low	hard	-0.699689278
322	12	0	iran	5.5	subcutances three times a weak	high	medium	hard	-0.787756632
322	12	1	other	2.5	subcutances other days	medium	low	hard	-0.352164436
323	1	0	iran	5.5	mascular once a weak	high	medium	hard	-0.572088286
323	1	1	iran	0.5	subcutances three times a weak	high	medium	easy	-0.72867963
323	2	1	other	0.5	subcutances other days	high	medium	hard	-0.643869086
323	2	0	iran	5.5	subcutances other days	medium	medium	hard	-0.928090082
323	3	0	iran	5.5	mascular once a weak	high	medium	hard	-0.572088286
323	3	1	iran	2.5	subcutances three times a weak	high	medium	easy	-0.646894171
323	4	0	iran	0.5	subcutances three times a weak	medium	low	hard	-0.699689278
323	4	1	other	2.5	mascular once a weak	high	low	hard	0.00383736
323	5	0	iran	0.5	mascular once a weak	medium	low	easy	-0.303092094
323	5	1	other	2.5	subcutances three times a weak	high	low	easy	-0.030902146
323	6	1	other	2.5	subcutances three times a weak	high	low	easy	-0.030902146
323	6	0	other	0.5	subcutances other days	high	medium	easy	-0.462940247
323	7	0	other	0.5	mascular once a weak	medium	medium	hard	-0.900801637
323	7	1	other	5.5	subcutances other days	medium	low	hard	-0.312098058
323	8	0	iran	2.5	mascular once a weak	high	low	hard	-0.0957683
323	8	1	iran	5.5	subcutances three times a weak	high	low	easy	-0.090441429
323	9	1	iran	2.5	subcutances three times a weak	medium	low	hard	-0.617903819
323	9	0	other	0.5	subcutances other days	high	medium	hard	-0.643869086
323	10	1	other	2.5	mascular once a weak	high	low	hard	0.00383736
323	10	0	iran	5.5	mascular once a weak	medium	medium	easy	-0.697626621
323	11	0	other	5.5	subcutances other days	high	medium	easy	-0.34108841
323	11	1	iran	0.5	subcutances three times a weak	medium	low	hard	-0.699689278

323	12	0	iran	5.5	subcutances three times a weak	high	medium	hard	-0.787756632
323	12	1	other	2.5	subcutances other days	medium	low	hard	-0.352164436
324	1	1	iran	5.5	mascular once a weak	high	medium	hard	-0.572088286
324	1	0	iran	0.5	subcutances three times a weak	high	medium	easy	-0.72867963
324	2	1	other	0.5	subcutances other days	high	medium	hard	-0.643869086
324	2	0	iran	5.5	subcutances other days	medium	medium	hard	-0.928090082
324	3	0	iran	5.5	mascular once a weak	high	medium	hard	-0.572088286
324	3	1	iran	2.5	subcutances three times a weak	high	medium	easy	-0.646894171
324	4	0	iran	0.5	subcutances three times a weak	medium	low	hard	-0.699689278
324	4	1	other	2.5	mascular once a weak	high	low	hard	0.00383736
324	5	1	iran	0.5	mascular once a weak	medium	low	easy	-0.303092094
324	5	0	other	2.5	subcutances three times a weak	high	low	easy	-0.030902146
324	6	0	other	2.5	subcutances three times a weak	high	low	easy	-0.030902146
324	6	1	other	0.5	subcutances other days	high	medium	easy	-0.462940247
324	7	0	other	0.5	mascular once a weak	medium	medium	hard	-0.900801637
324	7	1	other	5.5	subcutances other days	medium	low	hard	-0.312098058
324	8	1	iran	2.5	mascular once a weak	high	low	hard	-0.0957683
324	8	0	iran	5.5	subcutances three times a weak	high	low	easy	-0.090441429
324	9	1	iran	2.5	subcutances three times a weak	medium	low	hard	-0.617903819
324	9	0	other	0.5	subcutances other days	high	medium	hard	-0.643869086
324	10	1	other	2.5	mascular once a weak	high	low	hard	0.00383736
324	10	0	iran	5.5	mascular once a weak	medium	medium	easy	-0.697626621
324	11	0	other	5.5	subcutances other days	high	medium	easy	-0.34108841
324	11	1	iran	0.5	subcutances three times a weak	medium	low	hard	-0.699689278
324	12	0	iran	5.5	subcutances three times a weak	high	medium	hard	-0.787756632
324	12	1	other	2.5	subcutances other days	medium	low	hard	-0.352164436
325	1	1	iran	5.5	mascular once a weak	high	medium	hard	-0.572088286
325	1	0	iran	0.5	subcutances three times a weak	high	medium	easy	-0.72867963
325	2	0	other	0.5	subcutances other days	high	medium	hard	-0.643869086
325	2	1	iran	5.5	subcutances other days	medium	medium	hard	-0.928090082
325	3	1	iran	5.5	mascular once a weak	high	medium	hard	-0.572088286
325	3	0	iran	2.5	subcutances three times a weak	high	medium	easy	-0.646894171
325	4	0	iran	0.5	subcutances three times a weak	medium	low	hard	-0.699689278
325	4	1	other	2.5	mascular once a weak	high	low	hard	0.00383736

325	5	1	iran	0.5	mascular once a weak	medium	low	easy	-0.303092094
325	5	0	other	2.5	subcutances three times a weak	high	low	easy	-0.030902146
325	6	1	other	2.5	subcutances three times a weak	high	low	easy	-0.030902146
325	6	0	other	0.5	subcutances other days	high	medium	easy	-0.462940247
325	7	1	other	0.5	mascular once a weak	medium	medium	hard	-0.900801637
325	7	0	other	5.5	subcutances other days	medium	low	hard	-0.312098058
325	8	1	iran	2.5	mascular once a weak	high	low	hard	-0.0957683
325	8	0	iran	5.5	subcutances three times a weak	high	low	easy	-0.090441429
325	9	1	iran	2.5	subcutances three times a weak	medium	low	hard	-0.617903819
325	9	0	other	0.5	subcutances other days	high	medium	hard	-0.643869086
325	10	0	other	2.5	mascular once a weak	high	low	hard	0.00383736
325	10	1	iran	5.5	mascular once a weak	medium	medium	easy	-0.697626621
325	11	1	other	5.5	subcutances other days	high	medium	easy	-0.34108841
325	11	0	iran	0.5	subcutances three times a weak	medium	low	hard	-0.699689278
325	12	1	iran	5.5	subcutances three times a weak	high	medium	hard	-0.787756632
325	12	0	other	2.5	subcutances other days	medium	low	hard	-0.352164436
326	1	1	iran	5.5	mascular once a weak	high	medium	hard	-0.572088286
326	1	0	iran	0.5	subcutances three times a weak	high	medium	easy	-0.72867963
326	2	1	other	0.5	subcutances other days	high	medium	hard	-0.643869086
326	2	0	iran	5.5	subcutances other days	medium	medium	hard	-0.928090082
326	3	0	iran	5.5	mascular once a weak	high	medium	hard	-0.572088286
326	3	1	iran	2.5	subcutances three times a weak	high	medium	easy	-0.646894171
326	4	0	iran	0.5	subcutances three times a weak	medium	low	hard	-0.699689278
326	4	1	other	2.5	mascular once a weak	high	low	hard	0.00383736
326	5	0	iran	0.5	mascular once a weak	medium	low	easy	-0.303092094
326	5	1	other	2.5	subcutances three times a weak	high	low	easy	-0.030902146
326	6	0	other	2.5	subcutances three times a weak	high	low	easy	-0.030902146
326	6	1	other	0.5	subcutances other days	high	medium	easy	-0.462940247
326	7	0	other	0.5	mascular once a weak	medium	medium	hard	-0.900801637
326	7	1	other	5.5	subcutances other days	medium	low	hard	-0.312098058
326	8	1	iran	2.5	mascular once a weak	high	low	hard	-0.0957683
326	8	0	iran	5.5	subcutances three times a weak	high	low	easy	-0.090441429
326	9	0	iran	2.5	subcutances three times a weak	medium	low	hard	-0.617903819
326	9	1	other	0.5	subcutances other days	high	medium	hard	-0.643869086

326	10	1	other	2.5	mascular once a weak	high	low	hard	0.00383736
326	10	0	iran	5.5	mascular once a weak	medium	medium	easy	-0.697626621
326	11	1	other	5.5	subcutances other days	high	medium	easy	-0.34108841
326	11	0	iran	0.5	subcutances three times a weak	medium	low	hard	-0.699689278
326	12	1	iran	5.5	subcutances three times a weak	high	medium	hard	-0.787756632
326	12	0	other	2.5	subcutances other days	medium	low	hard	-0.352164436
327	1	0	iran	5.5	mascular once a weak	high	medium	hard	-0.572088286
327	1	1	iran	0.5	subcutances three times a weak	high	medium	easy	-0.72867963
327	2	1	other	0.5	subcutances other days	high	medium	hard	-0.643869086
327	2	0	iran	5.5	subcutances other days	medium	medium	hard	-0.928090082
327	3	0	iran	5.5	mascular once a weak	high	medium	hard	-0.572088286
327	3	1	iran	2.5	subcutances three times a weak	high	medium	easy	-0.646894171
327	4	0	iran	0.5	subcutances three times a weak	medium	low	hard	-0.699689278
327	4	1	other	2.5	mascular once a weak	high	low	hard	0.00383736
327	5	0	iran	0.5	mascular once a weak	medium	low	easy	-0.303092094
327	5	1	other	2.5	subcutances three times a weak	high	low	easy	-0.030902146
327	6	1	other	2.5	subcutances three times a weak	high	low	easy	-0.030902146
327	6	0	other	0.5	subcutances other days	high	medium	easy	-0.462940247
327	7	0	other	0.5	mascular once a weak	medium	medium	hard	-0.900801637
327	7	1	other	5.5	subcutances other days	medium	low	hard	-0.312098058
327	8	0	iran	2.5	mascular once a weak	high	low	hard	-0.0957683
327	8	1	iran	5.5	subcutances three times a weak	high	low	easy	-0.090441429
327	9	0	iran	2.5	subcutances three times a weak	medium	low	hard	-0.617903819
327	9	1	other	0.5	subcutances other days	high	medium	hard	-0.643869086
327	10	1	other	2.5	mascular once a weak	high	low	hard	0.00383736
327	10	0	iran	5.5	mascular once a weak	medium	medium	easy	-0.697626621
327	11	1	other	5.5	subcutances other days	high	medium	easy	-0.34108841
327	11	0	iran	0.5	subcutances three times a weak	medium	low	hard	-0.699689278
327	12	1	iran	5.5	subcutances three times a weak	high	medium	hard	-0.787756632
327	12	0	other	2.5	subcutances other days	medium	low	hard	-0.352164436
328	1	0	iran	5.5	mascular once a weak	high	medium	hard	-0.572088286
328	1	1	iran	0.5	subcutances three times a weak	high	medium	easy	-0.72867963
328	2	1	other	0.5	subcutances other days	high	medium	hard	-0.643869086
328	2	0	iran	5.5	subcutances other days	medium	medium	hard	-0.928090082

328	3	0	iran	5.5	mascular once a weak	high	medium	hard	-0.572088286
328	3	1	iran	2.5	subcutances three times a weak	high	medium	easy	-0.646894171
328	4	0	iran	0.5	subcutances three times a weak	medium	low	hard	-0.699689278
328	4	1	other	2.5	mascular once a weak	high	low	hard	0.00383736
328	5	0	iran	0.5	mascular once a weak	medium	low	easy	-0.303092094
328	5	1	other	2.5	subcutances three times a weak	high	low	easy	-0.030902146
328	6	1	other	2.5	subcutances three times a weak	high	low	easy	-0.030902146
328	6	0	other	0.5	subcutances other days	high	medium	easy	-0.462940247
328	7	0	other	0.5	mascular once a weak	medium	medium	hard	-0.900801637
328	7	1	other	5.5	subcutances other days	medium	low	hard	-0.312098058
328	8	0	iran	2.5	mascular once a weak	high	low	hard	-0.0957683
328	8	1	iran	5.5	subcutances three times a weak	high	low	easy	-0.090441429
328	9	0	iran	2.5	subcutances three times a weak	medium	low	hard	-0.617903819
328	9	1	other	0.5	subcutances other days	high	medium	hard	-0.643869086
328	10	1	other	2.5	mascular once a weak	high	low	hard	0.00383736
328	10	0	iran	5.5	mascular once a weak	medium	medium	easy	-0.697626621
328	11	1	other	5.5	subcutances other days	high	medium	easy	-0.34108841
328	11	0	iran	0.5	subcutances three times a weak	medium	low	hard	-0.699689278
328	12	1	iran	5.5	subcutances three times a weak	high	medium	hard	-0.787756632
328	12	0	other	2.5	subcutances other days	medium	low	hard	-0.352164436
329	1	1	iran	5.5	mascular once a weak	high	medium	hard	-0.572088286
329	1	0	iran	0.5	subcutances three times a weak	high	medium	easy	-0.72867963
329	2	0	other	0.5	subcutances other days	high	medium	hard	-0.643869086
329	2	1	iran	5.5	subcutances other days	medium	medium	hard	-0.928090082
329	3	0	iran	5.5	mascular once a weak	high	medium	hard	-0.572088286
329	3	1	iran	2.5	subcutances three times a weak	high	medium	easy	-0.646894171
329	4	0	iran	0.5	subcutances three times a weak	medium	low	hard	-0.699689278
329	4	1	other	2.5	mascular once a weak	high	low	hard	0.00383736
329	5	1	iran	0.5	mascular once a weak	medium	low	easy	-0.303092094
329	5	0	other	2.5	subcutances three times a weak	high	low	easy	-0.030902146
329	6	0	other	2.5	subcutances three times a weak	high	low	easy	-0.030902146
329	6	1	other	0.5	subcutances other days	high	medium	easy	-0.462940247
329	7	0	other	0.5	mascular once a weak	medium	medium	hard	-0.900801637
329	7	1	other	5.5	subcutances other days	medium	low	hard	-0.312098058

329	8	1	iran	2.5	mascular once a weak	high	low	hard	-0.0957683
329	8	0	iran	5.5	subcutances three times a weak	high	low	easy	-0.090441429
329	9	1	iran	2.5	subcutances three times a weak	medium	low	hard	-0.617903819
329	9	0	other	0.5	subcutances other days	high	medium	hard	-0.643869086
329	10	0	other	2.5	mascular once a weak	high	low	hard	0.00383736
329	10	1	iran	5.5	mascular once a weak	medium	medium	easy	-0.697626621
329	11	0	other	5.5	subcutances other days	high	medium	easy	-0.34108841
329	11	1	iran	0.5	subcutances three times a weak	medium	low	hard	-0.699689278
329	12	0	iran	5.5	subcutances three times a weak	high	medium	hard	-0.787756632
329	12	1	other	2.5	subcutances other days	medium	low	hard	-0.352164436
330	1	1	iran	5.5	mascular once a weak	high	medium	hard	-0.572088286
330	1	0	iran	0.5	subcutances three times a weak	high	medium	easy	-0.72867963
330	2	0	other	0.5	subcutances other days	high	medium	hard	-0.643869086
330	2	1	iran	5.5	subcutances other days	medium	medium	hard	-0.928090082
330	3	0	iran	5.5	mascular once a weak	high	medium	hard	-0.572088286
330	3	1	iran	2.5	subcutances three times a weak	high	medium	easy	-0.646894171
330	4	1	iran	0.5	subcutances three times a weak	medium	low	hard	-0.699689278
330	4	0	other	2.5	mascular once a weak	high	low	hard	0.00383736
330	5	1	iran	0.5	mascular once a weak	medium	low	easy	-0.303092094
330	5	0	other	2.5	subcutances three times a weak	high	low	easy	-0.030902146
330	6	0	other	2.5	subcutances three times a weak	high	low	easy	-0.030902146
330	6	1	other	0.5	subcutances other days	high	medium	easy	-0.462940247
330	7	0	other	0.5	mascular once a weak	medium	medium	hard	-0.900801637
330	7	0	other	5.5	subcutances other days	medium	low	hard	-0.312098058
330	8	1	iran	2.5	mascular once a weak	high	low	hard	-0.0957683
330	8	0	iran	5.5	subcutances three times a weak	high	low	easy	-0.090441429
330	9	1	iran	2.5	subcutances three times a weak	medium	low	hard	-0.617903819
330	9	0	other	0.5	subcutances other days	high	medium	hard	-0.643869086
330	10	0	other	2.5	mascular once a weak	high	low	hard	0.00383736
330	10	1	iran	5.5	mascular once a weak	medium	medium	easy	-0.697626621
330	11	0	other	5.5	subcutances other days	high	medium	easy	-0.34108841
330	11	1	iran	0.5	subcutances three times a weak	medium	low	hard	-0.699689278
330	12	0	iran	5.5	subcutances three times a weak	high	medium	hard	-0.787756632
330	12	1	other	2.5	subcutances other days	medium	low	hard	-0.352164436

331	1	1	iran	5.5	mascular once a weak	high	medium	hard	-0.572088286
331	1	0	iran	0.5	subcutances three times a weak	high	medium	easy	-0.72867963
331	2	0	other	0.5	subcutances other days	high	medium	hard	-0.643869086
331	2	1	iran	5.5	subcutances other days	medium	medium	hard	-0.928090082
331	3	0	iran	5.5	mascular once a weak	high	medium	hard	-0.572088286
331	3	1	iran	2.5	subcutances three times a weak	high	medium	easy	-0.646894171
331	4	1	iran	0.5	subcutances three times a weak	medium	low	hard	-0.699689278
331	4	0	other	2.5	mascular once a weak	high	low	hard	0.00383736
331	5	1	iran	0.5	mascular once a weak	medium	low	easy	-0.303092094
331	5	0	other	2.5	subcutances three times a weak	high	low	easy	-0.030902146
331	6	0	other	2.5	subcutances three times a weak	high	low	easy	-0.030902146
331	6	1	other	0.5	subcutances other days	high	medium	easy	-0.462940247
331	7	0	other	0.5	mascular once a weak	medium	medium	hard	-0.900801637
331	7	1	other	5.5	subcutances other days	medium	low	hard	-0.312098058
331	8	1	iran	2.5	mascular once a weak	high	low	hard	-0.0957683
331	8	0	iran	5.5	subcutances three times a weak	high	low	easy	-0.090441429
331	9	1	iran	2.5	subcutances three times a weak	medium	low	hard	-0.617903819
331	9	0	other	0.5	subcutances other days	high	medium	hard	-0.643869086
331	10	0	other	2.5	mascular once a weak	high	low	hard	0.00383736
331	10	1	iran	5.5	mascular once a weak	medium	medium	easy	-0.697626621
331	11	0	other	5.5	subcutances other days	high	medium	easy	-0.34108841
331	11	1	iran	0.5	subcutances three times a weak	medium	low	hard	-0.699689278
331	12	0	iran	5.5	subcutances three times a weak	high	medium	hard	-0.787756632
331	12	1	other	2.5	subcutances other days	medium	low	hard	-0.352164436
332	1	0	iran	5.5	mascular once a weak	high	medium	hard	-0.572088286
332	1	1	iran	0.5	subcutances three times a weak	high	medium	easy	-0.72867963
332	2	1	other	0.5	subcutances other days	high	medium	hard	-0.643869086
332	2	0	iran	5.5	subcutances other days	medium	medium	hard	-0.928090082
332	3	0	iran	5.5	mascular once a weak	high	medium	hard	-0.572088286
332	3	1	iran	2.5	subcutances three times a weak	high	medium	easy	-0.646894171
332	4	0	iran	0.5	subcutances three times a weak	medium	low	hard	-0.699689278
332	4	1	other	2.5	mascular once a weak	high	low	hard	0.00383736
332	5	0	iran	0.5	mascular once a weak	medium	low	easy	-0.303092094
332	5	1	other	2.5	subcutances three times a weak	high	low	easy	-0.030902146

332	6	1	other	2.5	subcutances three times a weak	high	low	easy	-0.030902146
332	6	0	other	0.5	subcutances other days	high	medium	easy	-0.462940247
332	7	0	other	0.5	mascular once a weak	medium	medium	hard	-0.900801637
332	7	1	other	5.5	subcutances other days	medium	low	hard	-0.312098058
332	8	0	iran	2.5	mascular once a weak	high	low	hard	-0.0957683
332	8	1	iran	5.5	subcutances three times a weak	high	low	easy	-0.090441429
332	9	0	iran	2.5	subcutances three times a weak	medium	low	hard	-0.617903819
332	9	1	other	0.5	subcutances other days	high	medium	hard	-0.643869086
332	10	1	other	2.5	mascular once a weak	high	low	hard	0.00383736
332	10	0	iran	5.5	mascular once a weak	medium	medium	easy	-0.697626621
332	11	1	other	5.5	subcutances other days	high	medium	easy	-0.34108841
332	11	0	iran	0.5	subcutances three times a weak	medium	low	hard	-0.699689278
332	12	1	iran	5.5	subcutances three times a weak	high	medium	hard	-0.787756632
332	12	0	other	2.5	subcutances other days	medium	low	hard	-0.352164436
333	1	0	iran	5.5	mascular once a weak	high	medium	hard	-0.572088286
333	1	1	iran	0.5	subcutances three times a weak	high	medium	easy	-0.72867963
333	2	1	other	0.5	subcutances other days	high	medium	hard	-0.643869086
333	2	0	iran	5.5	subcutances other days	medium	medium	hard	-0.928090082
333	3	0	iran	5.5	mascular once a weak	high	medium	hard	-0.572088286
333	3	1	iran	2.5	subcutances three times a weak	high	medium	easy	-0.646894171
333	4	1	iran	0.5	subcutances three times a weak	medium	low	hard	-0.699689278
333	4	0	other	2.5	mascular once a weak	high	low	hard	0.00383736
333	5	1	iran	0.5	mascular once a weak	medium	low	easy	-0.303092094
333	5	0	other	2.5	subcutances three times a weak	high	low	easy	-0.030902146
333	6	1	other	2.5	subcutances three times a weak	high	low	easy	-0.030902146
333	6	0	other	0.5	subcutances other days	high	medium	easy	-0.462940247
333	7	1	other	0.5	mascular once a weak	medium	medium	hard	-0.900801637
333	7	0	other	5.5	subcutances other days	medium	low	hard	-0.312098058
333	8	0	iran	2.5	mascular once a weak	high	low	hard	-0.0957683
333	8	1	iran	5.5	subcutances three times a weak	high	low	easy	-0.090441429
333	9	0	iran	2.5	subcutances three times a weak	medium	low	hard	-0.617903819
333	9	1	other	0.5	subcutances other days	high	medium	hard	-0.643869086
333	10	1	other	2.5	mascular once a weak	high	low	hard	0.00383736
333	10	0	iran	5.5	mascular once a weak	medium	medium	easy	-0.697626621

333	11	1	other	5.5	subcutances other days	high	medium	easy	-0.34108841
333	11	0	iran	0.5	subcutances three times a weak	medium	low	hard	-0.699689278
333	12	0	iran	5.5	subcutances three times a weak	high	medium	hard	-0.787756632
333	12	1	other	2.5	subcutances other days	medium	low	hard	-0.352164436
334	1	1	iran	5.5	mascular once a weak	high	medium	hard	-0.572088286
334	1	0	iran	0.5	subcutances three times a weak	high	medium	easy	-0.72867963
334	2	0	other	0.5	subcutances other days	high	medium	hard	-0.643869086
334	2	1	iran	5.5	subcutances other days	medium	medium	hard	-0.928090082
334	3	0	iran	5.5	mascular once a weak	high	medium	hard	-0.572088286
334	3	1	iran	2.5	subcutances three times a weak	high	medium	easy	-0.646894171
334	4	1	iran	0.5	subcutances three times a weak	medium	low	hard	-0.699689278
334	4	0	other	2.5	mascular once a weak	high	low	hard	0.00383736
334	5	1	iran	0.5	mascular once a weak	medium	low	easy	-0.303092094
334	5	0	other	2.5	subcutances three times a weak	high	low	easy	-0.030902146
334	6	1	other	2.5	subcutances three times a weak	high	low	easy	-0.030902146
334	6	0	other	0.5	subcutances other days	high	medium	easy	-0.462940247
334	7	0	other	0.5	mascular once a weak	medium	medium	hard	-0.900801637
334	7	1	other	5.5	subcutances other days	medium	low	hard	-0.312098058
334	8	1	iran	2.5	mascular once a weak	high	low	hard	-0.0957683
334	8	0	iran	5.5	subcutances three times a weak	high	low	easy	-0.090441429
334	9	1	iran	2.5	subcutances three times a weak	medium	low	hard	-0.617903819
334	9	0	other	0.5	subcutances other days	high	medium	hard	-0.643869086
334	10	0	other	2.5	mascular once a weak	high	low	hard	0.00383736
334	10	1	iran	5.5	mascular once a weak	medium	medium	easy	-0.697626621
334	11	1	other	5.5	subcutances other days	high	medium	easy	-0.34108841
334	11	0	iran	0.5	subcutances three times a weak	medium	low	hard	-0.699689278
334	12	0	iran	5.5	subcutances three times a weak	high	medium	hard	-0.787756632
334	12	1	other	2.5	subcutances other days	medium	low	hard	-0.352164436
335	1	0	iran	5.5	mascular once a weak	high	medium	hard	-0.572088286
335	1	0	iran	0.5	subcutances three times a weak	high	medium	easy	-0.72867963
335	2	1	other	0.5	subcutances other days	high	medium	hard	-0.643869086
335	2	0	iran	5.5	subcutances other days	medium	medium	hard	-0.928090082
335	3	0	iran	5.5	mascular once a weak	high	medium	hard	-0.572088286
335	3	0	iran	2.5	subcutances three times a weak	high	medium	easy	-0.646894171

335	4	0	iran	0.5	subcutances three times a weak	medium	low	hard	-0.699689278
335	4	1	other	2.5	mascular once a weak	high	low	hard	0.00383736
335	5	0	iran	0.5	mascular once a weak	medium	low	easy	-0.303092094
335	5	1	other	2.5	subcutances three times a weak	high	low	easy	-0.030902146
335	6	1	other	2.5	subcutances three times a weak	high	low	easy	-0.030902146
335	6	0	other	0.5	subcutances other days	high	medium	easy	-0.462940247
335	7	0	other	0.5	mascular once a weak	medium	medium	hard	-0.900801637
335	7	1	other	5.5	subcutances other days	medium	low	hard	-0.312098058
335	8	0	iran	2.5	mascular once a weak	high	low	hard	-0.0957683
335	8	0	iran	5.5	subcutances three times a weak	high	low	easy	-0.090441429
335	9	0	iran	2.5	subcutances three times a weak	medium	low	hard	-0.617903819
335	9	1	other	0.5	subcutances other days	high	medium	hard	-0.643869086
335	10	1	other	2.5	mascular once a weak	high	low	hard	0.00383736
335	10	0	iran	5.5	mascular once a weak	medium	medium	easy	-0.697626621
335	11	1	other	5.5	subcutances other days	high	medium	easy	-0.34108841
335	11	0	iran	0.5	subcutances three times a weak	medium	low	hard	-0.699689278
335	12	0	iran	5.5	subcutances three times a weak	high	medium	hard	-0.787756632
335	12	1	other	2.5	subcutances other days	medium	low	hard	-0.352164436
336	1	1	iran	5.5	mascular once a weak	high	medium	hard	-0.572088286
336	1	0	iran	0.5	subcutances three times a weak	high	medium	easy	-0.72867963
336	2	1	other	0.5	subcutances other days	high	medium	hard	-0.643869086
336	2	0	iran	5.5	subcutances other days	medium	medium	hard	-0.928090082
336	3	0	iran	5.5	mascular once a weak	high	medium	hard	-0.572088286
336	3	1	iran	2.5	subcutances three times a weak	high	medium	easy	-0.646894171
336	4	0	iran	0.5	subcutances three times a weak	medium	low	hard	-0.699689278
336	4	1	other	2.5	mascular once a weak	high	low	hard	0.00383736
336	5	0	iran	0.5	mascular once a weak	medium	low	easy	-0.303092094
336	5	1	other	2.5	subcutances three times a weak	high	low	easy	-0.030902146
336	6	1	other	2.5	subcutances three times a weak	high	low	easy	-0.030902146
336	6	0	other	0.5	subcutances other days	high	medium	easy	-0.462940247
336	7	0	other	0.5	mascular once a weak	medium	medium	hard	-0.900801637
336	7	1	other	5.5	subcutances other days	medium	low	hard	-0.312098058
336	8	0	iran	2.5	mascular once a weak	high	low	hard	-0.0957683
336	8	1	iran	5.5	subcutances three times a weak	high	low	easy	-0.090441429

336	9	0	iran	2.5	subcutances three times a weak	medium	low	hard	-0.617903819
336	9	1	other	0.5	subcutances other days	high	medium	hard	-0.643869086
336	10	1	other	2.5	mascular once a weak	high	low	hard	0.00383736
336	10	0	iran	5.5	mascular once a weak	medium	medium	easy	-0.697626621
336	11	1	other	5.5	subcutances other days	high	medium	easy	-0.34108841
336	11	0	iran	0.5	subcutances three times a weak	medium	low	hard	-0.699689278
336	12	0	iran	5.5	subcutances three times a weak	high	medium	hard	-0.787756632
336	12	1	other	2.5	subcutances other days	medium	low	hard	-0.352164436
337	1	1	iran	5.5	mascular once a weak	high	medium	hard	-0.572088286
337	1	0	iran	0.5	subcutances three times a weak	high	medium	easy	-0.72867963
337	2	0	other	0.5	subcutances other days	high	medium	hard	-0.643869086
337	2	1	iran	5.5	subcutances other days	medium	medium	hard	-0.928090082
337	3	0	iran	5.5	mascular once a weak	high	medium	hard	-0.572088286
337	3	1	iran	2.5	subcutances three times a weak	high	medium	easy	-0.646894171
337	4	1	iran	0.5	subcutances three times a weak	medium	low	hard	-0.699689278
337	4	0	other	2.5	mascular once a weak	high	low	hard	0.00383736
337	5	0	iran	0.5	mascular once a weak	medium	low	easy	-0.303092094
337	5	1	other	2.5	subcutances three times a weak	high	low	easy	-0.030902146
337	6	0	other	2.5	subcutances three times a weak	high	low	easy	-0.030902146
337	6	1	other	0.5	subcutances other days	high	medium	easy	-0.462940247
337	7	0	other	0.5	mascular once a weak	medium	medium	hard	-0.900801637
337	7	1	other	5.5	subcutances other days	medium	low	hard	-0.312098058
337	8	1	iran	2.5	mascular once a weak	high	low	hard	-0.0957683
337	8	0	iran	5.5	subcutances three times a weak	high	low	easy	-0.090441429
337	9	1	iran	2.5	subcutances three times a weak	medium	low	hard	-0.617903819
337	9	0	other	0.5	subcutances other days	high	medium	hard	-0.643869086
337	10	1	other	2.5	mascular once a weak	high	low	hard	0.00383736
337	10	0	iran	5.5	mascular once a weak	medium	medium	easy	-0.697626621
337	11	0	other	5.5	subcutances other days	high	medium	easy	-0.34108841
337	11	1	iran	0.5	subcutances three times a weak	medium	low	hard	-0.699689278
337	12	0	iran	5.5	subcutances three times a weak	high	medium	hard	-0.787756632
337	12	1	other	2.5	subcutances other days	medium	low	hard	-0.352164436
338	1	0	iran	5.5	mascular once a weak	high	medium	hard	-0.572088286
338	1	1	iran	0.5	subcutances three times a weak	high	medium	easy	-0.72867963

338	2	1	other	0.5	subcutances other days	high	medium	hard	-0.643869086
338	2	0	iran	5.5	subcutances other days	medium	medium	hard	-0.928090082
338	3	0	iran	5.5	mascular once a weak	high	medium	hard	-0.572088286
338	3	1	iran	2.5	subcutances three times a weak	high	medium	easy	-0.646894171
338	4	0	iran	0.5	subcutances three times a weak	medium	low	hard	-0.699689278
338	4	1	other	2.5	mascular once a weak	high	low	hard	0.00383736
338	5	0	iran	0.5	mascular once a weak	medium	low	easy	-0.303092094
338	5	1	other	2.5	subcutances three times a weak	high	low	easy	-0.030902146
338	6	1	other	2.5	subcutances three times a weak	high	low	easy	-0.030902146
338	6	0	other	0.5	subcutances other days	high	medium	easy	-0.462940247
338	7	0	other	0.5	mascular once a weak	medium	medium	hard	-0.900801637
338	7	1	other	5.5	subcutances other days	medium	low	hard	-0.312098058
338	8	0	iran	2.5	mascular once a weak	high	low	hard	-0.0957683
338	8	1	iran	5.5	subcutances three times a weak	high	low	easy	-0.090441429
338	9	1	iran	2.5	subcutances three times a weak	medium	low	hard	-0.617903819
338	9	0	other	0.5	subcutances other days	high	medium	hard	-0.643869086
338	10	1	other	2.5	mascular once a weak	high	low	hard	0.00383736
338	10	0	iran	5.5	mascular once a weak	medium	medium	easy	-0.697626621
338	11	0	other	5.5	subcutances other days	high	medium	easy	-0.34108841
338	11	1	iran	0.5	subcutances three times a weak	medium	low	hard	-0.699689278
338	12	0	iran	5.5	subcutances three times a weak	high	medium	hard	-0.787756632
338	12	1	other	2.5	subcutances other days	medium	low	hard	-0.352164436
339	1	1	iran	5.5	mascular once a weak	high	medium	hard	-0.572088286
339	1	0	iran	0.5	subcutances three times a weak	high	medium	easy	-0.72867963
339	2	1	other	0.5	subcutances other days	high	medium	hard	-0.643869086
339	2	0	iran	5.5	subcutances other days	medium	medium	hard	-0.928090082
339	3	0	iran	5.5	mascular once a weak	high	medium	hard	-0.572088286
339	3	1	iran	2.5	subcutances three times a weak	high	medium	easy	-0.646894171
339	4	0	iran	0.5	subcutances three times a weak	medium	low	hard	-0.699689278
339	4	1	other	2.5	mascular once a weak	high	low	hard	0.00383736
339	5	0	iran	0.5	mascular once a weak	medium	low	easy	-0.303092094
339	5	1	other	2.5	subcutances three times a weak	high	low	easy	-0.030902146
339	6	0	other	2.5	subcutances three times a weak	high	low	easy	-0.030902146
339	6	1	other	0.5	subcutances other days	high	medium	easy	-0.462940247

339	7	0	other	0.5	mascular once a weak	medium	medium	hard	-0.900801637
339	7	1	other	5.5	subcutances other days	medium	low	hard	-0.312098058
339	8	1	iran	2.5	mascular once a weak	high	low	hard	-0.0957683
339	8	0	iran	5.5	subcutances three times a weak	high	low	easy	-0.090441429
339	9	0	iran	2.5	subcutances three times a weak	medium	low	hard	-0.617903819
339	9	1	other	0.5	subcutances other days	high	medium	hard	-0.643869086
339	10	1	other	2.5	mascular once a weak	high	low	hard	0.00383736
339	10	0	iran	5.5	mascular once a weak	medium	medium	easy	-0.697626621
339	11	1	other	5.5	subcutances other days	high	medium	easy	-0.34108841
339	11	0	iran	0.5	subcutances three times a weak	medium	low	hard	-0.699689278
339	12	1	iran	5.5	subcutances three times a weak	high	medium	hard	-0.787756632
339	12	0	other	2.5	subcutances other days	medium	low	hard	-0.352164436
340	1	0	iran	5.5	mascular once a weak	high	medium	hard	-0.572088286
340	1	1	iran	0.5	subcutances three times a weak	high	medium	easy	-0.72867963
340	2	1	other	0.5	subcutances other days	high	medium	hard	-0.643869086
340	2	0	iran	5.5	subcutances other days	medium	medium	hard	-0.928090082
340	3	0	iran	5.5	mascular once a weak	high	medium	hard	-0.572088286
340	3	1	iran	2.5	subcutances three times a weak	high	medium	easy	-0.646894171
340	4	0	iran	0.5	subcutances three times a weak	medium	low	hard	-0.699689278
340	4	1	other	2.5	mascular once a weak	high	low	hard	0.00383736
340	5	0	iran	0.5	mascular once a weak	medium	low	easy	-0.303092094
340	5	1	other	2.5	subcutances three times a weak	high	low	easy	-0.030902146
340	6	1	other	2.5	subcutances three times a weak	high	low	easy	-0.030902146
340	6	0	other	0.5	subcutances other days	high	medium	easy	-0.462940247
340	7	0	other	0.5	mascular once a weak	medium	medium	hard	-0.900801637
340	7	1	other	5.5	subcutances other days	medium	low	hard	-0.312098058
340	8	0	iran	2.5	mascular once a weak	high	low	hard	-0.0957683
340	8	1	iran	5.5	subcutances three times a weak	high	low	easy	-0.090441429
340	9	1	iran	2.5	subcutances three times a weak	medium	low	hard	-0.617903819
340	9	0	other	0.5	subcutances other days	high	medium	hard	-0.643869086
340	10	1	other	2.5	mascular once a weak	high	low	hard	0.00383736
340	10	0	iran	5.5	mascular once a weak	medium	medium	easy	-0.697626621
340	11	1	other	5.5	subcutances other days	high	medium	easy	-0.34108841
340	11	0	iran	0.5	subcutances three times a weak	medium	low	hard	-0.699689278

340	12	0	iran	5.5	subcutances three times a weak	high	medium	hard	-0.787756632
340	12	1	other	2.5	subcutances other days	medium	low	hard	-0.352164436
341	1	0	iran	5.5	mascular once a weak	high	medium	hard	-0.572088286
341	1	1	iran	0.5	subcutances three times a weak	high	medium	easy	-0.72867963
341	2	1	other	0.5	subcutances other days	high	medium	hard	-0.643869086
341	2	0	iran	5.5	subcutances other days	medium	medium	hard	-0.928090082
341	3	0	iran	5.5	mascular once a weak	high	medium	hard	-0.572088286
341	3	1	iran	2.5	subcutances three times a weak	high	medium	easy	-0.646894171
341	4	0	iran	0.5	subcutances three times a weak	medium	low	hard	-0.699689278
341	4	1	other	2.5	mascular once a weak	high	low	hard	0.00383736
341	5	0	iran	0.5	mascular once a weak	medium	low	easy	-0.303092094
341	5	1	other	2.5	subcutances three times a weak	high	low	easy	-0.030902146
341	6	1	other	2.5	subcutances three times a weak	high	low	easy	-0.030902146
341	6	0	other	0.5	subcutances other days	high	medium	easy	-0.462940247
341	7	0	other	0.5	mascular once a weak	medium	medium	hard	-0.900801637
341	7	1	other	5.5	subcutances other days	medium	low	hard	-0.312098058
341	8	0	iran	2.5	mascular once a weak	high	low	hard	-0.0957683
341	8	1	iran	5.5	subcutances three times a weak	high	low	easy	-0.090441429
341	9	1	iran	2.5	subcutances three times a weak	medium	low	hard	-0.617903819
341	9	0	other	0.5	subcutances other days	high	medium	hard	-0.643869086
341	10	1	other	2.5	mascular once a weak	high	low	hard	0.00383736
341	10	0	iran	5.5	mascular once a weak	medium	medium	easy	-0.697626621
341	11	1	other	5.5	subcutances other days	high	medium	easy	-0.34108841
341	11	0	iran	0.5	subcutances three times a weak	medium	low	hard	-0.699689278
341	12	0	iran	5.5	subcutances three times a weak	high	medium	hard	-0.787756632
341	12	1	other	2.5	subcutances other days	medium	low	hard	-0.352164436
342	1	1	iran	5.5	mascular once a weak	high	medium	hard	-0.572088286
342	1	0	iran	0.5	subcutances three times a weak	high	medium	easy	-0.72867963
342	2	0	other	0.5	subcutances other days	high	medium	hard	-0.643869086
342	2	1	iran	5.5	subcutances other days	medium	medium	hard	-0.928090082
342	3	0	iran	5.5	mascular once a weak	high	medium	hard	-0.572088286
342	3	1	iran	2.5	subcutances three times a weak	high	medium	easy	-0.646894171
342	4	1	iran	0.5	subcutances three times a weak	medium	low	hard	-0.699689278
342	4	0	other	2.5	mascular once a weak	high	low	hard	0.00383736

342	5	1	iran	0.5	mascular once a weak	medium	low	easy	-0.303092094
342	5	0	other	2.5	subcutances three times a weak	high	low	easy	-0.030902146
342	6	0	other	2.5	subcutances three times a weak	high	low	easy	-0.030902146
342	6	1	other	0.5	subcutances other days	high	medium	easy	-0.462940247
342	7	0	other	0.5	mascular once a weak	medium	medium	hard	-0.900801637
342	7	0	other	5.5	subcutances other days	medium	low	hard	-0.312098058
342	8	1	iran	2.5	mascular once a weak	high	low	hard	-0.0957683
342	8	0	iran	5.5	subcutances three times a weak	high	low	easy	-0.090441429
342	9	1	iran	2.5	subcutances three times a weak	medium	low	hard	-0.617903819
342	9	0	other	0.5	subcutances other days	high	medium	hard	-0.643869086
342	10	0	other	2.5	mascular once a weak	high	low	hard	0.00383736
342	10	1	iran	5.5	mascular once a weak	medium	medium	easy	-0.697626621
342	11	0	other	5.5	subcutances other days	high	medium	easy	-0.34108841
342	11	1	iran	0.5	subcutances three times a weak	medium	low	hard	-0.699689278
342	12	0	iran	5.5	subcutances three times a weak	high	medium	hard	-0.787756632
342	12	1	other	2.5	subcutances other days	medium	low	hard	-0.352164436
343	1	1	iran	5.5	mascular once a weak	high	medium	hard	-0.572088286
343	1	0	iran	0.5	subcutances three times a weak	high	medium	easy	-0.72867963
343	2	0	other	0.5	subcutances other days	high	medium	hard	-0.643869086
343	2	1	iran	5.5	subcutances other days	medium	medium	hard	-0.928090082
343	3	1	iran	5.5	mascular once a weak	high	medium	hard	-0.572088286
343	3	0	iran	2.5	subcutances three times a weak	high	medium	easy	-0.646894171
343	4	0	iran	0.5	subcutances three times a weak	medium	low	hard	-0.699689278
343	4	1	other	2.5	mascular once a weak	high	low	hard	0.00383736
343	5	1	iran	0.5	mascular once a weak	medium	low	easy	-0.303092094
343	5	0	other	2.5	subcutances three times a weak	high	low	easy	-0.030902146
343	6	0	other	2.5	subcutances three times a weak	high	low	easy	-0.030902146
343	6	1	other	0.5	subcutances other days	high	medium	easy	-0.462940247
343	7	1	other	0.5	mascular once a weak	medium	medium	hard	-0.900801637
343	7	0	other	5.5	subcutances other days	medium	low	hard	-0.312098058
343	8	1	iran	2.5	mascular once a weak	high	low	hard	-0.0957683
343	8	0	iran	5.5	subcutances three times a weak	high	low	easy	-0.090441429
343	9	1	iran	2.5	subcutances three times a weak	medium	low	hard	-0.617903819
343	9	0	other	0.5	subcutances other days	high	medium	hard	-0.643869086

343	10	0	other	2.5	mascular once a weak	high	low	hard	0.00383736
343	10	1	iran	5.5	mascular once a weak	medium	medium	easy	-0.697626621
343	11	1	other	5.5	subcutances other days	high	medium	easy	-0.34108841
343	11	0	iran	0.5	subcutances three times a weak	medium	low	hard	-0.699689278
343	12	0	iran	5.5	subcutances three times a weak	high	medium	hard	-0.787756632
343	12	1	other	2.5	subcutances other days	medium	low	hard	-0.352164436
344	1	0	iran	5.5	mascular once a weak	high	medium	hard	-0.572088286
344	1	1	iran	0.5	subcutances three times a weak	high	medium	easy	-0.72867963
344	2	1	other	0.5	subcutances other days	high	medium	hard	-0.643869086
344	2	0	iran	5.5	subcutances other days	medium	medium	hard	-0.928090082
344	3	0	iran	5.5	mascular once a weak	high	medium	hard	-0.572088286
344	3	1	iran	2.5	subcutances three times a weak	high	medium	easy	-0.646894171
344	4	0	iran	0.5	subcutances three times a weak	medium	low	hard	-0.699689278
344	4	1	other	2.5	mascular once a weak	high	low	hard	0.00383736
344	5	0	iran	0.5	mascular once a weak	medium	low	easy	-0.303092094
344	5	1	other	2.5	subcutances three times a weak	high	low	easy	-0.030902146
344	6	1	other	2.5	subcutances three times a weak	high	low	easy	-0.030902146
344	6	0	other	0.5	subcutances other days	high	medium	easy	-0.462940247
344	7	0	other	0.5	mascular once a weak	medium	medium	hard	-0.900801637
344	7	1	other	5.5	subcutances other days	medium	low	hard	-0.312098058
344	8	0	iran	2.5	mascular once a weak	high	low	hard	-0.0957683
344	8	1	iran	5.5	subcutances three times a weak	high	low	easy	-0.090441429
344	9	1	iran	2.5	subcutances three times a weak	medium	low	hard	-0.617903819
344	9	0	other	0.5	subcutances other days	high	medium	hard	-0.643869086
344	10	1	other	2.5	mascular once a weak	high	low	hard	0.00383736
344	10	0	iran	5.5	mascular once a weak	medium	medium	easy	-0.697626621
344	11	0	other	5.5	subcutances other days	high	medium	easy	-0.34108841
344	11	1	iran	0.5	subcutances three times a weak	medium	low	hard	-0.699689278
344	12	0	iran	5.5	subcutances three times a weak	high	medium	hard	-0.787756632
344	12	1	other	2.5	subcutances other days	medium	low	hard	-0.352164436
345	1	0	iran	5.5	mascular once a weak	high	medium	hard	-0.572088286
345	1	1	iran	0.5	subcutances three times a weak	high	medium	easy	-0.72867963
345	2	1	other	0.5	subcutances other days	high	medium	hard	-0.643869086
345	2	0	iran	5.5	subcutances other days	medium	medium	hard	-0.928090082

345	3	1	iran	5.5	mascular once a weak	high	medium	hard	-0.572088286
345	3	0	iran	2.5	subcutances three times a weak	high	medium	easy	-0.646894171
345	4	0	iran	0.5	subcutances three times a weak	medium	low	hard	-0.699689278
345	4	1	other	2.5	mascular once a weak	high	low	hard	0.00383736
345	5	0	iran	0.5	mascular once a weak	medium	low	easy	-0.303092094
345	5	1	other	2.5	subcutances three times a weak	high	low	easy	-0.030902146
345	6	1	other	2.5	subcutances three times a weak	high	low	easy	-0.030902146
345	6	0	other	0.5	subcutances other days	high	medium	easy	-0.462940247
345	7	0	other	0.5	mascular once a weak	medium	medium	hard	-0.900801637
345	7	1	other	5.5	subcutances other days	medium	low	hard	-0.312098058
345	8	0	iran	2.5	mascular once a weak	high	low	hard	-0.0957683
345	8	1	iran	5.5	subcutances three times a weak	high	low	easy	-0.090441429
345	9	1	iran	2.5	subcutances three times a weak	medium	low	hard	-0.617903819
345	9	0	other	0.5	subcutances other days	high	medium	hard	-0.643869086
345	10	1	other	2.5	mascular once a weak	high	low	hard	0.00383736
345	10	0	iran	5.5	mascular once a weak	medium	medium	easy	-0.697626621
345	11	1	other	5.5	subcutances other days	high	medium	easy	-0.34108841
345	11	0	iran	0.5	subcutances three times a weak	medium	low	hard	-0.699689278
345	12	0	iran	5.5	subcutances three times a weak	high	medium	hard	-0.787756632
345	12	1	other	2.5	subcutances other days	medium	low	hard	-0.352164436
346	1	0	iran	5.5	mascular once a weak	high	medium	hard	-0.572088286
346	1	1	iran	0.5	subcutances three times a weak	high	medium	easy	-0.72867963
346	2	1	other	0.5	subcutances other days	high	medium	hard	-0.643869086
346	2	0	iran	5.5	subcutances other days	medium	medium	hard	-0.928090082
346	3	0	iran	5.5	mascular once a weak	high	medium	hard	-0.572088286
346	3	1	iran	2.5	subcutances three times a weak	high	medium	easy	-0.646894171
346	4	0	iran	0.5	subcutances three times a weak	medium	low	hard	-0.699689278
346	4	1	other	2.5	mascular once a weak	high	low	hard	0.00383736
346	5	0	iran	0.5	mascular once a weak	medium	low	easy	-0.303092094
346	5	1	other	2.5	subcutances three times a weak	high	low	easy	-0.030902146
346	6	1	other	2.5	subcutances three times a weak	high	low	easy	-0.030902146
346	6	0	other	0.5	subcutances other days	high	medium	easy	-0.462940247
346	7	0	other	0.5	mascular once a weak	medium	medium	hard	-0.900801637
346	7	1	other	5.5	subcutances other days	medium	low	hard	-0.312098058

346	8	0	iran	2.5	mascular once a weak	high	low	hard	-0.0957683
346	8	1	iran	5.5	subcutances three times a weak	high	low	easy	-0.090441429
346	9	1	iran	2.5	subcutances three times a weak	medium	low	hard	-0.617903819
346	9	0	other	0.5	subcutances other days	high	medium	hard	-0.643869086
346	10	1	other	2.5	mascular once a weak	high	low	hard	0.00383736
346	10	0	iran	5.5	mascular once a weak	medium	medium	easy	-0.697626621
346	11	1	other	5.5	subcutances other days	high	medium	easy	-0.34108841
346	11	0	iran	0.5	subcutances three times a weak	medium	low	hard	-0.699689278
346	12	1	iran	5.5	subcutances three times a weak	high	medium	hard	-0.787756632
346	12	0	other	2.5	subcutances other days	medium	low	hard	-0.352164436
347	1	0	iran	5.5	mascular once a weak	high	medium	hard	-0.572088286
347	1	1	iran	0.5	subcutances three times a weak	high	medium	easy	-0.72867963
347	2	0	other	0.5	subcutances other days	high	medium	hard	-0.643869086
347	2	1	iran	5.5	subcutances other days	medium	medium	hard	-0.928090082
347	3	0	iran	5.5	mascular once a weak	high	medium	hard	-0.572088286
347	3	1	iran	2.5	subcutances three times a weak	high	medium	easy	-0.646894171
347	4	1	iran	0.5	subcutances three times a weak	medium	low	hard	-0.699689278
347	4	0	other	2.5	mascular once a weak	high	low	hard	0.00383736
347	5	0	iran	0.5	mascular once a weak	medium	low	easy	-0.303092094
347	5	1	other	2.5	subcutances three times a weak	high	low	easy	-0.030902146
347	6	0	other	2.5	subcutances three times a weak	high	low	easy	-0.030902146
347	6	1	other	0.5	subcutances other days	high	medium	easy	-0.462940247
347	7	0	other	0.5	mascular once a weak	medium	medium	hard	-0.900801637
347	7	1	other	5.5	subcutances other days	medium	low	hard	-0.312098058
347	8	0	iran	2.5	mascular once a weak	high	low	hard	-0.0957683
347	8	1	iran	5.5	subcutances three times a weak	high	low	easy	-0.090441429
347	9	1	iran	2.5	subcutances three times a weak	medium	low	hard	-0.617903819
347	9	0	other	0.5	subcutances other days	high	medium	hard	-0.643869086
347	10	1	other	2.5	mascular once a weak	high	low	hard	0.00383736
347	10	0	iran	5.5	mascular once a weak	medium	medium	easy	-0.697626621
347	11	0	other	5.5	subcutances other days	high	medium	easy	-0.34108841
347	11	1	iran	0.5	subcutances three times a weak	medium	low	hard	-0.699689278
347	12	1	iran	5.5	subcutances three times a weak	high	medium	hard	-0.787756632
347	12	0	other	2.5	subcutances other days	medium	low	hard	-0.352164436

348	1	0	iran	5.5	mascular once a weak	high	medium	hard	-0.572088286
348	1	1	iran	0.5	subcutances three times a weak	high	medium	easy	-0.72867963
348	2	0	other	0.5	subcutances other days	high	medium	hard	-0.643869086
348	2	1	iran	5.5	subcutances other days	medium	medium	hard	-0.928090082
348	3	0	iran	5.5	mascular once a weak	high	medium	hard	-0.572088286
348	3	1	iran	2.5	subcutances three times a weak	high	medium	easy	-0.646894171
348	4	1	iran	0.5	subcutances three times a weak	medium	low	hard	-0.699689278
348	4	0	other	2.5	mascular once a weak	high	low	hard	0.00383736
348	5	1	iran	0.5	mascular once a weak	medium	low	easy	-0.303092094
348	5	0	other	2.5	subcutances three times a weak	high	low	easy	-0.030902146
348	6	0	other	2.5	subcutances three times a weak	high	low	easy	-0.030902146
348	6	1	other	0.5	subcutances other days	high	medium	easy	-0.462940247
348	7	0	other	0.5	mascular once a weak	medium	medium	hard	-0.900801637
348	7	1	other	5.5	subcutances other days	medium	low	hard	-0.312098058
348	8	1	iran	2.5	mascular once a weak	high	low	hard	-0.0957683
348	8	0	iran	5.5	subcutances three times a weak	high	low	easy	-0.090441429
348	9	1	iran	2.5	subcutances three times a weak	medium	low	hard	-0.617903819
348	9	0	other	0.5	subcutances other days	high	medium	hard	-0.643869086
348	10	0	other	2.5	mascular once a weak	high	low	hard	0.00383736
348	10	1	iran	5.5	mascular once a weak	medium	medium	easy	-0.697626621
348	11	0	other	5.5	subcutances other days	high	medium	easy	-0.34108841
348	11	1	iran	0.5	subcutances three times a weak	medium	low	hard	-0.699689278
348	12	1	iran	5.5	subcutances three times a weak	high	medium	hard	-0.787756632
348	12	0	other	2.5	subcutances other days	medium	low	hard	-0.352164436
349	1	0	iran	5.5	mascular once a weak	high	medium	hard	-0.572088286
349	1	1	iran	0.5	subcutances three times a weak	high	medium	easy	-0.72867963
349	2	1	other	0.5	subcutances other days	high	medium	hard	-0.643869086
349	2	0	iran	5.5	subcutances other days	medium	medium	hard	-0.928090082
349	3	0	iran	5.5	mascular once a weak	high	medium	hard	-0.572088286
349	3	1	iran	2.5	subcutances three times a weak	high	medium	easy	-0.646894171
349	4	0	iran	0.5	subcutances three times a weak	medium	low	hard	-0.699689278
349	4	1	other	2.5	mascular once a weak	high	low	hard	0.00383736
349	5	0	iran	0.5	mascular once a weak	medium	low	easy	-0.303092094
349	5	1	other	2.5	subcutances three times a weak	high	low	easy	-0.030902146

349	6	1	other	2.5	subcutances three times a weak	high	low	easy	-0.030902146
349	6	0	other	0.5	subcutances other days	high	medium	easy	-0.462940247
349	7	0	other	0.5	mascular once a weak	medium	medium	hard	-0.900801637
349	7	1	other	5.5	subcutances other days	medium	low	hard	-0.312098058
349	8	0	iran	2.5	mascular once a weak	high	low	hard	-0.0957683
349	8	1	iran	5.5	subcutances three times a weak	high	low	easy	-0.090441429
349	9	1	iran	2.5	subcutances three times a weak	medium	low	hard	-0.617903819
349	9	0	other	0.5	subcutances other days	high	medium	hard	-0.643869086
349	10	1	other	2.5	mascular once a weak	high	low	hard	0.00383736
349	10	0	iran	5.5	mascular once a weak	medium	medium	easy	-0.697626621
349	11	1	other	5.5	subcutances other days	high	medium	easy	-0.34108841
349	11	0	iran	0.5	subcutances three times a weak	medium	low	hard	-0.699689278
349	12	0	iran	5.5	subcutances three times a weak	high	medium	hard	-0.787756632
349	12	1	other	2.5	subcutances other days	medium	low	hard	-0.352164436
350	1	1	iran	5.5	mascular once a weak	high	medium	hard	-0.572088286
350	1	0	iran	0.5	subcutances three times a weak	high	medium	easy	-0.72867963
350	2	0	other	0.5	subcutances other days	high	medium	hard	-0.643869086
350	2	1	iran	5.5	subcutances other days	medium	medium	hard	-0.928090082
350	3	0	iran	5.5	mascular once a weak	high	medium	hard	-0.572088286
350	3	1	iran	2.5	subcutances three times a weak	high	medium	easy	-0.646894171
350	4	1	iran	0.5	subcutances three times a weak	medium	low	hard	-0.699689278
350	4	0	other	2.5	mascular once a weak	high	low	hard	0.00383736
350	5	1	iran	0.5	mascular once a weak	medium	low	easy	-0.303092094
350	5	0	other	2.5	subcutances three times a weak	high	low	easy	-0.030902146
350	6	0	other	2.5	subcutances three times a weak	high	low	easy	-0.030902146
350	6	1	other	0.5	subcutances other days	high	medium	easy	-0.462940247
350	7	0	other	0.5	mascular once a weak	medium	medium	hard	-0.900801637
350	7	1	other	5.5	subcutances other days	medium	low	hard	-0.312098058
350	8	1	iran	2.5	mascular once a weak	high	low	hard	-0.0957683
350	8	0	iran	5.5	subcutances three times a weak	high	low	easy	-0.090441429
350	9	1	iran	2.5	subcutances three times a weak	medium	low	hard	-0.617903819
350	9	0	other	0.5	subcutances other days	high	medium	hard	-0.643869086
350	10	0	other	2.5	mascular once a weak	high	low	hard	0.00383736
350	10	1	iran	5.5	mascular once a weak	medium	medium	easy	-0.697626621

350	11	0	other	5.5	subcutances other days	high	medium	easy	-0.34108841
350	11	1	iran	0.5	subcutances three times a weak	medium	low	hard	-0.699689278
350	12	0	iran	5.5	subcutances three times a weak	high	medium	hard	-0.787756632
350	12	1	other	2.5	subcutances other days	medium	low	hard	-0.352164436
351	1	0	iran	5.5	mascular once a weak	high	medium	hard	-0.572088286
351	1	1	iran	0.5	subcutances three times a weak	high	medium	easy	-0.72867963
351	2	1	other	0.5	subcutances other days	high	medium	hard	-0.643869086
351	2	0	iran	5.5	subcutances other days	medium	medium	hard	-0.928090082
351	3	0	iran	5.5	mascular once a weak	high	medium	hard	-0.572088286
351	3	1	iran	2.5	subcutances three times a weak	high	medium	easy	-0.646894171
351	4	0	iran	0.5	subcutances three times a weak	medium	low	hard	-0.699689278
351	4	1	other	2.5	mascular once a weak	high	low	hard	0.00383736
351	5	0	iran	0.5	mascular once a weak	medium	low	easy	-0.303092094
351	5	1	other	2.5	subcutances three times a weak	high	low	easy	-0.030902146
351	6	1	other	2.5	subcutances three times a weak	high	low	easy	-0.030902146
351	6	0	other	0.5	subcutances other days	high	medium	easy	-0.462940247
351	7	0	other	0.5	mascular once a weak	medium	medium	hard	-0.900801637
351	7	1	other	5.5	subcutances other days	medium	low	hard	-0.312098058
351	8	0	iran	2.5	mascular once a weak	high	low	hard	-0.0957683
351	8	1	iran	5.5	subcutances three times a weak	high	low	easy	-0.090441429
351	9	0	iran	2.5	subcutances three times a weak	medium	low	hard	-0.617903819
351	9	1	other	0.5	subcutances other days	high	medium	hard	-0.643869086
351	10	1	other	2.5	mascular once a weak	high	low	hard	0.00383736
351	10	0	iran	5.5	mascular once a weak	medium	medium	easy	-0.697626621
351	11	1	other	5.5	subcutances other days	high	medium	easy	-0.34108841
351	11	0	iran	0.5	subcutances three times a weak	medium	low	hard	-0.699689278
351	12	1	iran	5.5	subcutances three times a weak	high	medium	hard	-0.787756632
351	12	0	other	2.5	subcutances other days	medium	low	hard	-0.352164436
352	1	1	iran	5.5	mascular once a weak	high	medium	hard	-0.572088286
352	1	0	iran	0.5	subcutances three times a weak	high	medium	easy	-0.72867963
352	2	0	other	0.5	subcutances other days	high	medium	hard	-0.643869086
352	2	1	iran	5.5	subcutances other days	medium	medium	hard	-0.928090082
352	3	0	iran	5.5	mascular once a weak	high	medium	hard	-0.572088286
352	3	1	iran	2.5	subcutances three times a weak	high	medium	easy	-0.646894171

352	4	1	iran	0.5	subcutances three times a weak	medium	low	hard	-0.699689278
352	4	0	other	2.5	mascular once a weak	high	low	hard	0.00383736
352	5	1	iran	0.5	mascular once a weak	medium	low	easy	-0.303092094
352	5	0	other	2.5	subcutances three times a weak	high	low	easy	-0.030902146
352	6	0	other	2.5	subcutances three times a weak	high	low	easy	-0.030902146
352	6	1	other	0.5	subcutances other days	high	medium	easy	-0.462940247
352	7	0	other	0.5	mascular once a weak	medium	medium	hard	-0.900801637
352	7	1	other	5.5	subcutances other days	medium	low	hard	-0.312098058
352	8	1	iran	2.5	mascular once a weak	high	low	hard	-0.0957683
352	8	0	iran	5.5	subcutances three times a weak	high	low	easy	-0.090441429
352	9	1	iran	2.5	subcutances three times a weak	medium	low	hard	-0.617903819
352	9	0	other	0.5	subcutances other days	high	medium	hard	-0.643869086
352	10	0	other	2.5	mascular once a weak	high	low	hard	0.00383736
352	10	1	iran	5.5	mascular once a weak	medium	medium	easy	-0.697626621
352	11	0	other	5.5	subcutances other days	high	medium	easy	-0.34108841
352	11	1	iran	0.5	subcutances three times a weak	medium	low	hard	-0.699689278
352	12	0	iran	5.5	subcutances three times a weak	high	medium	hard	-0.787756632
352	12	1	other	2.5	subcutances other days	medium	low	hard	-0.352164436
353	1	1	iran	5.5	mascular once a weak	high	medium	hard	-0.572088286
353	1	0	iran	0.5	subcutances three times a weak	high	medium	easy	-0.72867963
353	2	1	other	0.5	subcutances other days	high	medium	hard	-0.643869086
353	2	0	iran	5.5	subcutances other days	medium	medium	hard	-0.928090082
353	3	0	iran	5.5	mascular once a weak	high	medium	hard	-0.572088286
353	3	1	iran	2.5	subcutances three times a weak	high	medium	easy	-0.646894171
353	4	0	iran	0.5	subcutances three times a weak	medium	low	hard	-0.699689278
353	4	1	other	2.5	mascular once a weak	high	low	hard	0.00383736
353	5	0	iran	0.5	mascular once a weak	medium	low	easy	-0.303092094
353	5	1	other	2.5	subcutances three times a weak	high	low	easy	-0.030902146
353	6	0	other	2.5	subcutances three times a weak	high	low	easy	-0.030902146
353	6	1	other	0.5	subcutances other days	high	medium	easy	-0.462940247
353	7	0	other	0.5	mascular once a weak	medium	medium	hard	-0.900801637
353	7	1	other	5.5	subcutances other days	medium	low	hard	-0.312098058
353	8	1	iran	2.5	mascular once a weak	high	low	hard	-0.0957683
353	8	0	iran	5.5	subcutances three times a weak	high	low	easy	-0.090441429

353	9	1	iran	2.5	subcutances three times a weak	medium	low	hard	-0.617903819
353	9	0	other	0.5	subcutances other days	high	medium	hard	-0.643869086
353	10	0	other	2.5	mascular once a weak	high	low	hard	0.00383736
353	10	1	iran	5.5	mascular once a weak	medium	medium	easy	-0.697626621
353	11	0	other	5.5	subcutances other days	high	medium	easy	-0.34108841
353	11	1	iran	0.5	subcutances three times a weak	medium	low	hard	-0.699689278
353	12	0	iran	5.5	subcutances three times a weak	high	medium	hard	-0.787756632
353	12	1	other	2.5	subcutances other days	medium	low	hard	-0.352164436
354	1	0	iran	5.5	mascular once a weak	high	medium	hard	-0.572088286
354	1	1	iran	0.5	subcutances three times a weak	high	medium	easy	-0.72867963
354	2	1	other	0.5	subcutances other days	high	medium	hard	-0.643869086
354	2	0	iran	5.5	subcutances other days	medium	medium	hard	-0.928090082
354	3	0	iran	5.5	mascular once a weak	high	medium	hard	-0.572088286
354	3	1	iran	2.5	subcutances three times a weak	high	medium	easy	-0.646894171
354	4	0	iran	0.5	subcutances three times a weak	medium	low	hard	-0.699689278
354	4	1	other	2.5	mascular once a weak	high	low	hard	0.00383736
354	5	0	iran	0.5	mascular once a weak	medium	low	easy	-0.303092094
354	5	1	other	2.5	subcutances three times a weak	high	low	easy	-0.030902146
354	6	0	other	2.5	subcutances three times a weak	high	low	easy	-0.030902146
354	6	1	other	0.5	subcutances other days	high	medium	easy	-0.462940247
354	7	0	other	0.5	mascular once a weak	medium	medium	hard	-0.900801637
354	7	1	other	5.5	subcutances other days	medium	low	hard	-0.312098058
354	8	1	iran	2.5	mascular once a weak	high	low	hard	-0.0957683
354	8	0	iran	5.5	subcutances three times a weak	high	low	easy	-0.090441429
354	9	0	iran	2.5	subcutances three times a weak	medium	low	hard	-0.617903819
354	9	1	other	0.5	subcutances other days	high	medium	hard	-0.643869086
354	10	0	other	2.5	mascular once a weak	high	low	hard	0.00383736
354	10	1	iran	5.5	mascular once a weak	medium	medium	easy	-0.697626621
354	11	0	other	5.5	subcutances other days	high	medium	easy	-0.34108841
354	11	1	iran	0.5	subcutances three times a weak	medium	low	hard	-0.699689278
354	12	0	iran	5.5	subcutances three times a weak	high	medium	hard	-0.787756632
354	12	1	other	2.5	subcutances other days	medium	low	hard	-0.352164436
355	1	0	iran	5.5	mascular once a weak	high	medium	hard	-0.572088286
355	1	0	iran	0.5	subcutances three times a weak	high	medium	easy	-0.72867963

355	2	1	other	0.5	subcutances other days	high	medium	hard	-0.643869086
355	2	0	iran	5.5	subcutances other days	medium	medium	hard	-0.928090082
355	3	0	iran	5.5	mascular once a weak	high	medium	hard	-0.572088286
355	3	0	iran	2.5	subcutances three times a weak	high	medium	easy	-0.646894171
355	4	0	iran	0.5	subcutances three times a weak	medium	low	hard	-0.699689278
355	4	1	other	2.5	mascular once a weak	high	low	hard	0.00383736
355	5	0	iran	0.5	mascular once a weak	medium	low	easy	-0.303092094
355	5	1	other	2.5	subcutances three times a weak	high	low	easy	-0.030902146
355	6	1	other	2.5	subcutances three times a weak	high	low	easy	-0.030902146
355	6	0	other	0.5	subcutances other days	high	medium	easy	-0.462940247
355	7	0	other	0.5	mascular once a weak	medium	medium	hard	-0.900801637
355	7	1	other	5.5	subcutances other days	medium	low	hard	-0.312098058
355	8	0	iran	2.5	mascular once a weak	high	low	hard	-0.0957683
355	8	0	iran	5.5	subcutances three times a weak	high	low	easy	-0.090441429
355	9	0	iran	2.5	subcutances three times a weak	medium	low	hard	-0.617903819
355	9	1	other	0.5	subcutances other days	high	medium	hard	-0.643869086
355	10	1	other	2.5	mascular once a weak	high	low	hard	0.00383736
355	10	0	iran	5.5	mascular once a weak	medium	medium	easy	-0.697626621
355	11	1	other	5.5	subcutances other days	high	medium	easy	-0.34108841
355	11	0	iran	0.5	subcutances three times a weak	medium	low	hard	-0.699689278
355	12	1	iran	5.5	subcutances three times a weak	high	medium	hard	-0.787756632
355	12	0	other	2.5	subcutances other days	medium	low	hard	-0.352164436
356	1	0	iran	5.5	mascular once a weak	high	medium	hard	-0.572088286
356	1	1	iran	0.5	subcutances three times a weak	high	medium	easy	-0.72867963
356	2	1	other	0.5	subcutances other days	high	medium	hard	-0.643869086
356	2	0	iran	5.5	subcutances other days	medium	medium	hard	-0.928090082
356	3	0	iran	5.5	mascular once a weak	high	medium	hard	-0.572088286
356	3	1	iran	2.5	subcutances three times a weak	high	medium	easy	-0.646894171
356	4	0	iran	0.5	subcutances three times a weak	medium	low	hard	-0.699689278
356	4	1	other	2.5	mascular once a weak	high	low	hard	0.00383736
356	5	0	iran	0.5	mascular once a weak	medium	low	easy	-0.303092094
356	5	1	other	2.5	subcutances three times a weak	high	low	easy	-0.030902146
356	6	1	other	2.5	subcutances three times a weak	high	low	easy	-0.030902146
356	6	0	other	0.5	subcutances other days	high	medium	easy	-0.462940247

356	7	0	other	0.5	mascular once a weak	medium	medium	hard	-0.900801637
356	7	1	other	5.5	subcutances other days	medium	low	hard	-0.312098058
356	8	0	iran	2.5	mascular once a weak	high	low	hard	-0.0957683
356	8	1	iran	5.5	subcutances three times a weak	high	low	easy	-0.090441429
356	9	1	iran	2.5	subcutances three times a weak	medium	low	hard	-0.617903819
356	9	0	other	0.5	subcutances other days	high	medium	hard	-0.643869086
356	10	1	other	2.5	mascular once a weak	high	low	hard	0.00383736
356	10	0	iran	5.5	mascular once a weak	medium	medium	easy	-0.697626621
356	11	1	other	5.5	subcutances other days	high	medium	easy	-0.34108841
356	11	0	iran	0.5	subcutances three times a weak	medium	low	hard	-0.699689278
356	12	1	iran	5.5	subcutances three times a weak	high	medium	hard	-0.787756632
356	12	0	other	2.5	subcutances other days	medium	low	hard	-0.352164436
357	1	1	iran	5.5	mascular once a weak	high	medium	hard	-0.572088286
357	1	0	iran	0.5	subcutances three times a weak	high	medium	easy	-0.72867963
357	2	1	other	0.5	subcutances other days	high	medium	hard	-0.643869086
357	2	0	iran	5.5	subcutances other days	medium	medium	hard	-0.928090082
357	3	0	iran	5.5	mascular once a weak	high	medium	hard	-0.572088286
357	3	1	iran	2.5	subcutances three times a weak	high	medium	easy	-0.646894171
357	4	0	iran	0.5	subcutances three times a weak	medium	low	hard	-0.699689278
357	4	1	other	2.5	mascular once a weak	high	low	hard	0.00383736
357	5	0	iran	0.5	mascular once a weak	medium	low	easy	-0.303092094
357	5	1	other	2.5	subcutances three times a weak	high	low	easy	-0.030902146
357	6	1	other	2.5	subcutances three times a weak	high	low	easy	-0.030902146
357	6	0	other	0.5	subcutances other days	high	medium	easy	-0.462940247
357	7	0	other	0.5	mascular once a weak	medium	medium	hard	-0.900801637
357	7	1	other	5.5	subcutances other days	medium	low	hard	-0.312098058
357	8	1	iran	2.5	mascular once a weak	high	low	hard	-0.0957683
357	8	0	iran	5.5	subcutances three times a weak	high	low	easy	-0.090441429
357	9	1	iran	2.5	subcutances three times a weak	medium	low	hard	-0.617903819
357	9	0	other	0.5	subcutances other days	high	medium	hard	-0.643869086
357	10	1	other	2.5	mascular once a weak	high	low	hard	0.00383736
357	10	0	iran	5.5	mascular once a weak	medium	medium	easy	-0.697626621
357	11	0	other	5.5	subcutances other days	high	medium	easy	-0.34108841
357	11	1	iran	0.5	subcutances three times a weak	medium	low	hard	-0.699689278

357	12	0	iran	5.5	subcutances three times a weak	high	medium	hard	-0.787756632
357	12	1	other	2.5	subcutances other days	medium	low	hard	-0.352164436
358	1	0	iran	5.5	mascular once a weak	high	medium	hard	-0.572088286
358	1	1	iran	0.5	subcutances three times a weak	high	medium	easy	-0.72867963
358	2	1	other	0.5	subcutances other days	high	medium	hard	-0.643869086
358	2	0	iran	5.5	subcutances other days	medium	medium	hard	-0.928090082
358	3	1	iran	5.5	mascular once a weak	high	medium	hard	-0.572088286
358	3	0	iran	2.5	subcutances three times a weak	high	medium	easy	-0.646894171
358	4	0	iran	0.5	subcutances three times a weak	medium	low	hard	-0.699689278
358	4	1	other	2.5	mascular once a weak	high	low	hard	0.00383736
358	5	1	iran	0.5	mascular once a weak	medium	low	easy	-0.303092094
358	5	0	other	2.5	subcutances three times a weak	high	low	easy	-0.030902146
358	6	0	other	2.5	subcutances three times a weak	high	low	easy	-0.030902146
358	6	1	other	0.5	subcutances other days	high	medium	easy	-0.462940247
358	7	0	other	0.5	mascular once a weak	medium	medium	hard	-0.900801637
358	7	1	other	5.5	subcutances other days	medium	low	hard	-0.312098058
358	8	1	iran	2.5	mascular once a weak	high	low	hard	-0.0957683
358	8	0	iran	5.5	subcutances three times a weak	high	low	easy	-0.090441429
358	9	1	iran	2.5	subcutances three times a weak	medium	low	hard	-0.617903819
358	9	0	other	0.5	subcutances other days	high	medium	hard	-0.643869086
358	10	1	other	2.5	mascular once a weak	high	low	hard	0.00383736
358	10	0	iran	5.5	mascular once a weak	medium	medium	easy	-0.697626621
358	11	1	other	5.5	subcutances other days	high	medium	easy	-0.34108841
358	11	0	iran	0.5	subcutances three times a weak	medium	low	hard	-0.699689278
358	12	0	iran	5.5	subcutances three times a weak	high	medium	hard	-0.787756632
358	12	1	other	2.5	subcutances other days	medium	low	hard	-0.352164436
